# Copper(I)-Catalyzed Intramolecular Tandem Acylation/*O*-Arylation under Mild Conditions: Synthesis of Benzofuro[3,2-*c*]quinolin-6(5*H*)-ones and G-Quadruplex-Targeting Analogues

**DOI:** 10.1021/acs.joc.4c02813

**Published:** 2024-12-27

**Authors:** Show-Jen Chiou, Yi-Chien Lin, Yi-Fu Chang, Yu-Fen Chen, Tzu-Hao Lo, Chia-Shen Tsai, Hung-Chun Liao, Pei-Yi Tsai, Shih-Hsien Chuang, Jiann-Jyh Huang

**Affiliations:** † Institute of BioPharmaceutical Sciences, National Sun Yat-sen University, No. 70, Lien-hai Road, Kaohsiung 804201, Taiwan; ‡ Department of Applied Chemistry, National Chiayi University, No. 300, Syuefu Road, Chiayi 60004, Taiwan; § Development Center for Biotechnology, National Biotechnology Research Park, Taipei 11571, Taiwan

## Abstract

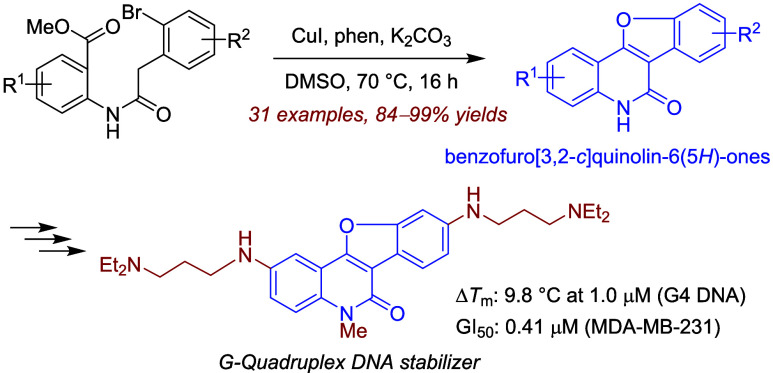

This paper presents a copper(I)-catalyzed intramolecular tandem acylation/*O*-arylation of methyl 2-[2-(2-bromophenyl)acetamido]benzoates for the synthesis of benzofuro[3,2-*c*]quinolin-6(5*H*)-ones under mild conditions. The combination of CuI, 1,10-phenanthroline, and K_2_CO_3_ in DMSO was found to be the optimal reaction condition, producing the target products in high yields (84–99%) at 70 °C for 16 h. The tandem reaction was applicable to substrates bearing halo, electron-withdrawing, and electron-donating groups at their phenyl moieties with a broad substrate scope. Further derivation produced compounds serving as G-quadruplex DNA (G4 DNA) stabilizers. The most potent analogue, 2,9-bis{[3-(diethylamino)propyl]amino}-5-methylbenzofuro[3,2-*c*]quinolin-6(5*H*)-one, significantly increased the melting temperature of G4 DNA by 9.8 °C at 1.0 μM, approximately 4.6 times more selective than duplex DNA. The G4 stabilizer also showed anticancer activity, actively inhibiting MDA-MB-231 cancer cells with a GI_50_ value of 0.41 μM.

## Introduction

**Figure 1:**
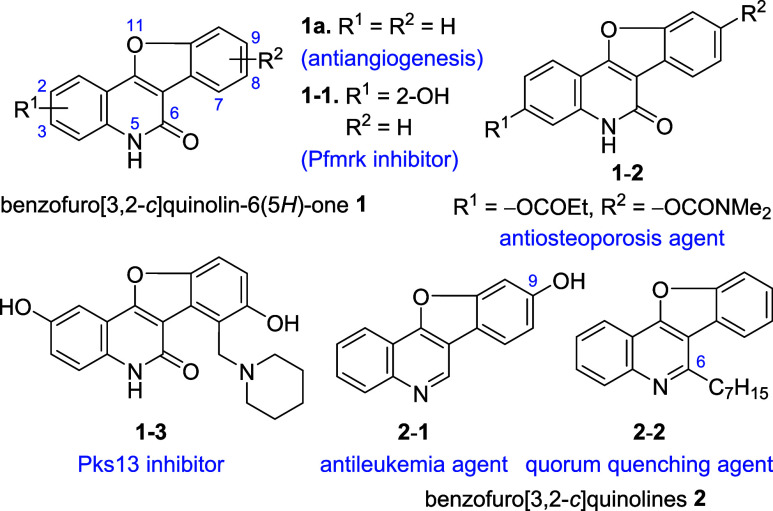
Biologically active benzofuro[3,2-*c*]quinolin-6(5*H*)-ones **1** and benzofuro[3,2-*c*]quinolines **2**.

Tetracyclic benzofuro[3,2-*c*]quinolin-6(5*H*)-one **1** is a subclass of benzofuroquinolinone known for its diverse biological activities (Figure 1). Even unsubstituted **1a** inhibits angiogenesis^1^ and simple monohydroxylated **1-1** inhibits *Plasmodium falciparum* cyclin-dependent kinase (Pfmrk).^2^ Disubstituted **1-2** with propanoyloxy and dimethylcarbamoyloxy groups exhibits antiosteoporosis activity^3^ and trisubstituted **1-3** with hydroxyl and piperidin-1-ylmethyl groups inhibits polyketide synthase 13 (Pks13).^4^ Furthermore, several benzofuro[3,2-*c*]quinolines **2** which can be readily prepared from **1** are also biologically active (Figure 1). Representative examples include benzofuroquinolines **2-1** with a C-9 hydroxyl group as an antileukemia agent^5^ and **2-2** with a C-6 heptyl substituent as a quorum quenching agent.^6^ These findings motivated us to investigate the synthesis and potential new biological applications of benzofuroquinolinone **1**.

The syntheses of benzofuro[3,2-*c*]quinolin-6(5*H*)-one **1** have been reported in over ten publications.^2,7−19^ However, only a few methods provide diverse derivatives. Patel and colleagues first reported its systematic synthesis through a copper-catalyzed tandem reaction of 2-[(2-bromophenyl)ethynyl]anilines using Cs_2_CO_3_ as the carbonyl and oxygen source.^16^ Also employing a tandem strategy,^20,21^ Jiang’s group cyclized 2-hydroxy-2′-amino-diphenylethyne via a palladium(II)-catalyzed carbonylative reaction to synthesize *N*-substituted derivatives.^17^ Ning and his colleagues cyclized 3-chlorooxindoles with imines to form dihydrobenzofuran spirooxindoles, which were subsequently transformed into **1** by trifluoromethanesulfonic acid.^19^ Although these novel synthetic methods can produce various **1** with broad substrate scopes, the preparation of their starting materials is not straightforward, which may limit their practical use. In addition, the yields of these reactions are generally less than 90%, which could lead to challenges in purification due to the low solubility of **1** in common organic solvents. Therefore, a concise synthesis could be valuable for preparing benzofuroquinolinone **1** and exploring its biological applications.

**Figure 2:**
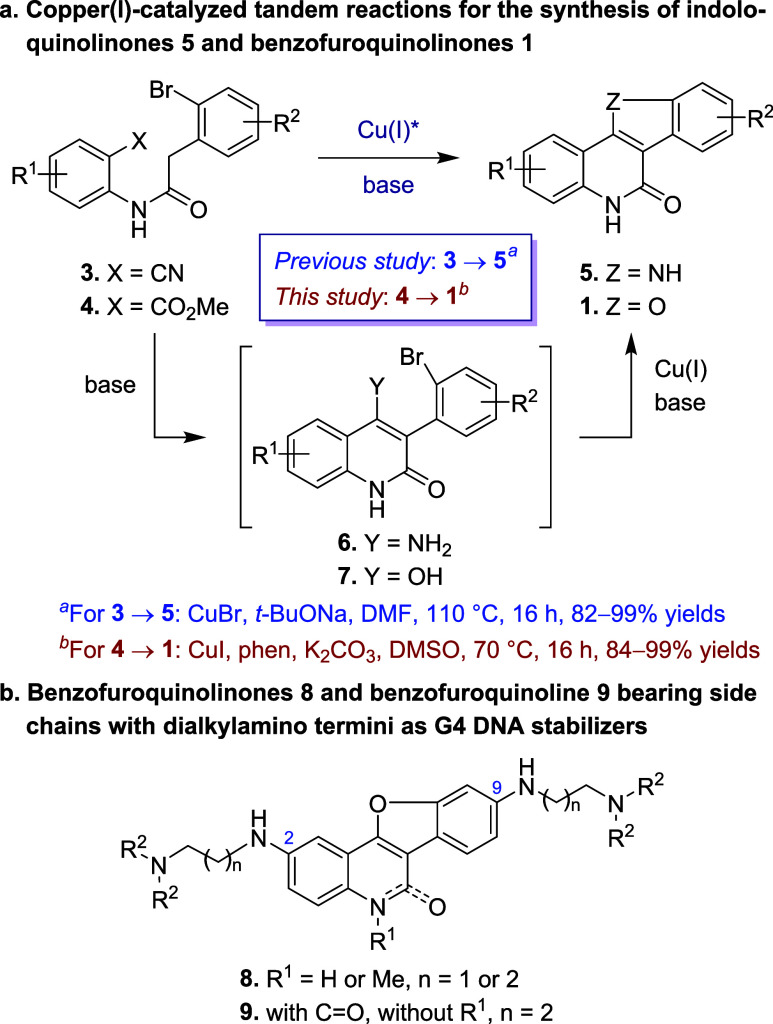
(a) Copper(I)-catalyzed tandem reactions for the synthesis of indoloquinolinones **5** and benzofuroquinolinones **1**. (b) The structures of G4 DNA stabilizers **8** and **9** reported in this work.

We have developed a tandem reaction for the synthesis of indolo[3,2-*c*]quinolinone **5**, a structurally similar analogue of benzofuroquinolinone **1**, using 2-(2-bromophenyl)-*N*-(2-cyanophenyl)acetamide **3** as the starting material (Figure 2a).^22^ The reaction starts with the intramolecular addition of the acetamide enolate to the nitrile, forming 4-aminoquinolinone **6**, followed by copper(I)-catalyzed *N*-arylation to produce **5**. Although the reaction has a broad substrate scope, it requires harsh conditions, including high reaction temperature (110 °C), a strong base (*t*-BuONa), and anhydrous DMF. To synthesize **1**, we hypothesized that a similar tandem reaction might occur in methyl 2-[2-(2-bromophenyl)acetamido]benzoate **4** (Figure 2a), the ester analogue of substrate **3**, but under milder conditions as esters are more reactive than nitriles. The proposed tandem reaction would begin with the acylation of the enolate of **4** by the neighboring ester group to form 4-hydroxyquinolinone **7**. Subsequent copper(I)-catalyzed *O*-arylation would transform **7** into target benzofuroquinolinone **1**.

Herein, we present copper(I)-catalyzed intramolecular tandem acylation/*O*-arylation for the synthesis of benzofuro[3,2-*c*]quinolin-6(5*H*)-one **1** (Figure 2a). The reaction produced various **1** in high yields and demonstrated a broad substrate scope under mild conditions, using the weaker base K_2_CO_3_ at a moderate temperature of 70 °C. The starting material **4** was prepared via a straightforward amidation reaction using SOCl_2_. Subsequently, we grafted **1** with two side chains bearing dialkylamino termini at the C-2 and C-9 positions. The resulting benzofuroquinolinones **8** and benzofuroquinoline **9** (Figure 2b) were found to stabilize G-quadruplex DNA (G4 DNA),^23,24^ the secondary structures formed in guanine-rich human telomeres and oncogene promoters.^25^ Alteration of G4 DNA stability and function has emerged as a promising drug target for cancer therapy.^26−29^ Therefore, this study achieved a new concise synthesis of **1** using a tandem strategy under mild conditions and demonstrated a new biological application of benzofuroquinolinone **1** and benzofuroquinoline **2** (Figure 1) to stabilize G4 DNA through their derivatives **8** and **9** (Figure 2b).

## Results and Discussion

### Optimization of the Reaction Conditions for the Tandem Reaction

Table 1 presents the optimization of the tandem reaction by using unsubstituted **4a** as the starting material. Compound **4a** was first treated with the optimal reaction conditions for substrate **3** to form indoloquinolinones **5** (CuI and *t*-BuONa in anhydrous DMF, Figure 2a) at 110 °C for 16 h.^22^ The reaction gave target **1a** in 82% yield (entry 1). Replacing CuBr with CuI (entry 2) or Cu_2_O (entry 3) resulted in inferior yields (63 and 56%). Using CuI with weaker bases K_3_PO_4_ (entry 4) or K_2_CO_3_ (entry 5) in DMF led to even lower yields of 50 and 32%, respectively. In contrast, CuI with the strong base NaH in DMF gave **1a** in 84% yield (entry 6), the highest conversion observed among the reactions conducted in DMF at 110 °C for 16 h (entries 1–6).

**Table 1:**
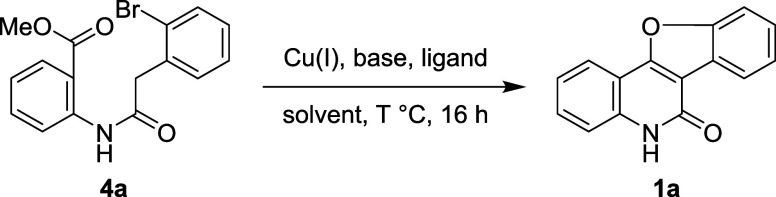
Optimization of the Reaction Conditions^a^

entry	Cu(I)	base	ligand^b^	solvent	*T* (°C)^c^	yield (%)^d^
1	CuBr	*t*-BuONa	—	DMF^e^	110	82
2	CuI	*t*-BuONa	—	DMF^e^	110	63
3	Cu_2_O	*t*-BuONa	—	DMF^e^	110	56
4	CuI	K_3_PO_4_	—	DMF	110	50
5	CuI	K_2_CO_3_	—	DMF	110	32
6	CuI	NaH	—	DMF^e^	110	84
7	CuI	K_2_CO_3_	—	CH_3_CN	70	0^f^
8	CuI	K_2_CO_3_	—	DMSO	70	57
9	CuI	Cs_2_CO_3_	—	DMSO	70	32
10	CuI	K_2_CO_3_	—	DMSO	110	90
11	CuBr	K_2_CO_3_	—	DMSO	110	66
12	CuI	Cs_2_CO_3_	L1	DMSO	70	65
13	CuI	Cs_2_CO_3_	L2	DMSO	70	71
14	CuI	K_2_CO_3_	L3	DMSO	70	44
15	CuI	K_3_PO_4_	L4	DMSO	70	9.0
16	CuI	K_3_PO_4_	L4	DMF	70	82
17	Cu_2_O	Cs_2_CO_3_	L5	DMSO	70	49
18	Cu_2_O	Cs_2_CO_3_	L5	CH_3_CN	70	0^f^
19	CuI	K_2_CO_3_	L6	DMSO	70	80
20	CuI	K_2_CO_3_	L7	DMSO	70	99 (98^g^)
21	CuI	K_2_CO_3_	L7	DMSO	60	94
22	CuI	K_2_CO_3_	L7	DMSO	50	84
23	CuI	K_2_CO_3_	L7	DMSO	40	69
24	CuI	K_2_CO_3_	L7	DMSO	r.t.	60

a The reaction was carried out using **4a** (∼100 mg), Cu(I) catalyst (5.0 mol %), base (2.1 equiv), and ligand (10 mol %) in 2.0 mL of solvent for 16 h.

b Ligands: L1, ethylenediamine; L2, 1,2-dimethylethylenediamine; L3, l-proline; L4, 2-methoxybenzoic acid methyl ester; L5, imidazole-4-carboxylic acid; L6, *trans*-*N,N*′-dimethylcyclohexane-1,2-diamine; and L7, 1,10-phenanthroline (phen). The structures of these ligands are shown in Figure 3.

c Oil bath temperature.

d NMR yield, using 1,3,5-trimethoxybenzene as the reference standard.

e Anhydrous DMF.

f Undetectable in NMR.

g Isolated yield.

We assumed that substrate **4** was converted to product **1** through the intermediate 4-hydroxyquinolinone **7** via an intramolecular acylation reaction (Figure 2a). Therefore, polar aprotic solvents such as CH_3_CN and DMSO, in addition to DMF (entries 1–6), were expected to facilitate this polar acylation reaction. However, the combination of CuI with K_2_CO_3_ did not produce **1a** in CH_3_CN at 70 °C (entry 7), whereas the same combination in DMSO gave **1a** in 57% yield (entry 8). Replacing K_2_CO_3_ (entry 8) with Cs_2_CO_3_ (entry 9) decreased the yield to 32%. When the reaction temperature was increased to 110 °C, CuI with K_2_CO_3_ in DMSO had a better yield (90%, entry 10) compared to CuBr under the same conditions (66%, entry 11). Notably, using DMSO as the solvent resulted in a significantly higher yield (90%, entry 10) compared to that of DMF (32%, entry 5). This improvement may be attributed to the faster formation of acylation product **7** in the highly polar DMSO.

To further optimize the tandem reaction, we combined Cu(I) catalysts with a series of bidentate ligands (Figure 3) in polar aprotic solvents (DMSO, DMF, and CH_3_CN) at 70 °C. In DMSO, ethylenediamine (L1) with CuI and Cs_2_CO_3_ produced **1a** in 65% yield (entry 12). 1,2-Dimethylethylenediamine (L2) achieved a higher yield (71%, entry 13). The combination of l-proline (L3) with CuI and K_2_CO_3_ gave **1a** in a 44% yield (entry 14). A poor yield of 9.0% was observed when 2-methoxybenzoic acid methyl ester (L4) was used with CuI and K_3_PO_4_ in DMSO (entry 15). However, the same combination of L4, CuI, and K_3_PO_4_ in DMF gave a significantly higher yield of **1a** (82%, entry 16). Imidazole-4-carboxylic acid (L5) with Cu_2_O and Cs_2_CO_3_ produced **1a** in 49% yield in DMSO (entry 17), but the combination failed in CH_3_CN (0% yield, entry 18). With *trans*-*N*,*N*′-dimethylcyclohexane-1,2-diamine (L6), CuI, and K_2_CO_3_, **1a** was generated in 80% yield (entry 19).

**Figure 3:**
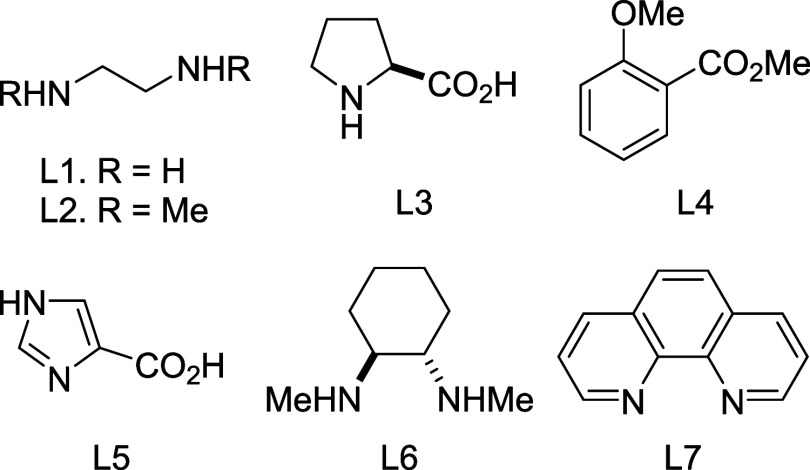
Structures of the ligands used for the tandem reaction.

Using 1,10-phenanthroline (phen, L7), CuI, and K_2_CO_3_ in DMSO proved to be the most efficient reaction conditions for the tandem reaction, giving **1a** in 99% NMR yield. Due to the high conversion, **1a** was obtained in 98% yield after simple purification (precipitation in water followed by recrystallization in methanol). The combination also produced **1a** in good yield at lower temperatures (entries 21–24). At 60 °C, 50 °C, 40 °C, and even room temperature, product **1a** was generated in 94, 84, 69, and 60% yields, respectively. Therefore, the optimal reaction conditions for the tandem reaction involve CuI as the catalyst, K_2_CO_3_ as the base, phen as the ligand, and DMSO as the solvent.

### Substrate Scope of the Tandem Reaction

**Scheme 1:**
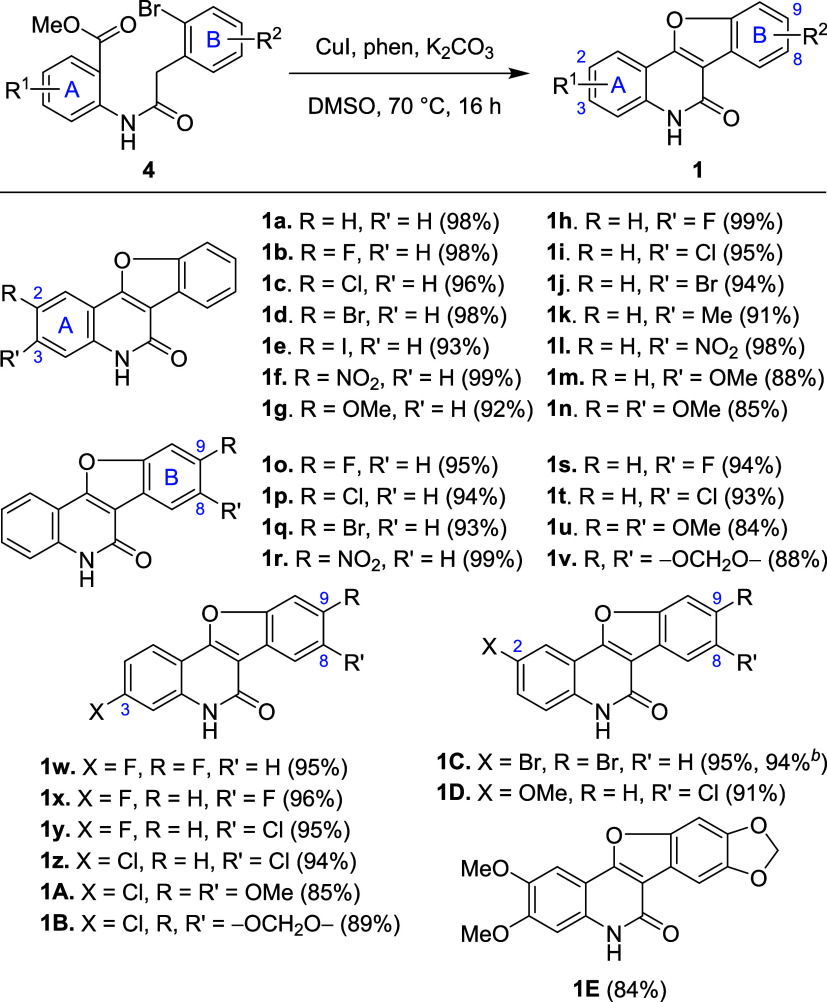
Substrate Scope of the Tandem Reaction The reaction was carried out by using **4** (100 mg), CuI (5.0 mol %), phen (10 mol %), and K_2_CO_3_ (2.1 equiv) in DMSO (2.0 mL) at 70 °C for 16 h. Using 2.0 g of the starting material.

The optimal reaction conditions (entry 20, Table 1) were applied to substrate **4** to synthesize benzofuro[3,2-*c*]quinolin-6(5*H*)-one **1** bearing various substituents at its two phenyl moieties (A- and B-rings, see Scheme 1) at 70 °C. For the synthesis of **1b**–**e** bearing a single halo group (F, Cl, Br, I) at the C-2 position (A-ring), the yields were high (93–98%), similar to that of unsubstituted **1a** (98%). The reaction also gave **1f** bearing a C-2 nitro group in a high yield (99%). In contrast, nitro-substituted indoloquinolinone **5**, analogous to **1f**, could only be produced in less than 10% yield from **3** via the tandem reaction described in our work (Figure 2a).^22^ The low yield for nitro substrates **3** to form **5** may result from the coordination of the nitro group to Cu(I).^30^ In the conversion of **4** to **1** reported herein, the bidentate ligand phen prevented Cu(I) from binding to the nitro group. Nitro-containing **1f** was thus obtained in a high yield. For the synthesis of **1g** with a C-2 methoxy substituent, the yield was slightly reduced to 92%. Compound **1g** is known to undergo a one-step demethylation reaction to produce Pfmrk inhibitor **1-1** (Figure 1).^19^

For the synthesis of **1h**–**j** bearing a single fluoro, chloro, or bromo group at the C-3 position (A-ring), the yields of the tandem reaction remained high (94–99%, see Scheme 1). For **1k** with a C-3 methyl substituent, the yield slightly decreased to 91%. Compound **1l** with a C-3 nitro group was also produced in high yield (98%), similar to its C-2 analogue **1f**. Like **1g** having a C-2 methoxy substituent, **1m** with the same substituent at the C-3 position showed a slightly lower yield (88%). The presence of two methoxy groups further deactivated the reaction, producing **1n** in an 85% yield. For substrates **4** having substituents at the B-ring, the optimal reaction conditions provided **1o**–**r** having one halo or one nitro group at the C-9 position in high yields (93–99%). Compounds **1s** and **1t** with one C-8 halo group (F or Cl) were also obtained in good yields (94 and 93%). Similar to methoxy-bearing **1g**, **1m**, and **1n** on the A-ring, alkoxy groups on the B-ring deactivated the reaction. Compounds **1u** with a dimethoxy group and **1v** with a methylenedioxy group were obtained in 84 and 88% yields, respectively.

The tandem reaction also showed good reactivity to give benzofuroquinolinones **1w**–**z** and **1A**–**E** bearing substituents on both the A- and B-rings. Products **1w**–**z**, each having one halo group at the C-3 position (F or Cl, A-ring) and one halo group at the C-8 or C-9 position (F or Cl, B-ring), were obtained in 94–96% yields. The yields for C-3 chloro-containing **1A** and **1B**, bearing electron-donating groups (dimethoxy or methylenedioxy) at the B-ring, decreased to 85 and 89%, respectively. These yields are very close to those for **1u** (88%) and **1v** (85%), which have the same substituents at the B-ring but lack the C-3 chloro groups. These results suggest that the halo group did not promote the tandem reaction. For **1C** containing two bromo groups at the C-2 and C-9 positions, the reaction showed good reactivity (95% yield). When 2.0 g of the starting material were used, **1C** was also isolated in a nearly identical yield (94%), demonstrating the applicability of the tandem reaction for larger-scale synthesis. The yield of **1D** having a C-2 methoxy group and a C-8 chloro group was 91%, which is very close to that of **1g** (92% yield) without the chloro group. The reaction was less reactive for **1E** having a dimethoxy group at the A-ring and a methylenedioxy group at the B-ring (84% yield).

Cumulatively, the optimal reaction conditions (CuI, K_2_CO_3_, phen, DMSO, 70 °C, 16 h) afforded compounds bearing halo and electron-withdrawing groups in very high yields (93–99%, Scheme 1). Although electron-donating groups (e.g., methyl, methoxy, and methylenedioxy) slightly deactivated the reaction, the corresponding products were still obtained in good yields (>84%). Consequently, **1** was purified by precipitation and recrystallization, obviating the need for column chromatography. The tandem reaction thus demonstrated a good substrate scope for the efficient synthesis of benzofuro[3,2-*c*]quinolin-6(5*H*)-ones **1** accommodating halo, electron-withdrawing, and electron-donating groups.

### Mechanistic Study of the Tandem Reaction

**Scheme 2:**
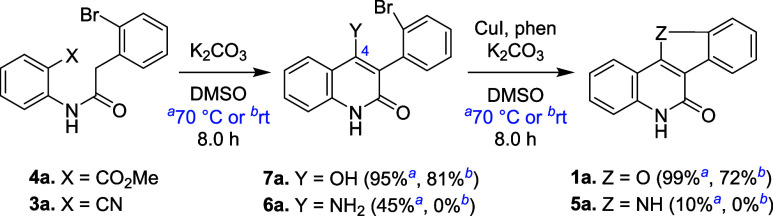
Control Experiments for the Mechanistic Study

Unsubstituted **4a** served as the model substrate to explore the reaction mechanism of the tandem reaction (Scheme 2). When **4a** was heated with K_2_CO_3_ in DMSO at 70 °C for 8.0 h, the intermediate 4-hydroxyquinolinone **7a** was either detected by proton NMR (without workup) or isolated in 95% yield. Subsequent addition of CuI and phen, followed by further heating at 70 °C for 8.0 h, also produced product **1a**. When isolated **7a** was treated with CuI/phen/K_2_CO_3_ in DMSO at 70 °C for 8.0 h, **1a** was obtained in 99% yield. Even at room temperature, the reactions gave **7a** and **1a** in 81 and 72% yields, respectively (Scheme 2). These control experiments confirmed that a tandem acylation/*O*-arylation occurred on substrates **4** to form benzofuroquinolinones **1** through intermediate **7** under mild conditions (Figure 2a). The reaction mechanism could account for the slightly reduced yields of **1** from substrates containing methyl, methoxy, and methylenedioxy groups (Scheme 1). The acidity of the α-proton adjacent to the carbonyl, the reactivity of the ester group, and the reactivity of *O*-arylation were reduced by these election-donating groups, which collectively decreased the overall reactivity of the tandem reaction.

Compared with ester substrate **4a**, nitrile **3a** was less reactive toward K_2_CO_3_ in DMSO (Scheme 2). The corresponding 4-aminoquinolinone **6a** was generated in 45% yield at 70 °C but was not formed at room temperature (0% yield) after reacting for 8.0 h. When the isolated **6a** was reacted with CuI/phen/K_2_CO_3_ in DMSO at 70 or 0 °C for 8.0 h, the yields for indoloquinolinone **5a** were 10 and 0%, respectively. As a result, ester **4a** was more reactive than **3a** in both the first step (forming **7a**) and the final arylation step (forming **1a**). The higher reactivity of *O*-arylation (**7a** → **1a**) compared to that of *N*-arylation (**6a** → **5a**) under the same reaction conditions was attributed to the more nucleophilic C-4 oxide in intermediate **7a** under basic conditions.

### Design and Synthesis of G4 DNA Stabilizers **8a**–**f** and **9**

Polyaromatic compounds with side chains containing amino groups form a distinct class of G4 DNA stabilizers.^31−35^ The aryl-heteroaryl planes of these compounds engage in π–π stacking with the nucleobases of G4 DNA. Additionally, the positively charged amino groups, under physiological pH conditions, interact with the negatively charged phosphate backbone. BRACO-19^36^ and PIPER^37^ are prototypes of this class of G4 DNA stabilizers. Structurally related to benzofuroquinolinones **8** and benzofuroquinoline **9** (Figure 2b), indoloquinolines with amino side chains have been widely reported as potent G4 DNA stabilizers.^38−41^ Compounds **8** and **9** were therefore anticipated to stabilize the G4 DNA.

**Scheme 3:**
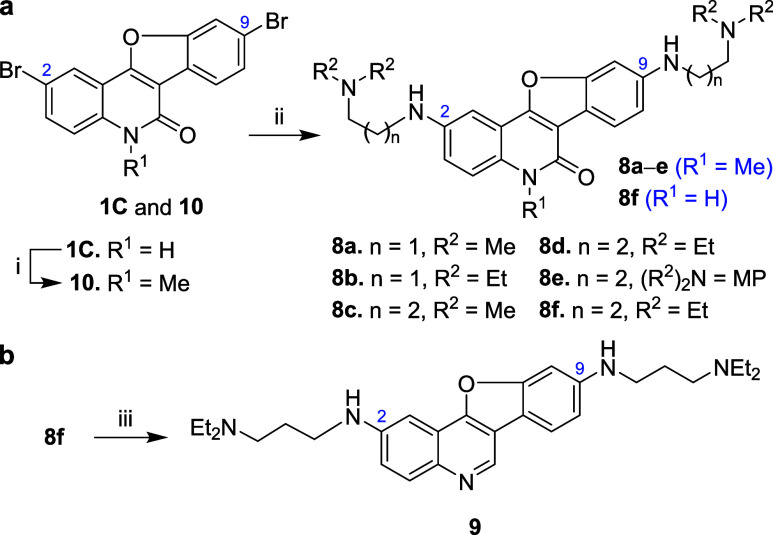
Synthesis of G4 DNA Stabilizers **8a**–**f** and **9** Reagents and conditions: (i) MeI, NaH, DMF, 45 °C, 16 h, 82%; (ii) (R^2^)_2_NCH_2_(CH_2_)_n_NH_2_, Pd(OAc)_2_, CyJohnPhos, *t*-BuONa, DME, *t*-BuOH, reflux, 39 h; **8a** (75%), **8b** (76%), **8c** (70%), **8d** (88%), **8e** (80%), and **8f** (75%); MP stands for *N*-morpholino; (iii) Red-Al, toluene, reflux, 24 h, 88%.

Scheme 3 presents the synthesis of **8** and **9** using 2,9-dibromobenzofuro[3,2-*c*]quinolin-6(5*H*)-one (**1C**, prepared from Scheme 1) as the starting material. Compound **1C** was *N*-methylated with methyl iodide and sodium hydride in DMF to produce **10** in 82% yield (Scheme 3a). Compounds **1C** and **10** were then coupled with various *N*,*N*-dialkylated alkanediamines using Pd(OAc)_2_, CyJohnPhos, and *t*-BuONa in DME and *t*-BuOH.^40^ The reaction gave the corresponding benzofuroquinolinones **8a**–**f** with different side chains containing dialkylamino termini at the C-2 and C-9 positions in 70–88% yields. Further reduction of **8f** with Red-Al produced benzofuroquinoline **9** bearing two 3-(diethylamino)propylamino side chains in 88% yield (Scheme 3b).

### Stabilization of G4 DNA by **8a**–**f** and **9**

**Figure 4:**
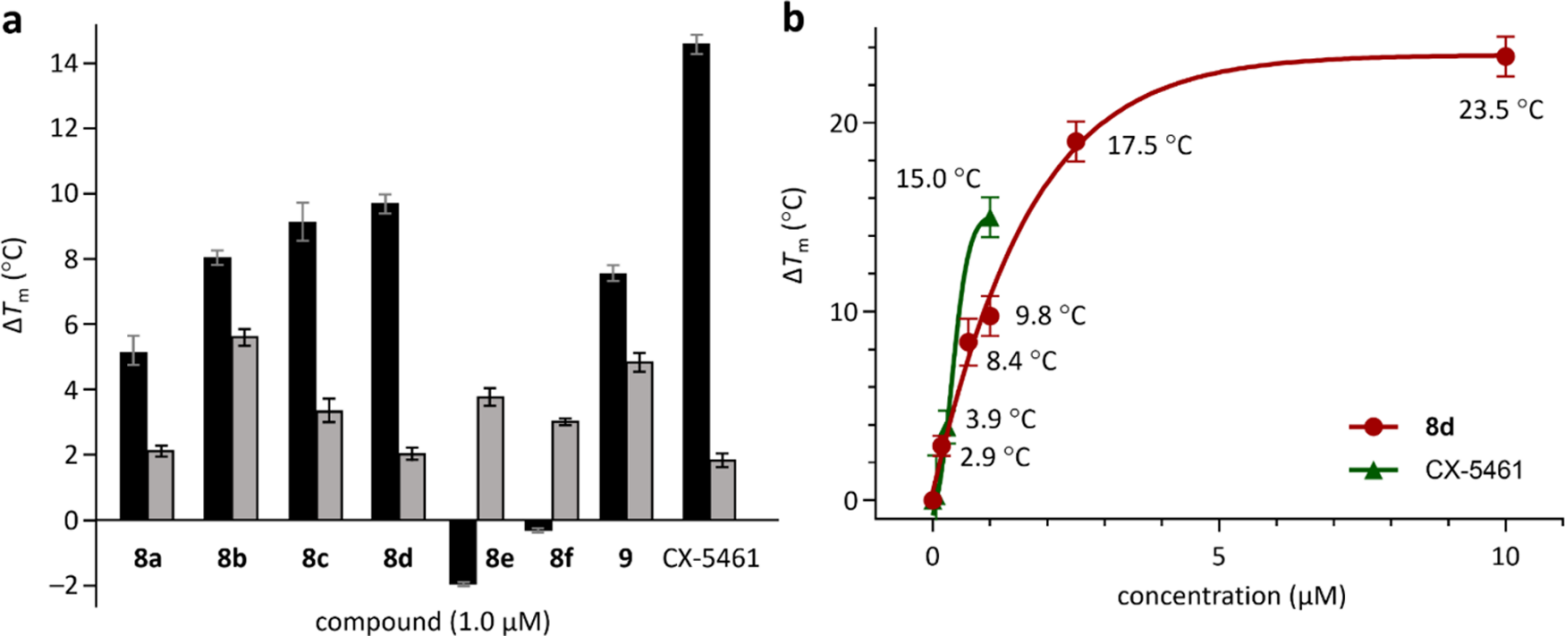
Stabilization of G4 DNA by **8a**–**f**, **9**, and CX-5461. (a) Variations in the melting temperatures (Δ*T*_m_) of telomeric G4 DNA (black) or duplex DNA (gray) treated with 1.0 μM compounds, as determined by the FRET melting assay. (b) Δ*T*_m_ values of G4 DNA treated with **8d** or CX-5461 at various concentrations. For **8d**, the Δ*T*_m_ values were 2.9, 8.4, 9.8, 17.5, and 23.5 °C at concentrations of 0.16, 0.625, 1.0, 2.5, and 10 μM, respectively. For CX-5461, the Δ*T*_m_ values were 3.9 and 15.0 °C at concentrations of 0.25 and 1.0 μM, respectively.

The potency and selectivity of **8a**–**f** and **9** to stabilize G4 DNA were determined using a fluorescence resonance energy transfer (FRET) melting assay with labeled F21T telomeric G4 DNA and T-loop duplex DNA. CX-5461,^42−44^ the leading drug candidate in clinical trials, was used as the positive control. First, G4 and duplex DNA were treated with **8a**–**f** and **9** at the concentration of 1.0 μM, and the variations in the melting temperatures of the DNA (Δ*T*_m_) are shown in Figure 4a. Compounds **8a**–**d** and **9** were found to increase the melting temperature of the G4 DNA (positive Δ*T*_m_). In contrast, **8e** and **8f** decreased the melting temperature (negative Δ*T*_m_). Among them, **8d** bearing two 3-(diethylamino)propylamino groups and an N-5 methyl group (see Scheme 3a) was the most potent and selective. It stabilized G4 DNA with an Δ*T*_m_ value of 9.8 °C, approximately 4.6 times more potent than its ability to stabilize duplex DNA (Δ*T*_m_: 2.1 °C). However, **8d** is about 1.5 times less potent than CX-5461 (Δ*T*_m_: 15.0 °C) at a concentration of 1.0 μM.

The dose-dependent potency of **8d** to stabilize G4 DNA was further evaluated and the results are shown in Figure 4b. Even at a concentration as low as 0.16 μM, **8d** increased the melting temperature of G4 DNA by 2.9 °C. The Δ*T*_m_ values increased with the concentration of **8d**, reaching 8.4 °C at 0.625 μM, 9.8 °C at 1.0 μM, 17.5 °C at 2.5 μM, and 23.5 °C at 10 μM. Although CX-5461 was more potent than **8d** at concentrations below 1.0 μM (Δ*T*_m_: 3.9 °C at 0.25 μM, 15.0 °C at 1.0 μM), its Δ*T*_m_ at concentrations greater than 1.0 μM could not be measured accurately due to forming precipitation in the medium. Thus, **8d** may exhibit greater biocompatibility than CX-5461 at concentrations above 1.0 μM. Moreover, **8d** inhibited the proliferation of MDA-MB-231 cancer cells (triple-negative breast cancer) with a GI_50_ value of 0.41 μM, indicating its potential as an anticancer lead for future optimization.

### Docking Simulations

**Figure 5:**
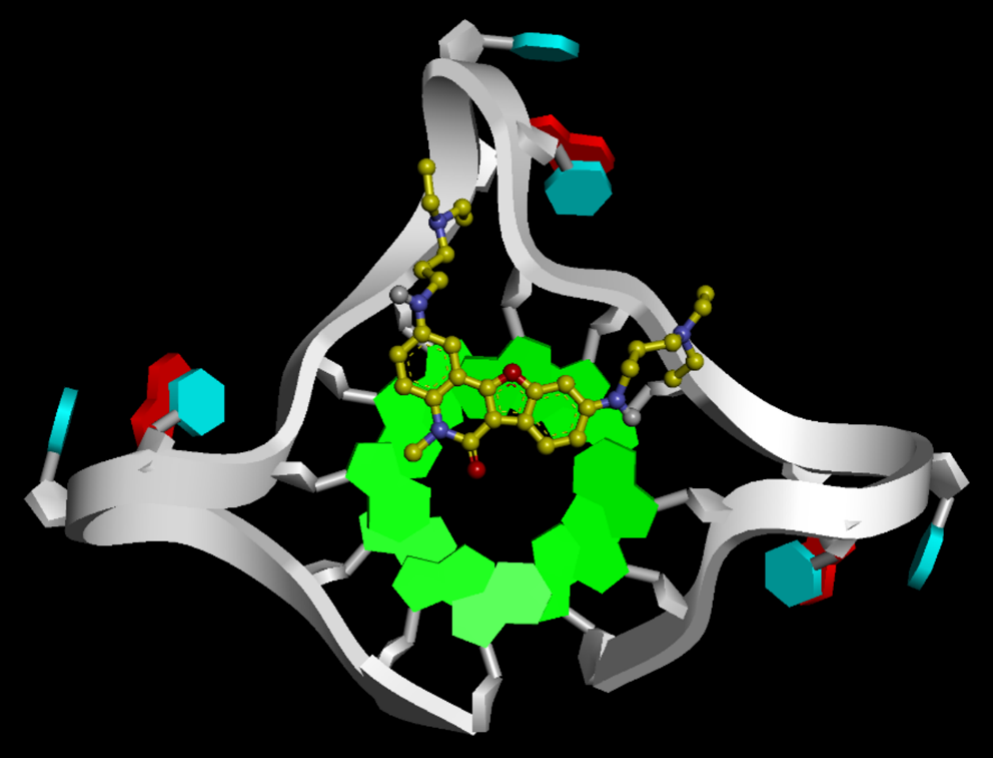
Interaction of **8d** with the propeller telomeric G4 DNA (PDB entry 1KF1, with the first 5′-adenosine deleted). Compound **8d** is shown in a yellow ball-and-stick form. Guanine, adenine, and thymine in the G4 DNA are represented in light green, red, and light blue rings, respectively. The DNA backbone is depicted as a white arrow.

To guide future optimization of the G4 stabilizers using **8d** as a lead, **8a**–**f** and **9** were docked into the crystal structure of the propeller telomeric G4 DNA (PDB entry 1KF1),^45^ where the first 5′-adenosine was deleted to match the DNA sequence of F21T used in the FRET melting assay. The lowest-energy pose of the most potent **8d** in the G4 DNA is depicted in Figure 5. Compound **8d** was found to stack on the G-quartet plane, with its side chain extending to the backbone of the G4 DNA. Intermolecular π–π interactions and electrostatic forces were responsible for the interaction of **8d** with G4 DNA. The mode of interaction shown in Figure 5 could explain the lower potency of **8a** and **8b** in stabilizing G4 DNA, as the dialkylamino termini at their shorter side chains could not reach the appropriate phosphate group in the backbone. For **8e** which did not stabilize G4 DNA (Δ*T*_m_: −2.0 °C), the steric hindrance from the larger morpholino groups at its termini likely impeded its interaction with the backbone of G4 DNA.

Results from Figure 4a have shown that **8b** and **8d** with diethylamino termini were slightly more potent than their dimethylamino analogues **8a** and **8c**. This was due to the stronger hydrophobic interactions between the ethyl groups in **8b** and **8d** with the deoxyribose of the G4 DNA compared to the methyl groups in **8a** and **8c** (Figure 5). Compound **8f**, the desmethyl analogue of **8d**, was inactive to stabilize G4 DNA. The high crystallinity of **8f** caused it to aggregate rather than stack with G-quartet. Finally, the carbonyl group in **8d** also contributed to its π–π interactions with the G-quartet. As a result, benzofuroquinolinone **8d** was more potent than benzofuroquinoline **9** lacking a carbonyl group.

## Conclusions

We developed an optimized copper(I)-catalyzed intramolecular tandem acylation/*O*-arylation of methyl 2-[2-(2-bromophenyl)acetamido]benzoates **4** to synthesize various benzofuro[3,2-*c*]quinolin-6(5*H*)-ones **1** in high yields under mild conditions. We further explored the biological application of **1** as a G4 DNA stabilizer by introducing two side chains with dialkylamino termini. The most potent **8d** significantly increased the melting temperature of G4 DNA and served as a lead for further optimization.

## Experimental Section

### General Information

All reagents and starting materials were used as purchased without further purification. The melting point was determined on an STUART SMP3 apparatus. Proton NMR spectra were recorded on a Varian Mercury-300 (300 MHz) or Agilent 400-MR (400 MHz) spectrometer. Carbon-13 NMR spectra were obtained on a Varian Mercury-300 (75 MHz), Agilent 400-MR (100 MHz), or JEOL ECZ500R/S1 (125 MHz) spectrometer. CDCl_3_ or DMSO-*d*_6_ served as the solvent for NMR measurements. Proton NMR chemical shifts were referenced to the residual CHCl_3_ (δ 7.26 ppm) in CDCl_3_ or the central peak of CHD_2_SOCD_3_ (δ 2.49 ppm) in DMSO-*d*_6_. Carbon-13 NMR chemical shifts were referenced to the central peak of CDCl_3_ (δ 77.0 ppm) or DMSO-*d*_6_ (δ 39.5 ppm). Multiplicities are abbreviated as follows: s, singlet; d, doublet; t, triplet; q, quartet; m, multiplet; and *J*, coupling constant (Hz). Infrared (IR) spectra were recorded on a PerkinElmer One FT-IR spectrometer with an ATR accessory. High-resolution mass spectra were obtained using an LTQ Orbitrap XL mass spectrometer (Thermo Fisher Scientific).

### Safety Statement

As the G-quadruplex stabilizers reported herein exhibit high potency and are cytotoxic or potentially cytotoxic to cells, proper procedures for handling these compounds must be strictly followed, in accordance with organizational guidelines.

### General Procedure for the Synthesis of Benzofuro[3,2-*c*]quinolin-6(5*H*)-ones **1a**–**z** and **1A–E**

Compounds **4a**–**z** through **4A**–**E** (100 mg, 1.0 equiv) were mixed with CuI (0.050 equiv), 1,10-phenanthroline (phen, 0.10 equiv), and K_2_CO_3_ (2.1 equiv) in DMSO (2.0 mL). The reaction mixture was heated to 70 °C in an oil bath for 16 h. The solution was quenched with saturated NH_4_Cl (0.20 mL) and stirred for 10 min. The solution was heated to 90 °C in an oil bath and slowly added with water to form precipitates. The solution was cooled to room temperature and the solids were collected by filtration to give targets **1a**–**z** and **1A**–**E** in 84–99% yield.

#### Benzofuro[3,2-*c*]quinolin-6(5*H*)-one (**1a**)

Yield: 66.2 mg, 98%; white solid; mp 310.5–311.0 °C; ^1^H NMR (400 MHz, DMSO-*d*_6_) δ 12.01 (s, 1 H), 8.11 (dd, *J* = 7.4, 1.0 Hz, 1 H), 8.07 (dd, *J* = 8.0, 1.4 Hz, 1 H), 7.85 (d, *J* = 8.0 Hz, 1 H), 7.62 (td, *J* = 8.0, 1.4 Hz, 1 H), 7.55–7.44 (m, 3 H), 7.35 (td, *J* = 8.0, 1.4 Hz, 1 H); ^13^C{^1^H} NMR (75 MHz, DMSO-*d*_6_) δ 159.6, 158.3, 155.2, 138.8, 131.4, 126.9, 125.1, 124.2, 123.0, 121.72, 121.65, 116.7, 112.3, 111.2, 110.4; IR: 3326, 3165, 3012, 2833, 1669, 1452, 1368, 1189, 1100, 917, 747, 539, 458 cm^–1^; HRMS (ESI) *m*/*z*: [M + H]^+^ Calcd for C_15_H_10_NO_2_ 236.0706; Found 236.0699; CAS *#*57046-70-5.

#### 2-Fluorobenzofuro[3,2-*c*]quinolin-6(5*H*)-one (**1b**)

Yield: 67.8 mg, 98%; white solid; mp 313.0–314.0 °C; ^1^H NMR (400 MHz, DMSO-*d*_6_) δ 12.08 (s, 1 H), 8.10 (ddd, *J* = 7.5, 1.5, 0.7 Hz, 1 H), 7.87–7.80 (m, 2 H), 7.57–7.45 (m, 4 H); ^13^C{^1^H} NMR (100 MHz, DMSO-*d*_6_) δ 159.2, 157.7 (d, *J* = 240.0 Hz), 157.5 (d, *J* = 2.8 Hz), 155.3, 135.6, 127.2, 125.2, 124.1, 121.9, 119.4 (d, *J* = 24.7 Hz), 118.8 (d, *J* = 8.4 Hz), 112.3, 111.7 (d, *J* = 9.3 Hz), 111.3, 106.9 (d, *J* = 24.5 Hz); IR: 3327, 3169, 3063, 2991, 2825, 1661, 1452, 1338, 1262, 1189, 1079, 739, 539 cm^–1^; HRMS (ESI) *m*/*z*: [M + H]^+^ Calcd for C_15_H_9_FNO_2_ 254.0612; Found 254.0608; CAS *#*124028-61-1.

#### 2-Chlorobenzofuro[3,2-*c*]quinolin-6(5*H*)-one (**1c**)

Yield: 67.7 mg, 96%; light yellow solid; mp > 350 °C; ^1^H NMR (400 MHz, DMSO-*d*_6_) δ 12.15 (s, 1 H), 8.11 (d, *J* = 7.5 Hz, 1 H), 8.06 (d, *J* = 1.9 Hz, 1 H), 7.85 (d, *J* = 8.2 Hz, 1 H), 7.66 (dd, *J* = 8.8, 2.2 Hz, 1 H), 7.58–7.46 (m, 3 H); ^13^C{^1^H} NMR (100 MHz, DMSO-*d*_6_) δ 159.3, 157.1, 155.4, 137.5, 131.2, 127.2, 127.0, 126.8, 125.3, 124.0, 121.9, 120.8, 118.6, 112.4, 111.4; IR: 3160, 3003, 2842, 2740, 1682, 1448, 1338, 1282, 1193, 1117, 867, 742, 535 cm^–1^; HRMS (ESI) *m*/*z*: [M + H]^+^ Calcd for C_15_H_9_ClNO_2_ 270.0316; Found 270.0312; CAS *#*124028-62-2.

#### 2-Bromobenzofuro[3,2-*c*]quinolin-6(5*H*)-one (**1d**)

Yield: 72.1 mg, 98%; white solid; mp > 350 °C; ^1^H NMR (400 MHz, DMSO-*d*_6_) δ 12.14 (s, 1 H), 8.19 (d, *J* = 2.2 Hz, 1 H), 8.11 (d, *J* = 7.3 Hz, 1 H), 7.86 (d, *J* = 8.2 Hz, 1 H), 7.77 (dd, *J* = 8.9, 2.2 Hz, 1 H), 7.57–7.43 (m, 3 H); ^13^C{^1^H} NMR (100 MHz, DMSO-*d*_6_) δ 159.3, 157.0, 155.4, 137.8, 133.9, 127.2, 125.3, 124.0, 123.7, 121.9, 118.8, 114.6, 112.9, 112.4, 111.4; IR: 3154, 3001, 2837, 2738, 1941, 1684, 1451, 1336, 1188, 1103, 862, 738, 543 cm^–1^; HRMS (ESI) *m*/*z*: [M + H]^+^ calcd for C_15_H_9_BrNO_2_ 313.9811; Found 313.9808; CAS *#*124028-63-3.

#### 2-Iodobenzofuro[3,2-*c*]quinolin-6(5*H*)-one (**1e**)

Yield: 70.8 mg, 93%; white solid; mp 337.4–338.2 °C; ^1^H NMR (400 MHz, DMSO-*d*_6_) δ 12.10 (s, 1 H), 8.34 (d, *J* = 2.0 Hz, 1 H), 8.10 (d, *J* = 7.7 Hz, 1 H), 7.90 (dd, *J* = 8.7, 2.0 Hz, 1 H), 7.85 (d, *J* = 8.0 Hz, 1 H), 7.59–7.43 (m, 2 H), 7.32 (d, *J* = 8.7 Hz, 1 H); ^13^C{^1^H} NMR (100 MHz, DMSO-*d*_6_) δ 159.3, 156.8, 155.3, 139.4, 138.2, 129.6, 127.2, 125.3, 124.0, 121.8, 118.8, 113.3, 112.4, 111.1, 86.2; IR: 3331, 3152, 3067, 2991, 2838, 1657, 1444, 1329, 1181, 1105, 867, 747, 548 cm^–1^; HRMS (ESI) *m*/*z*: [M + H]^+^ calcd for C_15_H_9_INO_2_ 361.9672; Found 361.9667; new compound.

#### 2-Nitrobenzofuro[3,2-*c*]quinolin-6(5*H*)-one (**1f**)

Yield: 70.6 mg, 99%; yellow solid; mp > 350 °C; ^1^H NMR (400 MHz, DMSO-*d*_6_) δ 12.57 (s, 1 H), 8.80 (d, *J* = 2.5 Hz, 1 H), 8.43 (dd, *J* = 9.0, 2.5 Hz, 1 H), 8.11 (d, *J* = 7.5 Hz, 1 H), 7.92 (d, *J* = 9.0 Hz, 1 H), 7.65 (d, *J* = 7.5 Hz, 1 H), 7.59 (t, *J* = 7.5 Hz, 1 H), 7.52 (t, *J* = 7.5 Hz, 1 H); ^13^C{^1^H} NMR (100 MHz, DMSO-*d*_6_) 159.6, 157.4, 155.5, 142.7, 142.2, 127.6, 125.8, 125.5, 123.7, 121.9, 118.0, 117.7, 112.6, 111.9, 110.9; IR: 3331, 3156, 2990, 2833, 1665, 1533, 1342, 1184, 1112, 891, 759, 705, 530 cm^–1^; HRMS (ESI) *m*/*z*: [M – H]^−^ calcd for C_15_H_7_N_2_O_4_ 279.0411; Found 279.0412; new compound.

#### 2-Methoxybenzofuro[3,2-*c*]quinolin-6(5*H*)-one (**1g**)

Yield: 64.5 mg, 92%; white solid; mp 328.8–329.7 °C; ^1^H NMR (400 MHz, DMSO-*d*_6_) δ 11.88 (brs, 1 H), 8.10 (d, *J* = 7.3 Hz, 1 H), 7.84 (d, *J* = 8.1 Hz, 1 H), 7.55–7.43 (m, 4 H), 7.25 (dd, *J* = 9.0, 2.5 Hz, 1 H), 3.88 (s, 3 H); ^13^C{^1^H} NMR (75 MHz, DMSO-*d*_6_) δ 159.1, 158.0, 155.2, 155.1, 133.5, 126.8, 125.1, 124.4, 121.8, 121.0, 118.3, 112.3, 111.5, 110.7, 102.6, 56.1; IR: 3341, 3158, 3082, 3005, 2831, 2731, 1664, 1443, 1276, 1214, 1184, 806, 753 cm^–1^; HRMS (ESI) *m*/*z*: [M + H]^+^ calcd for C_16_H_12_NO_3_ 266.0812; Found 266.0807; known compound.

#### 3-Fluorobenzofuro[3,2-*c*]quinolin-6(5*H*)-one (**1h**)

Yield: 68.5 mg, 99%; white solid; mp 305.9–306.6 °C; ^1^H NMR (300 MHz, DMSO-*d*_6_) δ 12.08 (s, 1 H), 8.17–8.05 (m, 2 H), 7.89–7.81 (m, 1 H), 7.58–7.41 (m, 2 H), 7.32–7.16 (m, 2 H); ^13^C{^1^H} NMR (75 MHz, DMSO-*d*_6_) δ 163.7 (d, *J* = 247.6 Hz), 159.7, 158.0, 155.2, 140.5 (d, *J* = 12.6 Hz), 126.8, 125.2, 124.4, 124.2 (d, *J* = 11.7 Hz), 121.6, 112.3, 111.3 (d, *J* = 23.8 Hz), 109.7, 108.3, 102.7 (d, *J* = 25.8 Hz); IR: 3101, 3025, 2859, 2821, 1687, 1576, 1452, 1258, 1135, 870, 777, 735, 696 cm^–1^; HRMS (ESI) *m*/*z*: [M + H]^+^ calcd for C_15_H_9_FNO_2_ 254.0612; Found 254.0607; known compound.

#### 3-Chlorobenzofuro[3,2-*c*]quinolin-6(5*H*)-one (**1i**)

Yield: 67.0 mg, 95%; white solid; mp 326.9–327.5 °C; ^1^H NMR (400 MHz, DMSO-*d*_6_) δ 12.09 (s, 1 H), 8.12–8.03 (m, 2 H), 7.85 (d, *J* = 8.4 Hz, 1 H), 7.55–7.45 (m, 3 H), 7.37 (dd, *J* = 8.4, 1.9 Hz, 1 H); ^13^C{^1^H} NMR (75 MHz, DMSO-*d*_6_) δ 159.4, 157.7, 155.3, 139.7, 135.6, 127.0, 125.2, 124.0, 123.5, 123.1, 121.7, 115.9, 112.3, 110.7, 110.1; IR: 3160, 3076, 2999, 2924, 2831, 1682, 1561, 1444, 1370, 1087, 866, 779, 739 cm^–1^; HRMS (ESI) *m*/*z*: [M + H]^+^ calcd for C_15_H_9_ClNO_2_ 270.0316; Found 270.0313; CAS *#*113737-87-4.

#### 3-Bromobenzofuro[3,2-*c*]quinolin-6(5*H*)-one (**1j**)

Yield: 69.1 mg, 94%; off-white solid; mp 325.5–326.0 °C; ^1^H NMR (300 MHz, DMSO-*d*_6_) δ 12.06 (s, 1 H), 8.10 (d, *J* = 7.3 Hz, 1 H), 8.00 (d, *J* = 8.5 Hz, 1 H), 7.85 (d, *J* = 8.0 Hz, 1 H), 7.69 (s, 1 H), 7.57–7.45 (m, 3 H); ^13^C{^1^H} NMR (100 MHz, DMSO-*d*_6_) δ 159.3, 157.7, 155.3, 139.7, 127.0, 125.7, 125.2, 124.2, 124.0, 123.5, 121.7, 118.8, 112.3, 110.8, 110.3; IR: 3152, 3066, 2995, 2825, 1677, 1554, 1448, 1367, 1146, 1087, 862, 739, 552 cm^–1^; HRMS (ESI) *m*/*z*: [M + H]^+^ calcd for C_15_H_9_BrNO_2_ 313.9811; Found 313.9808; new compound.

#### 3-Methylbenzofuro[3,2-*c*]quinolin-6(5*H*)-one (**1k**)

Yield: 62.6 mg, 91%; light yellow solid; mp 320.5–321.0 °C; ^1^H NMR (400 MHz, DMSO-*d*_6_) δ 11.92 (s, 1 H), 8.08 (d, *J* = 7.4 Hz, 1 H), 7.94 (d, *J* = 8.0 Hz, 1 H), 7.82 (d, *J* = 8.0 Hz, 1 H), 7.53–7.42 (m, 2 H), 7.29 (s, 1 H), 7.17 (d, *J* = 8.0 Hz, 1 H), 2.42 (s, 3 H); ^13^C{^1^H} NMR (75 MHz, DMSO-*d*_6_) δ 159.6, 158.5, 155.1, 141.6, 139.1, 126.5, 125.0 (2 × C), 124.4, 121.6, 121.5, 116.4, 112.2, 109.7, 109.0, 22.0; IR: 3149, 3058, 2994, 2918, 2833, 1665, 1449, 1353, 1295, 1106, 892, 781, 631 cm^–1^; HRMS (ESI) *m*/*z*: [M + H]^+^ calcd for C_16_H_12_NO_2_ 250.0863; Found 250.0857; CAS *#*113737-86-3.

#### 3-Nitrobenzofuro[3,2-*c*]quinolin-6(5*H*)-one (**1l**)

Yield: 69.8 mg, 98%; yellow solid; mp > 350 °C; ^1^H NMR (400 MHz, DMSO-*d*_6_) δ 12.44 (s, 1 H), 8.36 (d, *J* = 2.1 Hz, 1 H), 8.32 (d, *J* = 8.7 Hz, 1 H), 8.18–8.11 (m, 2 H), 7.93 (d, *J* = 7.9 Hz, 1 H), 7.62 (td, *J* = 7.9, 1.4 Hz, 1 H), 7.54 (td, *J* = 7.9, 1.0 Hz, 1 H); ^13^C{^1^H} NMR (100 MHz, DMSO-*d*_6_) δ 159.5, 156.8, 156.2, 148.9, 138.9, 128.0, 125.5, 124.1, 123.3, 122.4, 117.0, 115.9, 113.6, 112.5, 112.1; IR: 3347, 3170, 3121, 3074, 3018, 2818, 1671, 1531, 1344, 1107, 907, 841, 754 cm^–1^; HRMS (ESI) *m*/*z*: [M + H]^+^ Calcd for C_15_H_9_N_2_O_4_ 281.0557; Found 281.0552; new compound.

#### 3-Methoxybenzofuro[3,2-*c*]quinolin-6(5*H*)-one (**1m**)

Yield: 61.7 mg, 88%; white solid; mp 292.0–292.8 °C; ^1^H NMR (400 MHz, DMSO-*d*_6_) δ 11.84 (s, 1 H), 8.06–8.00 (m, 1 H), 7.94 (d, *J* = 8.8 Hz, 1 H), 7.78 (dd, *J* = 6.8, 1.3 Hz, 1 H), 7.50–7.35 (m, 2 H), 6.99 (d, *J* = 2.0 Hz, 1 H), 6.94 (dd, *J* = 8.8, 2.0 Hz, 1 H), 3.83 (s, 3 H); ^13^C{^1^H} NMR (100 MHz, DMSO-*d*_6_) δ 161.9, 159.8, 158.8, 154.9, 140.8, 126.1, 124.9, 124.4, 123.1, 121.3, 112.0, 111.9, 108.1, 105.0, 99.5, 55.9; IR: 3138, 3065, 2993, 2826, 1660, 1568, 1447, 1267, 1208, 1137, 907, 784, 696 cm^–1^; HRMS (ESI) *m*/*z*: [M + H]^+^ calcd for C_16_H_12_NO_3_ 266.0812; Found 266.0807; CAS *#*106636-00-4.

#### 2,3-Dimethoxybenzofuro[3,2-*c*]quinolin-6(5*H*)-one (**1n**)

Yield: 61.5 mg, 85%; white solid; mp 338.4–339.2 °C; ^1^H NMR (400 MHz, DMSO-*d*_6_) δ 11.80 (s, 1 H), 8.05 (d, *J* = 7.1 Hz, 1 H), 7.80 (d, *J* = 7.9 Hz, 1 H), 7.49–7.41 (m, 3 H), 7.06 (s, 1 H), 3.90 (s, 3 H), 3.85 (s, 3 H); ^13^C{^1^H} NMR (100 MHz, DMSO-*d*_6_) δ 159.2, 158.6, 154.9, 152.8, 146.0, 134.7, 126.1, 124.9, 124.6, 121.3, 112.0, 108.3, 103.7, 102.2, 99.2, 56.3, 56.1; IR: 3228, 3003, 2889, 2875, 1652, 1456, 1295, 1245, 1108, 781, 751, 514, 457 cm^–1^; HRMS (ESI) *m*/*z*: [M + H]^+^ calcd for C_17_H_14_NO_4_ 296.0917; Found 296.0913; new compound.

#### 9-Fluorobenzofuro[3,2-*c*]quinolin-6(5*H*)-one (**1o**)

Yield: 65.7 mg, 95%; yellow solid; mp 347.9–348.4 °C; ^1^H NMR (400 MHz, DMSO-*d*_6_) δ 12.05 (s, 1 H), 8.07 (dd, *J* = 8.6, 5.7 Hz, 1 H), 8.03 (d, *J* = 7.8 Hz, 1 H), 7.85 (dd, *J* = 7.8, 2.2 Hz, 1 H), 7.62 (t, *J* = 7.8 Hz, 1 H), 7.51 (d, *J* = 8.2 Hz, 1 H), 7.39–7.30 (m, 2 H); ^13^C{^1^H} NMR (100 MHz, DMSO-*d*_6_) δ 161.3 (d, *J* = 242.8 Hz), 159.3, 159.0, 155.4 (d, *J* = 14.0 Hz), 138.7, 131.4, 123.0, 122.4 (d, *J* = 10.3 Hz), 121.5, 120.9, 116.7, 113.2 (d, *J* = 23.8 Hz), 111.1, 110.2, 100.7 (d, *J* = 27.6 Hz); IR: 3156, 3079, 3003, 2863, 1673, 1605, 1495, 1372, 1100, 840, 747, 607, 525 cm^–1^; HRMS (ESI) *m*/*z*: [M – H]^−^ calcd for C_15_H_7_FNO_2_ 252.0466; Found 252.0465; new compound.

#### 9-Chlorobenzofuro[3,2-*c*]quinolin-6(5*H*)-one (**1p**)

Yield: 66.3 mg, 94%; white solid; mp 327.8–328.5 °C; ^1^H NMR (300 MHz, DMSO-*d*_6_) δ 12.05 (s, 1 H), 8.09–7.99 (m, 3 H), 7.63 (t, *J* = 7.6 Hz, 1 H), 7.56–7.45 (m, 2 H), 7.35 (t, *J* = 7.6 Hz, 1 H); ^13^C{^1^H} NMR (75 MHz, DMSO-*d*_6_) δ 159.2, 159.0, 155.4, 139.0, 131.6, 131.1, 125.6, 123.4, 123.0, 122.5, 121.6, 116.7, 112.9, 111.0, 110.1; IR: 3328, 3164, 3003, 2850, 1658, 1433, 1367, 1093, 1055, 849, 825, 758, 596 cm^–1^; HRMS (ESI) *m*/*z*: [M + H]^+^ calcd for C_15_H_9_ClNO_2_ 270.0316; Found 270.0316; new compound.

#### 9-Bromobenzofuro[3,2-*c*]quinolin-6(5*H*)-one (**1q**)

Yield: 68.4 mg, 93%; white solid; mp > 350 °C; ^1^H NMR (300 MHz, DMSO-*d*_6_) δ 12.06 (s, 1 H), 8.16 (d, *J* = 2.1 Hz, 1 H), 8.07–8.02 (m, 1 H), 7.84 (d, *J* = 8.5 Hz, 1 H), 7.68–7.61 (m, 2 H), 7.51 (d, *J* = 8.5 Hz, 1 H), 7.38–7.32 (m, 1 H); ^13^C{^1^H} NMR (75 MHz, DMSO-*d*_6_) δ 159.3 (2 × C), 154.2, 139.3, 131.8, 129.3, 126.5, 123.8, 123.1, 121.8, 117.3, 116.9, 114.5, 110.9, 109.7; IR: 3334, 3169, 3069, 3003, 2808, 1660, 1561, 1441, 1336, 1184, 1091, 885, 749 cm^–1^; HRMS (ESI) *m*/*z*: [M + H]^+^ calcd for C_15_H_9_BrNO_2_ 313.9811; Found 313.9807; new compound.

#### 9-Nitrobenzofuro[3,2-*c*]quinolin-6(5*H*)-one (**1r**)

Yield: 70.6 mg, 99%; yellow solid; mp > 350 °C; ^1^H NMR (400 MHz, DMSO-*d*_6_) δ 12.19 (s, 1 H), 8.78 (d, *J* = 2.0 Hz, 1 H), 8.38 (dd, *J* = 8.6, 2.0 Hz, 1 H), 8.27 (d, *J* = 8.6 Hz, 1 H), 8.11 (d, *J* = 7.5 Hz, 1 H), 7.74–7.68 (m, 1 H), 7.54 (d, *J* = 8.3 Hz, 1 H), 7.44–7.37 (m, 1 H); ^13^C{^1^H} NMR (100 MHz, DMSO-*d*_6_) δ 162.0, 159.0, 154.1, 145.8, 139.7, 132.7, 130.5, 123.3, 122.2, 121.8, 120.9, 116.9, 110.6, 110.0, 108.7; IR: 3330, 3167, 3111, 3001, 2830, 1666, 1525, 1338, 1187, 814, 758, 722, 590 cm^–1^; HRMS (ESI) *m*/*z*: [M – H]^−^ calcd for C_15_H_7_N_2_O_4_ 279.0411; Found 279.0413; new compound.

#### 8-Fluorobenzofuro[3,2-*c*]quinolin-6(5*H*)-one (**1s**)

Yield: 65.0 mg, 94%; white solid; mp 345.9–346.7 °C; ^1^H NMR (400 MHz, DMSO-*d*_6_) δ 12.05 (s, 1 H), 8.04 (d, *J* = 7.8 Hz, 1 H), 7.89 (dd, *J* = 7.9, 4.0 Hz, 1 H), 7.75 (dd, *J* = 7.8, 2.3 Hz, 1 H), 7.63 (t, *J* = 7.8 Hz, 1 H), 7.51 (d, *J* = 8.2 Hz, 1 H), 7.42–7.27 (m, 2 H); ^13^C{^1^H} NMR (100 MHz, DMSO-*d*_6_) δ 159.8 (d, *J* = 238.8 Hz), 159.5 (d, *J* = 54.6 Hz), 151.5, 139.1, 131.7, 125.5, 123.0, 121.7, 116.7, 114.2 (d, *J* = 26.2 Hz), 113.7 (d, *J* = 9.8 Hz), 111.0, 110.5, 107.4, 107.1; IR: 3332, 3100, 3006, 2820, 1665, 1442, 1255, 1140, 844, 733, 680, 540, cm^–1^; HRMS (ESI) *m*/*z*: [M + H]^+^ calcd for C_15_H_9_FNO_2_ 254.0612; Found 254.0610; known compound.

#### 8-Chlorobenzofuro[3,2-*c*]quinolin-6(5*H*)-one (**1t**)

Yield: 65.5 mg, 93%; white solid; mp > 350 °C; ^1^H NMR (400 MHz, DMSO-*d*_6_) δ 12.08 (s, 1 H), 8.04 (dd, *J* = 7.7 Hz, 1 H), 8.00 (d, *J* = 2.1 Hz, 1 H), 7.89 (d, *J* = 8.8 Hz, 1 H), 7.63 (t, *J* = 7.7 Hz, 1 H), 7.56–7.49 (m, 2 H), 7.35 (t, *J* = 7.7 Hz, 1 H); ^13^C{^1^H} NMR (100 MHz, DMSO-*d*_6_) δ 159.5, 159.2, 153.8, 139.1, 131.8, 129.4, 126.6, 125.9, 123.1, 121.8, 120.7, 116.8, 114.0, 110.9, 109.8; IR: 3338, 3168, 3006, 2812, 1667, 1445, 1090, 886, 801, 745, 660, 553, 451 cm^–1^; HRMS (ESI) *m*/*z*: [M + H]^+^ Calcd for C_15_H_9_ClNO_2_ 270.0316; Found 270.0316; known compound.

#### 8,9-Dimethoxybenzofuro[3,2-*c*]quinolin-6(5*H*)-one (**1u**)

Yield: 60.8 mg, 84%; light brown solid; mp 311.5–312.5 °C; ^1^H NMR (400 MHz, DMSO-*d*_6_) δ 11.97 (s, 1 H), 7.97 (d, *J* = 7.7 Hz, 1 H), 7.59–7.47 (m, 4 H), 7.32 (t, *J* = 7.7 Hz, 1 H), 3.87 (s, 3 H), 3.86 (s, 3 H); ^13^C{^1^H} NMR (100 MHz, DMSO-*d*_6_) δ 159.6, 157.2, 150.1, 149.4, 147.7, 138.0, 130.4, 122.8, 120.9, 116.6, 115.8, 111.5, 111.0, 102.6, 96.9, 56.5, 56.4; IR: 3330, 3061, 2997, 2839, 1666, 1483, 1358, 1299, 1210, 1120, 1047, 800, 744 cm^–1^; HRMS (ESI) *m*/*z*: [M + H]^+^ calcd for C_17_H_14_NO_4_ 296.0917; Found 296.0917; new compound.

#### [1,3]Dioxolo[4′,5′:5,6]benzofuro[3,2-*c*]quinolin-6(5*H*)-one (**1v**)

Yield: 62.7 mg, 88%; yellow solid; mp 344.8–345.5 °C; ^1^H NMR (400 MHz, DMSO-*d*_6_) δ 11.93 (s, 1 H), 7.96 (d, *J* = 7.6 Hz, 1 H), 7.59–7.46 (m, 3 H), 7.42 (s, 1 H), 7.31 (t, *J* = 7.6 Hz, 1 H), 6.14 (s, 2 H); ^13^C{^1^H} NMR (100 MHz, DMSO-*d*_6_) δ 159.4, 157.6, 150.6, 147.6, 145.8, 138.0, 130.5, 122.9, 121.0, 117.4, 116.6, 111.4, 111.1, 102.4, 99.7, 94.9; IR: 3274, 3091, 3015, 2904, 1666, 1457, 1295, 1107, 1043, 950, 852, 745, 664 cm^–1^; HRMS (ESI) *m*/*z*: [M – H]^−^ Calcd for C_16_H_8_NO_4_ 278.0459; Found 278.0455; new compound.

#### 3,9-Difluorobenzofuro[3,2-*c*]quinolin-6(5*H*)-one (**1w**)

Yield: 67.1 mg, 95%; white solid; mp > 350 °C; ^1^H NMR (400 MHz, DMSO-*d*_6_) δ 12.13 (s, 1 H), 8.10 (dd, *J* = 8.4, 6.1 Hz, 1 H), 7.87 (dd, *J* = 9.0, 4.0 Hz, 1 H), 7.72 (dd, *J* = 8.3, 2.7 Hz, 1 H), 7.35 (td, *J* = 9.3, 2.7 Hz, 1 H), 7.26–7.19 (m, 2 H); ^13^C{^1^H} NMR (125 MHz, DMSO-*d*_6_) δ 164.2 (d, *J* = 248.5 Hz), 160.3 (d, *J* = 201.5 Hz), 159.5, 159.4 (d, *J* = 68.8 Hz), 151.7, 141.0 (d, *J* = 12.6 Hz), 125.6 (d, *J* = 11.7 Hz), 124.4 (d, *J* = 10.7 Hz), 114.1 (d, *J* = 26.3 Hz), 113.6 (d, *J* = 9.8 Hz), 111.3 (d, *J* = 24.1 Hz), 110.0, 108.4, 107.3 (d, *J* = 26.2 Hz), 103.0 (d, *J* = 26.4 Hz); IR: 3176, 3078, 2998, 2890, 2849, 1688, 1603, 1453, 1253, 1245, 1137, 801, 690 cm^–1^; HRMS (ESI) *m*/*z*: [M + H]^+^ calcd for C_15_H_8_F_2_NO_2_ 272.0518; Found 272.0511; new compound.

#### 3,8-Difluorobenzofuro[3,2-*c*]quinolin-6(5*H*)-one (**1x**)

Yield: 67.8 mg, 96%; white solid; mp > 350 °C; ^1^H NMR (300 MHz, DMSO-*d*_6_) δ 12.14 (s, 1 H), 8.14–8.02 (m, 2 H), 7.86 (dd, *J* = 9.1, 2.3 Hz, 1 H), 7.40–7.33 (m, 1 H), 7.30–7.19 (m, 2 H); ^13^C{^1^H} NMR (125 MHz, DMSO-*d*_6_) δ 163.9 (d, *J* = 248.1 Hz), 161.6 (d, *J* = 243.1 Hz), 159.5, 158.9, 155.6 (d, *J* = 13.9 Hz), 140.6 (d, *J* = 13.1 Hz), 124.1 (d, *J* = 10.6 Hz), 122.5 (d, *J* = 10.2 Hz), 121.1, 113.2 (d, *J* = 23.8 Hz), 111.3 (d, *J* = 23.9 Hz), 109.7, 108.5, 103.0 (d, *J* = 26.3 Hz), 100.5 (d, *J* = 27.8 Hz); IR: 3151, 3086, 2968, 2869, 2813, 1679, 1491, 1376, 1257, 1104, 838, 796, 655 cm^–1^; HRMS (ESI) *m*/*z*: [M + H]^+^ calcd for C_15_H_8_F_2_NO_2_ 272.0518; Found 272.0513; new compound.

#### 8-Chloro-3-fluorobenzofuro[3,2-*c*]quinolin-6(5*H*)-one (**1y**)

Yield: 68.2 mg, 95%; white solid; mp > 350 °C; ^1^H NMR (400 MHz, DMSO-*d*_6_) δ 12.10 (s, 1 H), 8.10–8.00 (m, 1 H), 7.92 (s, 1 H), 7.83 (d, *J* = 8.9 Hz, 1 H), 7.49 (d, *J* = 8.9 Hz, 1 H), 7.28–7.11 (m, 2 H); ^13^C{^1^H} NMR (100 MHz, DMSO-*d*_6_) δ 163.9 (d, *J* = 248.1 Hz), 159.3, 159.1, 153.6, 140.7 (d, *J* = 12.5 Hz), 129.5, 126.6, 125.7, 124.5 (d, *J* = 10.7 Hz), 120.6, 114.0, 111.5 (d, *J* = 24.0 Hz), 109.1, 108.0, 102.8 (d, *J* = 26.1 Hz); IR: 3163, 3086, 3018, 2916, 2877, 1679, 1449, 1278, 1137, 847, 796, 742, 529 cm^–1^; HRMS (ESI) *m*/*z*: [M + H]^+^ calcd for C_15_H_8_ClFNO_2_ 288.0222; Found 288.0221; new compound.

#### 3,8-Dichlorobenzofuro[3,2-*c*]quinolin-6(5*H*)-one (**1z**)

Yield: 68.5 mg, 94%; white solid; mp > 350 °C; ^1^H NMR (400 MHz, DMSO-*d*_6_) δ 12.15 (s, 1 H), 8.03 (d, *J* = 8.5 Hz, 1 H), 7.97 (d, *J* = 2.3 Hz, 1 H), 7.88 (d, *J* = 8.8 Hz, 1 H), 7.55 (dd, *J* = 8.8, 2.3 Hz, 1 H), 7.50 (d, *J* = 2.0 Hz, 1 H), 7.38 (dd, *J* = 8.5, 2.0 Hz, 1 H); ^13^C{^1^H} NMR (125 MHz, DMSO-*d*_6_) δ 159.2, 159.0, 154.0, 140.1, 136.3, 129.8, 126.9, 125.9, 123.7, 123.3, 120.9, 116.2, 114.1, 110.2, 110.0; IR: 3138, 3078, 2984, 2886, 2852, 1688, 1573, 1453, 1252, 1133, 860, 801, 690 cm^–1^; HRMS (ESI) *m*/*z*: [M + H]^+^ calcd for C_15_H_8_Cl_2_NO_2_ 303.9927; Found 303.9924; new compound.

#### 3-Chloro-8,9-dimethoxybenzofuro[3,2-*c*]quinolin-6(5*H*)-one (**1A**)

Yield: 63.3 mg, 85%; gray solid; mp 308.5–309.5 °C; ^1^H NMR (400 MHz, DMSO-*d*_6_) δ 12.03 (s, 1 H), 7.94 (d, *J* = 8.5 Hz, 1 H), 7.50 (s, 1 H), 7.47 (d, *J* = 1.7 Hz, 1 H), 7.45 (s, 1 H), 7.33 (dd, *J* = 8.5, 1.7 Hz, 1 H), 3.86 (s, 3 H), 3.85 (s, 3 H); ^13^C{^1^H} NMR (75 MHz, DMSO-*d*_6_) δ 159.5, 156.5, 150.2, 149.6, 147.8, 138.7, 134.6, 122.9, 122.7, 115.8, 115.6, 111.2, 110.4, 102.5, 96.8, 56.5, 56.3; IR: 3142, 3091, 2997, 2911, 2826, 1674, 1483, 1295, 1124, 1043, 801, 762, 617 cm^–1^; HRMS (ESI) *m*/*z*: [M + H]^+^ Calcd for C_17_H_13_ClNO_4_ 330.0528; Found 330.0522; new compound.

#### 3-Chloro[1,3]dioxolo[4′,5′:5,6]benzofuro[3,2-*c*]quinolin-6(5*H*)-one (**1B**)

Yield: 65.4 mg, 89%; white solid; mp 349.5–350.0 °C; ^1^H NMR (400 MHz, DMSO-*d*_6_) δ 11.98 (s, 1 H), 7.94 (d, *J* = 8.6 Hz, 1 H), 7.51 (s, 1 H), 7.47 (d, *J* = 1.9 Hz, 1 H), 7.38 (s, 1 H), 7.33 (dd, *J* = 8.6, 1.9 Hz, 1 H), 6.14 (s, 2 H); ^13^C{^1^H} NMR (100 MHz, DMSO-*d*_6_) δ 159.4, 157.0, 150.8, 147.8, 146.0, 138.8, 134.8, 123.0, 122.9, 117.2, 115.9, 111.3, 110.3, 102.5, 99.7, 94.9; IR: 3146, 3082, 2991, 2849, 2825, 1679, 1462, 1366, 1302, 1097, 944, 846, 681 cm^–1^; HRMS (ESI) *m*/*z*: [M + H]^+^ calcd for C_16_H_9_ClNO_4_ 314.0215; Found 314.0210; new compound.

#### 2,9-Dibromobenzofuro[3,2-*c*]quinolin-6(5*H*)-one (**1C**)

Yield: 73.8 mg, 95%; white solid; mp > 350 °C; ^1^H NMR (400 MHz, DMSO-*d*_6_) δ 12.19 (s, 1 H), 8.22–8.06 (m, 2 H), 7.82 (d, *J* = 8.6 Hz, 1 H), 7.77 (d, *J* = 8.6 Hz, 1 H), 7.67 (d, *J* = 7.9 Hz, 1 H), 7.43 (d, *J* = 8.6 Hz, 1 H); ^13^C{^1^H} NMR (125 MHz, DMSO-*d*_6_) δ 158.4, 157.4, 153.7, 137.5, 133.8, 129.2, 125.6, 123.3 (2 × C), 118.4, 116.9, 114.2, 114.0, 112.0, 110.0; IR: 3159, 2998, 2878, 2827, 1679, 1440, 1253, 1184, 869, 801, 724, 677 cm^–1^; HRMS (ESI) *m*/*z*: [M – H]^−^ calcd for C_15_H_6_Br_2_NO_2_ 389.8771; Found 389.8774; new compound.

#### 8-Chloro-2-methoxybenzofuro[3,2-*c*]quinolin-6(5*H*)-one (**1D**)

Yield: 66.1 mg, 91%; white solid; mp 314.0–315.0 °C; ^1^H NMR (400 MHz, DMSO-*d*_6_) δ 11.95 (s, 1 H), 7.99 (d, *J* = 1.6 Hz, 1 H), 7.85 (d, *J* = 8.8 Hz, 1 H), 7.52 (dd, *J* = 8.8, 1.6 Hz, 1 H), 7.46–7.40 (m, 2 H), 7.26 (dd, *J* = 8.8, 2.3 Hz, 1 H), 3.86 (s, 3 H); ^13^C{^1^H} NMR (100 MHz, DMSO-*d*_6_) δ 159.1, 158.7, 155.1, 153.7, 133.7, 129.4, 126.6, 126.0, 121.5, 120.8, 118.4, 113.9, 111.2, 110.0, 102.6, 56.1; IR: 3163, 3065, 3005, 2899, 2835, 1657, 1516, 1435, 1218, 1060, 873, 779, 672 cm^–1^; HRMS (ESI) *m*/*z*: [M + H]^+^ calcd for C_16_H_11_ClNO_3_ 300.0422; Found 300.0412; new compound.

#### 2,3-Dimethoxy[1,3]dioxolo[4′,5′:5,6]benzofuro[3,2-*c*]quinolin-6(5*H*)-one (**1E**)

Yield: 63.0 mg, 84%; white solid; mp > 350 °C; ^1^H NMR (400 MHz, DMSO-*d*_6_) δ 11.76 (s, 1 H), 7.51 (s, 1 H), 7.40 (s, 1 H), 7.36 (s, 1 H), 7.04 (s, 1 H), 6.13 (s, 2 H), 3.88 (s, 3 H), 3.84 (s, 3 H); ^13^C{^1^H} NMR (125 MHz, DMSO-*d*_6_) δ 159.3, 158.3, 153.0, 150.6, 147.2, 146.6, 145.9, 134.2, 118.3, 109.4, 104.7, 103.6, 102.3, 100.7, 100.0, 94.6, 57.1, 56.7; IR: 3334, 3138, 2964, 2840, 1666, 1453, 1334, 1210, 1099, 996, 818, 579, 459 cm^–1^; HRMS (ESI) *m*/*z*: [M – H]^−^ calcd for C_18_H_12_NO_6_ 338.0670; Found 338.0673; new compound.

### Large-Scale Synthesis of **1C**

To a 100 mL round-bottom flask equipped with a stir bar were added methyl 5-bromo-2-[2-(2,4-dibromophenyl)acetamido]benzoate (2.011 g, 3.974 mmol, 1.0 equiv), CuI (39.2 mg, 0.206 mmol, 0.052 equiv), phen (72.0 mg, 0.400 mmol, 0.10 equiv), K_2_CO_3_ (1.155 g, 8.357 mmol, 2.1 equiv), and DMSO (20 mL). The reaction mixture was heated at 70 °C in an oil bath under a nitrogen atmosphere for 16 h. The reaction mixture was cooled to room temperature, saturated NH_4_Cl (2.0 mL) was added, and the mixture was stirred for 10 min. The solution was transferred to a one-liter beaker, slowly diluted with water (400 mL), and heated at 90 °C in an oil bath for 30 min. The solution was then cooled to room temperature and stirred for an additional 60 min. The resulting solids were filtered, washed with water (200 mL), and air-dried for 30 min to give the target **1C** (1.471 g, 3.743 mmol) in 94% yield.

### Synthesis of 3-(2-Bromophenyl)-4-hydroxyquinolin-2(1*H*)-one (**7a**)

Compounds **4a** (0.6073 g, 1.744 mmol, 1.0 equiv) and K_2_CO_3_ (0.5062 mg, 3.663 mmol, 2.1 equiv) in DMSO (1.0 mL) was heated at 70 °C in an oil bath for 8.0 h. The solution was quenched with 6 N HCl (1.2 mL, 1.0 equiv), stirred for 10 min, and slowly added to water to form precipitates. The solids were collected by filtration and air-dried to give **7a** (0.5238 g, 1.657 mmol) in 95% yield: white solids; mp 342.1–343.1 °C; ^1^H NMR (400 MHz, DMSO-*d*_6_) δ 11.45 (s, 1 H), 10.26 (s, 1 H), 7.92 (d, *J* = 8.0 Hz, 1 H), 7.67 (d, *J* = 8.0 Hz, 1 H), 7.52 (t, *J* = 7.6 Hz, 1 H), 7.40 (t, *J* = 7.7 Hz, 1 H), 7.33–7.24 (m, 3 H), 7.17 (t, *J* = 8.0 Hz, 1 H); ^13^C{^1^H} NMR (100 MHz, DMSO-*d*_6_) δ 162.5, 158.2, 138.9, 135.3, 133.9, 132.6, 131.3, 129.8, 127.9, 126.2, 123.7, 121.6, 115.5 (2 × C), 112.9; IR: 3284, 3150, 3110, 3003, 1638, 1599, 1402, 1295, 1140, 856, 752, 565 cm^–1^; HRMS (ESI) *m*/*z*: [M + H]^+^ Calcd for C_15_H_11_BrNO_2_ 315.9968; Found 315.9965; new compound.

### Synthesis of 2,9-Dibromo-5-methylbenzofuro[3,2-*c*]quinolin-6(5*H*)-one (**10**)

A solution of **1C** (0.219 g, 0.557 mmol, 1.0 equiv) and NaH (60% in mineral oil, 44.5 mg, 1.11 mmol, 2.0 equiv) in DMF (3.0 mL) was stirred at room temperature for 10 min. The reaction mixture was added with MeI (94.8 mg, 0.668 mmol, 1.2 equiv) and heated at 45 °C in an oil bath for an additional 16 h. The solution was added with H_2_O, and the resulting precipitates were filtered, washed with water, and dried under vacuum to give **10** (0.186 mg, 0.457 mmol) as a light yellow solid in 82% yield: mp 246.9–247.5 °C; ^1^H NMR (300 MHz, CDCl_3_) δ 8.39 (d, *J* = 2.0 Hz, 1 H), 8.23 (d, *J* = 2.3 Hz, 1 H), 7.71 (dd, *J* = 9.0, 2.3 Hz, 1 H), 7.57 (dd, *J* = 8.7, 2.0 Hz, 1 H), 7.50 (d, *J* = 8.7 Hz, 1 H), 7.36 (d, *J* = 9.0 Hz, 1 H), 3.80 (s, 3 H); ^13^C{^1^H} NMR (75 MHz, CDCl_3_) δ 158.8, 156.6, 154.2, 138.3, 133.8, 129.5, 126.1, 125.0, 124.7, 117.9, 116.9, 115.6, 113.8, 112.9, 110.3, 29.4; IR: 3001, 2993, 1665, 1479, 1439, 1217, 1208, 1128, 877, 822 cm^–1^; HRMS (ESI) *m*/*z*: [M + H]^+^ calcd for C_16_H_10_Br_2_NO_2_ 405.9073; Found 405.9067; new compound.

### General Procedure for the Synthesis of Benzofuro[3,2-*c*]quinolin-6(5*H*)-ones **8a–f** Bearing Different Side Chains at the C-2 and C-9 Positions

A solution of **1C** or **10** (200 mg, 1.0 equiv), Pd(OAc)_2_ (0.20 equiv), CyJohnPhos (0.40 equiv), *t*-BuONa (8.0 equiv), and the corresponding *N*,*N*-dialkylated alkanediamine (8.0 equiv) in DME/*t*-BuOH (8.0:8.0 mL) was degassed and heated under reflux in an oil bath for 39 h under N_2_. The reaction mixture was added to CH_2_Cl_2_ (10 mL), filtered through Celite, and concentrated under reduced pressure. The residue was purified by HPLC isocratically with MeOH (50%) and 0.1% formic acid in H_2_O (50%) for 5.0 min and MeOH (100%) for 6.0 min. The fractions of the target product were combined, concentrated, adjusted to pH 10 by 1.0 N NaOH, and extracted with CH_2_Cl_2_. The organic layer was dried over anhydrous MgSO_4_ and concentrated under reduced pressure to give **8a**–**f** as an oil in 70–88% yields.

#### 2,9-Bis{[2-(dimethylamino)ethyl]amino}-5-methylbenzofuro[3,2-*c*]quinolin-6(5*H*)-one (**8a**)

Yield: 155.3 mg, 75%; orange oil; ^1^H NMR (300 MHz, CDCl_3_) δ 7.48 (d, *J* = 2.4 Hz, 1 H), 7.41 (d, *J* = 8.7 Hz, 1 H), 7.33 (d, *J* = 9.1 Hz, 1 H), 7.25 (d, *J* = 2.8 Hz, 1 H), 6.99 (dd, *J* = 9.1, 2.8 Hz, 1 H), 6.78 (dd, *J* = 8.8, 2.4 Hz, 1 H), 3.80 (s, 3 H), 3.28 (t, *J* = 5.8 Hz, 4 H), 2.67–2.61 (m, 4 H), 2.30 (s, 6 H), 2.29 (s, 6 H); ^13^C{^1^H} NMR (75 MHz, CDCl_3_) δ 159.3, 157.3, 149.0, 146.1, 144.0, 131.9, 125.8, 118.4, 116.1, 113.9, 113.5, 111.4, 110.4, 103.5, 102.2, 58.0, 57.8, 45.2 (2 × C), 42.0, 41.5, 29.0; IR: 3410, 3017, 2970, 2845, 1674, 1504, 1485, 1434, 1271, 1200, 1135, 855, 532 cm^–1^; HRMS (ESI) *m*/*z*: [M + H]^+^ calcd for C_24_H_32_N_5_O_2_ 422.2551; Found 422.2548; new compound.

#### 2,9-Bis{[2-(diethylamino)ethyl]amino}-5-methylbenzofuro[3,2-*c*]quinolin-6(5*H*)-one (**8b**)

Yield: 178.4 mg, 76%; orange oil; ^1^H NMR (300 MHz, CDCl_3_) δ 7.49 (d, *J* = 2.6 Hz, 1 H), 7.41 (d, *J* = 8.8 Hz, 1 H), 7.32 (d, *J* = 9.2 Hz, 1 H), 7.24 (d, *J* = 2.6 Hz, 1 H), 6.98 (dd, *J* = 9.1, 2.6 Hz, 1 H), 6.76 (dd, *J* = 8.8, 2.6 Hz, 1 H), 4.55 (brs, 2 H), 3.80 (s, 3 H), 3.22 (t, *J* = 6.0 Hz, 4 H), 2.77–2.72 (m, 4 H), 2.63–2.53 (m, 8 H), 1.07–1.01 (m, 12 H); ^13^C{^1^H} NMR (75 MHz, CDCl_3_) δ 159.3, 157.3, 149.0, 146.3, 144.1, 131.9, 125.8, 118.4, 116.1, 114.0, 113.4, 111.3, 110.4, 103.9, 102.4, 51.6, 51.4, 46.7 (2 × C), 42.1, 41.7, 29.0, 11.82, 11.78; IR: 3091, 2944, 2826, 1688, 1496, 1433, 1255, 1209, 1132, 861, 501 cm^–1^; HRMS (ESI) *m*/*z*: [M + H]^+^ calcd for C_28_H_40_N_5_O_2_ 478.3177; Found 478.3168; new compound.

#### 2,9-Bis{[3-(dimethylamino)propyl]amino}-5-methylbenzofuro[3,2-*c*]quinolin-6(5*H*)-one (**8c**)

Yield: 154.6 mg, 70%; orange oil; ^1^H NMR (300 MHz, CDCl_3_) δ 7.46 (d, *J* = 2.6 Hz, 1 H), 7.40 (d, *J* = 8.8 Hz, 1 H), 7.28 (d, *J* = 9.1 Hz, 1 H), 7.20 (d, *J* = 2.6 Hz, 1 H), 6.91 (dd, *J* = 9.1, 2.6 Hz, 1 H), 6.71 (dd, *J* = 8.8, 2.6 Hz, 1 H), 3.78 (s, 3 H), 3.31–3.25 (m, 4 H), 2.47–2.40 (m, 4 H), 2.27 (s, 6 H), 2.25 (s, 6 H), 1.92–1.75 (m, 4 H); ^13^C{^1^H} NMR (75 MHz, CDCl_3_) δ 159.3, 157.3, 148.9, 146.1, 144.1, 131.8, 125.8, 118.2, 116.1, 114.0, 113.0, 111.3, 110.4, 103.7, 102.1, 58.30, 58.27, 45.6 (2 × C), 44.0, 43.6, 29.0, 27.0, 26.8; IR: 3394, 3074, 2991, 2857, 1670, 1488, 1436, 1270, 1209, 1144, 860, 751 cm^–1^; HRMS (ESI) *m*/*z*: [M + H]^+^ calcd for C_26_H_36_N_5_O_2_ 450.2864; Found 450.2855; new compound.

#### 2,9-Bis{[3-(diethylamino)propyl]amino}-5-methylbenzofuro[3,2-*c*]quinolin-6(5*H*)-one (**8d**)

Yield: 218.7 mg, 88%; orange oil; ^1^H NMR (300 MHz, CDCl_3_) δ 7.45 (d, *J* = 2.4 Hz, 1 H), 7.40 (d, *J* = 8.7 Hz, 1 H), 7.29 (d, *J* = 9.1 Hz, 1 H), 7.18 (d, *J* = 2.7 Hz, 1 H), 6.90 (dd, *J* = 9.1, 2.7 Hz, 1 H), 6.70 (dd, *J* = 8.7, 2.4 Hz, 1 H), 3.78 (s, 3 H), 3.30–3.25 (m, 4 H), 2.60–2.51 (m, 12 H), 1.88–1.78 (m, 4 H), 1.07–1.03 (m, 12 H); ^13^C{^1^H} NMR (75 MHz, CDCl_3_) δ 159.3, 157.3, 148.9, 146.3, 144.3, 131.7, 125.8, 118.2, 116.1, 114.0, 113.1, 111.3, 110.4, 103.4, 101.8, 52.2, 52.0, 46.94, 46.91, 44.5, 44.3, 29.0, 26.2, 25.9, 11.82, 11.76; IR: 3080, 2990, 2805, 1640, 1470, 1439, 1277, 1201, 1140, 865, 495 cm^–1^; HRMS (ESI) *m*/*z*: [M + H]^+^ calcd for C_30_H_44_N_5_O_2_ 506.3490; Found 506.3488; new compound.

#### 5-Methyl-2,9-bis[(3-morpholinopropyl)amino]benzofuro[3,2-*c*]quinolin-6(5*H*)-one (**8e**)

Yield: 209.8 mg, 80%; orange oil; ^1^H NMR (300 MHz, CDCl_3_) δ 7.46 (d, *J* = 2.4 Hz, 1 H), 7.42 (d, *J* = 8.8 Hz, 1 H), 7.33 (d, *J* = 9.1 Hz, 1 H), 7.22 (d, *J* = 2.6 Hz, 1 H), 6.94 (dd, *J* = 9.1, 2.6 Hz, 1 H), 6.73 (dd, *J* = 8.8, 2.4 Hz, 1 H), 3.82–3.76 (m, 11 H), 3.36–3.29 (m, 4 H), 2.59–2.53 (m, 12 H), 1.93–1.86 (m, 4 H); ^13^C{^1^H} NMR (75 MHz, CDCl_3_) δ 159.3, 157.3, 149.0, 146.0, 144.0, 131.8, 125.8, 118.2, 116.2, 114.0, 113.4, 111.5, 110.4, 103.3, 101.9, 66.9, 66.8, 57.6, 57.5, 53.74, 53.68, 43.9, 43.6, 29.0, 25.3, 25.2; IR: 3075, 2842, 1661, 1455, 1431, 1397, 1254, 1200, 1129, 1105, 530, 500 cm^–1^; HRMS (ESI) *m*/*z*: [M + H]^+^ calcd for C_30_H_40_N_5_O_4_ 534.3075; Found 534.3067; new compound.

#### 2,9-Bis{[3-(diethylamino)propyl]amino}benzofuro[3,2-*c*]quinolin-6(5*H*)-one (**8f**)

Yield: 187.6 mg, 75%; orange oil; ^1^H NMR (300 MHz, CDCl_3_) δ 11.03 (s, 1 H), 7.48 (d, *J* = 2.4 Hz, 1 H), 7.43 (d, *J* = 8.8 Hz, 1 H), 7.33 (d, *J* = 8.8 Hz, 1 H), 7.11 (d, *J* = 2.5 Hz, 1 H), 6.88 (dd, *J* = 8.8, 2.5 Hz, 1 H), 6.73 (dd, *J* = 8.8, 2.4 Hz, 1 H), 3.29 (q, *J* = 6.4 Hz, 4 H), 2.66–2.49 (m, 12 H), 1.92–1.77 (m, 4 H), 1.09–1.04 (m, 12 H); ^13^C{^1^H} NMR (75 MHz, CDCl_3_) δ 160.5, 159.0, 149.0, 146.4, 144.7, 130.2, 125.4, 119.0, 117.4, 113.1, 113.0, 111.4, 110.6, 103.6, 100.6, 52.3, 52.1, 46.9 (2 × C), 44.6, 44.4, 26.3, 26.0, 11.9, 11.8; IR: 3344, 3081, 2994, 2811, 1691, 1440, 1265, 1221, 1145, 865, 500 cm^–1^; HRMS (ESI) *m*/*z*: [M + H]^+^ calcd for C_29_H_42_N_5_O_2_ 492.3333; Found 492.3329; new compound.

### Synthesis of 2,9-Bis{[3-(diethylamino)propyl]amino}benzofuro[3,2-*c*]quinoline (**9**)

A solution of **8f** (18.2 mg, 0.0370 mmol, 1.0 equiv) in anhydrous toluene (3.0 mL) was added with Red-Al (60% in toluene, 0.217 mL, *d* = 1.04, 1.12 mmol, 30 equiv). The reaction mixture was heated under reflux in an oil bath for 24 h. The solution was quenched with H_2_O and extracted with EtOAc. The organic layer was washed with brine, dried over anhydrous MgSO_4_, and concentrated under reduced pressure. The residue was purified by HPLC using isocratic MeOH (50%) and 0.1% formic acid in H_2_O (50%) as the eluent. The fractions of **9** were combined, concentrated, adjusted to pH 10 by 1.0 N NaOH, and extracted with CH_2_Cl_2_. The organic layer was dried over anhydrous MgSO_4_ and concentrated under reduced pressure to give **9** (15.5 mg, 0.0326 mmol) as an yellow oil in 88% yield: ^1^H NMR (300 MHz, CDCl_3_) δ 9.10 (s, 1 H), 7.97 (d, *J* = 9.1 Hz, 1 H), 7.49 (d, *J* = 8.8 Hz, 1 H), 7.19–7.13 (m, 2 H), 7.07 (dd, *J* = 9.1, 2.5 Hz, 1 H), 6.77 (dd, *J* = 8.8, 2.5 Hz, 1 H), 3.37 (t, *J* = 6.2 Hz, 2 H), 3.29 (t, *J* = 6.3 Hz, 2 H), 2.72–2.40 (m, 12 H), 1.95–1.74 (m, 4 H), 1.12–1.02 (m, 12 H); ^13^C{^1^H} NMR (75 MHz, CDCl_3_) δ 156.7, 149.3, 147.2, 146.0, 141.6, 139.5, 130.5, 123.9, 120.5, 119.1, 116.7, 114.1, 112.1, 101.7, 95.7, 52.5, 52.2, 47.0 (2 × C), 44.7, 44.1, 26.2, 25.7, 11.90, 11.85; IR: 3331, 3044, 2991, 2888, 1601, 1570, 1495, 1222, 1201, 1133, 866, 780 cm^–1^; HRMS (ESI) *m*/*z*: [M + H]^+^ calcd for C_29_H_42_N_5_O 476.3384; Found 476.3374; new compound.

### FRET Melting Assay

The potency of compounds **8a**–**f**, **9**, and CX-5461 to stabilize G4 or duplex DNA was assessed using a fluorescence resonance energy transfer (FRET) assay^38^ with slight modifications. The oligodeoxynucleotides used were G4 F21T (5′-[FAM]-GGG TTA GGG TAG GGT TAG GG-[TAMRA]-3′) and duplex T-loop (5′-[FAM]-TAT AGC TAT ATT TTT TTA TAG CTA TA-[TAMRA]-3′), where FAM is the donor fluorophore 6-carboxyfluorescein and TAMRA is the acceptor fluorophore 6-carboxytetramethylrhodamine. Original oligodeoxynucleotide stock solutions (100 μM) were prepared in nuclease-free water (not DEPC-treated). The FRET probe (0.40 μM, 1.0 mL) was prepared in a FRET buffer (60 mM KCl, 10 mM Tris-HCl, pH 7.5), annealed at 90 °C for 5.0 min, and then slowly cooled to room temperature. Compounds were dissolved in DMSO (10 mM) and diluted to the desired concentrations using a FRET buffer. Annealed DNA (15 μL, 2 × concentration, 0.025 μM) was mixed with the compound (15 μL, 2 × concentration) in a tube (0.20 mL) and incubated at room temperature for 10 min. Fluorescence intensity was measured using the Qiagen Rotor-Gene Q real-time PCR system, with excitation at λ 470 nm and detection at λ 510 nm. Readings were taken at intervals of 0.5 °C between 25 and 95 °C, with each temperature held constant for 30 s to ensure stable measurements. All experiments were performed in triplicate. The Δ*T*_m_ value for each compound was calculated as its *T*_m_ minus the *T*_m_ of the FRET buffer control.

### Cell Proliferation Assay

The anticancer activity of **8d** to inhibit the proliferation of MDA-MB-231 cancer cells was measured using CellTiter96 assay kit (Promega) as previously described.^20^ In brief, MDA-MB-231 cells were maintained in DMEM containing 10% FCS and incubated at 37 °C in 5% CO_2_. Cells were plated at a density of 4000 cells/well for 24 h, treated with different concentrations of **8d**, and incubated for an additional 72 h. The cells were added with CellTiter96 Aqueous One Solution Reagent (Promega) and incubated for another 4.0 h. Cell viability was determined by measuring absorbance at 490 nm and the data were analyzed using GraphPad Prism version 4. The GI_50_ value (0.41 ± 0.3 μM) was calculated from two independent dose–response curves.

### Computational Method

The three-dimensional structure of the propeller 22-mer G4 DNA was obtained from the Protein Data Bank (entry 1KF1).^45^ The first 5′-adenosine was deleted to match the DNA sequence of the F21T DNA used in the FRET melting assay. The structures of compounds **8a**–**f** and **9** were created using ChemDraw and optimized with the MM2 method in Chem3D. The macromolecule and small molecules were processed according to the standard procedure previously used^22^ with AutoDockTools 1.5.6, using a grid size of 60 × 60 × 60 (spacing 0.375 & A-ring;) centered on the macromolecule. Docking was performed on Autodock 4 (version 4.2)^46^ with default parameters. The docking results were analyzed by using BIOVIA Discovery Studio Visualizer (BIOVIA Software, Inc.).

## Data Availability

The data underlying this study are available in the published article and its Supporting Information.

## Supporting Information Available

Synthesis of starting materials **4** and NMR spectra of all synthesized compounds (PDF)

## References

[ref1] Dakshanamurthy S.; Kim M.; Brown M. L.; Byers S. W. In-silico fragment-based identification of novel angiogenesis inhibitors. Bioorg. Med. Chem. Lett. 2007, 17, 4551–4556. 10.1016/j.bmcl.2007.05.104. 17591441

[ref2] Xiao Z.; Waters N. C.; Woodard C. L.; Li Z.; Li P.-K. Design and synthesis of Pfmrk inhibitors as potential antimalarial agents. Bioorg. Med. Chem. Lett. 2001, 11, 2875–2878. 10.1016/S0960-894X(01)00578-9. 11597420

[ref3] Kawashima K.; Inoue T.; Tsutsumi N.; Endo H. Effect of KCA4098 on the function of osteoblast-like cells and the formation of TRAP-positive multinucleated cells in a mouse bone marrow cell population. Biochem. Pharmacol. 1996, 51, 133–139. 10.1016/0006-2952(95)02126-4. 8615881

[ref4] Zhang W.; Liu L.-l.; Lun S.; Wang S.-S.; Xiao S.; Gunosewoyo H.; Yang F.; Tang J.; Bishai W. R.; Yu L.-F. Design and synthesis of mycobacterial pks13 inhibitors: conformationally rigid tetracyclic molecules. Eur. J. Med. Chem. 2021, 213, 113202 10.1016/j.ejmech.2021.113202. 33516983 PMC8689393

[ref5] Lin Y.; Xing D.; Wu W.-B.; Xu G.-Y.; Yu L.-F.; Tang J.; Zhou Y.-B.; Li J.; Yang F. Design, synthesis, and in vitro evaluation of benzofuro[3,2-*c*]quinoline derivatives as potential antileukemia agents. Molecules 2020, 25, 203 10.3390/molecules25010203. 31947824 PMC6983037

[ref6] Shanahan R. M.; Hickey A.; Reen F. J.; O’Gara F.; McGlacken G. P. Synthesis of benzofuroquinolines via phosphine-free direct arylation of 4-phenoxyquinolines in air. Eur. J. Org. Chem. 2018, 2018, 6140–6149. 10.1002/ejoc.201800923.

[ref7] Walser A.; Silverman G.; Flynn T.; Fryer R. I. Nucleophilic displacement of aromatic fluoride, part III, indoloquinolines and benzofuranoquinolines. Heterocycl. Chem. 1975, 12, 351–358. 10.1002/jhet.5570120227.

[ref8] Kawase Y.; Yamaguchi S.; Morita M.; Uesugi T. The synthesis of benzofuroquinolines. II. Two benzofuroquinolinones and some benzofuroquinoline derivatives. Bull. Chem. Soc. Jpn. 1980, 53, 1057–1060. 10.1246/bcsj.53.1057.

[ref9] Stadlbauer W.; Schmut O.; Kappe T. Synthese von benzofuranen durch cyclodehydrierung von phenylmalonylheterocyclen. Monatsh. Chem. 1980, 111, 1005–1013. 10.1007/BF00909657.

[ref10] Kappe T.; Brandner A.; Stadlbauer W. Synthese von kondensierten benzofuranen durch dehydratisierung cyclischer phenyl-β-dicarbonylverbindungen. Monatsh. Chem. 1987, 118, 1177–1184. 10.1007/BF00811290.

[ref11] Stadlbauer W.; Laschober R.; Kappe T. Palladium-katalysierte ringschlußreaktionen zu benzofuranen: ein neuer und effektiver zugang zu azacumöstrolderivaten. Liebigs Ann. Chem. 1990, 1990, 531–539. 10.1002/jlac.1990199001101.

[ref12] Yamaguchi S.; Uchiuzoh Y.; Sanada K. The synthesis of benzofuroquinolines. IX. A benzofuroisoquinolinone and a benzofuroisocoumarin. J. Heterocycl. Chem. 1995, 32, 419–423. 10.1002/jhet.5570320207.

[ref13] Li H.; Yang H.; Petersen J. L.; Wang K. K. Biradicals/zwitterions from thermolysis of enyne–isocyanates. Application to the synthesis of 2(1*H*)-pyridones, benzofuro[3,2-*c*]pyridin-1(2*H*)-ones, 2,5-dihydro-1*H*-pyrido[4,3-*b*]indol-1-ones, and related compounds. J. Org. Chem. 2004, 69, 4500–4508. 10.1021/jo049716t. 15202908

[ref14] Liang D.; Hu Z.; Peng J.; Huang J.; Zhu Q. Synthesis of phenanthridinones *via* palladium-catalyzed C(sp^2^)–H aminocarbonylation of unprotected *o*-arylanilines. Chem. Commun. 2013, 49, 173–175. 10.1039/C2CC36817J. 23168677

[ref15] Wang S.; Shao P.; Du G.; Xi C. MeOTf- and TBD-mediated carbonylation of *ortho*-arylanilines with CO_2_ leading to phenanthridinones. J. Org. Chem. 2016, 81, 6672–6676. 10.1021/acs.joc.6b01318. 27398605

[ref16] Ali W.; Modi A.; Behera A.; Mohanta P. R.; Patel B. K. Cs_2_CO_3_ as a source of carbonyl and ethereal oxygen in Cu-catalysed cascade synthesis of benzofuran[3,2-*c*]quinolin-6[5-*H*]ones. Org. Biomol. Chem. 2016, 14, 5940–5944. 10.1039/C6OB01029F. 27253673

[ref17] Ding D.; Zhu G.; Jiang X. Ligand-controlled palladium(II)-catalyzed regiodivergent carbonylation of alkynes: syntheses of indolo[3,2-*c*]coumarins and benzofuro[3,2-*c*]quinolinones. Angew. Chem., Int. Ed. 2018, 57, 9028–9032. 10.1002/anie.201804788. 29851274

[ref18] Li X.; Hu L.; Ma S.; Yu H.; Lu G.; Xu T. Divergent Rh catalysis: asymmetric dearomatization versus C–H activation initiated by C–C activation. ACS Catal. 2023, 13, 4873–4881. 10.1021/acscatal.3c00063.

[ref19] Wang F.; Pan J.-Q.; Shi R.-X.; Ning R.; Wu M. Diastereoselective synthesis of dihydrobenzofuran spirooxindoles and their transformation into benzofuroquinolinones by ring expansion of oxindole core. J. Org. Chem. 2024, 89, 5142–5147. 10.1021/acs.joc.3c02956. 38545874

[ref20] Ding D.; Mou T.; Feng M.; Jiang X. Utility of ligand effect in homogenous gold catalysis: enabling regiodivergent π-bond-activated cyclization. J. Am. Chem. Soc. 2016, 138, 5218–5221. 10.1021/jacs.6b01707. 27058740

[ref21] Ding D.; Mou T.; Xue J.; Jiang X. Access to divergent benzo-heterocycles *via* a catalyst-dependent strategy in the controllable cyclization of *o*-alkynyl-*N*-methoxyl-benzamides. Chem. Commun. 2017, 53, 5279–5282. 10.1039/C7CC01861D. 28443851

[ref22] Hsueh W.-Y.; Lee Y.-S. E.; Huang M.-S.; Lai C.-H.; Gao Y.-S.; Lin J.-C.; Chen Y.-F.; Chang C.-L.; Chou S.-Y.; Chen S.-F.; Lu Y.-Y.; Chang L.-H.; Lin S. F.; Lin Y.-H.; Hsu P.-C.; Wei W.-Y.; Huang Y.-C.; Kao Y.-F.; Teng L.-W.; Liu H.-H.; Chen Y.-C.; Yuan T.-T.; Chan Y.-W.; Huang P.-H.; Chao Y.-T.; Huang S.-Y.; Jian B.-H.; Huang H.-Y.; Yang S.-C.; Lo T.-H.; Huang G.-R.; Wang S.-Y.; Lin H.-S.; Chuang S.-H.; Huang J.-J. Copper(I)-catalyzed nitrile-addition/*N*-arylation ring-closure cascade: synthesis of 5,11-dihydro-6*H*-indolo[3,2-*c*]quinolin-6-ones as potent topoisomerase-I inhibitors. J. Med. Chem. 2021, 64, 1435–1453. 10.1021/acs.jmedchem.0c00727. 33492141

[ref23] Burge S.; Parkinson G. N.; Hazel P.; Todd A. K.; Neidel S. Quadruplex DNA: sequence, topology and structure. Nucleic Acids Res. 2006, 34, 5402–5415. 10.1093/nar/gkl655. 17012276 PMC1636468

[ref24] Bochman M. L.; Paeschke K.; Zakian V. A. DNA secondary structures: stability and function of G-quadruplex structures. Nat. Rev. Genet. 2012, 13, 770–780. 10.1038/nrg3296. 23032257 PMC3725559

[ref25] Chen L.; Dickerhoff J.; Sakai S.; Yang D. DNA G-quadruplex in human telomeres and oncogene promoters: structures, functions, and small molecule targeting. Acc. Chem. Res. 2022, 55, 2628–2646. 10.1021/acs.accounts.2c00337. 36054116 PMC9937053

[ref26] Han H.; Hurley L. H. G-quadruplex DNA: a potential target for anti-cancer drug design. Trends Pharmacol. Sci. 2000, 21, 136–142. 10.1016/S0165-6147(00)01457-7. 10740289

[ref27] Balasubramanian S.; Neidle S. G-quadruplex nucleic acids as therapeutic targets. Curr. Opin. Chem. Biol. 2009, 13, 345–353. 10.1016/j.cbpa.2009.04.637. 19515602 PMC2726962

[ref28] Neidle S. Human telomeric G-quadruplex: the current status of telomeric G-quadruplexes as therapeutic targets in human cancer. FEBS J. 2010, 277, 1118–1125. 10.1111/j.1742-4658.2009.07463.x. 19951354

[ref29] Carvalho J.; Mergny J.-L.; Salgado G. F.; Queiroz J. A.; Cruz C. G-quadruplex, friend or foe: the role of the G-quartet in anticancer strategies. Trends Mol. Med. 2020, 26, 848–861. 10.1016/j.molmed.2020.05.002. 32467069

[ref30] Izakovich E. N.; Khidekel’ M. L. Coordination compounds of transition metals in the chemistry of aromatic nitro-compounds. Russ. Chem. Rev. 1988, 57, 419–432. 10.1070/RC1988v057n05ABEH003360.

[ref31] Arola A.; Vilar R. Stabilisation of G-quadruplex DNA by small molecules. Curr. Top. Med. Chem. 2008, 8, 1405–1415. 10.2174/156802608786141106. 18991726

[ref32] Monchaud D.; Teulade-Fichou M.-P. A hitchhiker’s guide to G-quadruplex ligands. Org. Biomol. Chem. 2008, 6, 627–636. 10.1039/B714772B. 18264563

[ref33] Ma D.-L.; Zhang Z.; Wang M.; Lu L.; Zhong H.-J.; Leung C.-H. Recent developments in G-quadruplex probes. Chem. Biol. 2015, 22, 812–828. 10.1016/j.chembiol.2015.06.016. 26190823

[ref34] Duarte A. R.; Cadoni E.; Ressurreição A. S.; Moreira R.; Paulo A. Design of modular G-quadruplex ligands. ChemMedChem 2018, 13, 869–893. 10.1002/cmdc.201700747. 29512884

[ref35] Asamitsu S.; Obata S.; Yu Z.; Bando T.; Sugiyama H. Recent progress of targeted G-quadruplex-preferred ligands toward cancer therapy. Molecules 2019, 24, 429 10.3390/molecules24030429. 30682877 PMC6384606

[ref36] Read M.; Harrison R. J.; Romagnoli B.; Tanious F. A.; Gowan S. H.; Reszka A. P.; Wilson W. D.; Kelland L. R.; Neidle S. Structure-based design of selective and potent G quadruplex-mediated telomerase inhibitors. Proc. Natl. Acad. Sci. U.S.A. 2001, 98, 4844–4849. 10.1073/pnas.081560598. 11309493 PMC33125

[ref37] Han H.; Cliff C. L.; Hurley L. H. Accelerated assembly of G-quadruplex structures by a small molecule. Biochemistry 1999, 38, 6981–6986. 10.1021/bi9905922. 10353809

[ref38] Guyen B.; Schultes C. M.; Hazel P.; Mann J.; Neidle S. Synthesis and evaluation of analogues of 10*H*-indolo[3,2-*b*]quinoline as G-quadruplex stabilising ligands and potential inhibitors of the enzyme telomerase. Org. Biomol. Chem. 2004, 2, 981–988. 10.1039/b316055f. 15034620

[ref39] Lavrado J.; Borralho P. M.; Ohnmacht S. A.; Castro R. E.; Rodrigues C. M. P.; Moreira R.; dos Santos D. J. V. A.; Neidle S.; Paulo A. Synthesis, G-quadruplex stabilisation, docking studies, and effect on cancer cells of indolo[3,2-*b*]quinolines with one, two, or three basic side chains. ChemMedChem 2013, 8, 1648–1661. 10.1002/cmdc.201300288. 23960016

[ref40] Lavrado J.; Ohnmacht S. A.; Correia I.; Leitão C.; Pisco S.; Gunaratnam M.; Moreira R.; Neidle S.; dos Santos D. J. V. A.; Paulo A. Indolo[3,2-*c*]quinoline G-quadruplex stabilizers: a structural analysis of binding to the human telomeric G-quadruplex. ChemMedChem 2015, 10, 836–849. 10.1002/cmdc.201500067. 25820698

[ref41] Mendes E.; Bahls B.; Aljnadi I. M.; Paulo A. Indoloquinolines as scaffolds for the design of potent G-quadruplex ligands. Bioorg. Med. Chem. Lett. 2022, 72, 128862 10.1016/j.bmcl.2022.128862. 35716866

[ref42] Xu H.; di Antonio M.; McKinney S.; Mathew V.; Ho B.; O’Neil N. J.; dos Santos N.; Silvester J.; Wei V.; Garcia J.; Kabeer F.; Lai D.; Soriano P.; Banáth J.; Chiu D. S.; Yap D.; Le D. D.; Ye F. B.; Zhang A.; Thu K.; Soong J.; Lin S.-c.; Tsai A. H. C.; Osako T.; Algara T.; Saunders D. N.; Wong J.; Xian J.; Bally M. B.; Brenton J. D.; Brown G. W.; Shah S. P.; Cescon D.; Mak T. W.; Caldas C.; Stirling P. C.; Hieter P.; Balasubramanian S.; Aparicio S. CX-5461 is a DNA G-quadruplex stabilizer with selective lethality in BRCA1/2 deficient tumours. Nat. Commun. 2017, 8, 14432 10.1038/ncomms14432. 28211448 PMC5321743

[ref43] Xu H.; Hurley L. H. A first-in-class clinical G-quadruplex-targeting drug. The bench-to-bedside translation of the fluoroquinolone QQ58 to CX-5461 (Pidnarulex). Bioorg. Med. Chem. Lett. 2022, 77, 129016 10.1016/j.bmcl.2022.129016. 36195286

[ref44] Jin M.; Hurley L. H.; Xu H. A synthetic lethal approach to drug targeting of G-quadruplexes based on CX-5461. Bioorg. Med. Chem. Lett. 2023, 91, 129384 10.1016/j.bmcl.2023.129384. 37339720

[ref45] Parkinson G. N.; Lee M. P. H.; Neidle S. Crystal structure of parallel quadruplexes from human telomeric DNA. Nature 2002, 417, 876–880. 10.1038/nature755. 12050675

[ref46] Morris G. M.; Huey R.; Lindstrom W.; Sanner M. F.; Belew R. K.; Goodsell D. S.; Olson A. J. AutoDock4 and AutoDockTools4: automated docking with selective receptor flexibility. J. Comput. Chem. 2009, 30, 2785–2791. 10.1002/jcc.21256. 19399780 PMC2760638

