# Supplementary material for: Copper(I)-Catalyzed Intramolecular Tandem Acylation/O-Arylation under Mild Conditions: Synthesis of Benzofuro[3,2-c]quinolin-6(5H)-ones and G-Quadruplex-Targeting Analogues
Source: J Org Chem. 2024 Dec 27;90(1):794–805. doi: 10.1021/acs.joc.4c02813 (PMC11731314; doi:10.1021/acs.joc.4c02813)

Supporting Information

Copper(I)-Catalyzed Intramolecular Tandem Acylation/*O*-Arylation Under Mild Conditions: Synthesis of Benzofuro[3,2-*c*]quinolin-6(5*H*)-ones and G-Quadruplex-Targeting Analogs

Show-Jen Chiou,[‡] Yi-Chien Lin,[§] Yi-Fu Chang,[‡] Yu-Fen Chen,[‡] Tzu-Hao Lo,[‡] Chia-Shen Tsai,[‡] Hung-Chun Liao,[‡] Pei-Yi Tsai,[§] Shih-Hsien Chuang,[§] and Jiann-Jyh Huang,[†,‡,*]

[†]*Institute of BioPharmaceutical Sciences, National Sun Yat-sen University, No. 70, Lien-hai Rd., Kaohsiung 804201, Taiwan*

[‡]*Department of Applied Chemistry, National Chiayi University, No. 300, Syuefu Rd., Chiayi City 60004, Taiwan*

[§]*Development Center for Biotechnology, National Biotechnology Research Park, Taipei City 11571, Taiwan*

*Corresponding author: Tel.: +886-7-525-2000 ext. 7206; Fax: +886-7-525-0197; Email: lukehuang@mail.nsysu.edu.tw (Jiann-Jyh Huang)

Table of Contents: Page

Synthesis of Methyl 2-[2-(2-Bromophenyl)acetamido]benzoates 4a–z and 4A–E S2

^{1}H and ^{13}C{^{1}H} NMR Spectra of the Synthesized Compounds 1a–z, 1A–E, 7a, 10, 8a–f, 9, 4a–z, and 4A–E S13

Experimental Section

General Information. All reagents and starting materials were used as purchased without further purification. The melting point was determined on a STUART SMP3 apparatus. Proton NMR spectra were recorded on Varian Mercury-300 (300 MHz) or Agilent 400-MR (400 MHz) spectrometer. Carbon-13 NMR spectra were obtained on Varian Mercury-300 (75 MHz), Agilent 400-MR (100 MHz), or JEOL ECZ500R/S1 (125 MHz) spectrometer. $CDCl_3$ or DMSO-d_6 served as the solvent for NMR measurements. Proton NMR chemical shifts were referenced to the residual $CHCl_3$ (δ 7.26 ppm) in $CDCl_3$ or the central peak of CHD_2SOCD_3 (δ 2.49 ppm) in DMSO-d_6. Carbon-13 NMR chemical shifts were referenced to the central peak of $CDCl_3$ (δ 77.0 ppm) or DMSO-d_6 (δ 39.5 ppm). Multiplicities are abbreviated as follows: s, singlet; d, doublet; t, triplet; q, quartet; m, multiplet; *J*, coupling constant (Hz). Infrared (IR) spectra were recorded on a PerkinElmer One FT-IR spectrometer with an ATR accessory. High-resolution mass spectra were obtained using an LTQ Orbitrap XL mass spectrometer (Thermo Fisher Scientific).

Synthesis of Methyl 2-[2-(2-Bromophenyl)acetamido]benzoates 4a–z and 4A–E

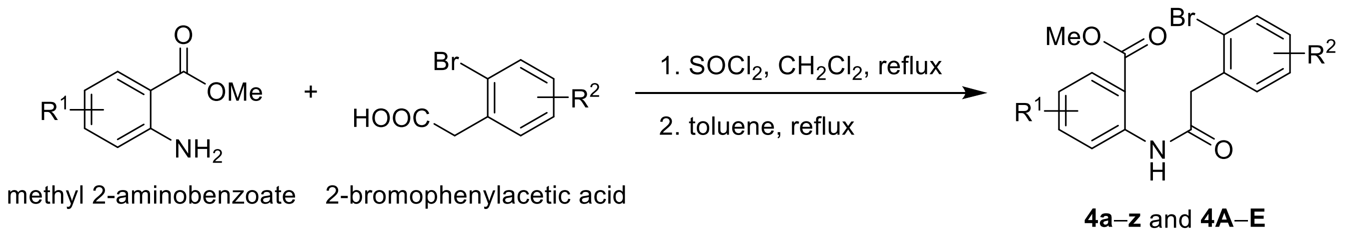

4a–**z** and **4A**–**E**

General Procedure. A solution of 2-bromophenylacetic acid (2.0 mmol, 1.0 equiv) in CH_2Cl_2 (10 mL) was slowly added with $SOCl_2$ (8.0 mmol, 4.0 equiv). The reaction mixture was heated under reflux in an oil bath for 4.0 h. The solution was concentrated under reduced pressure and the residue was re-dissolved in toluene (10 mL). The solution was added with methyl 2-aminobenzoate (2.2 mmol, 1.1 equiv) and heated under reflux in an oil bath for 16 h. The solution was concentrated under reduced pressure and the residue was recrystallized in MeOH to give targets **4a**–**z** and **4A**–**E** in 81–99% yields.

*Methyl 2-[2-(2-Bromophenyl)acetamido]benzoate (**4a**).* Yield: 614.2 mg, 88%; white solids; mp 98.0–98.5 °C; ^{1}H NMR (400 MHz, $CDCl_3$) *δ* 11.02 (s, 1 H), 8.72 (dd, *J* = 8.6, 1.2 Hz, 1 H), 7.99 (dd, *J* = 8.3,

1.7 Hz, 1 H), 7.61 (dd, J = 7.8, 1.2 Hz, 1 H), 7.52 (ddd, J = 8.6, 7.3, 1.7 Hz, 1 H), 7.43 (dd, J = 7.8, 1.8 Hz, 1 H), 7.35 (td, J = 7.8, 1.2 Hz, 1 H), 7.19 (td, J = 7.8, 1.8 Hz, 1 H), 7.07 (ddd, J = 8.3, 7.3, 1.2 Hz, 1 H), 3.94 (s, 2 H), 3.85 (s, 3 H); $^{13}C\{^1H\}$ NMR (100 MHz, $CDCl_3$) δ 168.7, 168.3, 141.2, 134.5, 134.3, 133.0, 131.9, 130.7, 129.1, 127.8, 125.3, 122.7, 120.5, 115.3, 52.2, 45.7; IR: 3253, 2956, 2909, 1703, 1682, 1584, 1529, 1436, 1261 cm^{-1}; HRMS (ESI) m/z: $[M + H]^+$ Calcd for $C_{16}H_{15}BrNO_3$ 348.0230; Found 348.0224; CAS # 1345540-20-6.

*Methyl 2-[2-(2-Bromophenyl)acetamido]-5-fluorobenzoate (**4b**).* Yield: 591.0 mg, 81%; white solids; mp 109.5–110.3 °C; ^1H NMR (400 MHz, $CDCl_3$) δ 10.84 (s, 1 H), 8.72 (dd, J = 9.4, 5.1 Hz, 1 H), 7.66 (dd, J = 9.2, 3.1 Hz, 1 H), 7.61 (dd, J = 8.0, 1.3 Hz, 1 H), 7.42 (dd, J = 7.6, 1.8 Hz, 1 H), 7.35 (td, J = 7.6, 1.3 Hz, 1 H), 7.26–7.16 (m, 2 H), 3.93 (s, 2 H), 3.85 (s, 3 H); $^{13}C\{^1H\}$ NMR (100 MHz, $CDCl_3$) δ 168.6, 167.3, 157.4 (d, J = 243.7 Hz), 137.5, 134.1, 133.1, 132.0, 129.2, 127.9, 125.3, 122.4 (d, J = 7.2 Hz), 121.5 (d, J = 22.0 Hz), 116.9, 116.7, 52.5, 45.6; IR: 3331, 3063, 2952, 1682, 1529, 1427, 1291, 1228, 739, 492 cm^{-1}; HRMS (ESI) m/z: $[M + H]^+$ Calcd for $C_{16}H_{14}BrFNO_3$ 366.0136; Found 366.0129; new compound.

*Methyl 2-[2-(2-Bromophenyl)acetamido]-5-chlorobenzoate (**4c**).* Yield: 622.7 mg, 81%; white solids; mp 117.5–118.2 °C; ^1H NMR (400 MHz, $CDCl_3$) δ 10.93 (s, 1 H), 8.71 (d, J = 9.1 Hz, 1 H), 7.95 (d, J = 2.6 Hz, 1 H), 7.61 (dd, J = 7.6, 1.3 Hz, 1 H), 7.47 (dd, J = 9.1, 2.6 Hz, 1 H), 7.42 (dd, J = 7.6, 1.8 Hz, 1 H), 7.35 (td, J = 7.6, 1.3 Hz, 1 H), 7.20 (td, J = 7.6, 1.8 Hz, 1 H), 3.93 (s, 2 H), 3.85 (s, 3 H); $^{13}C\{^1H\}$ NMR (100 MHz, $CDCl_3$) δ 168.7, 167.2, 139.8, 134.3, 134.0, 133.1, 132.0, 130.3, 129.2, 127.9, 127.7, 125.3, 121.9, 116.5, 52.5, 45.7; IR: 3326, 3058, 2952, 1707, 1584, 1218, 1073, 739, 662, 454 cm^{-1}; HRMS (ESI) m/z: $[M + H]^+$ Calcd for $C_{16}H_{14}BrClNO_3$ 381.9840; Found 381.9834; new compound.

*Methyl 5-Bromo-2-[2-(2-bromophenyl)acetamido]benzoate (**4d**).* Yield: 711.5 mg, 83%; white solids; mp 168.0–169.0 °C; ^1H NMR (400 MHz, $CDCl_3$) δ 10.93 (s, 1 H), 8.65 (d, J = 9.1 Hz, 1 H), 8.10 (d, J = 2.4 Hz, 1 H), 7.64–7.57 (m, 2 H), 7.43–7.32 (m, 2 H), 7.24–7.17 (m, 1 H), 3.93 (s, 2 H), 3.85 (s, 3 H); $^{13}C\{^1H\}$ NMR (75 MHz, $CDCl_3$) δ 168.8, 167.2, 140.2, 137.2, 134.0, 133.3, 133.1, 132.0, 129.3, 127.9, 125.3,

122.2, 116.8, 115.1, 52.5, 45.7; IR: 3282, 3236, 3128, 2911, 1703, 1588, 1527, 1433, 1263, 1087, 881, 471 cm^{-1}; HRMS (ESI) *m*/*z*: $[M + H]^+$ Calcd for $C_{16}H_{14}Br_2NO_3$ 425.9335; Found 425.9330; new compound.

*Methyl 2-[2-(2-Bromophenyl)acetamido]-5-iodobenzoate (**4e**).* Yield: 849.8 mg, 90%; brown solids; mp 119.4–119.8 °C; ^{1}H NMR (400 MHz, $CDCl_3$) δ 10.94 (s, 1 H), 8.52 (d, J = 9.0 Hz, 1 H), 8.29 (d, J = 2.2 Hz, 1 H), 7.78 (dd, J = 9.0, 2.2 Hz, 1 H), 7.61 (d, J = 7.8 Hz, 1 H), 7.41 (dd, J = 7.8, 1.6 Hz, 1 H), 7.35 (td, J = 7.8, 1.1 Hz, 1 H), 7.20 (td, J = 7.8, 1.6 Hz, 1 H), 3.92 (s, 2 H), 3.85 (s, 3 H); $^{13}C\{^1H\}$ NMR (75 MHz, $CDCl_3$) δ 168.8, 167.1, 143.0, 140.8, 139.2, 134.0, 133.1, 132.0, 129.3, 127.9, 125.3, 122.4, 117.1, 85.2, 52.5, 45.7; IR: 3469, 3320, 3110, 2948, 1710, 1680, 1510, 1228, 1076, 790, 471 cm^{-1}; HRMS (ESI) *m*/*z*: $[M + H]^+$ Calcd for $C_{16}H_{14}BrINO_3$ 473.9196; Found 473.9193; new compound.

*Methyl 2-[2-(2-Bromophenyl)acetamido]-5-nitrobenzoate (**4f**).* Yield: 700.1 mg, 89%; yellow solids; mp 134.0–134.8 °C; ^{1}H NMR (400 MHz, $CDCl_3$) δ 11.35 (s, 1 H), 8.96 (d, J = 9.4 Hz, 1 H), 8.89 (d, J = 2.8 Hz, 1 H), 8.36 (dd, J = 9.4, 2.8 Hz, 1 H), 7.63 (dd, J = 7.8, 1.3 Hz, 1 H), 7.42 (dd, J = 7.8, 1.9 Hz, 1 H), 7.37 (td, J = 7.8, 1.3 Hz, 1 H), 7.23 (td, J = 7.8, 1.9 Hz, 1 H), 3.99 (s, 2 H), 3.92 (s, 3 H); $^{13}C\{^1H\}$ NMR (100 MHz, $CDCl_3$) δ 169.5, 167.0, 146.4, 142.0, 133.6, 133.3, 132.2, 129.7, 129.5, 128.2, 126.9, 125.5, 120.7, 115.2, 53.1, 46.0; IR: 3278, 3103, 2951, 1714, 1689, 1584, 1495, 1340, 1262, 1149, 749, 525 cm^{-1}; HRMS (ESI) *m*/*z*: $[M + H]^+$ Calcd for $C_{16}H_{14}BrN_2O_5$ 393.0081; Found 393.0075; new compound.

*Methyl 2-[2-(2-Bromophenyl)acetamido]-5-methoxybenzoate (**4g**).* Yield: 717.6 mg, 95%; brown solids; mp 135.8–136.8 °C; ^{1}H NMR (400 MHz, $CDCl_3$) δ 10.70 (s, 1 H), 8.62 (d, J = 9.2 Hz, 1 H), 7.59 (dd, J = 7.8, 1.3 Hz, 1 H), 7.45 (d, J = 3.1 Hz, 1 H), 7.41 (dd, J = 7.8, 1.8 Hz, 1 H), 7.33 (td, J = 7.8, 1.3 Hz, 1 H), 7.17 (td, J = 7.8, 1.8 Hz, 1 H), 7.08 (dd, J = 9.2, 3.1 Hz, 1 H), 3.90 (s, 2 H), 3.82 (s, 3 H), 3.78 (s, 3 H); $^{13}C\{^1H\}$ NMR (75 MHz, $CDCl_3$) δ 168.3, 168.0, 154.6, 134.8, 134.4, 133.0, 132.0, 129.1, 127.8, 125.4, 122.1, 120.7, 116.4, 114.6, 55.6, 52.3, 45.6; IR: 3307, 3262, 3083, 3009, 2835, 1704, 1601, 1527, 1219, 1083, 739, 520 cm^{-1}; HRMS (ESI) *m*/*z*: $[M + H]^+$ Calcd for $C_{17}H_{17}BrNO_4$ 378.0335; Found 378.0329; new compound.

*Methyl 2-[2-(2-Bromophenyl)acetamido]-4-fluorobenzoate **(4h)***. Yield: 652.8 mg, 89%; white solids; mp 105.3–105.6 °C; ^{1}H NMR (400 MHz, $CDCl_3$) δ 11.19 (s, 1 H), 8.54 (dd, J = 12.0, 2.6 Hz, 1 H), 7.99 (dd, J = 8.9, 6.5 Hz, 1 H), 7.60 (dd, J = 7.8, 1.2 Hz, 1 H), 7.40 (dd, J = 7.8, 1.8 Hz, 1 H), 7.34 (td, J = 7.8, 1.2 Hz, 1 H), 7.19 (td, J = 7.8, 1.8 Hz, 1 H), 6.77–6.72 (m, 1 H), 3.93 (s, 2 H), 3.83 (s, 3 H); ^{13}C{^{1}H} NMR (100 MHz, $CDCl_3$) δ 169.1, 167.8, 166.2 (d, J = 254.0 Hz), 143.5, 134.0, 133.1 (2 × C), 132.0, 129.3, 127.9, 125.4, 111.4, 110.1 (d, J = 22.6 Hz), 107.7 (d, J = 28.8 Hz), 52.4, 45.7; IR: 3236, 3113, 2910, 1721, 1677, 1439, 1257, 1181, 887, 739, 523 cm^{-1}; HRMS (ESI) *m/z*: $[M + H]^+$ Calcd for $C_{16}H_{14}BrFNO_3$ 366.0136; Found 366.0131; new compound.

*Methyl 2-[2-(2-Bromophenyl)acetamido]-4-chlorobenzoate **(4i)***. Yield: 613.3 mg, 80%; white solids; mp 115.5–116.2 °C; ^{1}H NMR (400 MHz, $CDCl_3$) δ 11.09 (s, 1 H), 8.84 (dd, J = 2.2, 0.9 Hz, 1 H), 7.92 (dd, J = 8.6, 0.9 Hz, 1 H), 7.61 (d, J = 7.7 Hz, 1 H), 7.42 (dd, J = 7.7, 1.8 Hz, 1 H), 7.35 (t, J = 7.7 Hz, 1 H), 7.20 (td, J = 7.7, 1.8 Hz, 1 H), 7.04 (dd, J = 8.6, 0.9 Hz, 1 H), 3.94 (s, 2 H), 3.85 (s, 3 H); ^{13}C{^{1}H} NMR (75 MHz, $CDCl_3$) δ 168.9, 167.8, 142.1, 140.9, 134.0, 133.1, 132.0, 131.8, 129.3, 127.9, 125.3, 122.9, 120.4, 113.4, 52.4, 45.6; IR: 3400, 3243, 3125, 2954, 2847, 1709, 1578, 1429, 1308, 1250, 690, 457 cm^{-1}; HRMS (ESI) *m/z*: $[M + H]^+$ Calcd for $C_{16}H_{14}BrClNO_3$ 381.9840; Found 381.9835; new compound.

*Methyl 4-Bromo-2-[2-(2-bromophenyl)acetamido]benzoate **(4j)***. Yield: 701.4 mg, 82%; brown solids; mp 108.2–108.9 °C; ^{1}H NMR (400 MHz, $CDCl_3$) δ 11.07 (s, 1 H), 9.00 (dd, J = 2.0, 1.0 Hz, 1 H), 7.83 (dd, J = 8.6, 1.0 Hz, 1 H), 7.61 (d, J = 7.8 Hz, 1 H), 7.41 (dd, J = 7.8, 1.8 Hz, 1 H), 7.35 (td, J = 7.8, 1.2 Hz, 1 H), 7.22–7.18 (m, 2 H), 3.94 (s, 2 H), 3.85 (s, 3 H); ^{13}C{^{1}H} NMR (75 MHz, $CDCl_3$) δ 168.9, 167.9, 142.0, 134.0, 133.1, 132.0, 131.8, 129.6, 129.3, 127.9, 125.9, 125.3, 123.4, 113.8, 52.4, 45.6; IR: 3236, 3120, 3012, 2954, 2907, 1705, 1684, 1575, 1246, 1097, 770, 552 cm^{-1}; HRMS (ESI) *m/z*: $[M + H]^+$ Calcd for $C_{16}H_{14}Br_2NO_3$ 425.9335; Found 425.9331; new compound.

*Methyl 2-[2-(2-Bromophenyl)acetamido]-4-methylbenzoate **(4k)***. Yield: 715.8 mg, 99%; light yellow solids; mp 110.1–110.6 °C; ^{1}H NMR (400 MHz, $CDCl_3$) δ 11.06 (s, 1 H), 8.56 (d, J = 0.7 Hz, 1 H), 7.86 (d, J = 8.1 Hz, 1 H), 7.59 (dd, J = 7.7, 1.3 Hz, 1 H), 7.41 (dd, J = 7.7, 1.8 Hz, 1 H), 7.32 (td, J = 7.7, 1.3

Hz, 1 H), 7.17 (td, J = 7.7, 1.8 Hz, 1 H), 6.86 (ddd, J = 8.1, 1.7, 0.7 Hz, 1 H), 3.92 (s, 2 H), 3.82 (s, 3 H), 2.35 (s, 3 H); $^{13}C\{^{1}H\}$ NMR (100 MHz, $CDCl_3$) δ 168.8, 168.5, 145.8, 141.2, 134.4, 133.1, 132.0, 130.7, 129.1, 127.9, 125.4, 123.7, 120.9, 112.7, 52.2, 45.7, 22.1; IR: 3313, 3051, 2952, 1703, 1682, 1579, 1409, 1254, 1087, 919, 658, 425 cm^{-1}; HRMS (ESI) m/z: $[M + H]^+$ Calcd for $C_{17}H_{17}BrNO_3$ 362.0386; Found 362.0380; new compound.

*Methyl 2-[2-(2-Bromophenyl)acetamido]-4-nitrobenzoate (**4l**).* Yield: 669.4 mg, 85%; orange solids; mp 117.6–118.6 °C; ^{1}H NMR (400 MHz, $CDCl_3$) δ 11.06 (s, 1 H), 9.63 (d, J = 2.3 Hz, 1 H), 8.15 (d, J = 8.8 Hz, 1 H), 7.86 (dd, J = 8.8, 2.3 Hz, 1 H), 7.63 (dd, J = 7.8, 1.3 Hz, 1 H), 7.47–7.35 (m, 2 H), 7.22 (td, J = 7.8, 1.9 Hz, 1 H), 3.98 (s, 2 H), 3.91 (s, 3 H); $^{13}C\{^{1}H\}$ NMR (75 MHz, $CDCl_3$) δ 169.1, 166.9, 151.2, 142.1, 133.6, 133.2, 132.0, 131.9, 129.5, 128.0, 125.3, 119.6, 116.8, 115.5, 53.0, 45.6; IR: 3494, 3384, 3275, 2961, 1697, 1529, 1257, 1073, 894, 823, 734, 577 cm^{-1}; HRMS (ESI) m/z: $[M + H]^+$ Calcd for $C_{16}H_{14}BrN_2O_5$ 393.0081; Found 393.0077; new compound.

*Methyl 2-[2-(2-Bromophenyl)acetamido]-4-methoxybenzoate (**4m**).* Yield: 695.9 mg, 92%; white solids; mp 103.5–104.0 °C; ^{1}H NMR (300 MHz, $CDCl_3$) δ 11.34 (s, 1 H), 8.41 (d, J = 2.6 Hz, 1 H), 7.92 (dd, J = 9.0, 0.8 Hz, 1 H), 7.61 (dd, J = 7.8, 1.3 Hz, 1 H), 7.42 (dd, J = 7.8, 1.8 Hz, 1 H), 7.34 (td, J = 7.8, 1.3 Hz, 1 H), 7.18 (td, J = 7.8, 1.8 Hz, 1 H), 6.59 (dd, J = 9.0, 2.6 Hz, 1 H), 3.95 (s, 2 H), 3.84 (s, 3 H), 3.83 (s, 3 H); $^{13}C\{^{1}H\}$ NMR (75 MHz, $CDCl_3$) δ 169.0, 168.3, 164.4, 143.5, 134.3, 133.0, 132.3, 132.0, 129.1, 127.8, 125.3, 110.3, 107.6, 103.81, 55.5, 51.9, 45.7; IR: 3247, 3115, 3005, 2948, 2837, 1686, 1581, 1433, 1246, 1042, 746, 477 cm^{-1}; HRMS (ESI) m/z: $[M + H]^+$ Calcd for $C_{17}H_{17}BrNO_4$ 378.0335; Found 378.0330; new compound.

*Methyl 2-[2-(2-Bromophenyl)acetamido]-4,5-dimethoxybenzoate (**4n**).* Yield: 769.1 mg, 94%; white solids; mp 122.0–122.8 °C; ^{1}H NMR (400 MHz, $CDCl_3$) δ 11.22 (s, 1 H), 8.49 (s, 1 H), 7.61 (d, J = 7.7 Hz, 1 H), 7.43–7.40 (m, 2 H), 7.34 (td, J = 7.7, 1.2 Hz, 1 H), 7.19 (td, J = 7.7, 1.7 Hz, 1 H), 3.94 (s, 2 H), 3.93 (s, 3 H), 3.87 (s, 3 H), 3.85 (s, 3 H); $^{13}C\{^{1}H\}$ NMR (75 MHz, $CDCl_3$) δ 168.7, 168.1, 153.9, 143.9, 137.5, 134.3, 133.0, 132.0, 129.1, 127.8, 125.3, 112.0, 106.7, 103.4, 56.2, 56.1, 52.1, 45.6; IR: 3233, 3139,

3007, 2965, 2831, 1692, 1594, 1357, 1215, 1161, 879, 579 cm^{-1}; HRMS (ESI) *m*/*z*: $[M + H]^+$ Calcd for $C_{18}H_{19}BrNO_5$ 408.0441; Found 408.0437; new compound.

*Methyl 2-[2-(2-Bromo-4-fluorophenyl)acetamido]benzoate (**4o**).* Yield: 648.1 mg, 88%; orange solids; mp 105.3–106.2 °C; ^1H NMR (400 MHz, $CDCl_3$) δ 11.07 (s, 1 H), 8.71 (dd, J = 8.5, 1.2 Hz, 1 H), 8.00 (dd, J = 8.0, 1.7 Hz, 1 H), 7.53 (ddd, J = 8.6, 7.4, 1.7 Hz, 1 H), 7.43–7.33 (m, 2 H), 7.11–7.04 (m, 2 H), 3.90 (s, 2 H), 3.86 (s, 3 H); $^{13}C\{^1H\}$ NMR (100 MHz, $CDCl_3$) δ 168.5, 168.4, 161.8 (d, J = 250.5 Hz), 141.2, 134.6, 132.7 (d, J = 8.5 Hz), 130.8, 130.3 (d, J = 3.5 Hz), 125.3 (d, J = 9.4 Hz), 122.7, 120.4, 120.1, 115.2, 115.0 (d, J = 21.0 Hz), 52.3, 44.8; IR: 3320, 3093, 2954, 2848, 1741, 1682, 1488, 1255, 1166, 860, 741, 465 cm^{-1}; HRMS (ESI) *m*/*z*: $[M + H]^+$ Calcd for $C_{16}H_{14}BrFNO_3$ 366.0136; Found 366.0129; new compound.

*Methyl 2-[2-(2-Bromo-4-chlorophenyl)acetamido]benzoate (**4p**).* Yield: 637.2 mg, 83%; white solids; mp 87.4–88.4 °C; ^1H NMR (400 MHz, $CDCl_3$) δ 11.10 (s, 1 H), 8.69 (d, J = 8.5 Hz, 1 H), 8.00 (dd, J = 8.0, 1.6 Hz, 1 H), 7.63 (d, J = 1.9 Hz, 1 H), 7.55–7.50 (m, 1 H), 7.38–7.31 (m, 2 H), 7.11–7.06 (m, 1 H), 3.90 (s, 2 H), 3.87 (s, 3 H); $^{13}C\{^1H\}$ NMR (75 MHz, $CDCl_3$) δ 168.5, 168.2, 141.2, 134.6, 134.1, 132.9, 132.62, 132.57, 130.8, 128.0, 125.6, 122.8, 120.4, 115.2, 52.3, 45.0; IR: 3243, 3118, 2950, 2897, 1695, 1678, 1589, 1515, 1270, 1089, 760, 524 cm^{-1}; HRMS (ESI) *m*/*z*: $[M + H]^+$ Calcd for $C_{16}H_{14}BrClNO_3$ 381.9840; Found 381.9835; new compound.

*Methyl 2-[2-(2,4-Dibromophenyl)acetamido]benzoate (**4q**).* Yield: 731.2 mg, 86%; white solids; mp 95.7–96.5 °C; ^1H NMR (400 MHz, $CDCl_3$) δ 11.13 (s, 1 H), 8.70 (d, J = 8.0 Hz, 1 H), 8.00 (dd, J = 8.0, 1.1 Hz, 1 H), 7.57 (d, J = 2.2 Hz, 1 H), 7.53 (t, J = 8.0 Hz, 1 H), 7.47 (d, J = 8.5 Hz, 1 H), 7.32 (dd, J = 8.5, 2.2 Hz, 1 H), 7.09 (t, J = 8.0 Hz, 1 H), 3.90 (s, 2 H), 3.88 (s, 3 H); $^{13}C\{^1H\}$ NMR (75 MHz, $CDCl_3$) δ 168.5, 167.9, 141.1, 136.3, 134.7, 134.6, 134.3, 132.1, 130.8, 124.0, 122.8, 121.6, 120.4, 115.3, 52.3, 45.3; IR: 3403, 3302, 3116, 2952, 1686, 1589, 1448, 1259, 1085, 756, 703, 475 cm^{-1}; HRMS (ESI) *m*/*z*: $[M + H]^+$ Calcd for $C_{16}H_{14}Br_2NO_3$ 425.9335; Found 425.9330; new compound.

*Methyl 2-[2-(2-Bromo-4-nitrophenyl)acetamido]benzoate (**4r**).* Yield: 637.9 mg, 81%; white solids; mp

110.0–110.9 °C; ^{1}H NMR (300 MHz, $CDCl_3$) δ 11.23 (s, 1 H), 8.67 (dd, J = 8.5, 1.2 Hz, 1 H), 8.49 (d, J = 2.0 Hz, 1 H), 8.20 (dd, J = 8.4, 2.3 Hz, 1 H), 8.02 (dd, J = 8.2, 2.0 Hz, 1 H), 7.62 (d, J = 8.2 Hz, 1 H), 7.57–7.51 (m, 1 H), 7.14–7.08 (m, 1 H), 4.04 (s, 2 H), 3.88 (s, 3 H); $^{13}C\{^1H\}$ NMR (75 MHz, $CDCl_3$) δ 168.6, 166.9, 147.6, 141.7, 141.0, 134.8, 132.4, 130.9, 128.0, 125.6, 123.0, 122.6, 120.4, 115.1, 52.4, 45.4; IR: 3401, 3278, 3101, 3033, 2952, 1705, 1671, 1520, 1343, 1261, 1080, 600 cm^{-1}; HRMS (ESI) *m/z*: [M + H]$^+$ Calcd for $C_{16}H_{14}BrN_2O_5$ 393.0081; Found 393.0074; new compound.

*Methyl 2-[2-(2-Bromo-5-fluorophenyl)acetamido]benzoate (**4s**).* Yield: 623.8 mg, 85%; dark brown solids; mp 115.7–116.5 °C; ^{1}H NMR (400 MHz, $CDCl_3$) δ 11.12 (s, 1 H), 8.71 (dd, J = 8.7, 0.9 Hz, 1 H), 8.00 (dd, J = 8.0, 1.7 Hz, 1 H), 7.62–7.49 (m, 2 H), 7.18 (dd, J = 9.0, 3.0 Hz, 1 H), 7.12–7.05 (m, 1 H), 6.99–6.87 (m, 1 H), 3.91 (s, 2 H), 3.87 (s, 3 H); $^{13}C\{^1H\}$ NMR (75 MHz, $CDCl_3$) δ 168.5, 168.0, 162.0 (d, J = 247.6 Hz), 141.1, 136.2 (d, J = 7.8 Hz), 134.6, 134.1 (d, J = 8.1 Hz), 130.8, 122.8, 120.4, 119.4 (d, J = 3.4 Hz), 118.9 (d, J = 23.1 Hz), 116.3 (d, J = 22.4 Hz), 115.2, 52.3, 45.6; IR: 3368, 3255, 3115, 2956, 1684, 1591, 1451, 1262, 1152, 881, 754, 482 cm^{-1}; HRMS (ESI) *m/z*: [M + H]$^+$ Calcd for $C_{16}H_{14}BrFNO_3$ 366.0136; Found 366.0133; new compound.

*Methyl 2-[2-(2-Bromo-5-chlorophenyl)acetamido]benzoate (**4t**).* Yield: 721.4 mg, 94%; white solids; mp 113.4–114.2 °C; ^{1}H NMR (400 MHz, $CDCl_3$) δ 11.12 (s, 1 H), 8.70 (dd, J = 8.5, 1.2 Hz, 1 H), 8.00 (dd, J = 8.0, 1.7 Hz, 1 H), 7.57–7.50 (m, 2 H), 7.42 (d, J = 2.5 Hz, 1 H), 7.17 (dd, J = 8.5, 2.5 Hz, 1 H), 7.11–7.05 (m, 1 H), 3.90 (s, 2 H), 3.88 (s, 3 H); $^{13}C\{^1H\}$ NMR (75 MHz, $CDCl_3$) δ 168.5, 167.9, 141.1, 136.0, 134.6, 134.0, 133.7, 131.8, 130.8, 129.2, 123.2, 122.8, 120.4, 115.2, 52.3, 45.4; IR: 3363, 3250, 3196, 2984, 1675, 1593, 1450, 1257, 1166, 1026, 757, 454 cm^{-1}; HRMS (ESI) *m/z*: [M + H]$^+$ Calcd for $C_{16}H_{14}BrClNO_3$ 381.9840; Found 381.9837; new compound.

*Methyl 2-[2-(2-Bromo-4,5-dimethoxyphenyl)acetamido]benzoate (**4u**).* Yield: 729.9 mg, 89%; brown solids; mp 129.9–130.7 °C; ^{1}H NMR (400 MHz, $CDCl_3$) δ 11.00 (s, 1 H), 8.71 (dd, J = 8.5, 1.2 Hz, 1 H), 7.98 (dd, J = 8.0, 1.7 Hz, 1 H), 7.52 (ddd, J = 8.5, 7.2, 1.7 Hz, 1 H), 7.09–7.04 (m, 2 H), 6.94 (s, 1 H), 3.91 (s, 3 H), 3.88 (s, 3 H), 3.86 (s, 2 H), 3.85 (s, 3 H); $^{13}C\{^1H\}$ NMR (75 MHz, $CDCl_3$) δ 169.0, 168.3,

149.0, 148.7, 141.2, 134.5, 130.7, 126.1, 122.7, 120.5 (2 × C), 115.7, 115.3, 114.3, 56.2, 56.1, 52.2, 45.3; IR: 3253, 3125, 3007, 2954, 2846, 1686, 1503, 1439, 1263, 1166, 760, 588 cm^{-1}; HRMS (ESI) *m/z*: [M + H]$^+$ Calcd for $C_{18}H_{19}BrNO_5$ 408.0441; Found 408.0434; new compound.

*Methyl 2-[2-(6-Bromobenzo[d][1,3]dioxol-5-yl)acetamido]benzoate (**4v**).* Yield: 642.1 mg, 82%; brown solids; mp 127.3–128.1 °C; ^{1}H NMR (300 MHz, $CDCl_3$) δ 11.02 (s, 1 H), 8.71 (dd, *J* = 8.5, 1.0 Hz, 1 H), 7.99 (dd, *J* = 8.0, 1.6 Hz, 1 H), 7.55–7.49 (m, 1 H), 7.11–7.03 (m, 2 H), 6.90 (s, 1 H), 5.99 (s, 2 H), 3.87 (s, 3 H), 3.84 (s, 2 H); ^{13}C{^{1}H} NMR (75 MHz, $CDCl_3$) δ 168.9, 168.3, 147.9, 147.7, 141.2, 134.5, 130.7, 127.1, 122.7, 120.5, 115.8, 115.3, 112.9, 111.3, 101.8, 52.2, 45.4; IR: 3300, 3268, 3122, 2921, 1689, 1589, 1483, 1326, 1260, 1026, 760, 632 cm^{-1}; HRMS (ESI) *m/z*: [M + H]$^+$ Calcd for $C_{17}H_{15}BrNO_5$ 392.0128; Found 392.0123; new compound.

*Methyl 2-[2-(2-Bromo-4-fluorophenyl)acetamido]-4-fluorobenzoate (**4w**).* Yield: 639.8 mg, 83%; white solids; mp 88.9–89.6 °C; ^{1}H NMR (300 MHz, $CDCl_3$) δ 11.28 (s, 1 H), 8.54 (dd, *J* = 11.9, 2.6 Hz, 1 H), 8.02 (dd, *J* = 9.0, 6.5 Hz, 1 H), 7.56 (dd, *J* = 8.8, 5.3 Hz, 1 H), 7.16 (dd, *J* = 8.9, 3.0 Hz, 1 H), 6.98–6.89 (m, 1 H), 6.85–6.71 (m, 1 H), 3.91 (s, 2 H), 3.87 (s, 3 H); ^{13}C{^{1}H} NMR (75 MHz, $CDCl_3$) δ 168.2, 167.8, 166.2 (d, *J* = 253.7 Hz), 162.0 (d, *J* = 247.8 Hz), 143.3 (d, *J* = 13.0 Hz), 135.9 (d, *J* = 8.0 Hz), 134.2 (d, *J* = 8.4 Hz), 133.1 (d, *J* = 10.6 Hz), 119.4 (d, *J* = 3.4 Hz), 119.0 (d, *J* = 23.1 Hz), 116.4 (d, *J* = 22.3 Hz), 111.3 (d, *J* = 2.9 Hz), 110.1 (d, *J* = 22.4 Hz), 107.6 (d, *J* = 28.5 Hz), 52.3, 45.5; IR: 3371, 3248, 3192, 3121, 2961, 1682, 1597, 1436, 1255, 1198, 786, 565 cm^{-1}; HRMS (ESI) *m/z*: [M + H]$^+$ Calcd for $C_{16}H_{13}BrF_2NO_3$ 384.0041; Found 384.0032; new compound.

*Methyl 2-[2-(2-Bromo-5-fluorophenyl)acetamido]-4-fluorobenzoate (**4x**).* Yield: 654.1 mg, 85%; white solids; mp 102.2–102.7 °C; ^{1}H NMR (300 MHz, $CDCl_3$) δ 11.23 (s, 1 H), 8.54 (dd, *J* = 11.9, 2.6 Hz, 1 H), 8.01 (dd, *J* = 8.9, 6.5 Hz, 1 H), 7.42–7.35 (m, 2 H), 7.08 (td, *J* = 8.3, 2.7 Hz, 1 H), 6.80–6.73 (m, 1 H), 3.91 (s, 2 H), 3.86 (s, 3 H); ^{13}C{^{1}H} NMR (75 MHz, $CDCl_3$) δ 168.7, 167.8, 166.2 (d, *J* = 253.6 Hz), 161.9 (d, *J* = 250.7 Hz), 143.4 (d, *J* = 12.9 Hz), 133.1 (d, *J* = 10.7 Hz), 132.8 (d, *J* = 8.6 Hz), 130.0 (d, *J* = 3.7 Hz), 125.3 (d, *J* = 9.9 Hz), 120.3 (d, *J* = 24.6 Hz), 115.0 (d, *J* = 21.0 Hz), 111.3 (d, *J* = 2.8 Hz),

110.1 (d, J = 22.5 Hz), 107.6 (d, J = 28.6 Hz), 52.3, 44.8; IR: 3377, 3250, 3113, 2960, 1689, 1599, 1486, 1260, 1172, 879, 784, 593 cm^{-1}; HRMS (ESI) *m/z*: $[M + H]^+$ Calcd for $C_{16}H_{13}BrF_2NO_3$ 384.0041; Found 384.0034; new compound.

*Methyl 2-[2-(2-Bromo-5-chlorophenyl)acetamido]-4-fluorobenzoate (**4y**).* Yield: 723.6 mg, 90%; white solids; mp 116.5–117.5 °C; ^{1}H NMR (300 MHz, $CDCl_3$) δ 11.29 (s, 1 H), 8.53 (dd, J = 11.9, 2.6 Hz, 1 H), 8.02 (dd, J = 9.0, 6.5 Hz, 1 H), 7.53 (d, J = 8.6 Hz, 1 H), 7.41 (d, J = 2.5 Hz, 1 H), 7.19 (dd, J = 8.6, 2.5 Hz, 1 H), 6.82–6.74 (m, 1 H), 3.90 (s, 2 H), 3.87 (s, 3 H); ^{13}C{^{1}H} NMR (75 MHz, $CDCl_3$) δ 168.1, 167.8, 166.2 (d, J = 253.5 Hz), 143.3 (d, J = 13.1 Hz), 135.6, 134.0, 133.8, 133.1 (d, J = 10.7 Hz), 131.8, 129.3, 123.1, 111.4, 110.1 (d, J = 22.4 Hz), 107.7 (d, J = 28.5 Hz), 52.4, 45.3; IR: 3305, 3246, 3194, 2961, 1684, 1531, 1262, 1123, 845, 783, 694, 533 cm^{-1}; HRMS (ESI) *m/z*: $[M + H]^+$ Calcd for $C_{16}H_{13}BrClFNO_3$ 399.9746; Found 399.9742; new compound.

*Methyl 2-[2-(2-Bromo-5-chlorophenyl)acetamido]-4-chlorobenzoate (**4z**).* Yield: 684.8 mg, 82%; brown solids; mp 103.0–103.5 °C; ^{1}H NMR (300 MHz, $CDCl_3$) δ 11.18 (s, 1 H), 8.82 (d, J = 2.1 Hz, 1 H), 7.93 (d, J = 8.6 Hz, 1 H), 7.53 (d, J = 8.5 Hz, 1 H), 7.41 (d, J = 2.5 Hz, 1 H), 7.18 (dd, J = 8.5, 2.5 Hz, 1 H), 7.06 (dd, J = 8.6, 2.1 Hz, 1 H), 3.90 (s, 2 H), 3.88 (s, 3 H); ^{13}C{^{1}H} NMR (75 MHz, $CDCl_3$) δ 168.1, 167.9, 142.0, 141.0, 135.6, 134.0, 133.8, 131.84, 131.80, 129.3, 123.1, 120.4 (2 × C), 113.4, 52.5, 45.3; IR: 3505, 3381, 3236, 2950, 2843, 1678, 1578, 1405, 1247, 1100, 784, 457 cm^{-1}; HRMS (ESI) *m/z*: $[M + H]^+$ Calcd for $C_{16}H_{13}BrCl_2NO_3$ 415.9450; Found 415.9445; new compound.

*Methyl 2-[2-(2-Bromo-4,5-dimethoxyphenyl)acetamido]-4-chlorobenzoate (**4A**).* Yield: 818.3 mg, 92%; brown solids; mp 133.8–134.5 °C; ^{1}H NMR (300 MHz, $CDCl_3$) δ 11.07 (s, 1 H), 8.83 (d, J = 2.1 Hz, 1 H), 7.91 (d, J = 8.6 Hz, 1 H), 7.07 (s, 1 H), 7.04 (dd, J = 8.6, 2.1 Hz, 1 H), 6.92 (s, 1 H), 3.91 (s, 3 H), 3.88 (s, 3 H), 3.86 (s, 2 H), 3.85 (s, 3 H); ^{13}C{^{1}H} NMR (75 MHz, $CDCl_3$) δ 169.2, 167.7, 149.1, 148.7, 142.1, 140.9, 131.8, 125.7, 122.9, 120.4, 115.7, 115.3, 114.2, 113.4, 56.2, 56.1, 52.4, 45.3; IR: 3474, 3358, 3315, 3007, 2952, 1684, 1507, 1246, 1100, 906, 774, 547 cm^{-1}; HRMS (ESI) *m/z*: $[M + H]^+$ Calcd for $C_{18}H_{18}BrClNO_5$ 442.0051; Found 442.0043; new compound.

*Methyl 2-[2-(6-Bromobenzo[d][1,3]dioxol-5-yl)acetamido]-4-chlorobenzoate (**4B**).* Yield: 836.7 mg, 98%; brown solids; mp 141.8–142.3 °C; ^{1}H NMR (300 MHz, $CDCl_3$) δ 11.09 (s, 1 H), 8.83 (d, J = 2.1 Hz, 1 H), 7.92 (d, J = 8.6 Hz, 1 H), 7.26 (s, 1 H), 7.06–7.02 (m, 2 H), 6.88 (s, 1 H), 5.99 (s, 2 H), 3.87 (s, 3 H), 3.83 (s, 2 H); $^{13}C\{^1H\}$ NMR (75 MHz, $CDCl_3$) δ 169.0, 167.8, 148.0, 147.7, 142.1, 140.9, 131.8, 126.7, 122.9, 120.4, 115.8, 113.5, 113.0, 111.3, 101.9, 52.4, 45.3; IR: 3297, 3114, 2949, 2893, 1682, 1580, 1406, 1251, 1103, 926, 775, 473 cm^{-1}; HRMS (ESI) *m*/*z*: $[M + H]^+$ Calcd for $C_{17}H_{14}BrClNO_5$ 425.9738; Found 425.9734; new compound.

*Methyl 5-Bromo-2-[2-(2,4-dibromophenyl)acetamido]benzoate (**4C**).* Yield: 932.2 mg, 92%; white solids; mp 158.0–158.9 °C; ^{1}H NMR (400 MHz, $CDCl_3$) δ 11.02 (s, 1 H), 8.63 (d, J = 9.0 Hz, 1 H), 8.12 (d, J = 2.4 Hz, 1 H), 7.62 (dd, J = 9.0, 2.4 Hz, 1 H), 7.55 (d, J = 2.4 Hz, 1 H), 7.47 (d, J = 8.5 Hz, 1 H), 7.32 (dd, J = 8.5, 2.4 Hz, 1 H), 3.89 (s, 3 H), 3.88 (s, 2 H); $^{13}C\{^1H\}$ NMR (75 MHz, $CDCl_3$) δ 167.9, 167.3, 140.1, 137.3, 136.0, 134.7, 134.3, 133.3, 132.2, 123.9, 122.1, 121.6, 116.8, 115.2, 52.6, 45.3; IR: 3378, 3297, 3125, 3063, 2952, 1687, 1510, 1251, 1026, 788, 713, 521 cm^{-1}; HRMS (ESI) *m*/*z*: $[M + H]^+$ Calcd for $C_{16}H_{13}Br_3NO_3$ 503.8440; Found 503.8438; new compound.

*Methyl 2-[2-(2-Bromo-5-chlorophenyl)acetamido]-5-methoxybenzoate (**4D**).* Yield: 819.8 mg, 99%; white solids; mp 130.8–131.5 °C; ^{1}H NMR (300 MHz, $CDCl_3$) δ 10.82 (s, 1 H), 8.61 (d, J = 9.2 Hz, 1 H), 7.52 (d, J = 8.5 Hz, 1 H), 7.48 (d, J = 3.1 Hz, 1 H), 7.42 (d, J = 2.6 Hz, 1 H), 7.17 (dd, J = 8.5, 2.6 Hz, 1 H), 7.10 (dd, J = 9.2, 3.1 Hz, 1 H), 3.88 (s, 2 H), 3.87 (s, 3 H), 3.80 (s, 3 H); $^{13}C\{^1H\}$ NMR (75 MHz, $CDCl_3$) δ 168.1, 167.4, 154.7, 136.1, 134.7, 133.9, 133.7, 131.8, 129.1, 123.1, 122.0, 120.8, 116.4, 114.6, 55.6, 52.4, 45.2; IR: 3313, 3277, 3073, 2956, 2835, 1697, 1530, 1224, 1084, 832, 768, 454 cm^{-1}; HRMS (ESI) *m*/*z*: $[M + H]^+$ Calcd for $C_{17}H_{16}BrClNO_4$ 411.9946; Found 411.9940; new compound.

*Methyl 2-[2-(6-Bromobenzo[d][1,3]dioxol-5-yl)acetamido]-4,5-dimethoxybenzoate (**4E**).* Yield: 751.1 mg, 83%; gray solids; mp 143.1–143.9 °C; ^{1}H NMR (400 MHz, $CDCl_3$) δ 11.20 (s, 1 H), 8.48 (s, 1 H), 7.43 (s, 1 H), 7.05 (s, 1 H), 6.88 (s, 1 H), 5.98 (s, 2 H), 3.93 (s, 3 H), 3.87 (s, 3 H), 3.87 (s, 3 H), 3.83 (s, 2 H); $^{13}C\{^1H\}$ NMR (75 MHz, $CDCl_3$) δ 168.8, 168.1, 153.8, 147.9, 147.6, 143.9, 137.4, 127.1, 115.7,

112.9, 111.9, 111.4, 106.7, 103.4, 101.8, 56.12, 56.05, 52.03, 45.3; IR: 3300, 3260, 2999, 2950, 2831, 1677, 1531, 1358, 1215, 1127, 781, 506 cm^{-1}; HRMS (ESI) *m*/*z*: $[M + H]^+$ Calcd for $C_{19}H_{19}BrNO_7$ 452.0339; Found 452.0344; new compound.

Benzofuro[3,2-*c*]quinolin-6(5*H*)-one (1a)

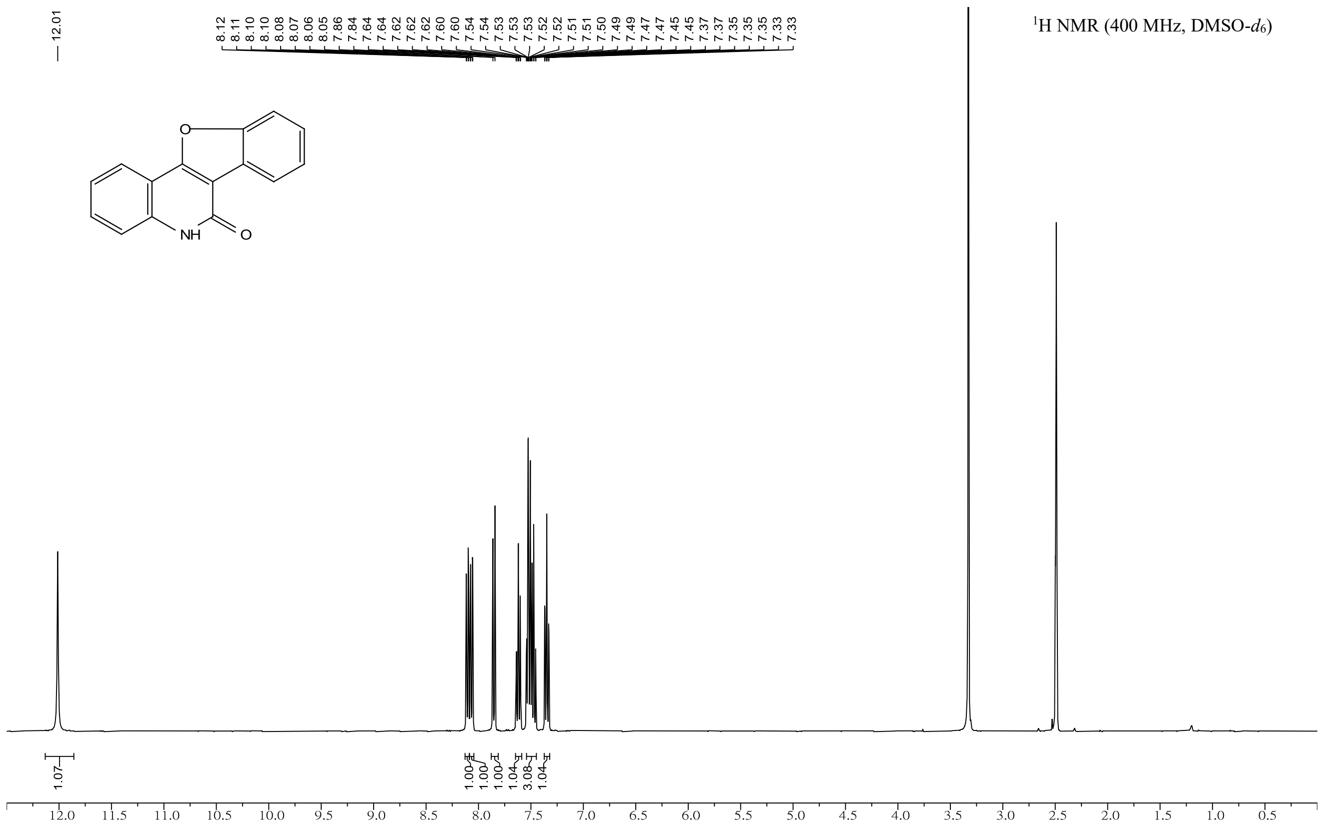

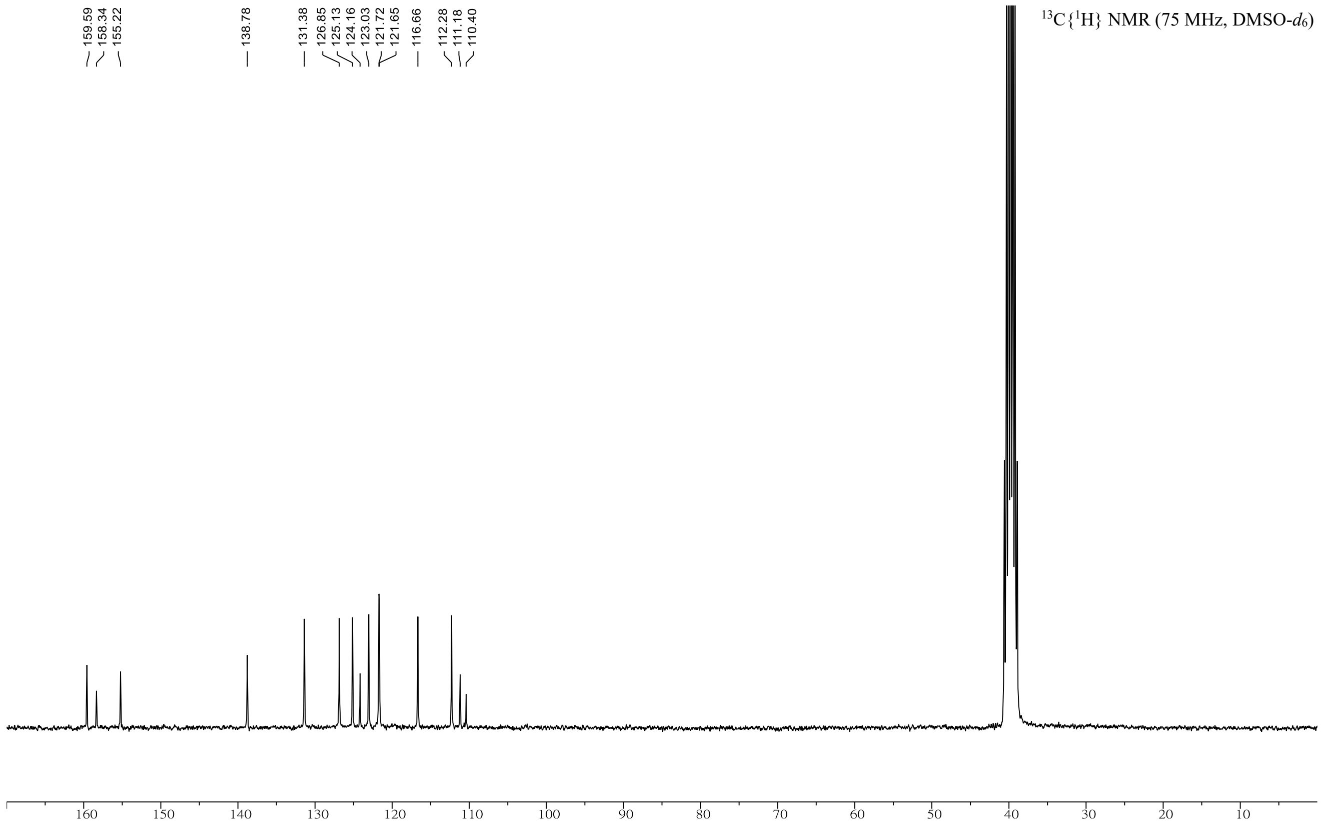

2-Fluorobenzofuro[3,2-*c*]quinolin-6(5*H*)-one (1b)

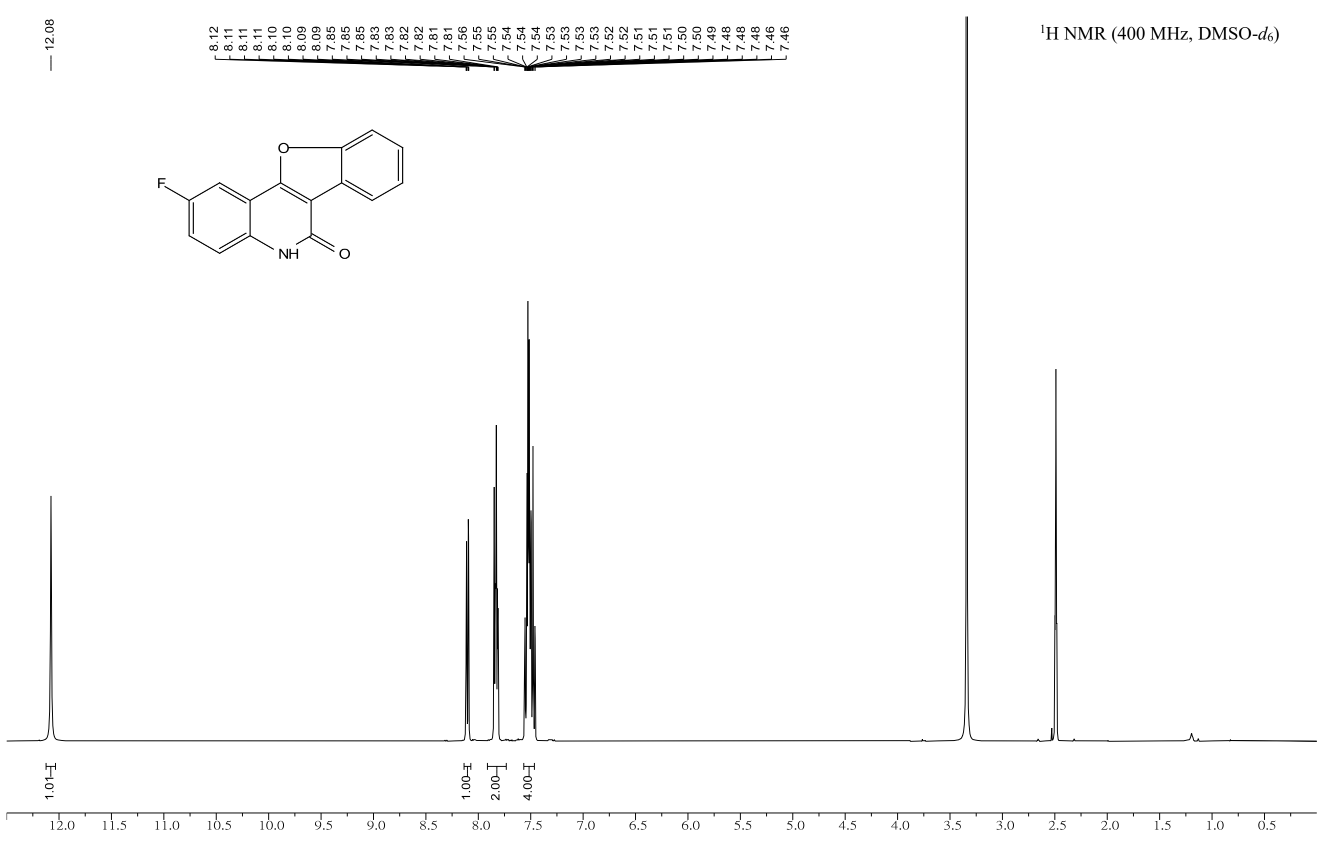

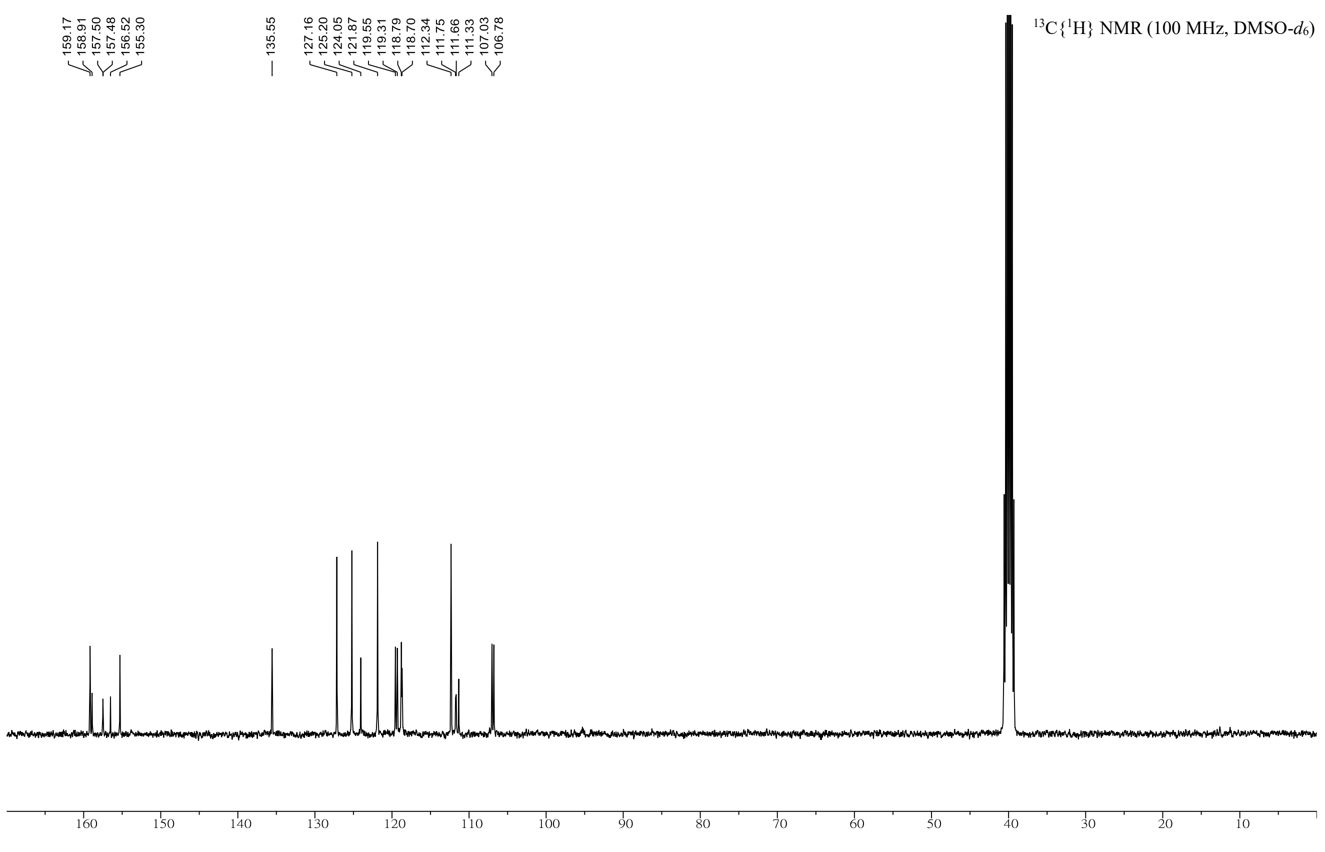

2-Chlorobenzofuro[3,2-*c*]quinolin-6(5*H*)-one (1c)

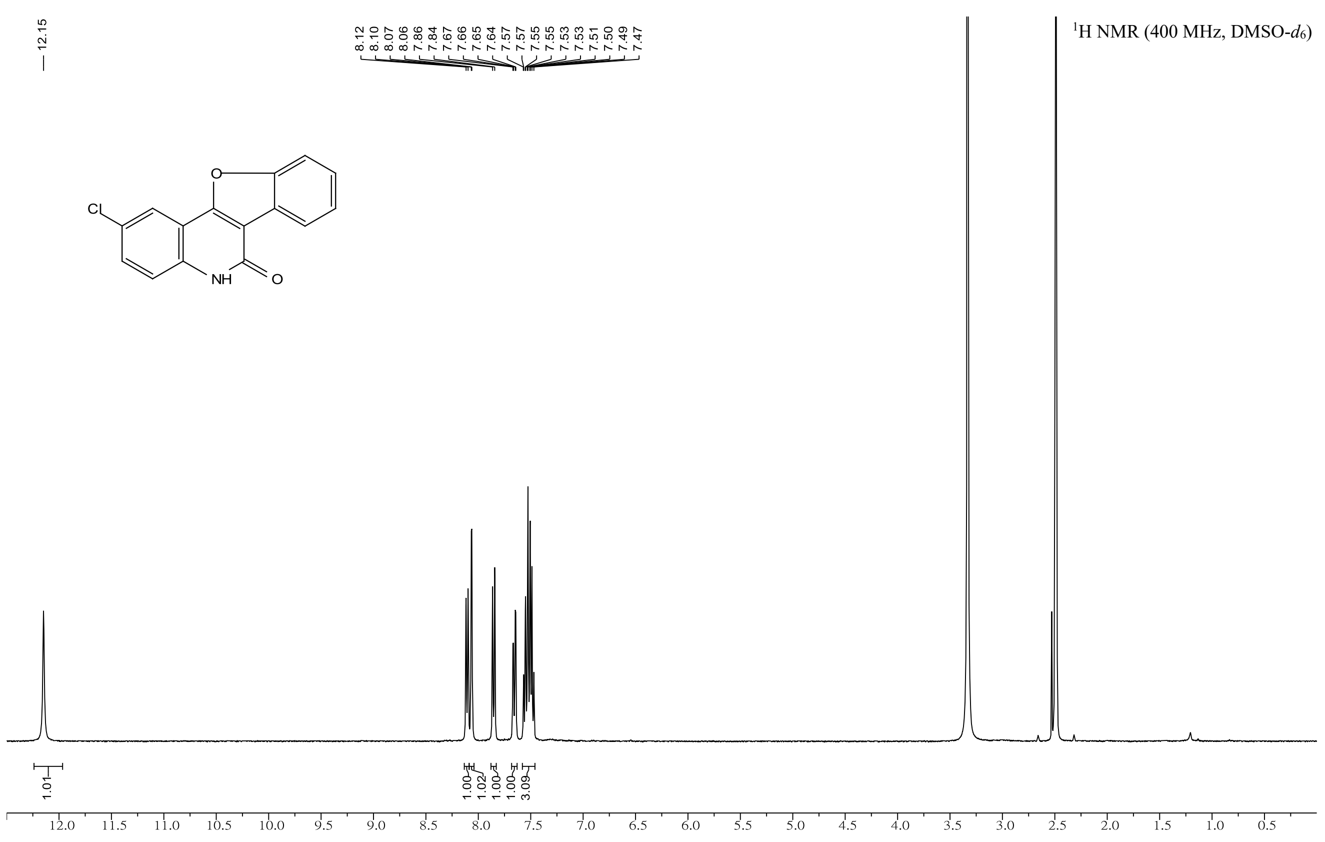

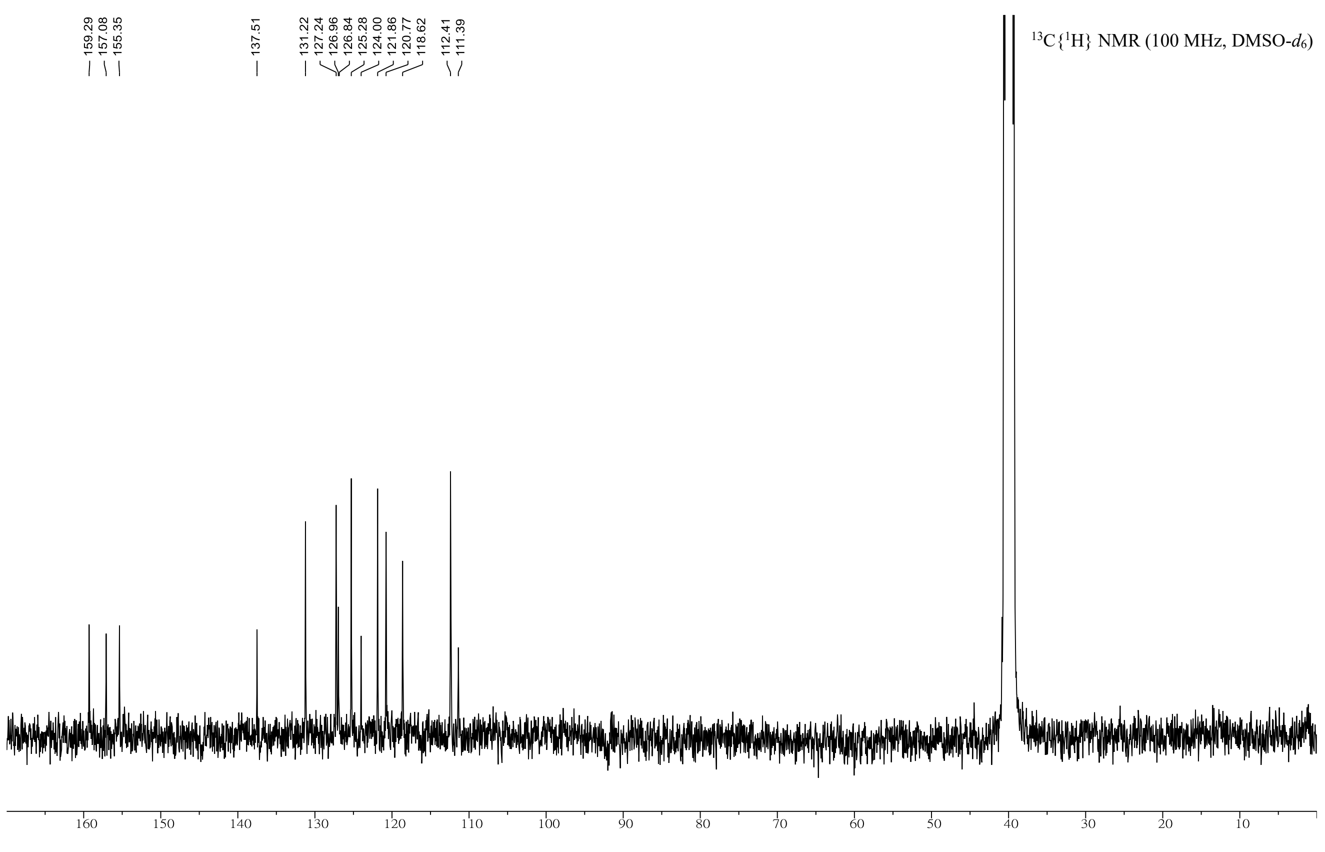

2-Bromobenzofuro[3,2-*c*]quinolin-6(5*H*)-one (1d)

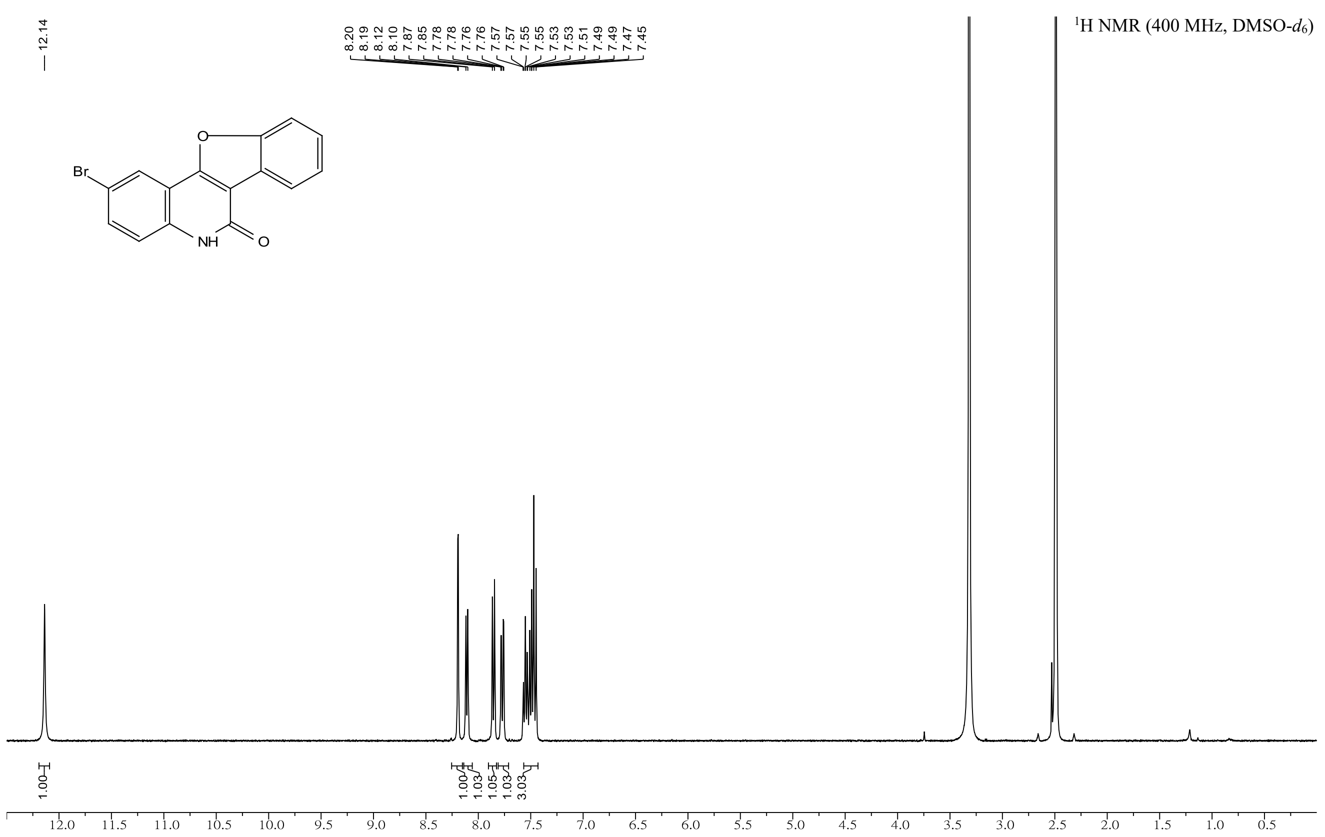

^{1}H NMR (400 MHz, DMSO-d_6)

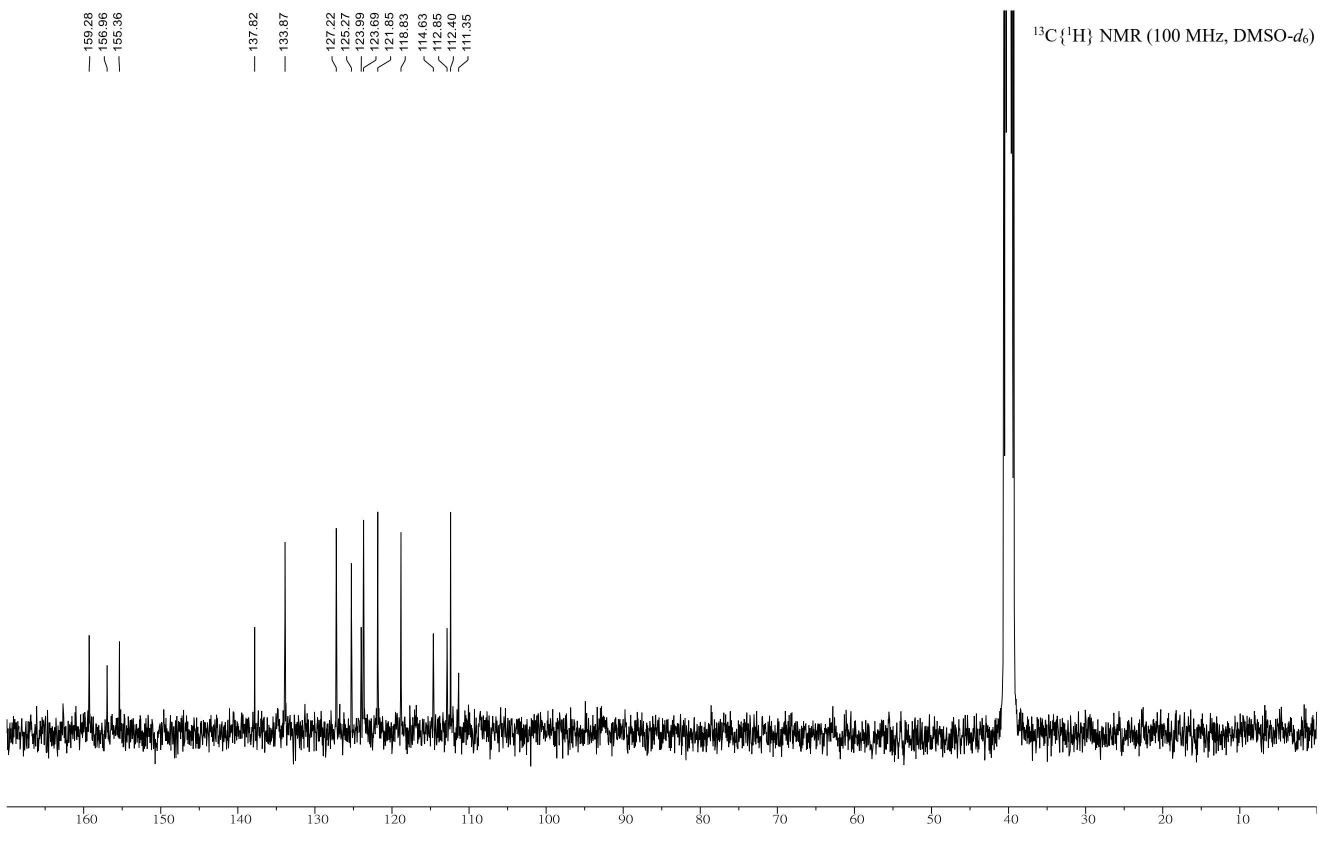

^{13}C{^{1}H} NMR (100 MHz, DMSO-d_6)

2-Iodobenzofuro[3,2-*c*]quinolin-6(5*H*)-one (1e)

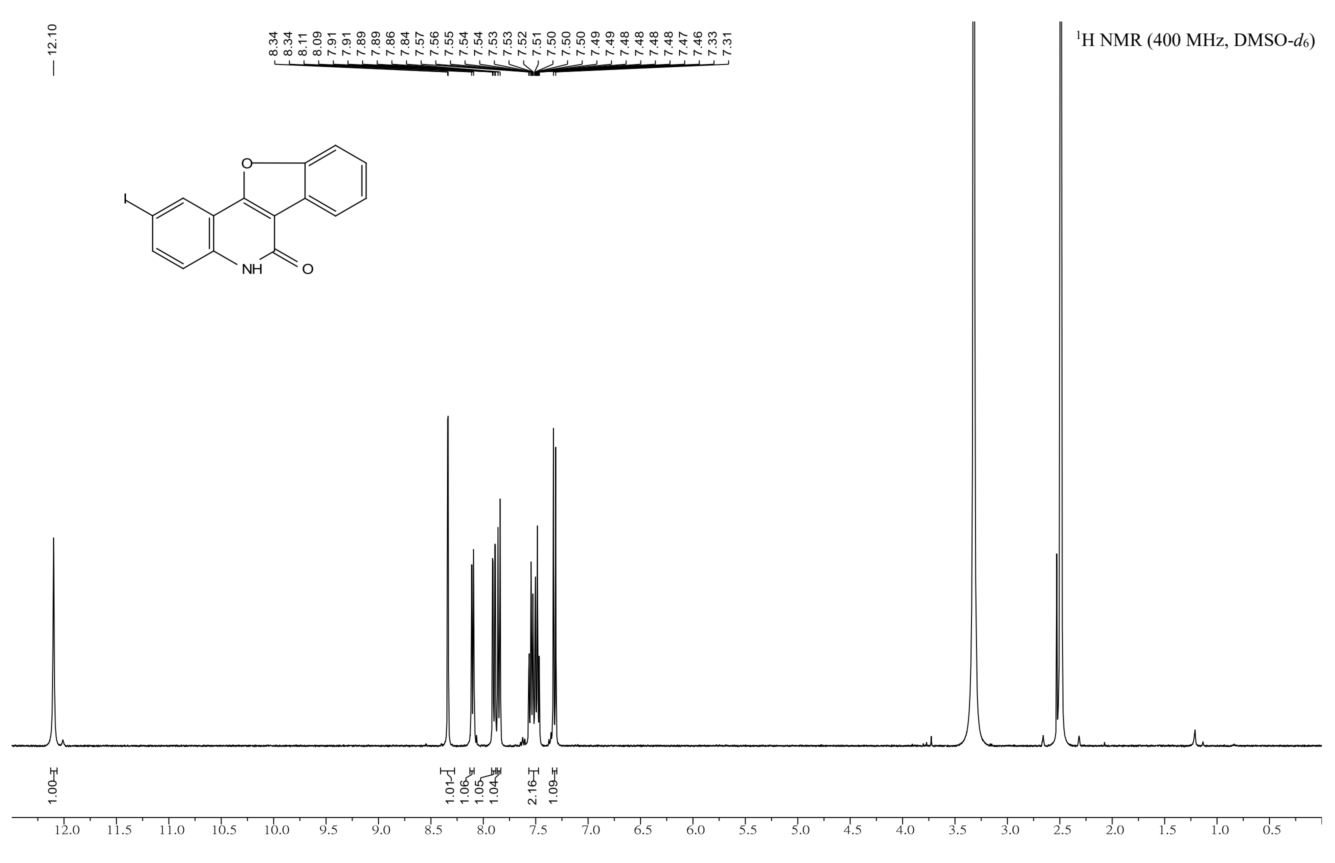

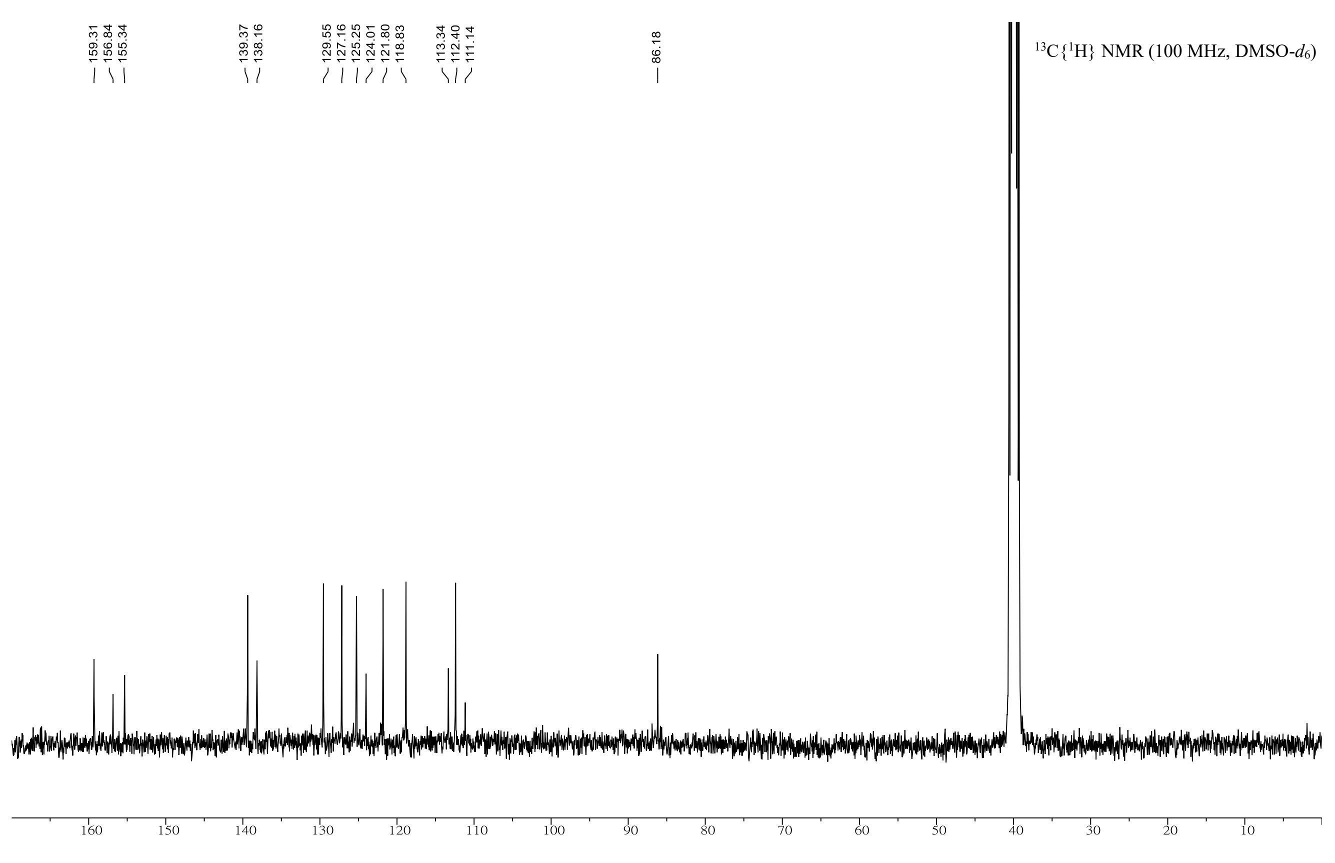

2-Nitrobenzofuro[3,2-*c*]quinolin-6(5*H*)-one (1f)

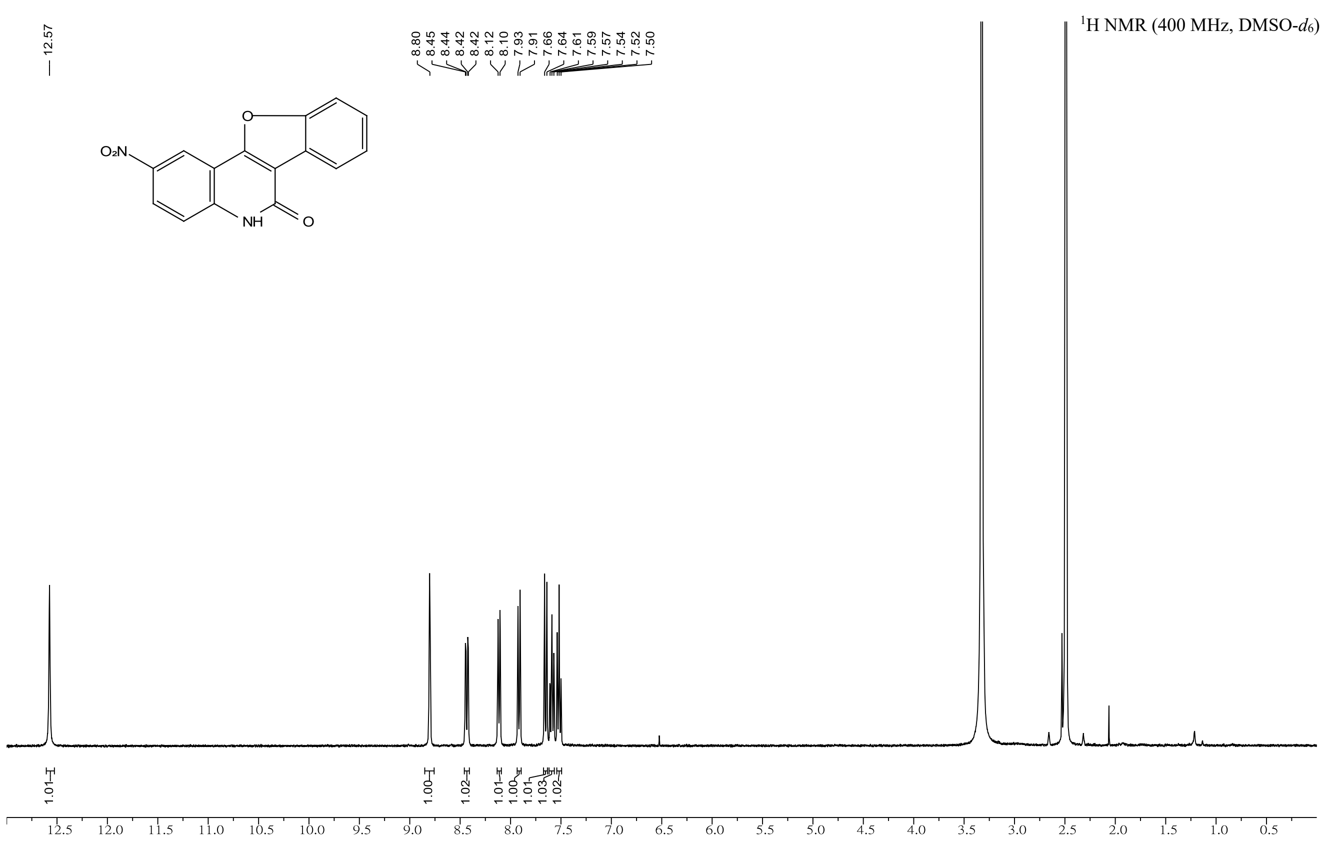

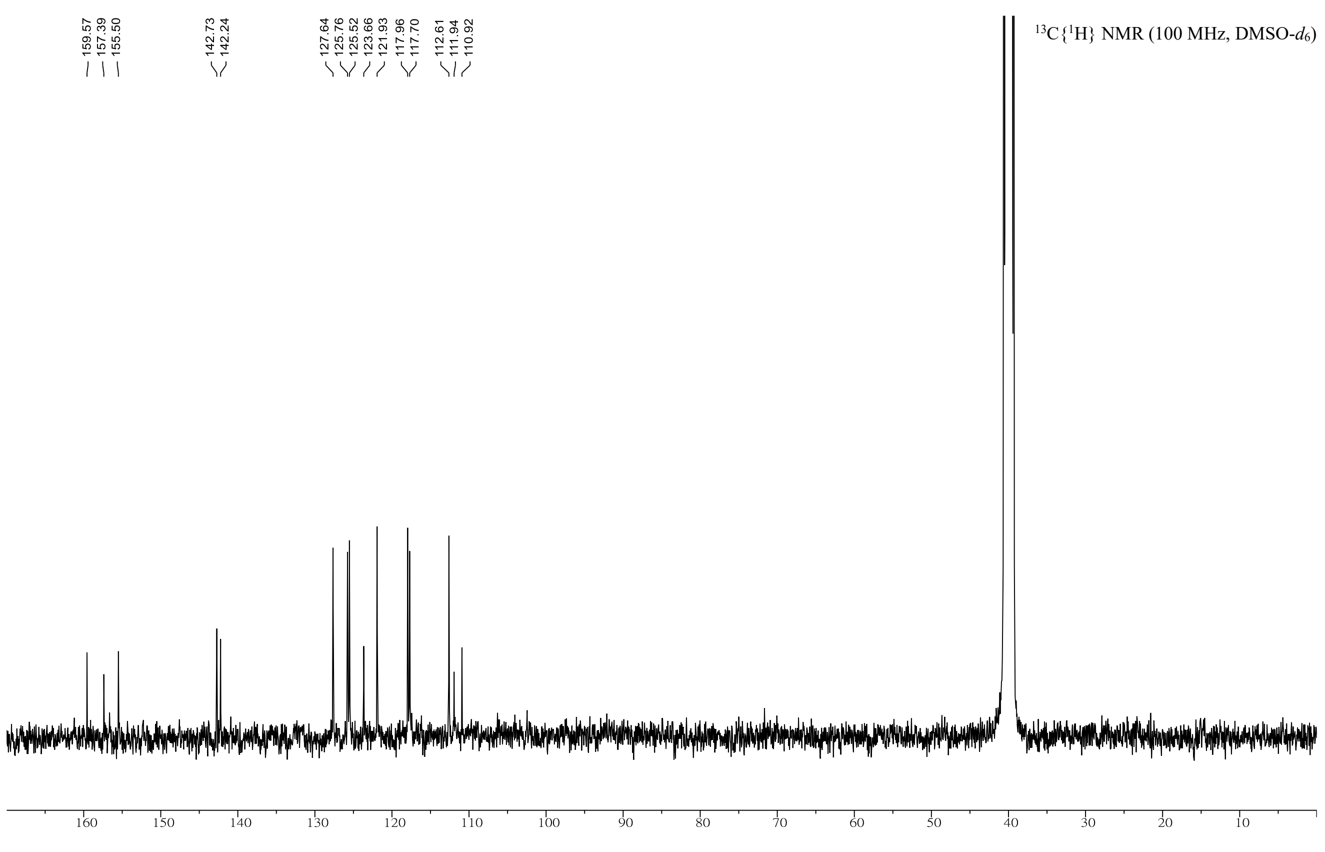

2-Methoxybenzofuro[3,2-*c*]quinolin-6(5*H*)-one (1g)

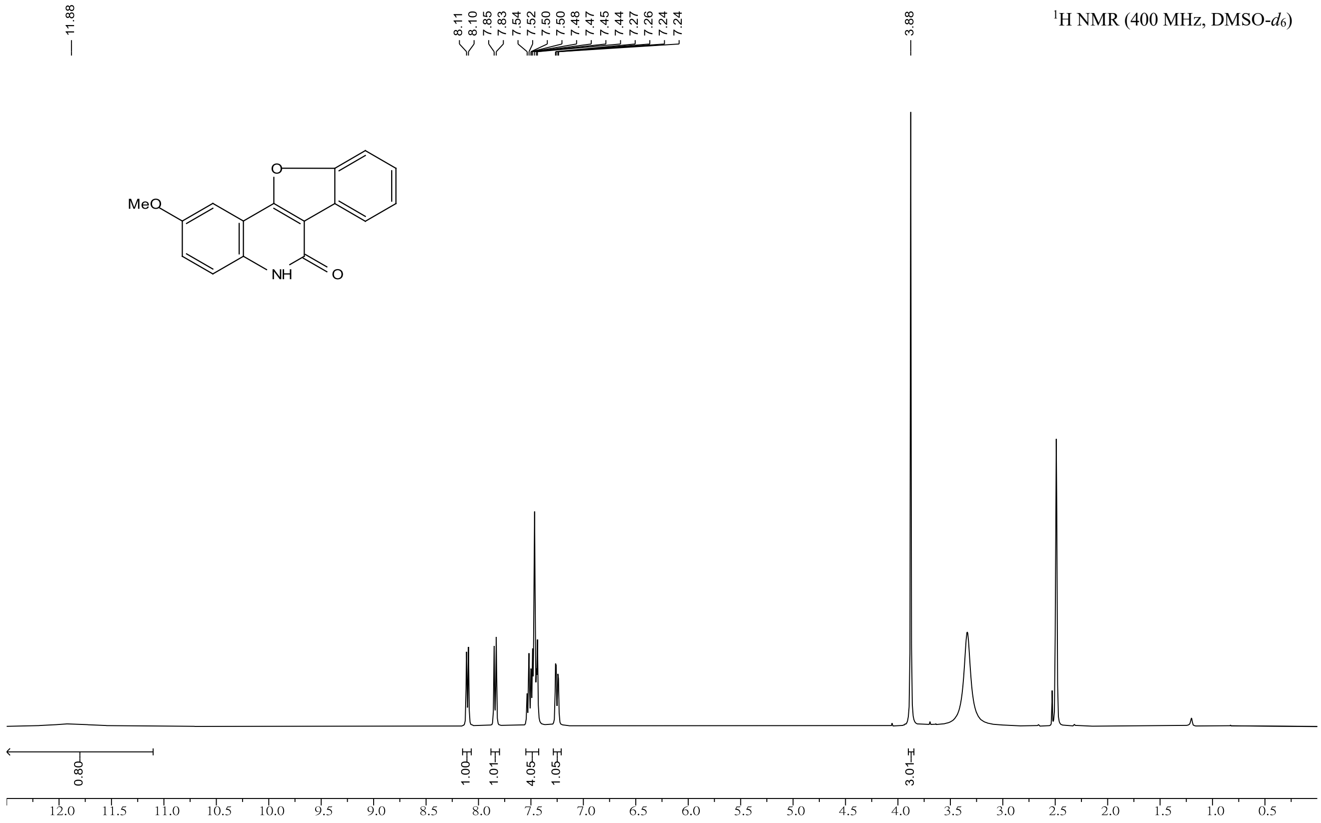

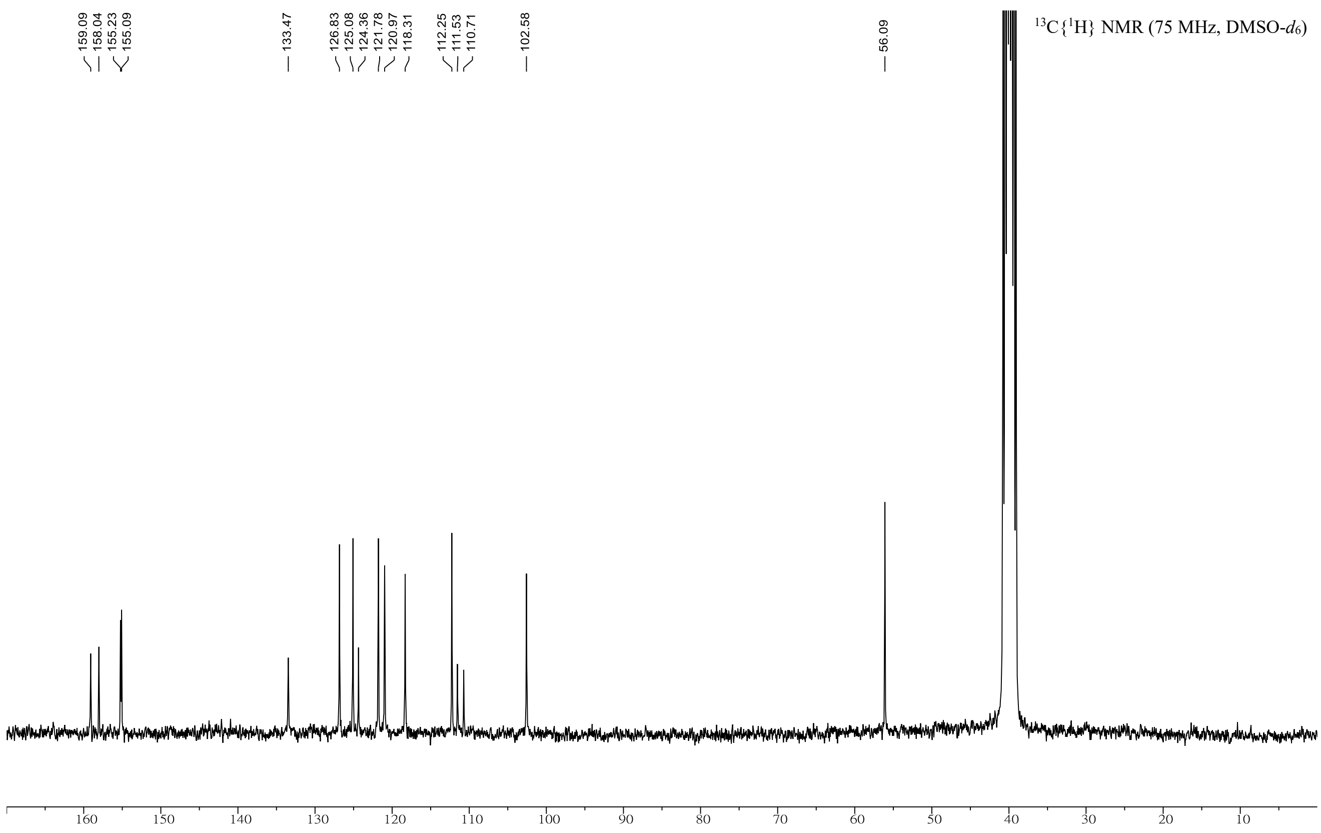

3-Fluorobenzofuro[3,2-*c*]quinolin-6(5*H*)-one (1h)

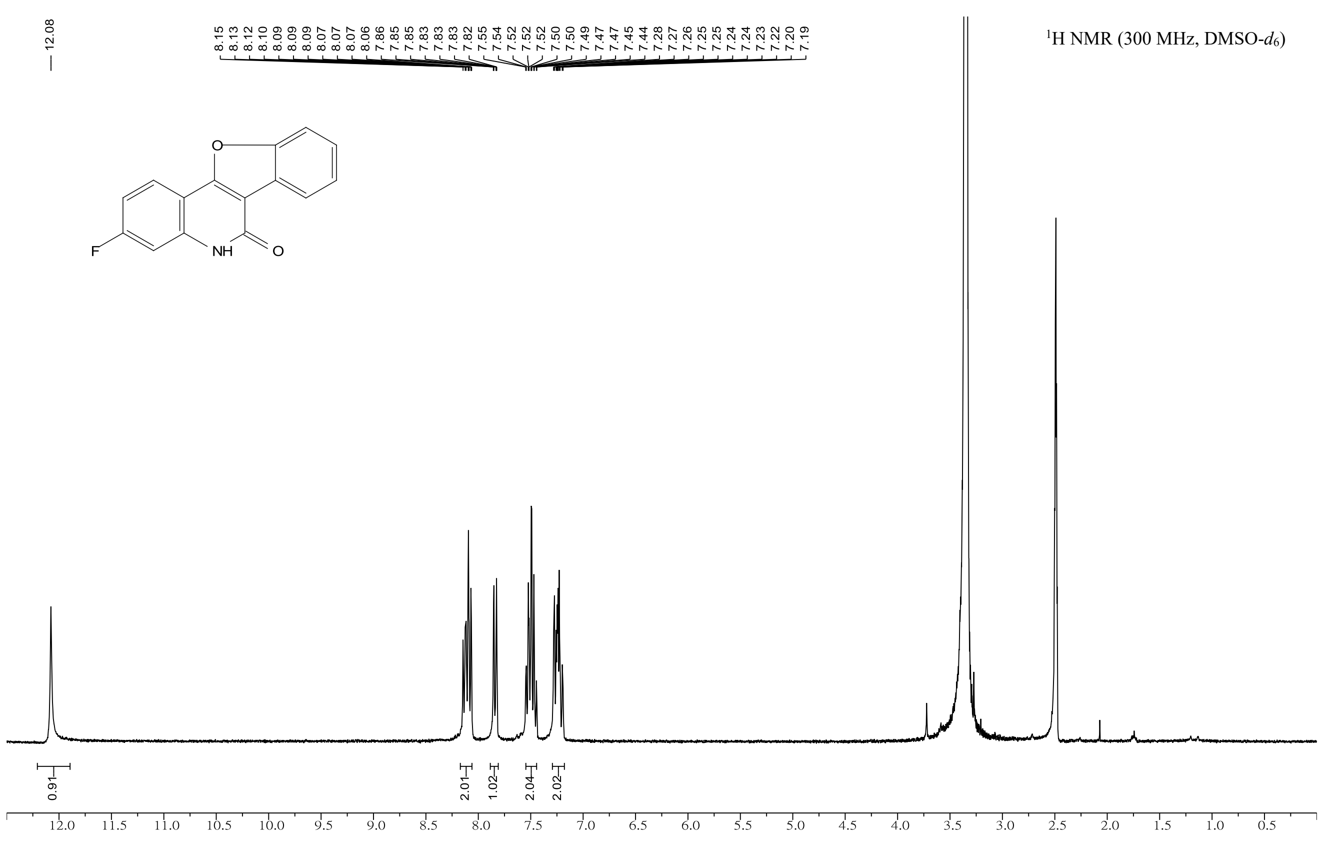

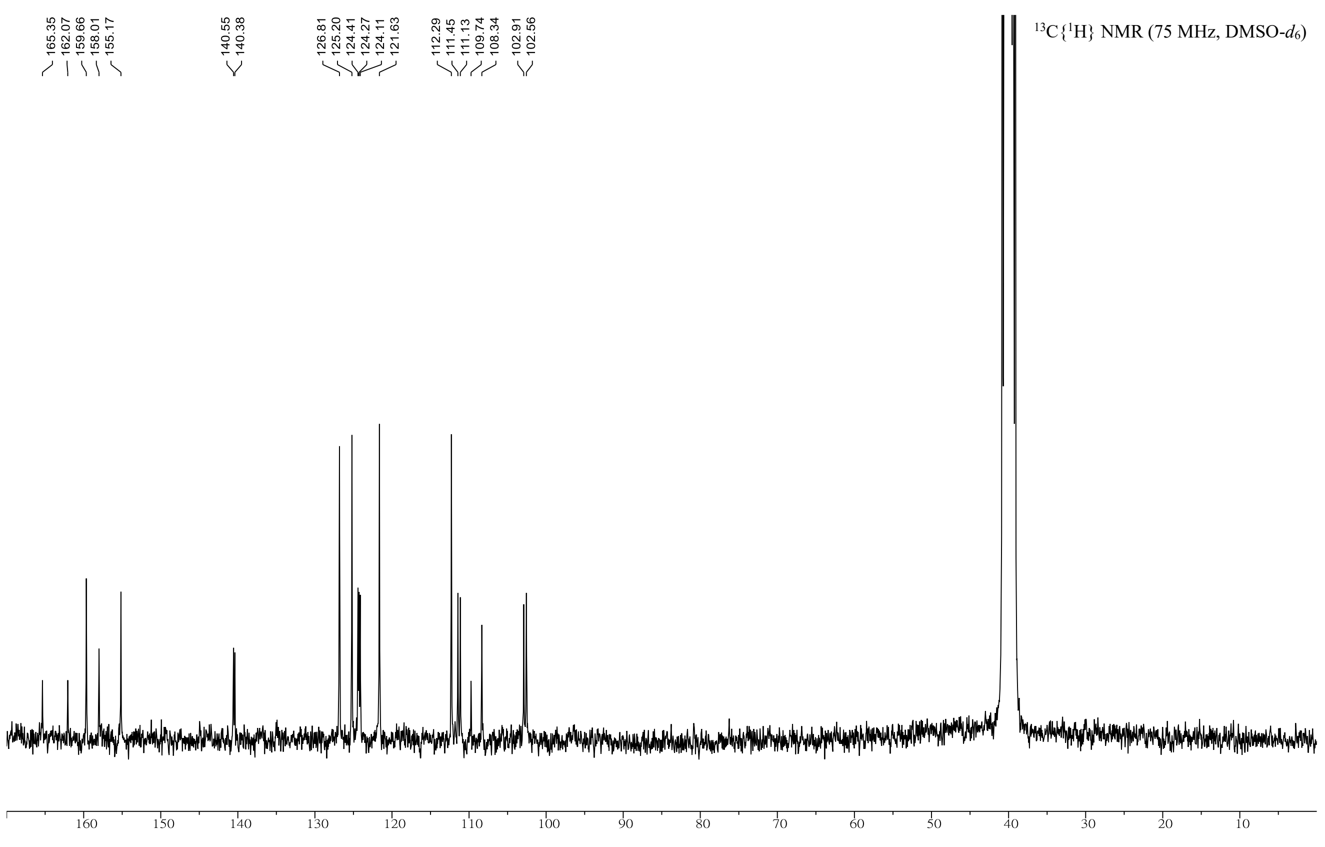

3-Chlorobenzofuro[3,2-*c*]quinolin-6(5*H*)-one (1i)

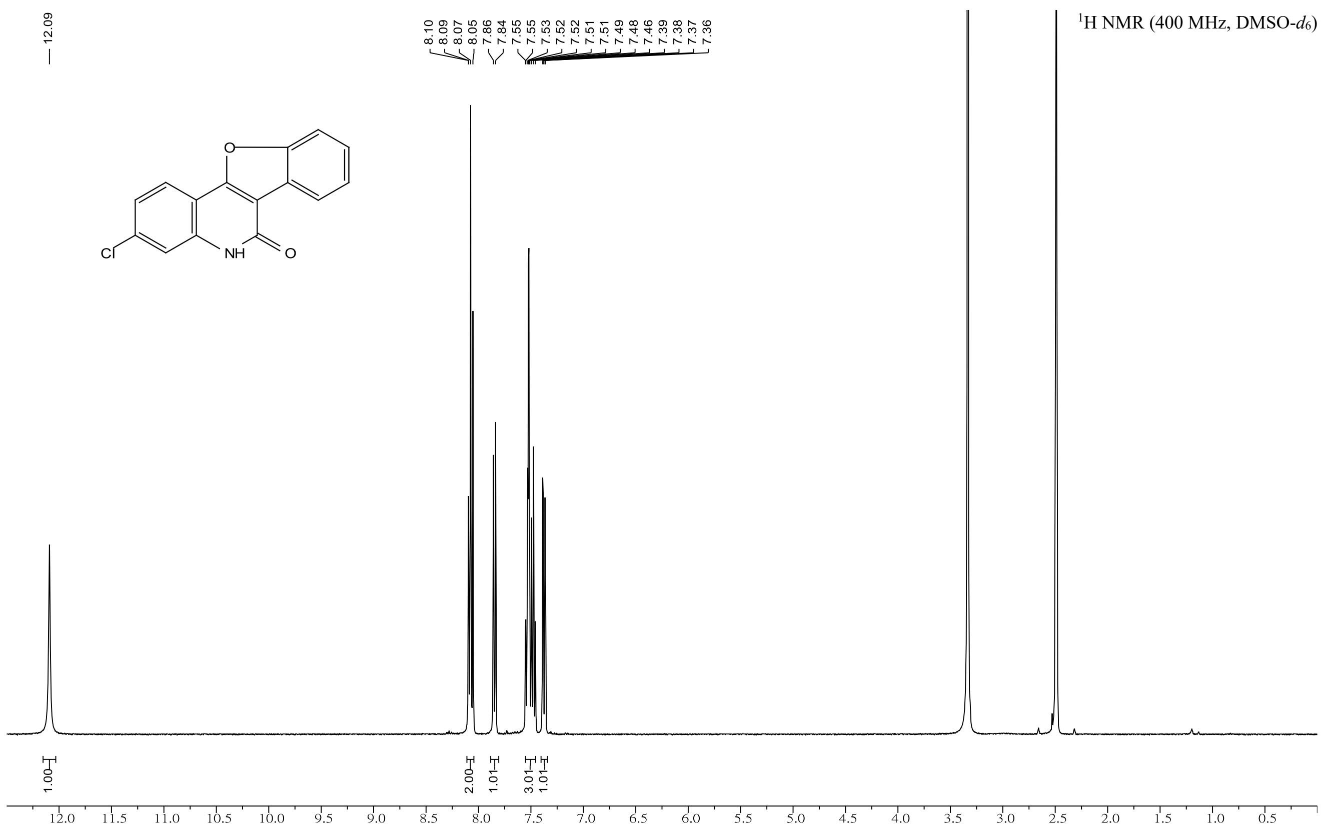

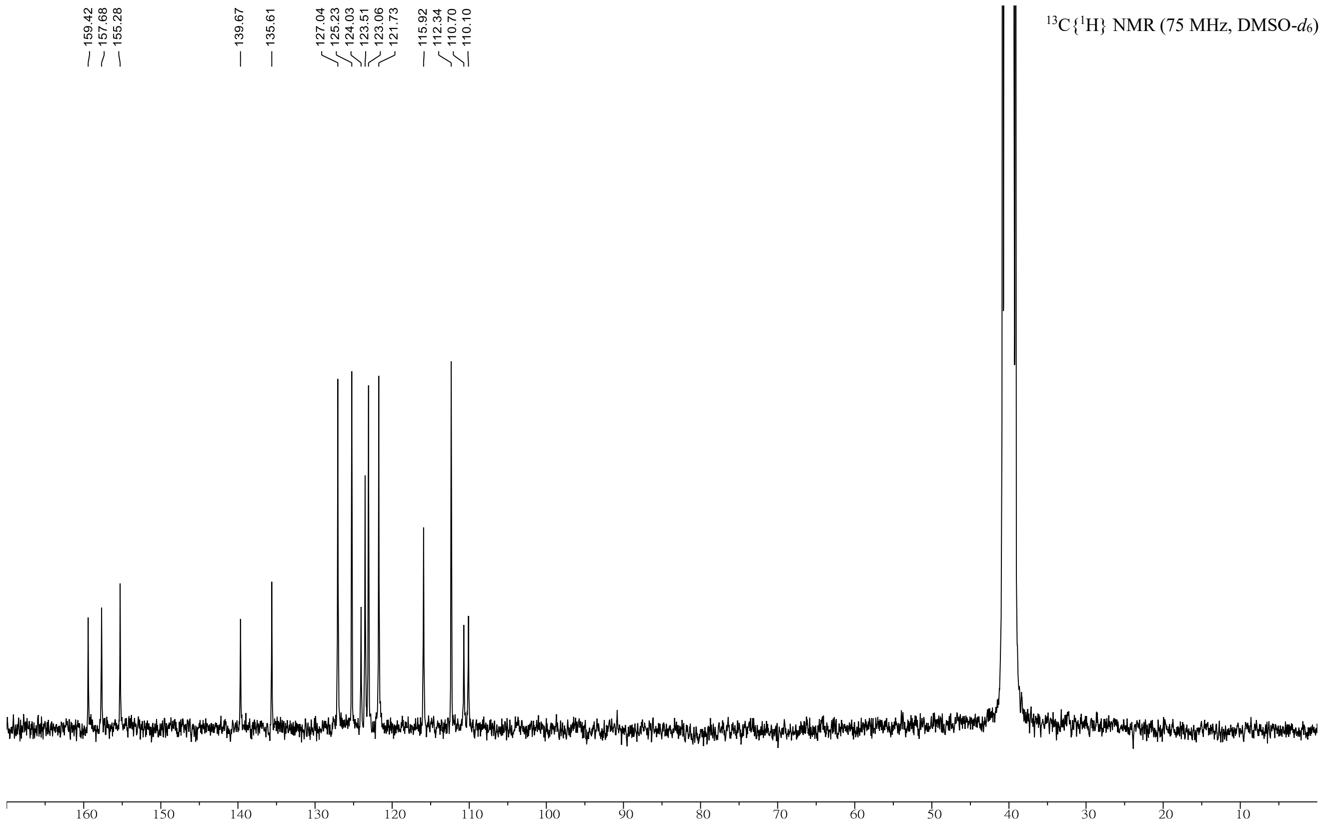

3-Bromobenzofuro[3,2-*c*]quinolin-6(5*H*)-one (1j)

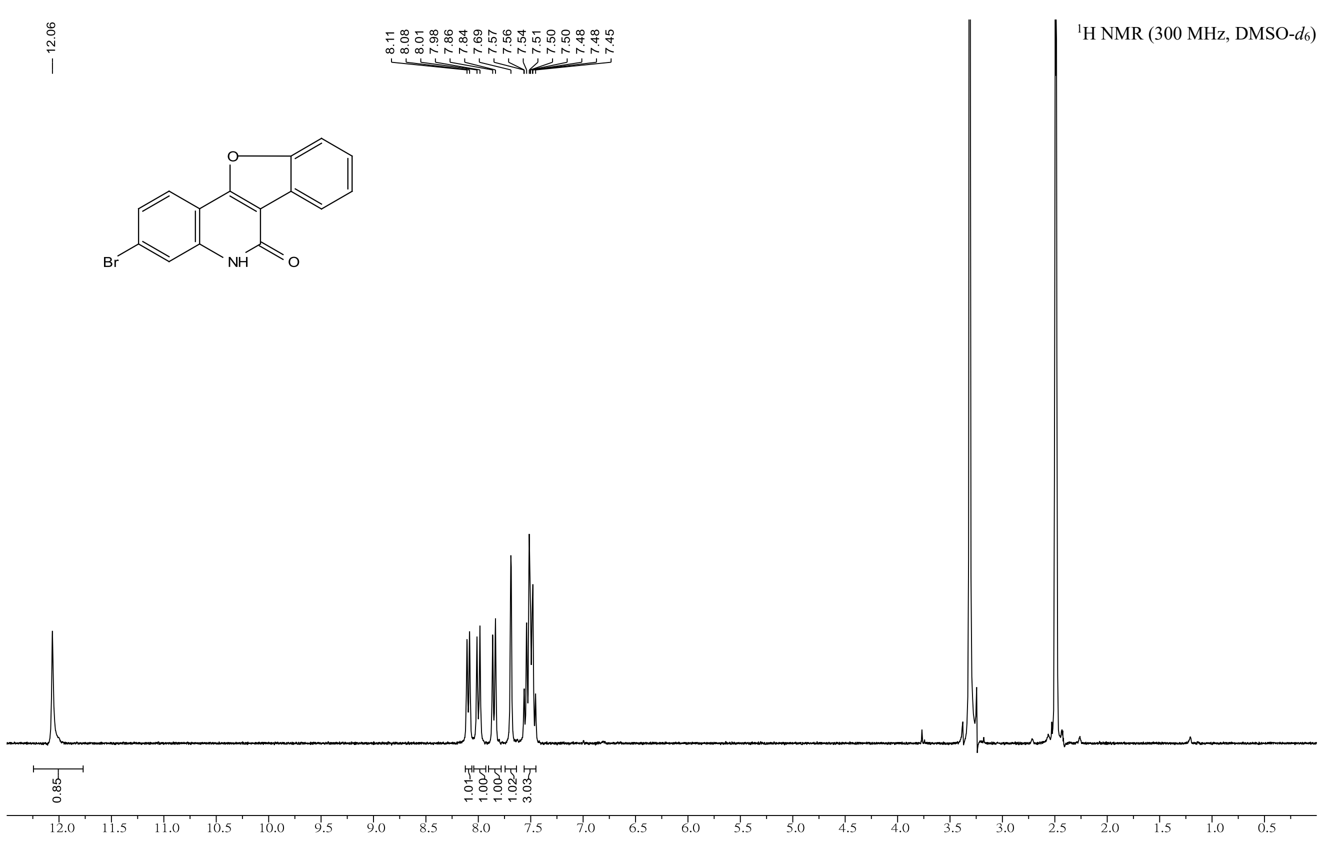

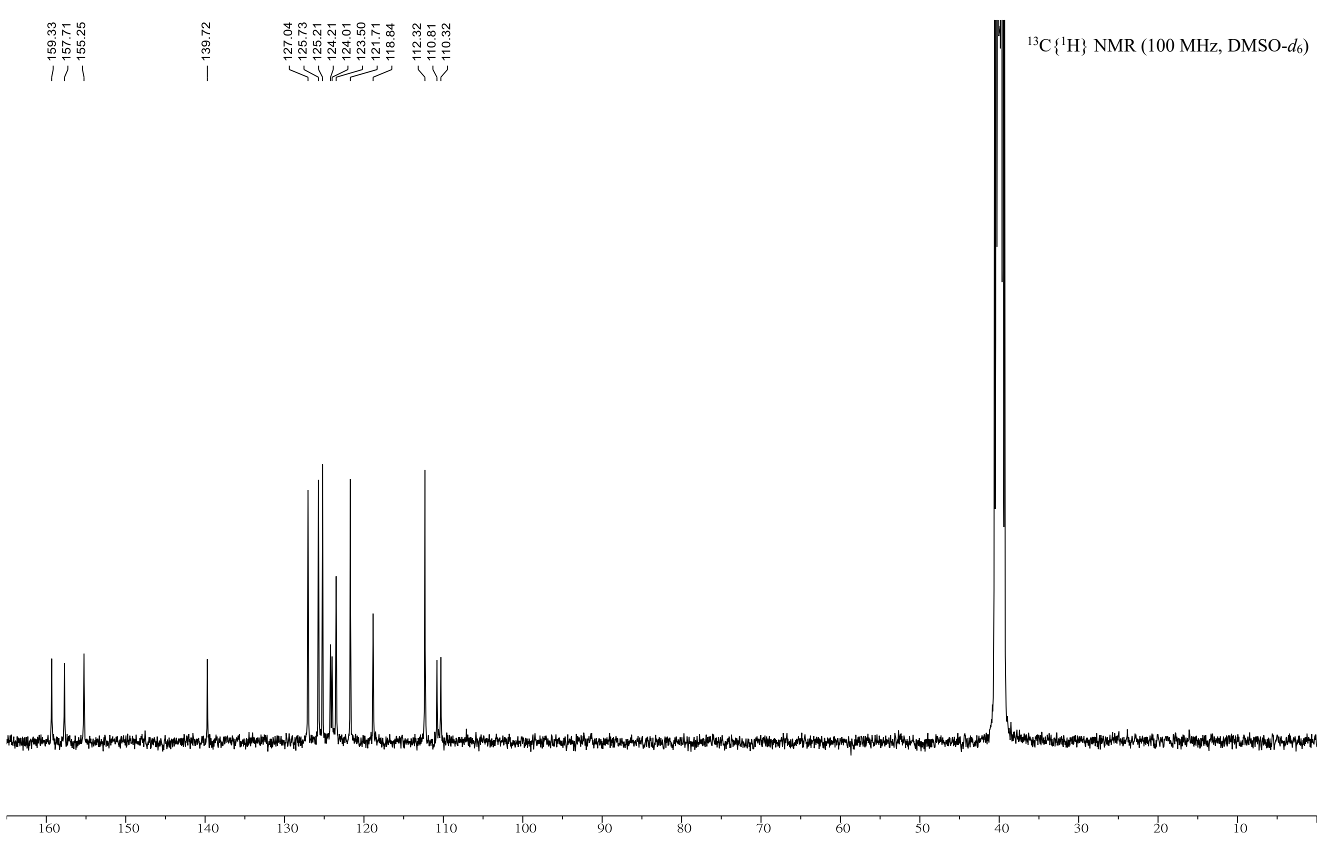

3-Methylbenzofuro[3,2-*c*]quinolin-6(5*H*)-one (1k)

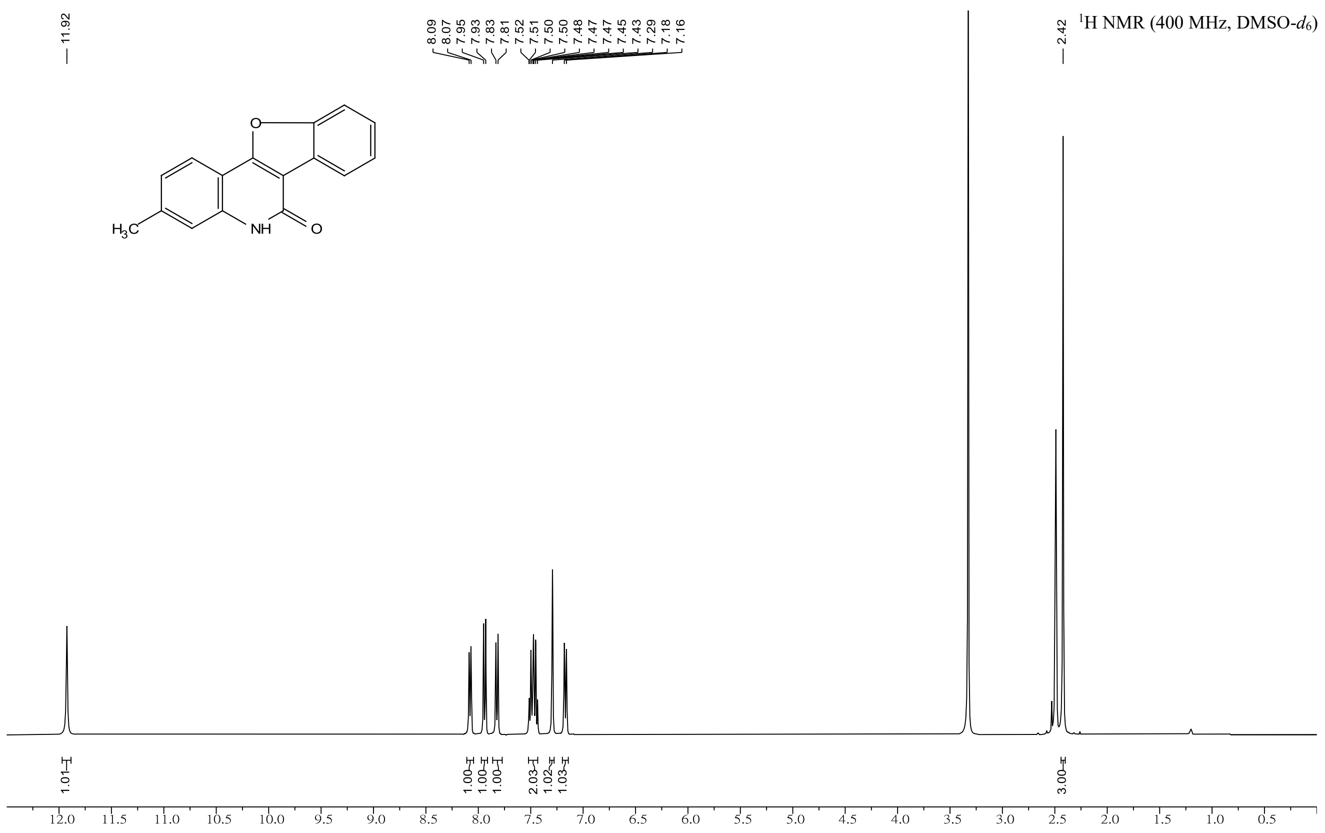

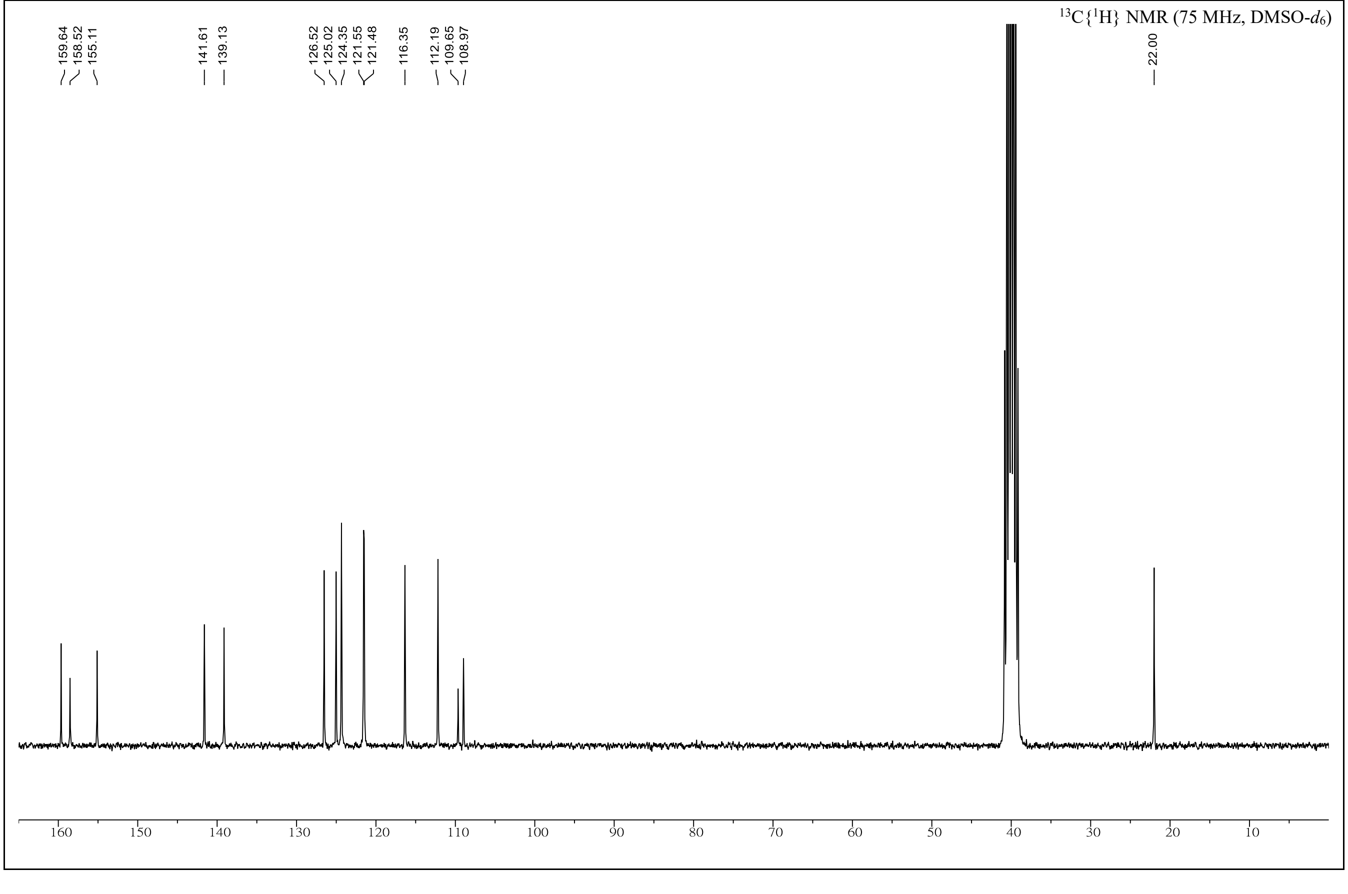

3-Nitrobenzofuro[3,2-*c*]quinolin-6(5*H*)-one (1l)

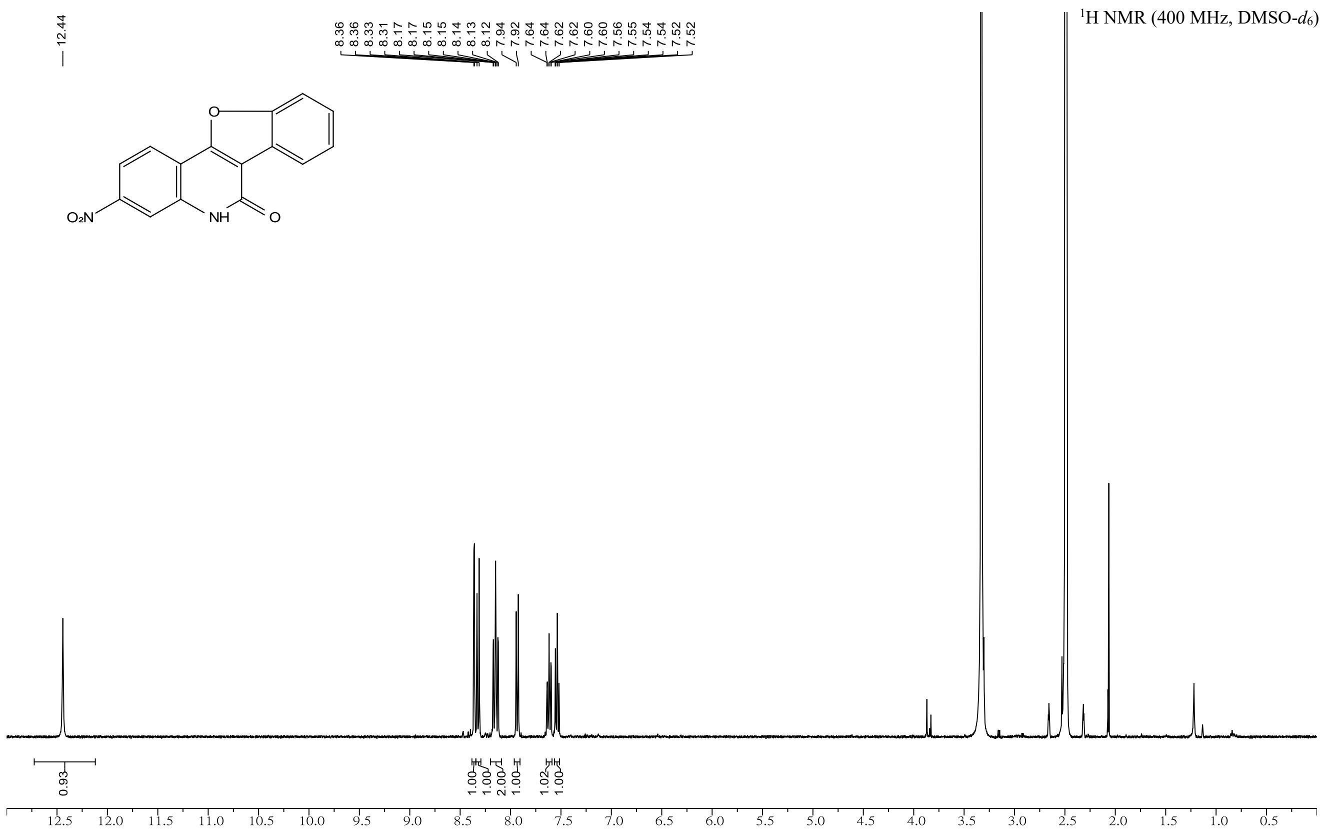

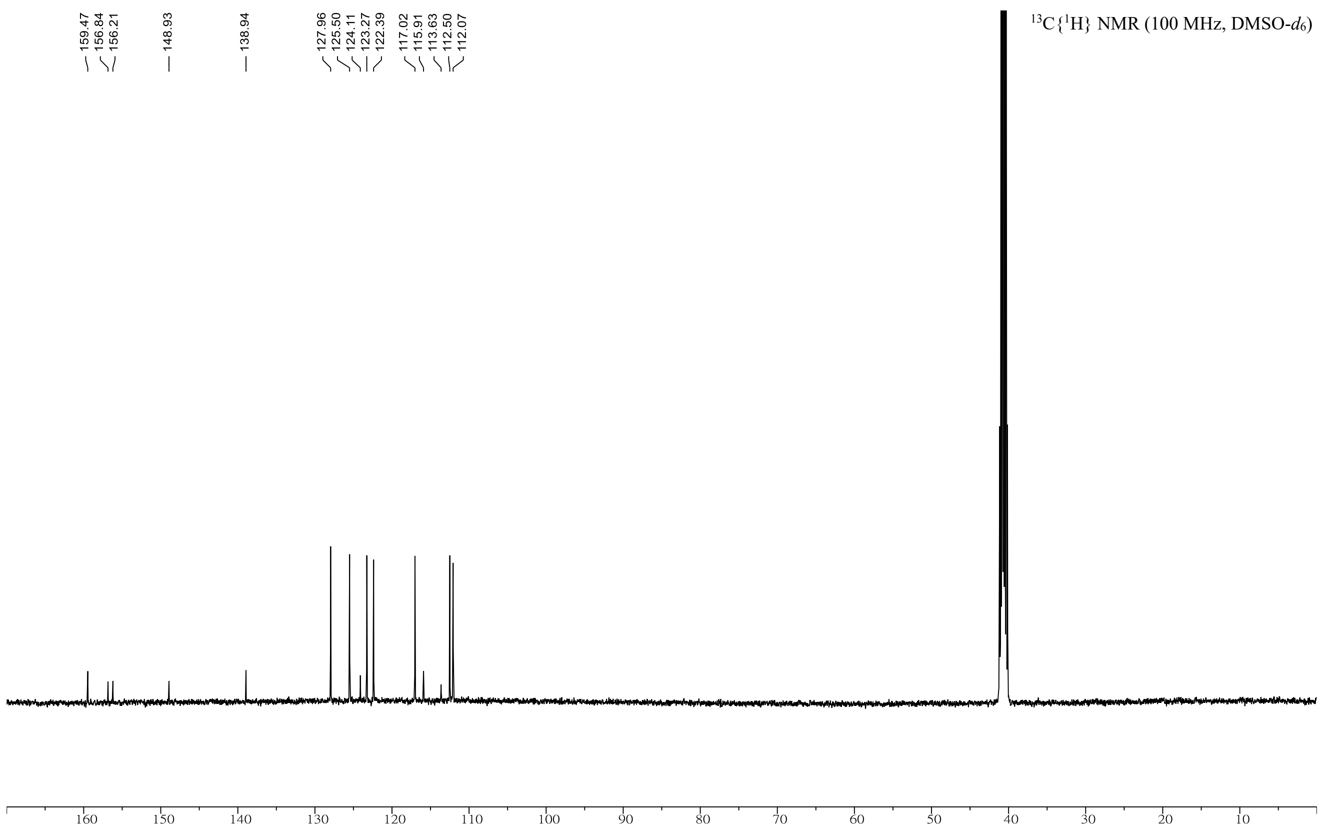

3-Methoxybenzofuro[3,2-*c*]quinolin-6(5*H*)-one (1m)

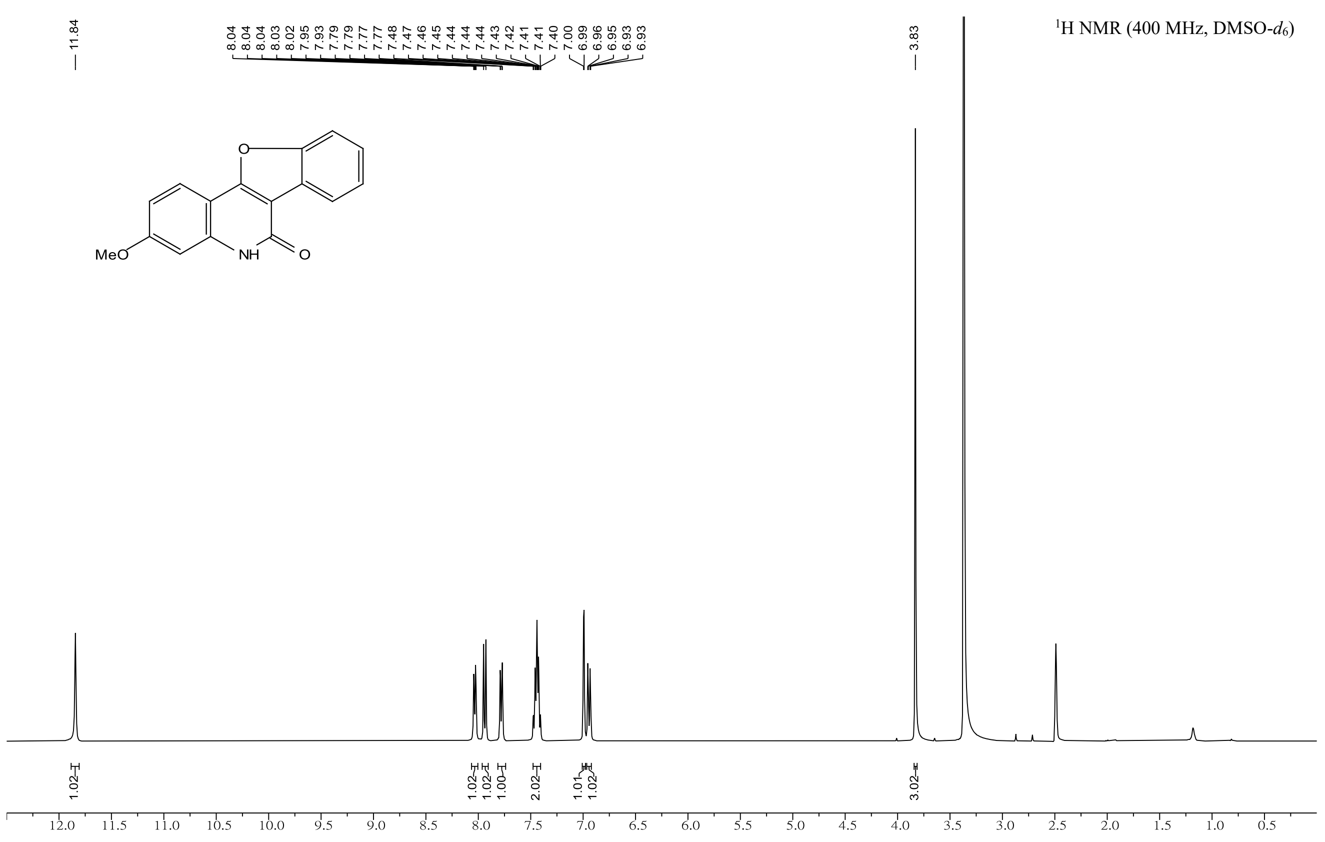

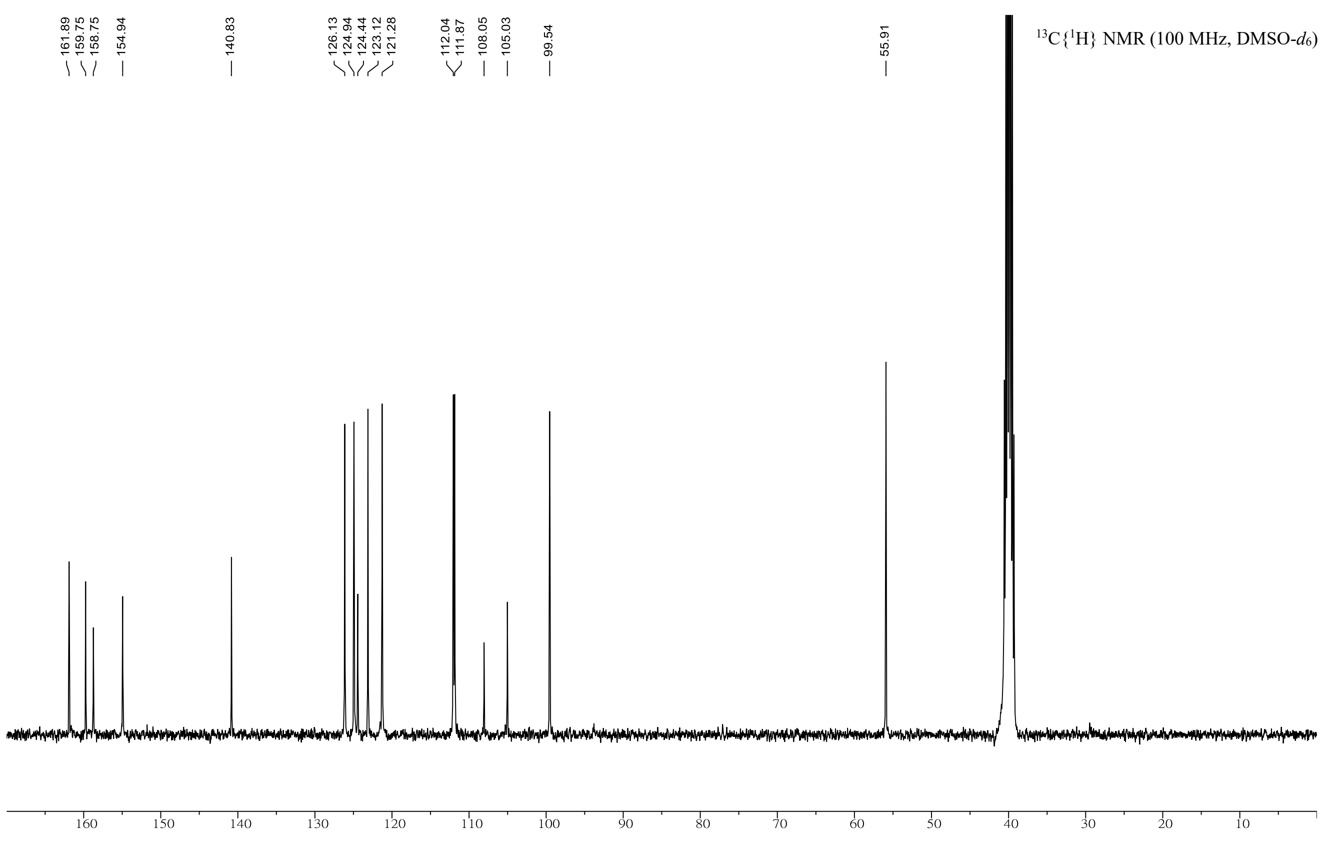

2,3-Dimethoxybenzofuro[3,2-*c*]quinolin-6(5*H*)-one (1n)

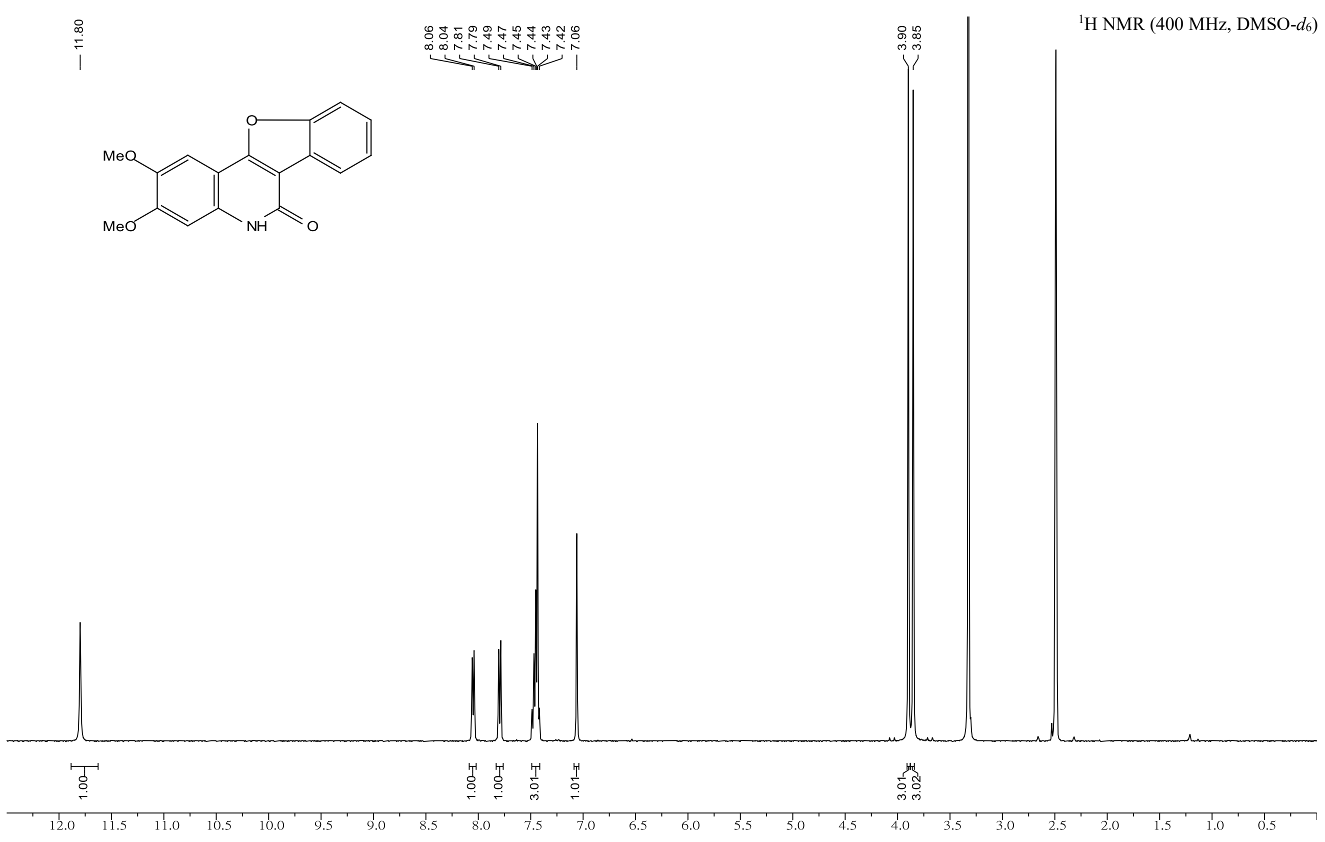

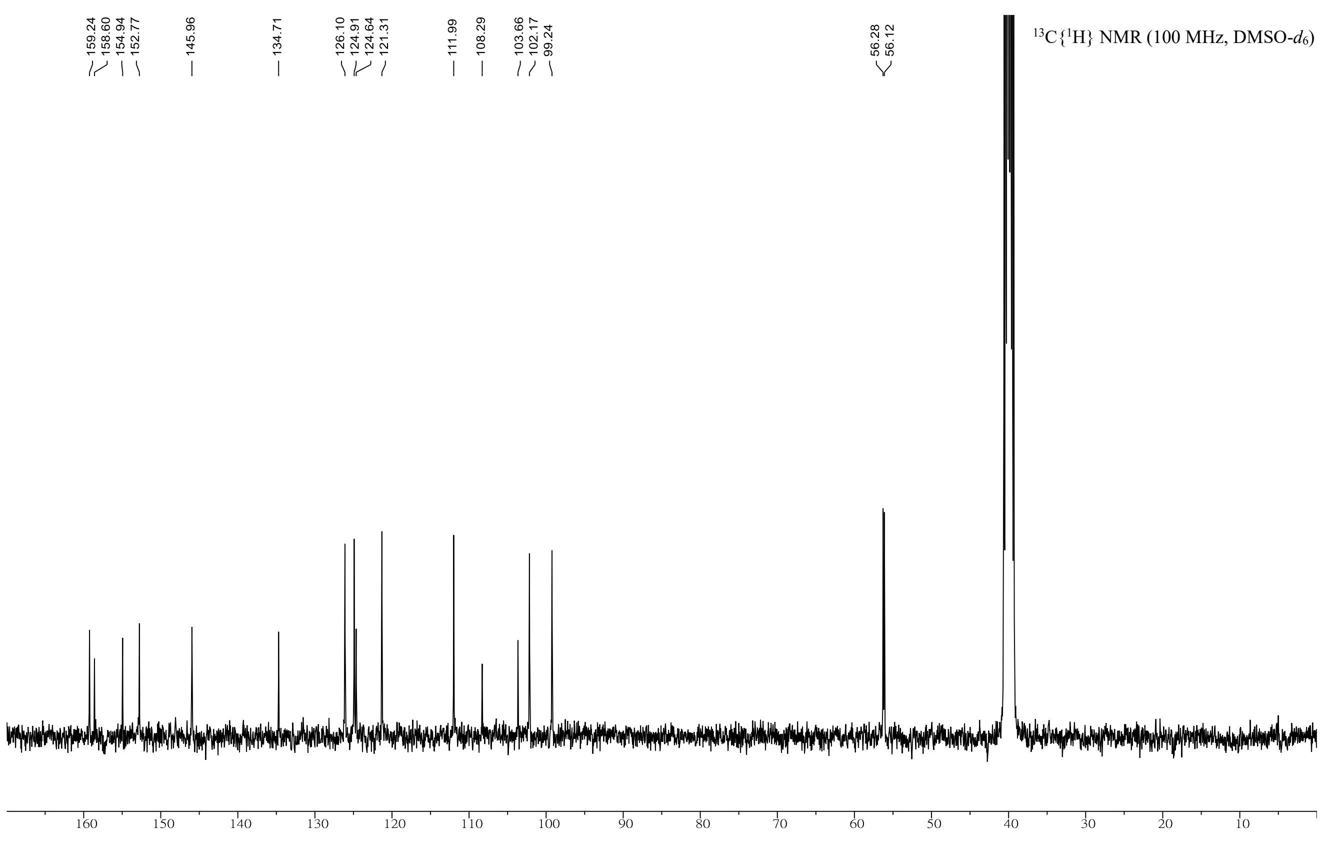

9-Fluorobenzofuro[3,2-*c*]quinolin-6(5*H*)-one (1o)

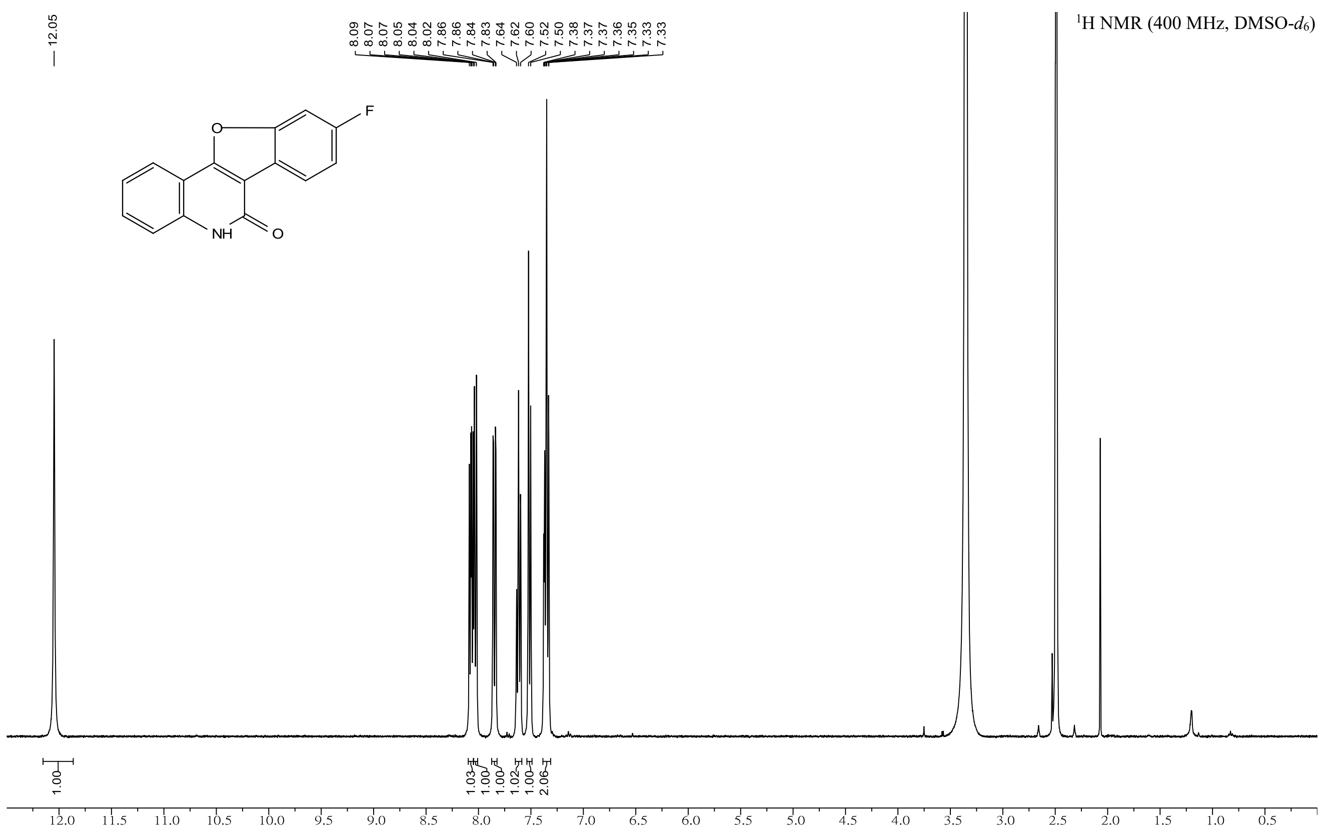

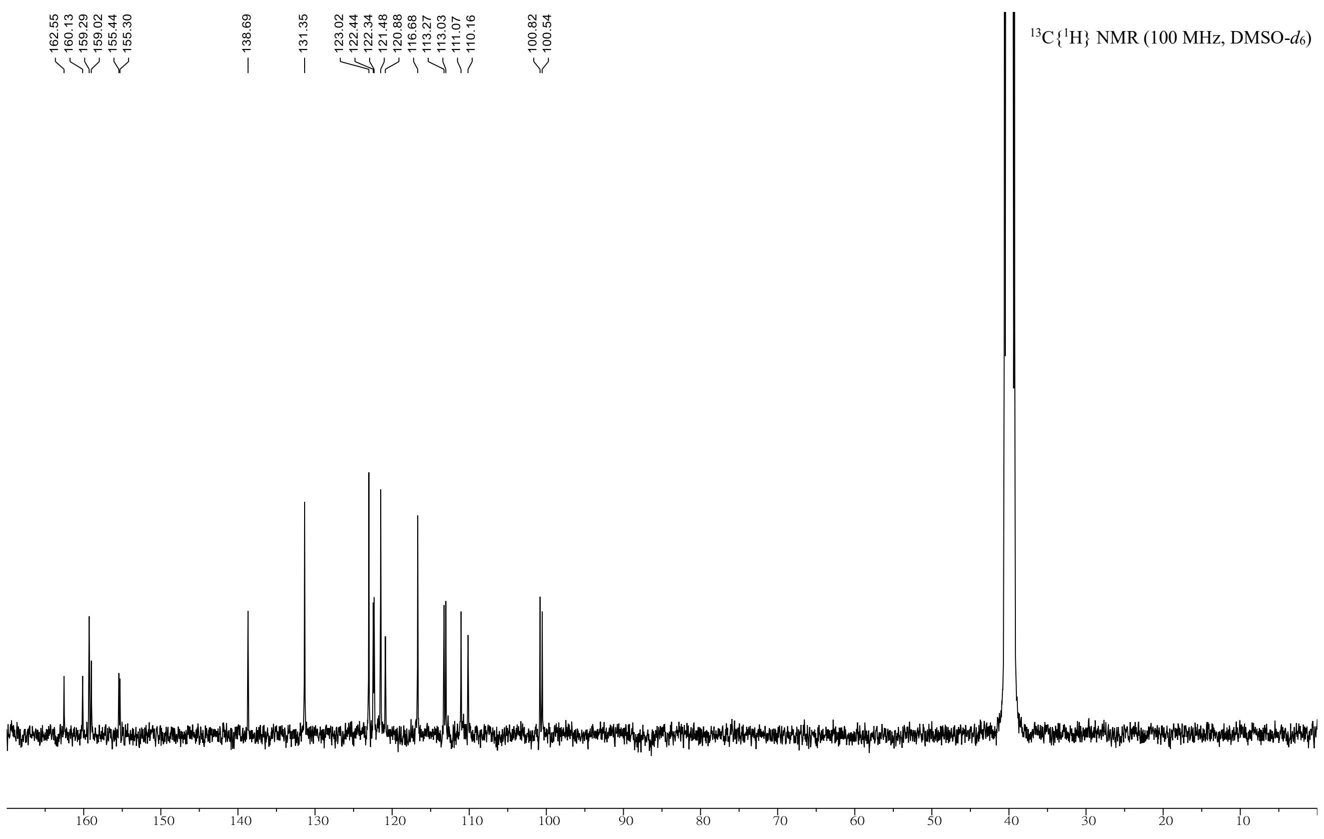

9-Chlorobenzofuro[3,2-*c*]quinolin-6(5*H*)-one (1p)

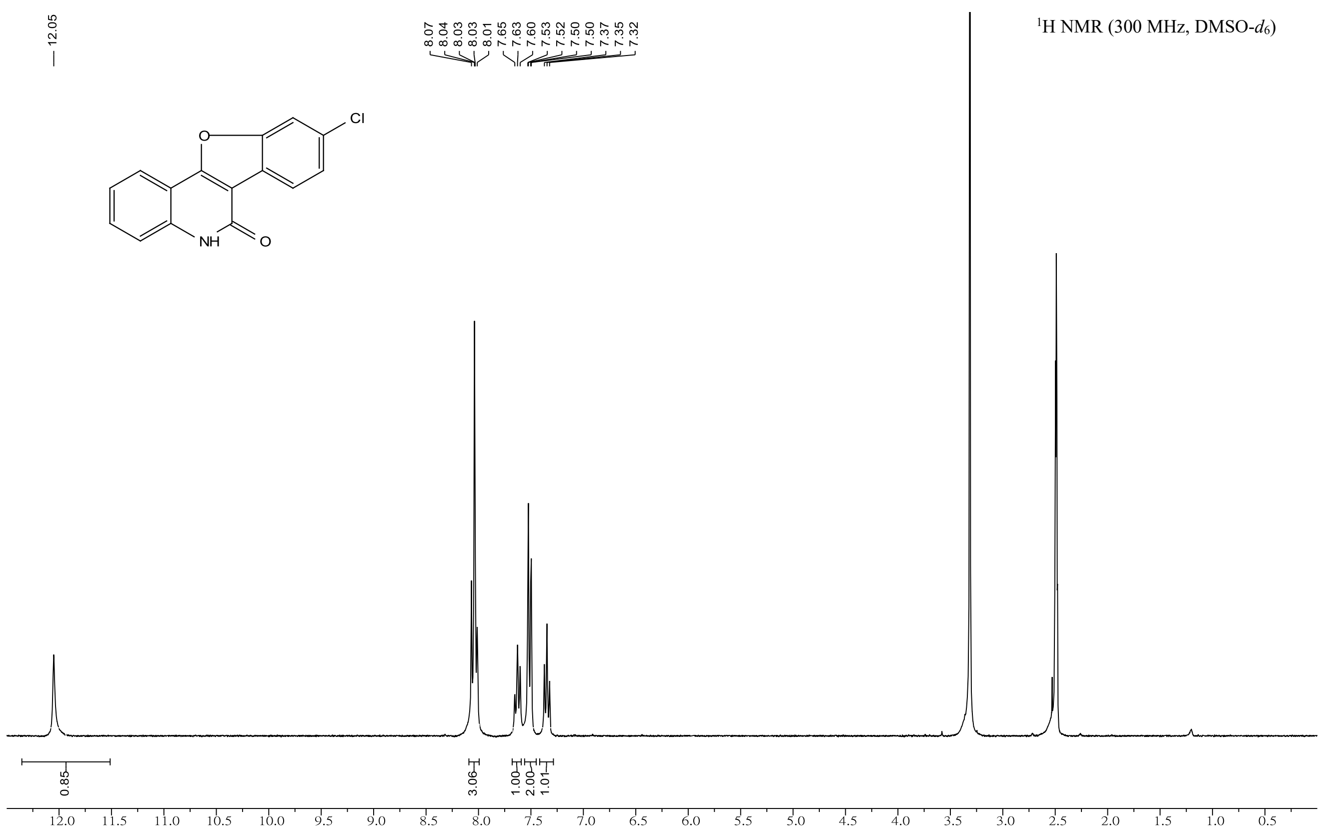

^{1}H NMR (300 MHz, DMSO-d_6)

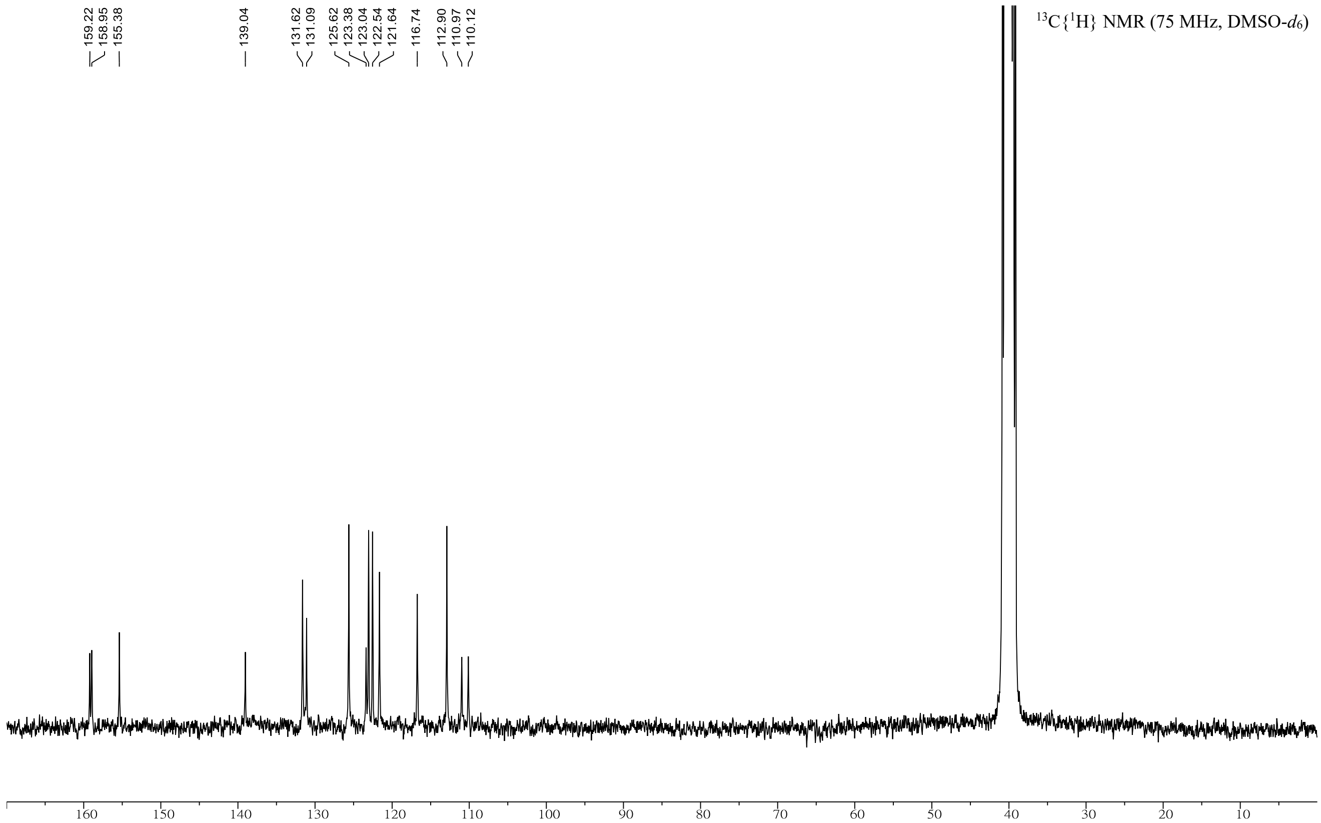

^{13}C{^{1}H} NMR (75 MHz, DMSO-d_6)

9-Bromobenzofuro[3,2-*c*]quinolin-6(5*H*)-one (1q)

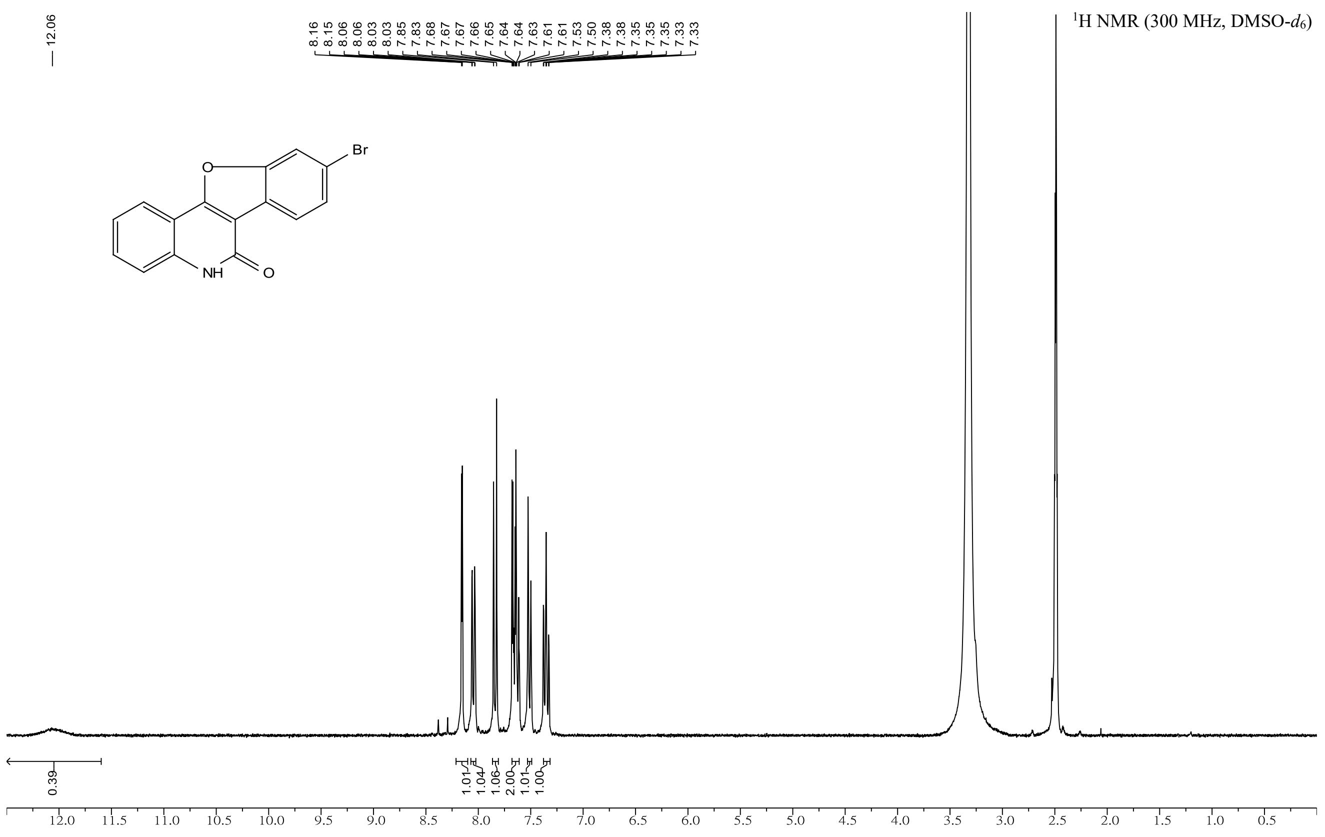

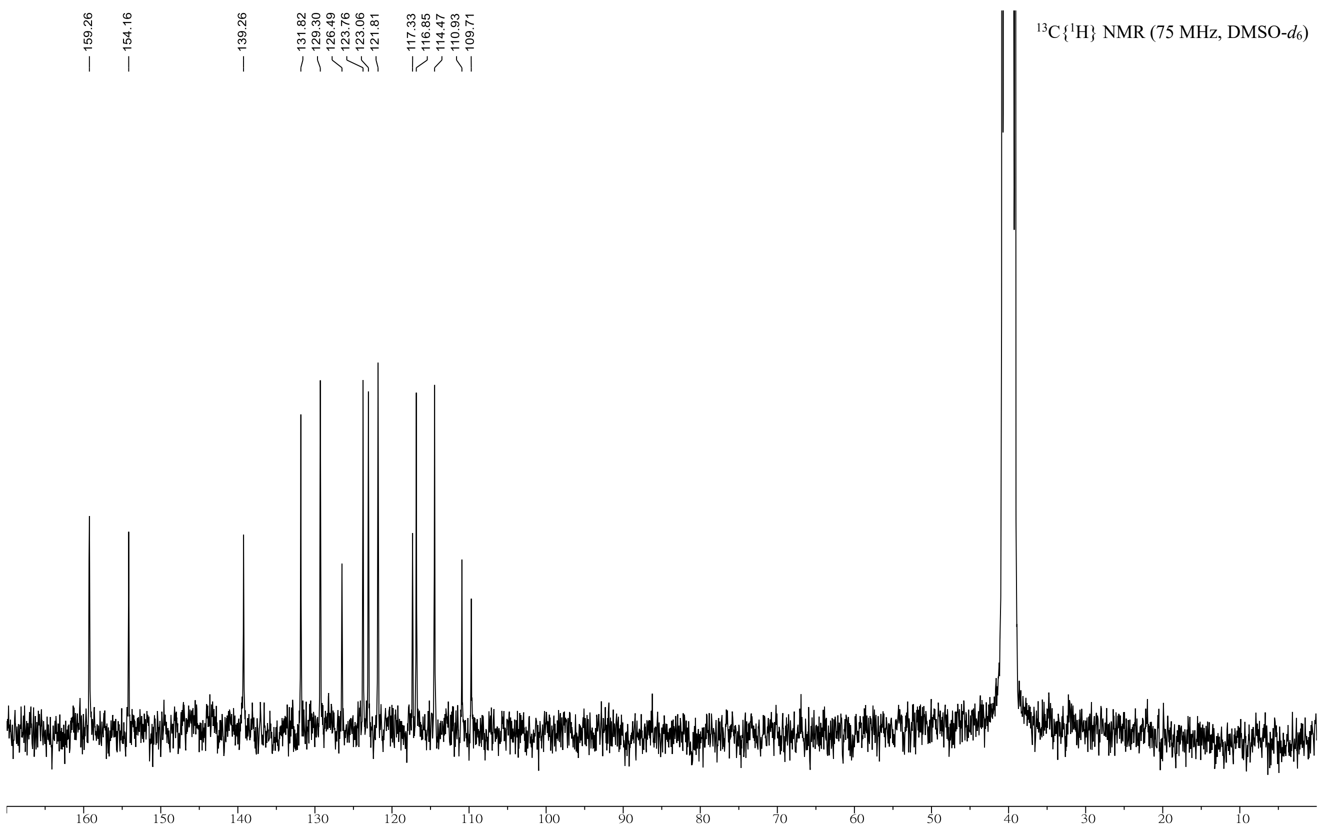

9-Nitrobenzofuro[3,2-*c*]quinolin-6(5*H*)-one (1r)

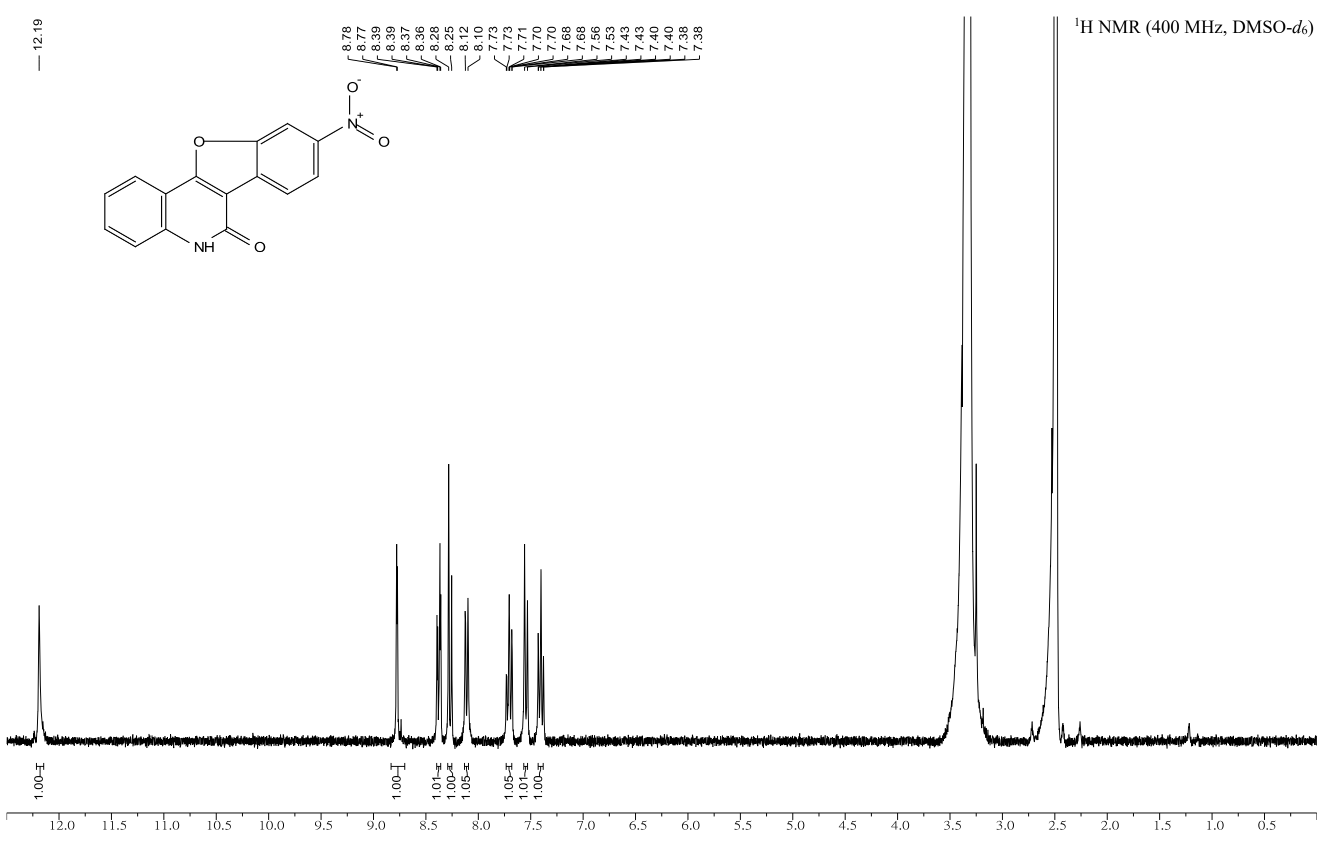

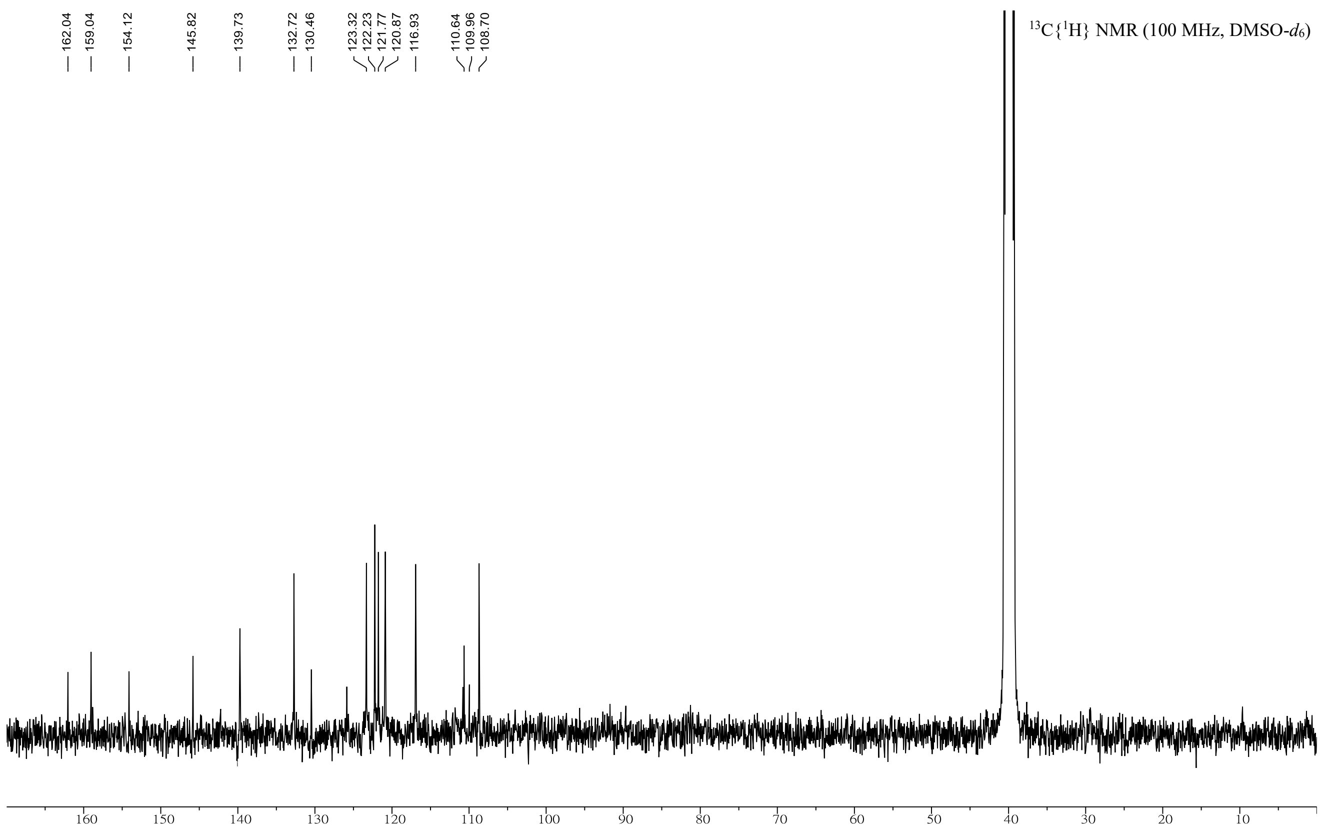

8-Fluorobenzofuro[3,2-*c*]quinolin-6(5*H*)-one (1s)

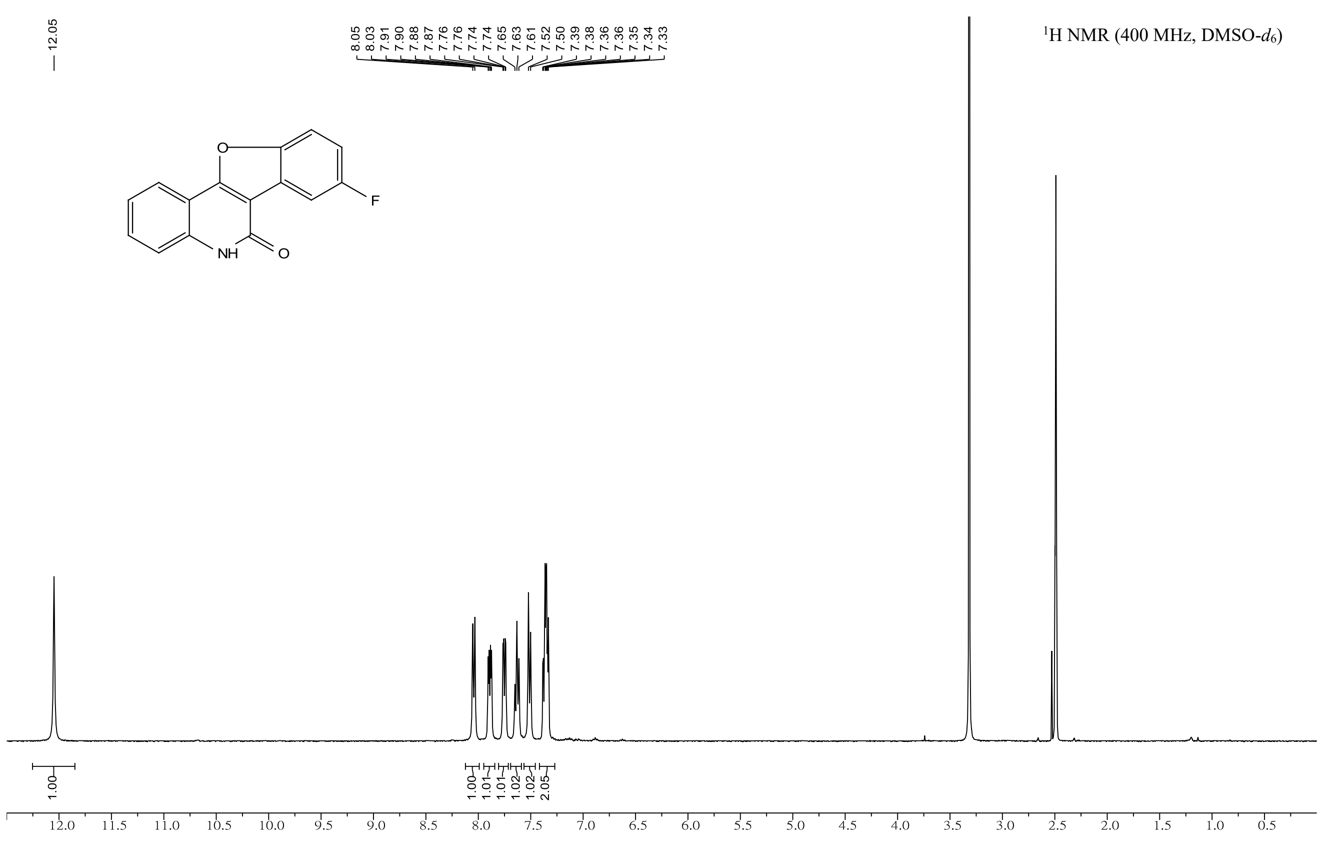

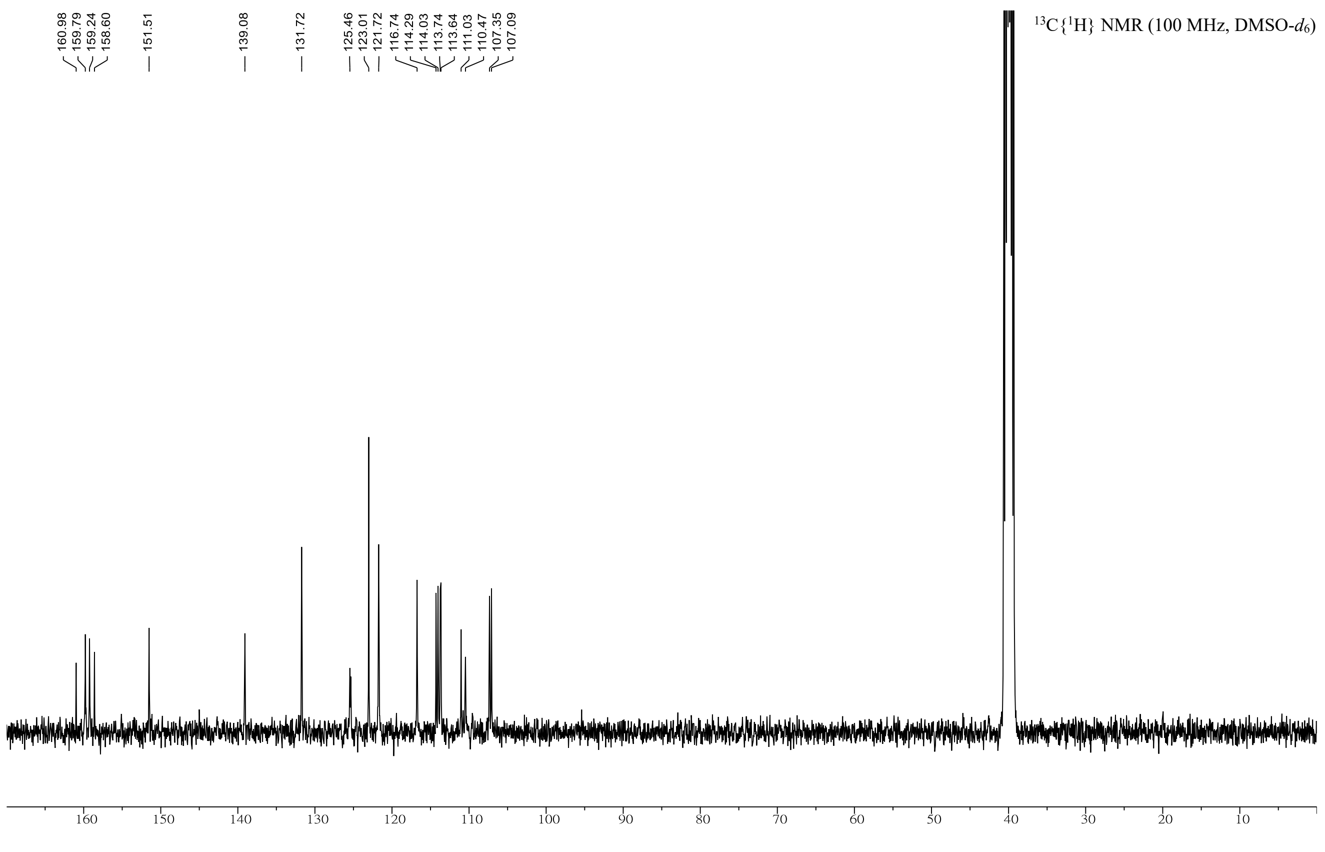

8-Chlorobenzofuro[3,2-*c*]quinolin-6(5*H*)-one (1t)

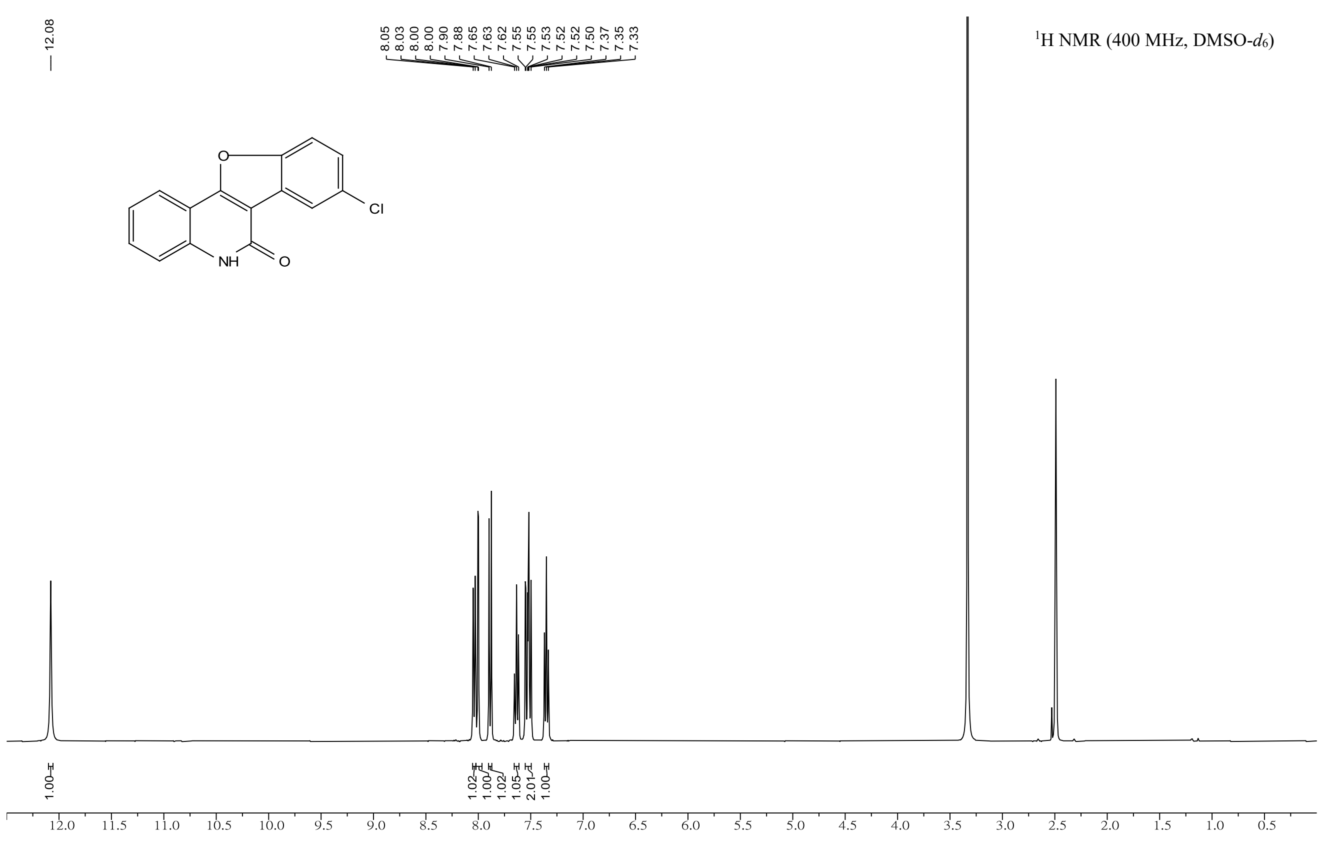

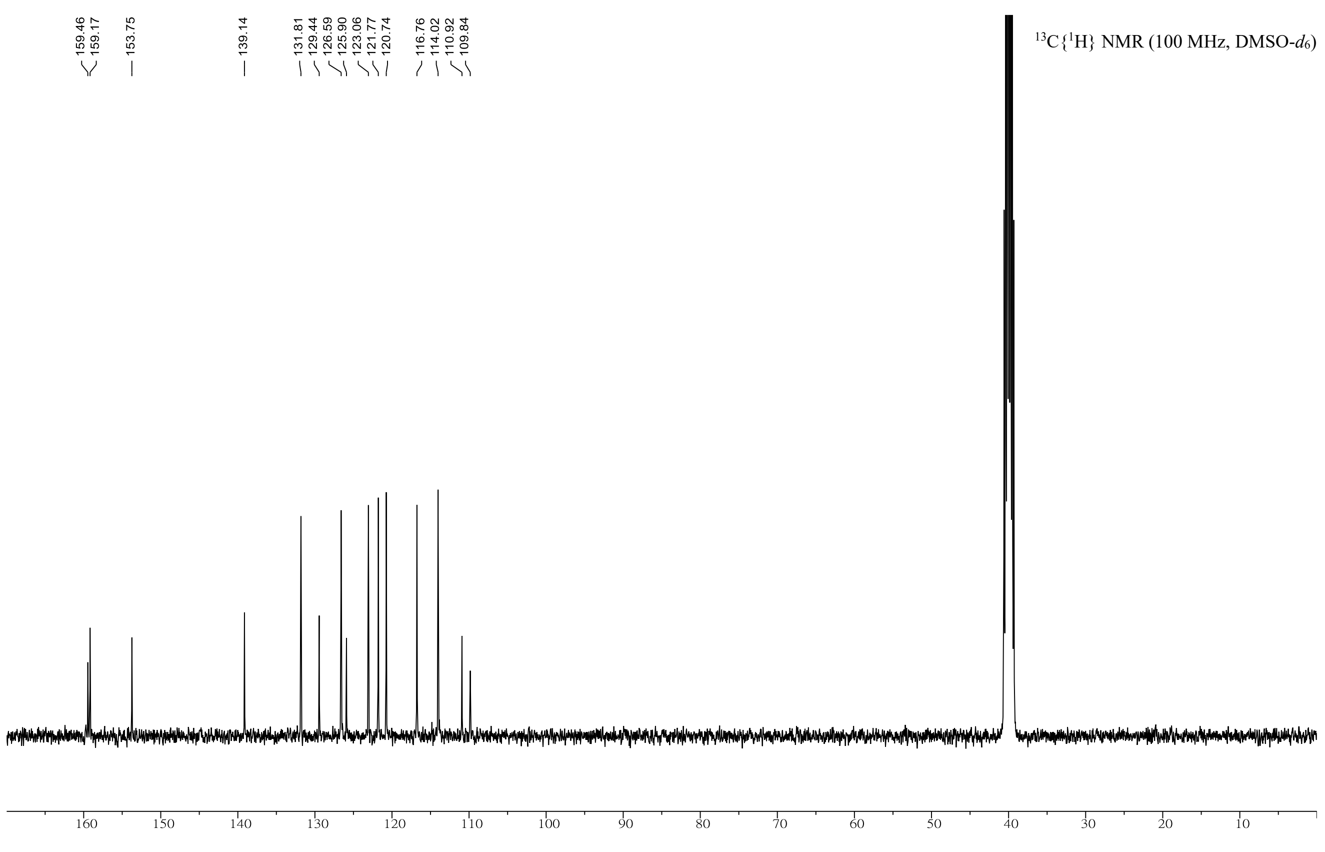

8,9-Dimethoxybenzofuro[3,2-*c*]quinolin-6(5*H*)-one (1u)

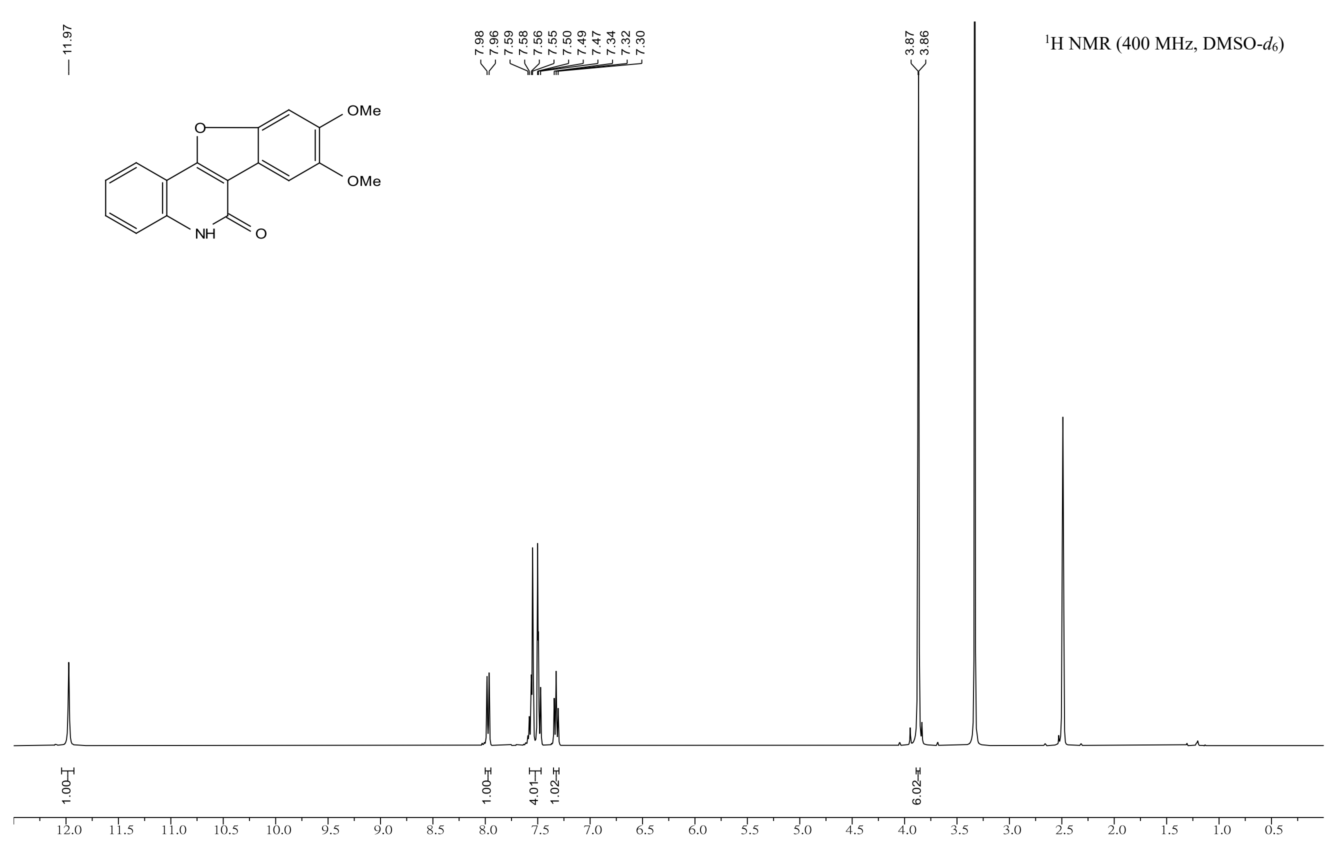

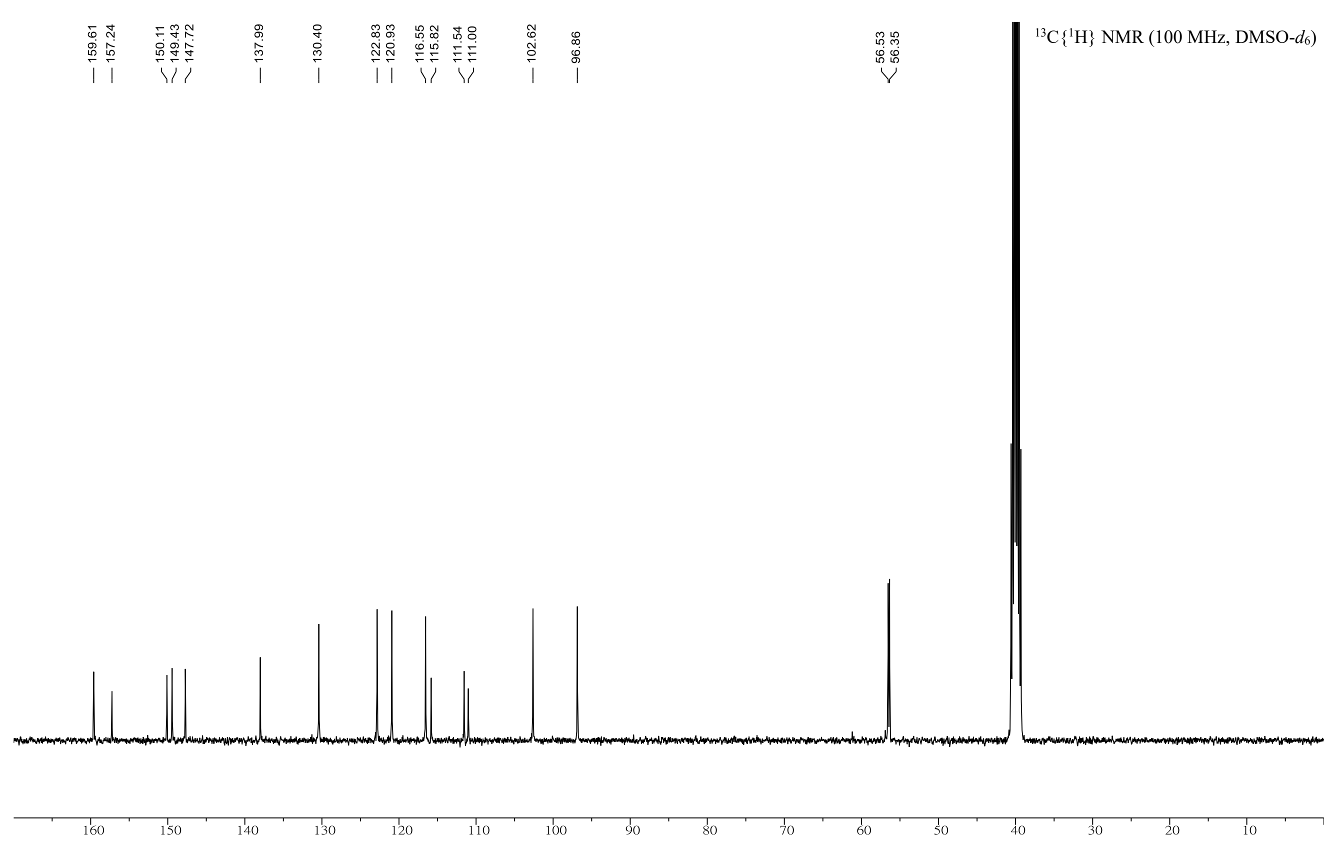

[1,3]Dioxolo[4',5':5,6]benzofuro[3,2-*c*]quinolin-6(5*H*)-one (1v)

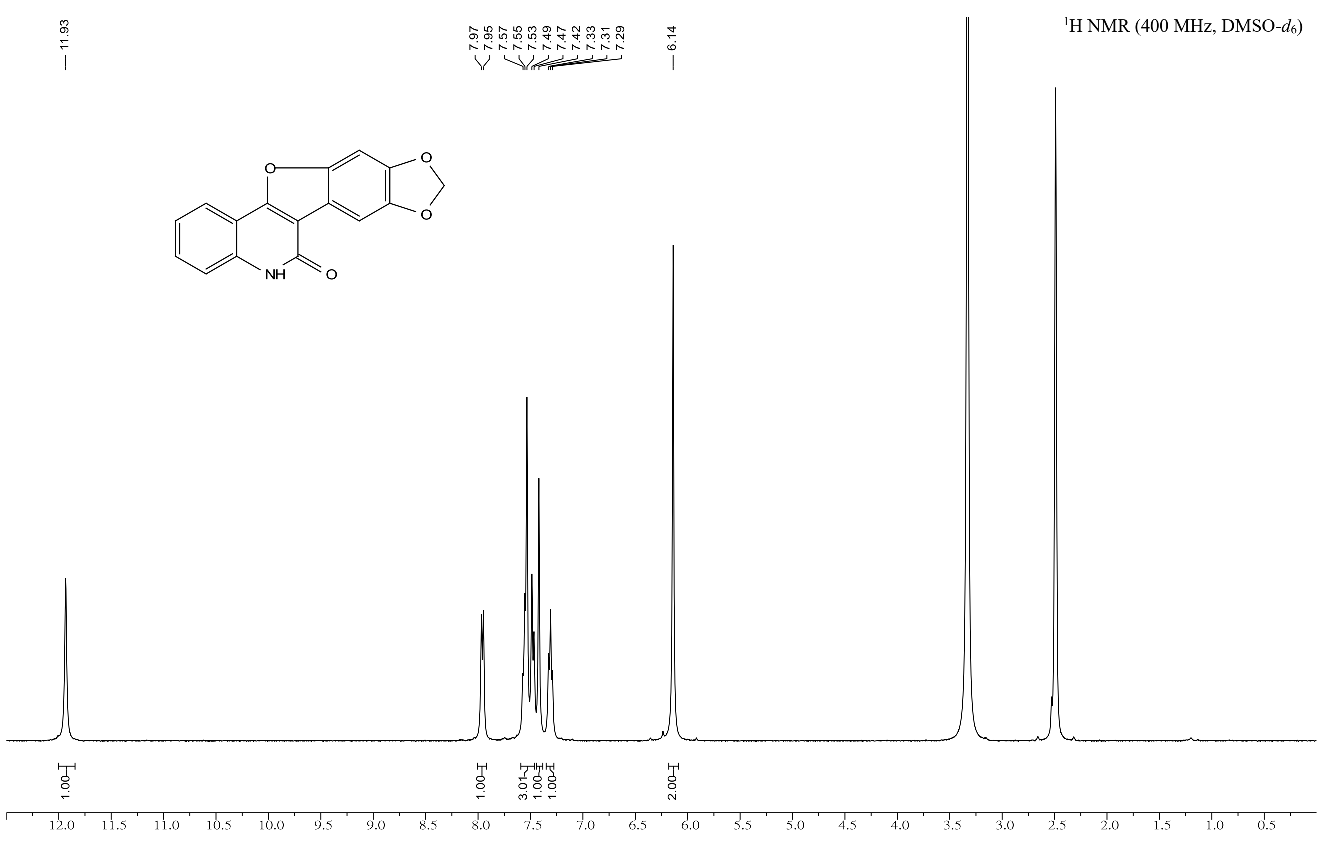

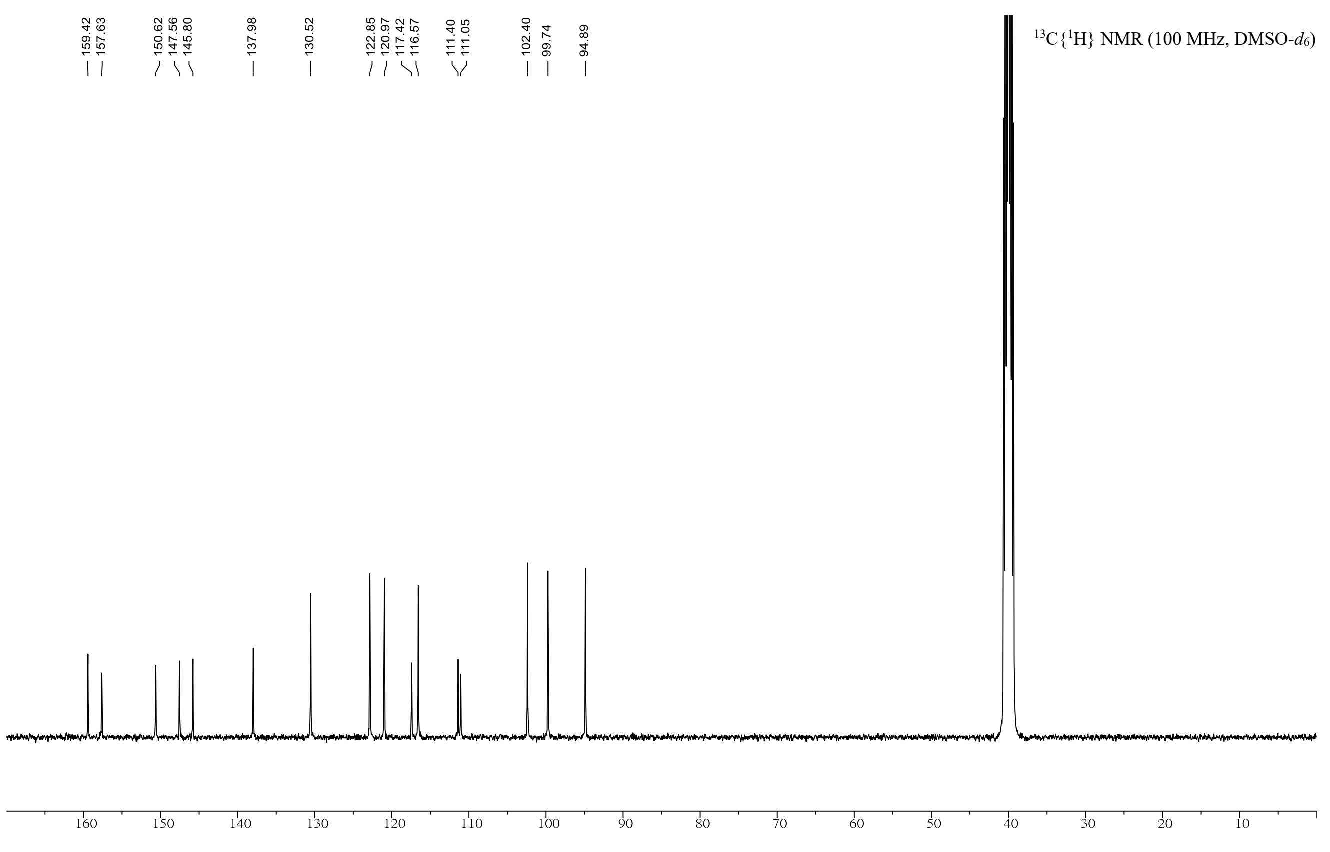

3,9-Difluorobenzofuro[3,2-*c*]quinolin-6(*5H*)-one (1w)

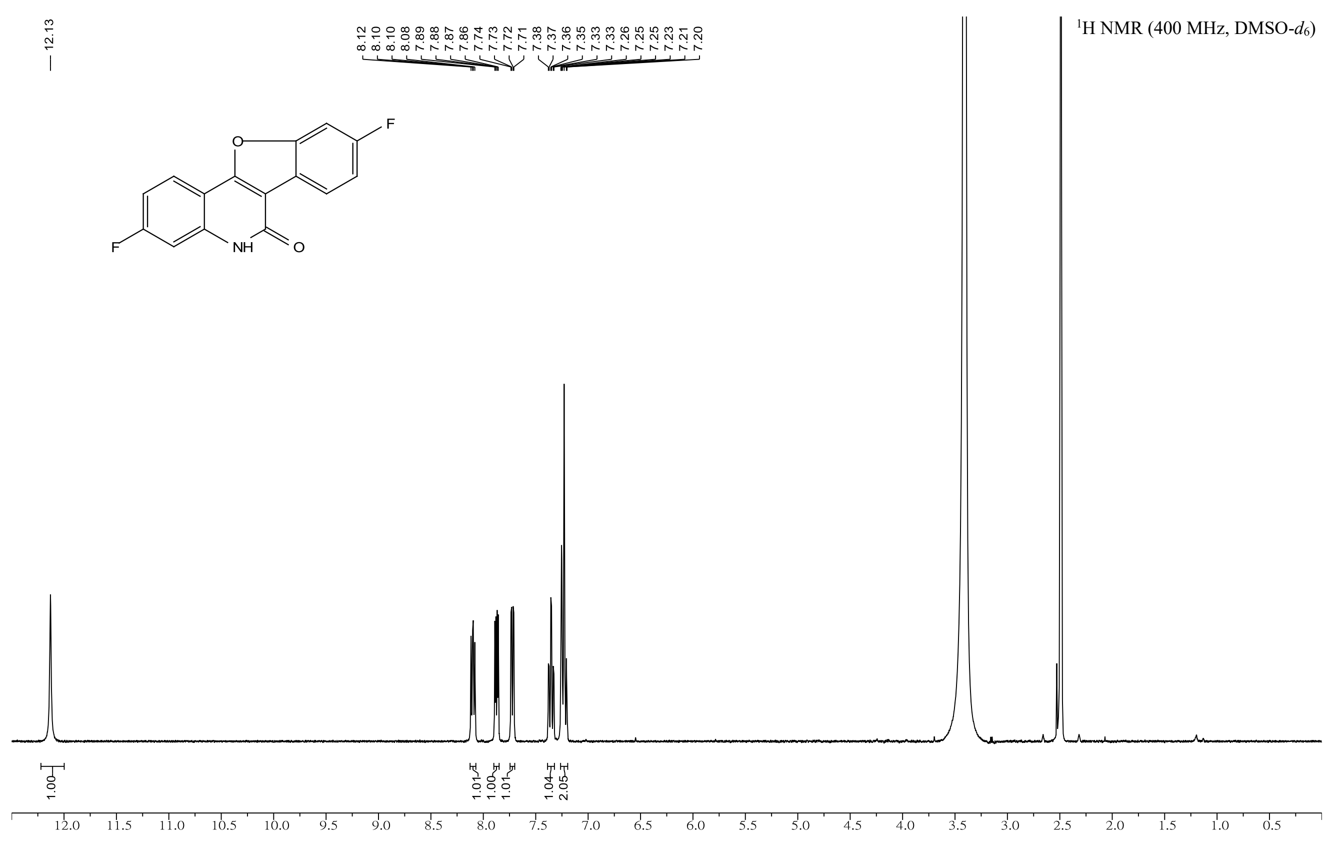

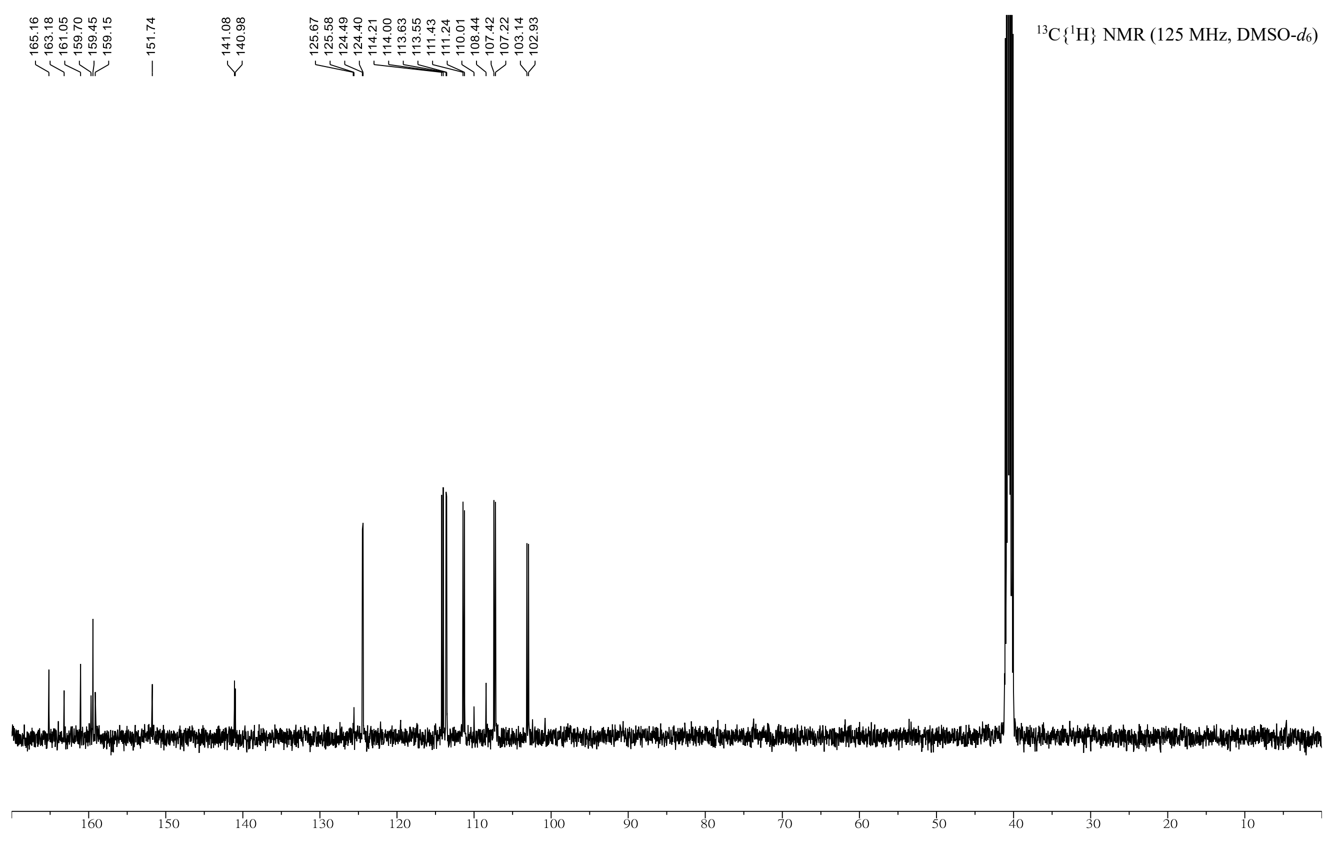

3,8-Difluorobenzofuro[3,2-*c*]quinolin-6(5*H*)-one (1x)

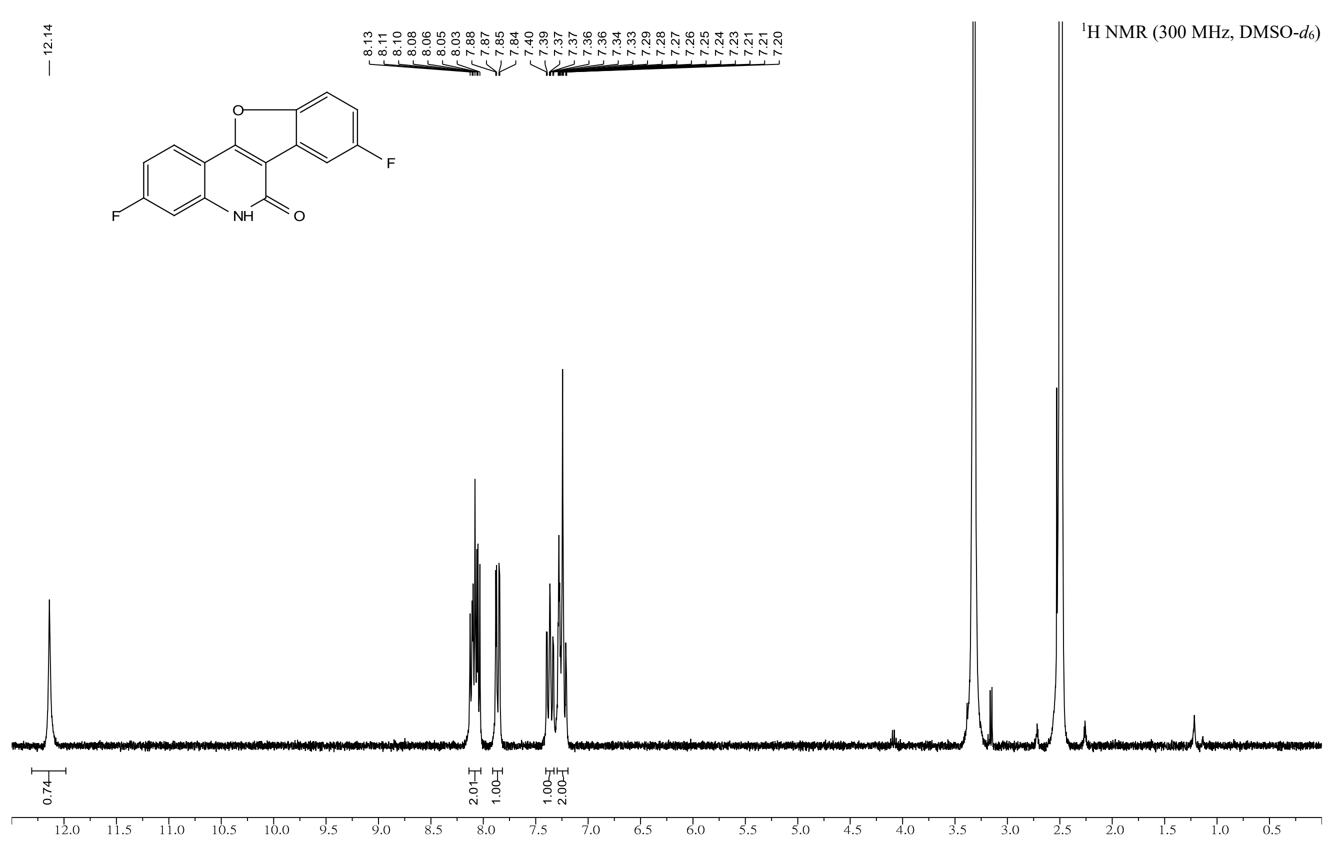

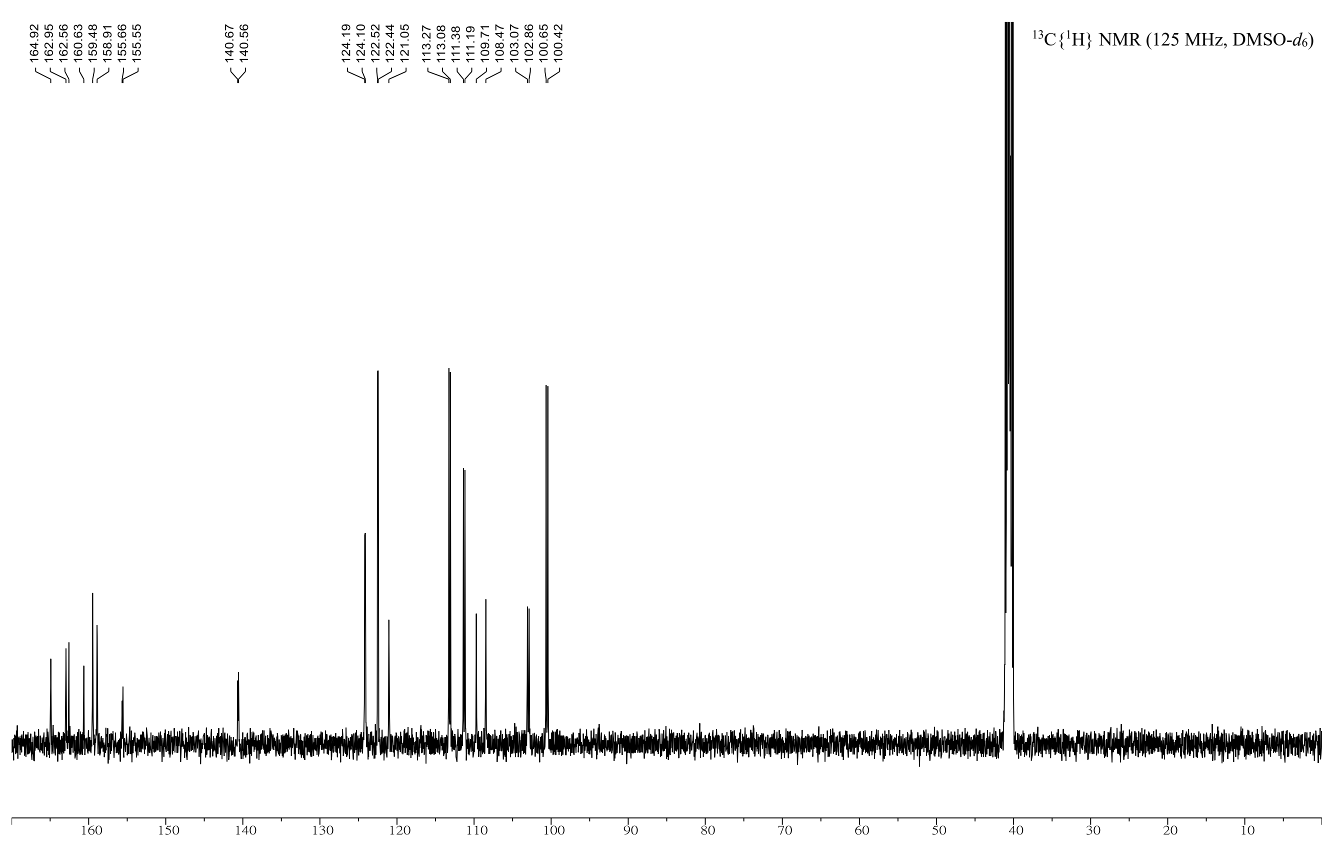

8-Chloro-3-fluorobenzofuro[3,2-*c*]quinolin-6(5*H*)-one (1y)

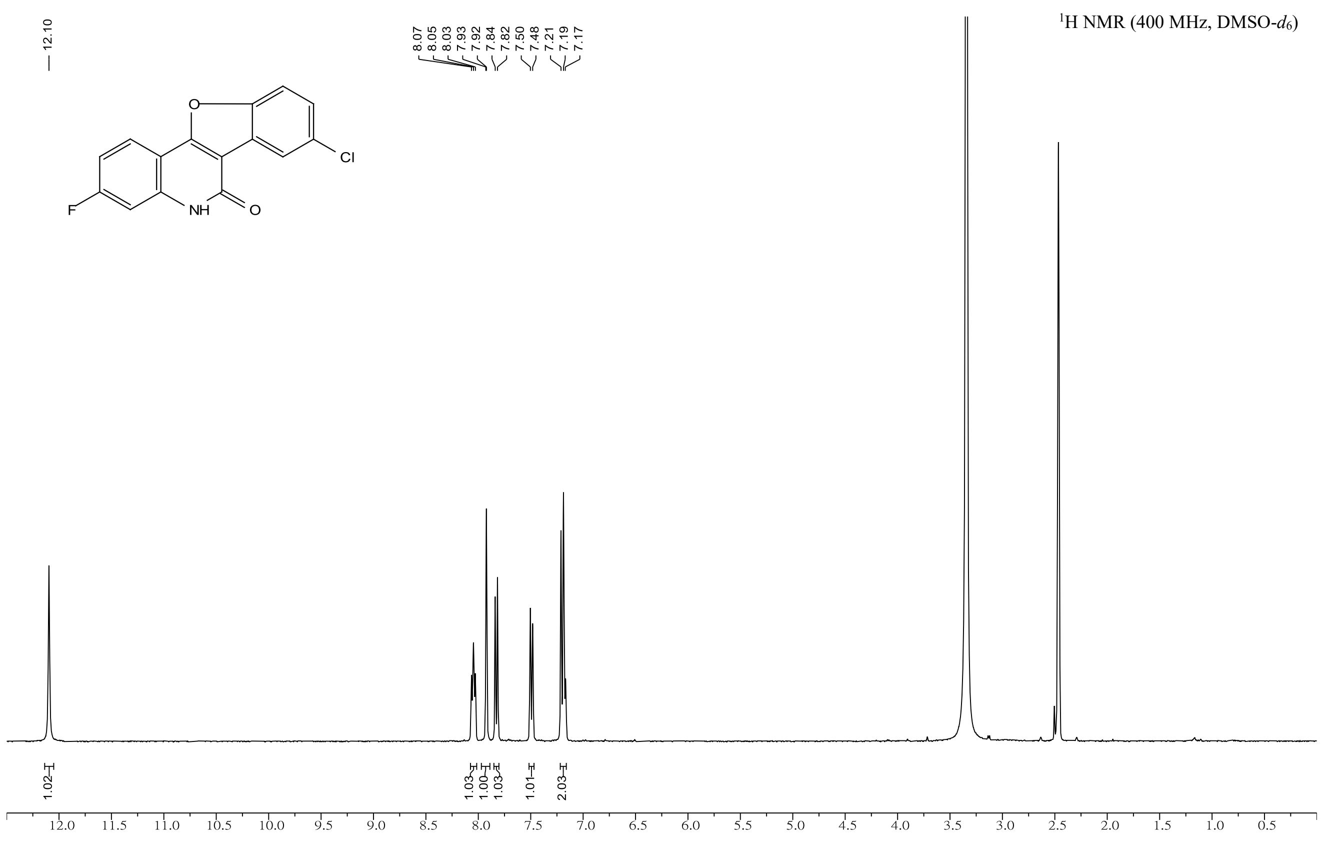

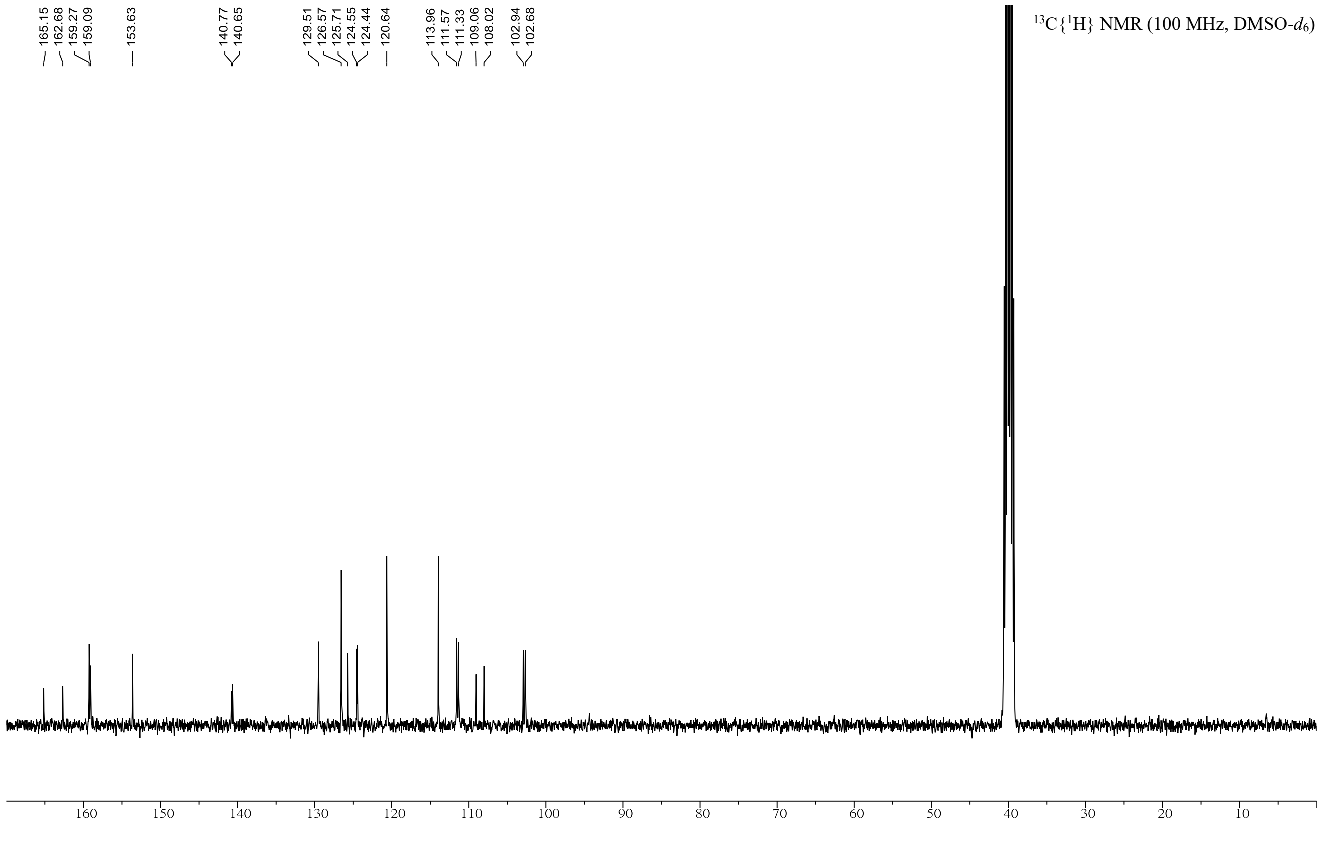

3,8-Dichlorobenzofuro[3,2-*c*]quinolin-6(5*H*)-one (1z)

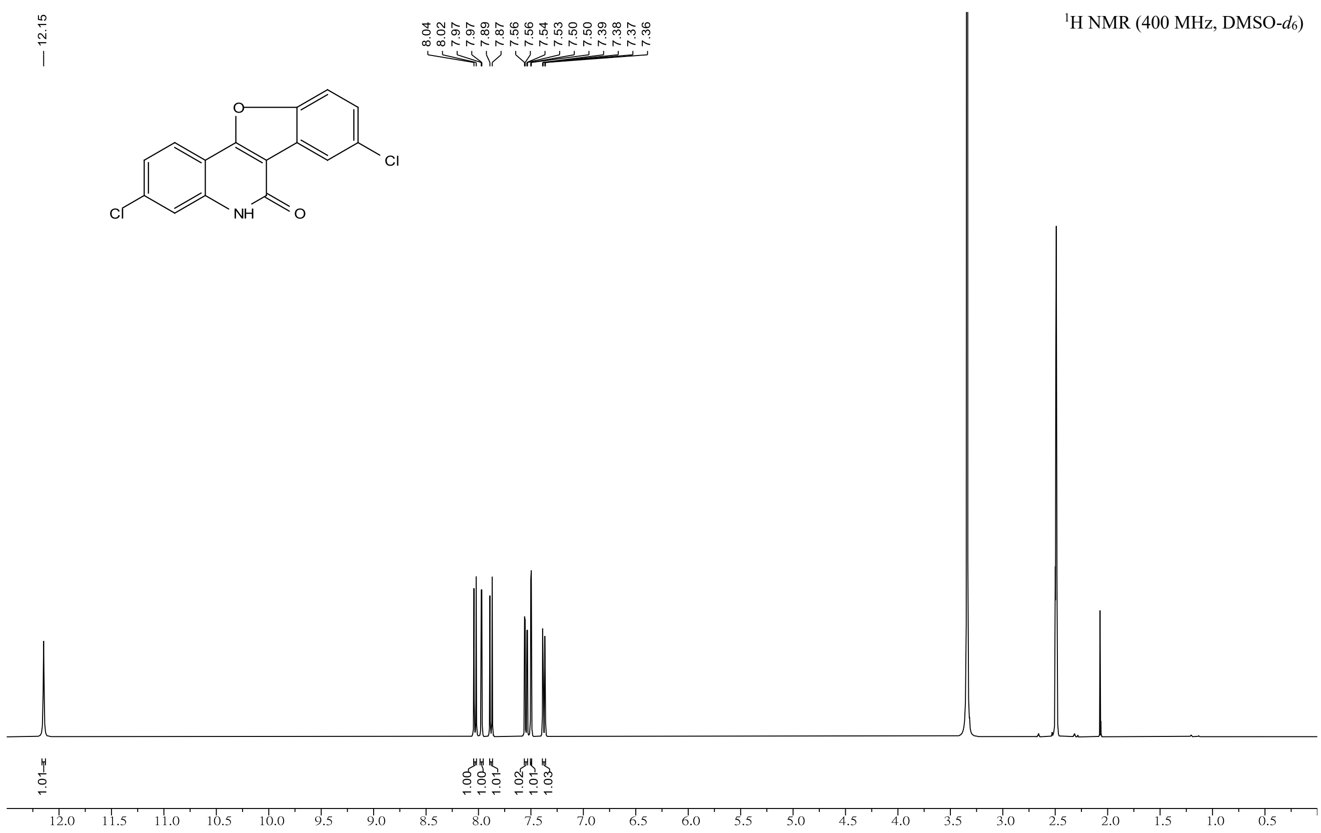

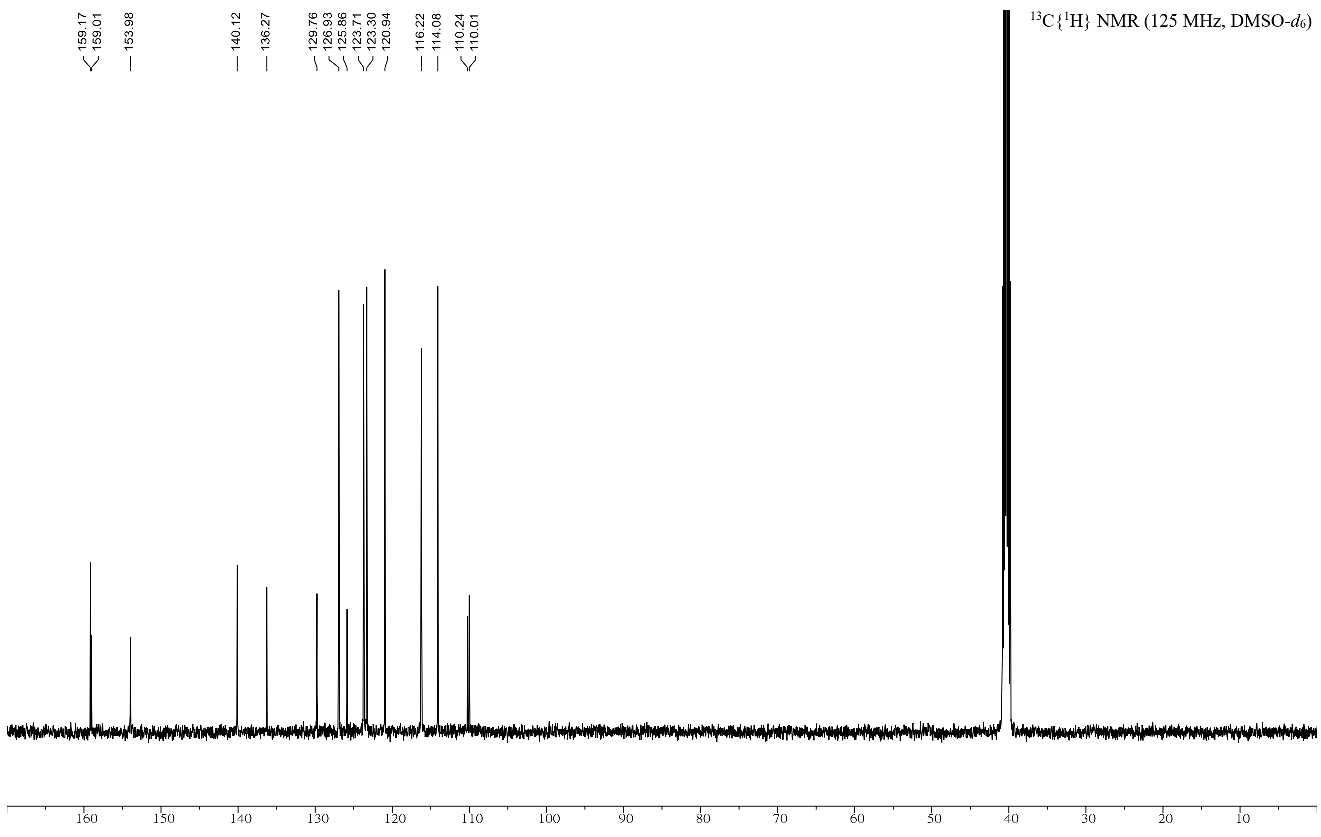

3-Chloro-8,9-dimethoxybenzofuro[3,2-*c*]quinolin-6(5*H*)-one (1A)

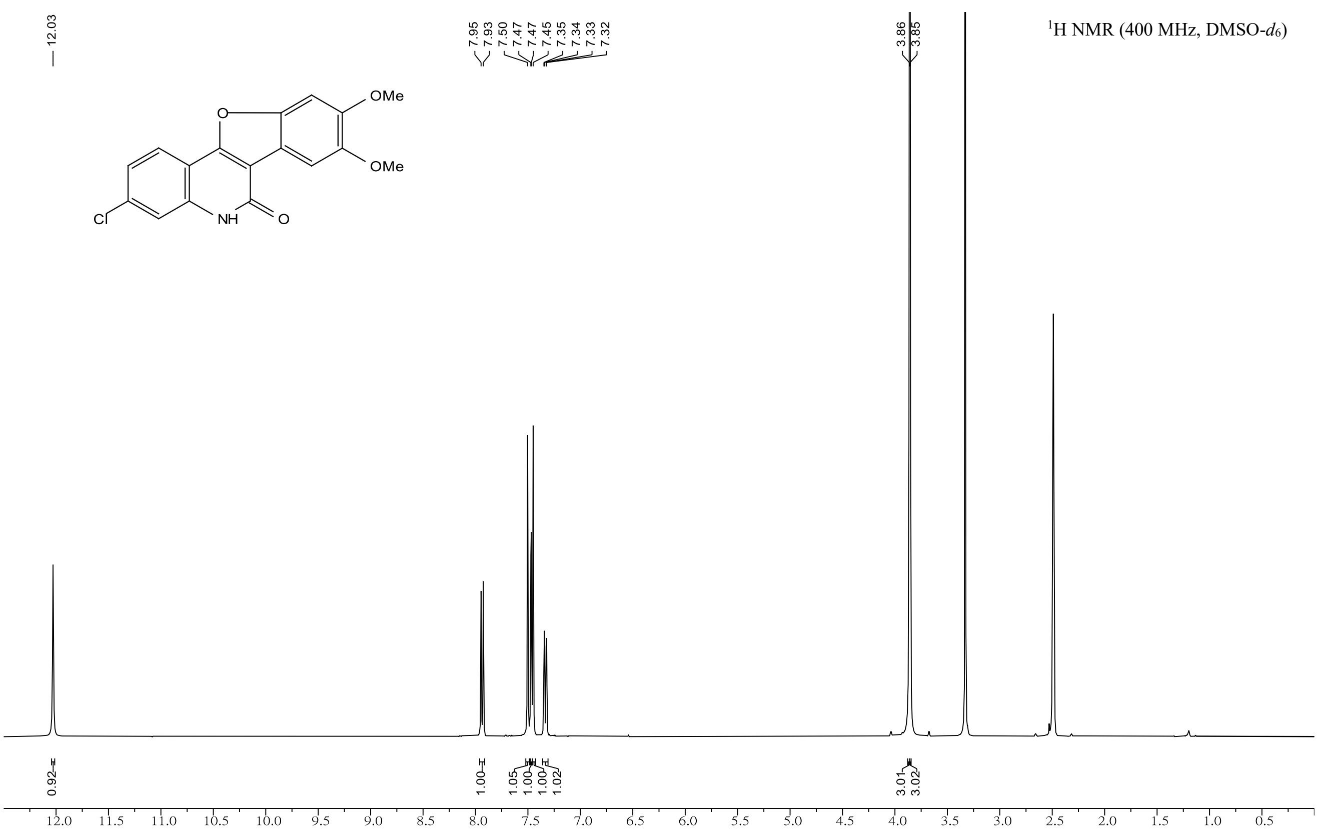

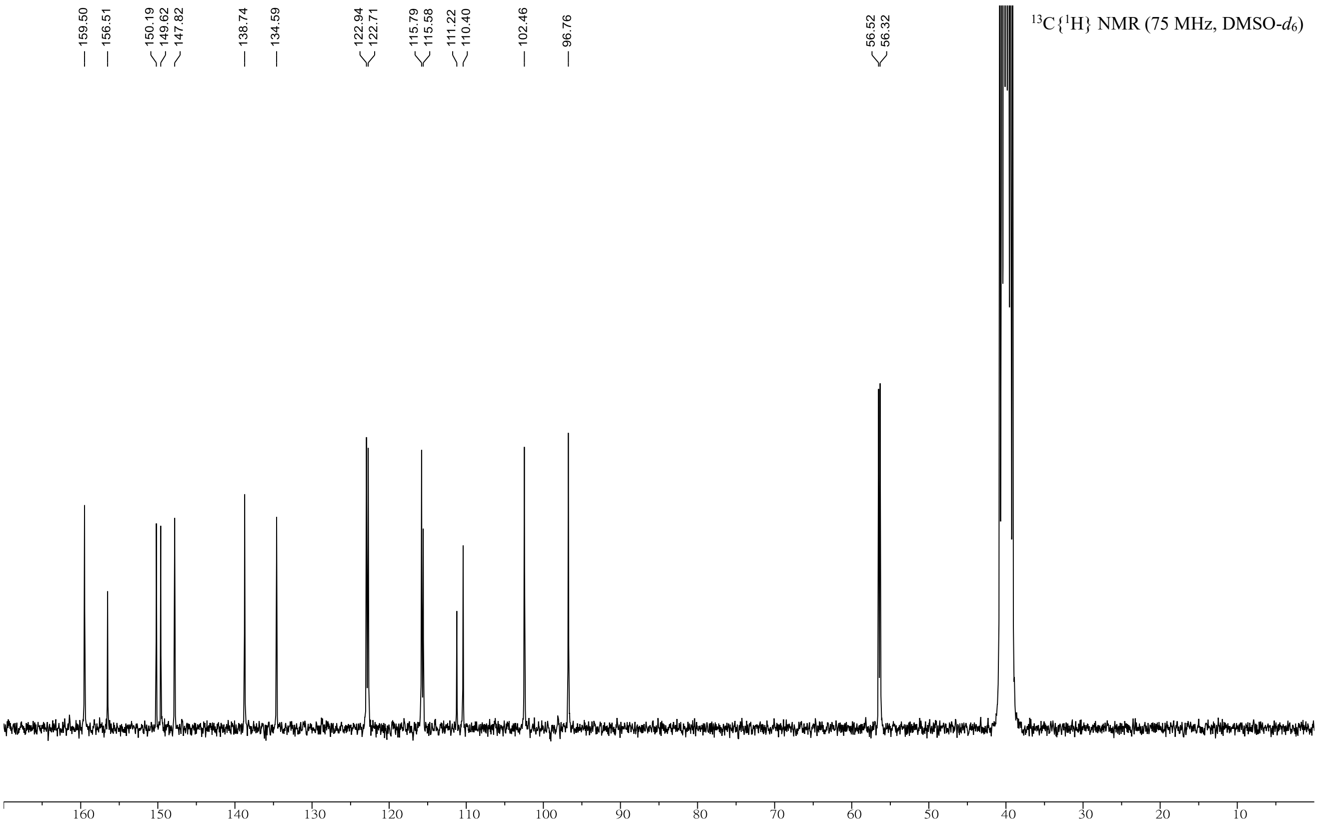

3-Chloro[1,3]dioxolo[4',5':5,6]benzofuro[3,2-*c*]quinolin-6(5*H*)-one (1B)

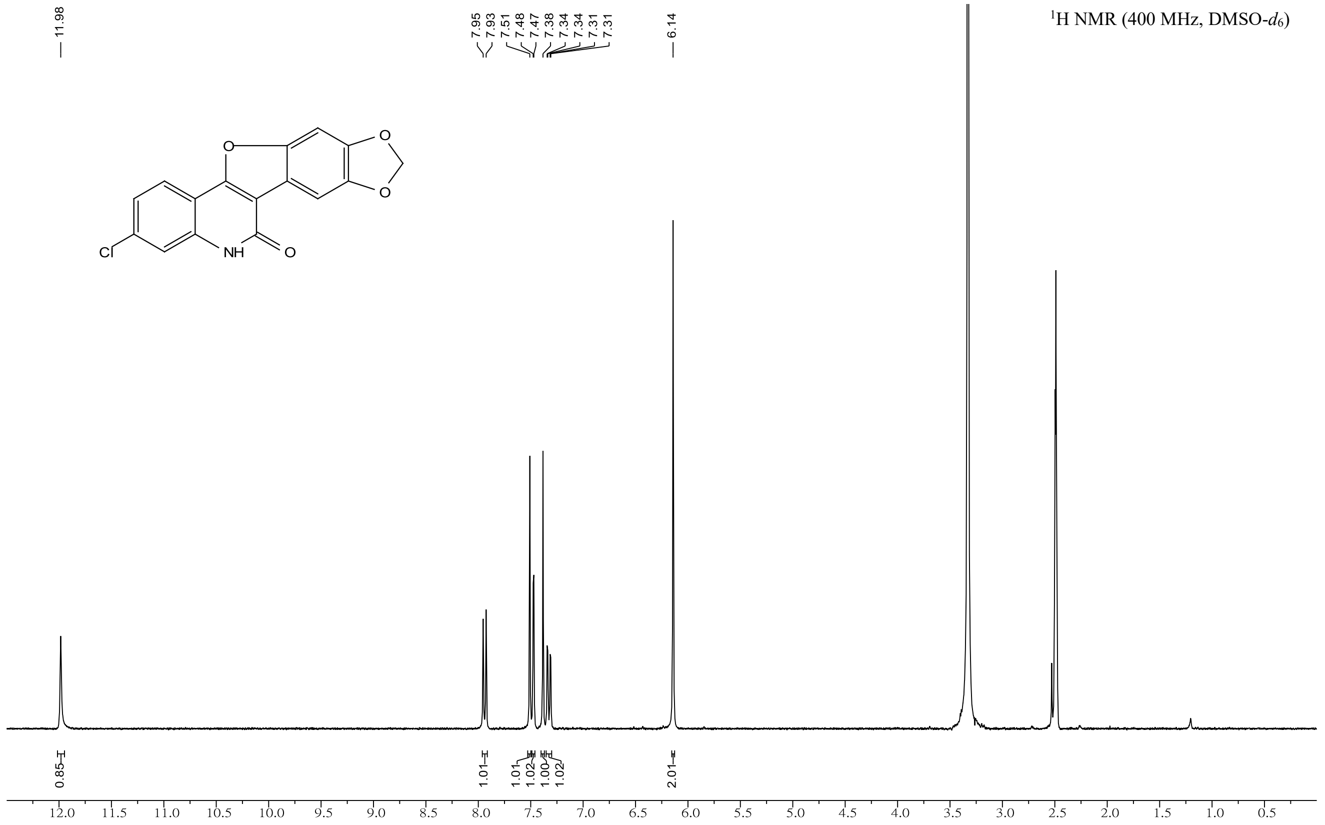

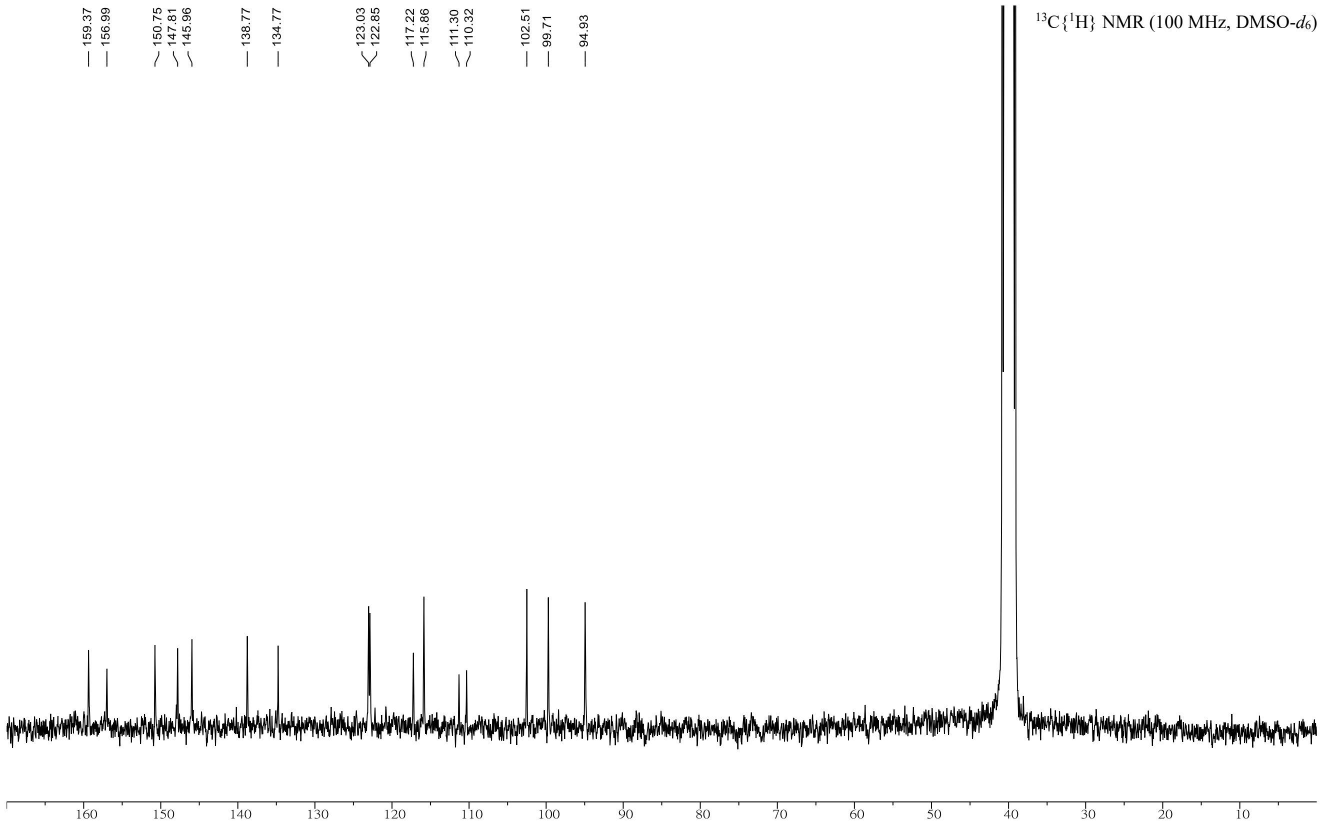

2,9-Dibromobenzofuro[3,2-*c*]quinolin-6(5*H*)-one (1C)

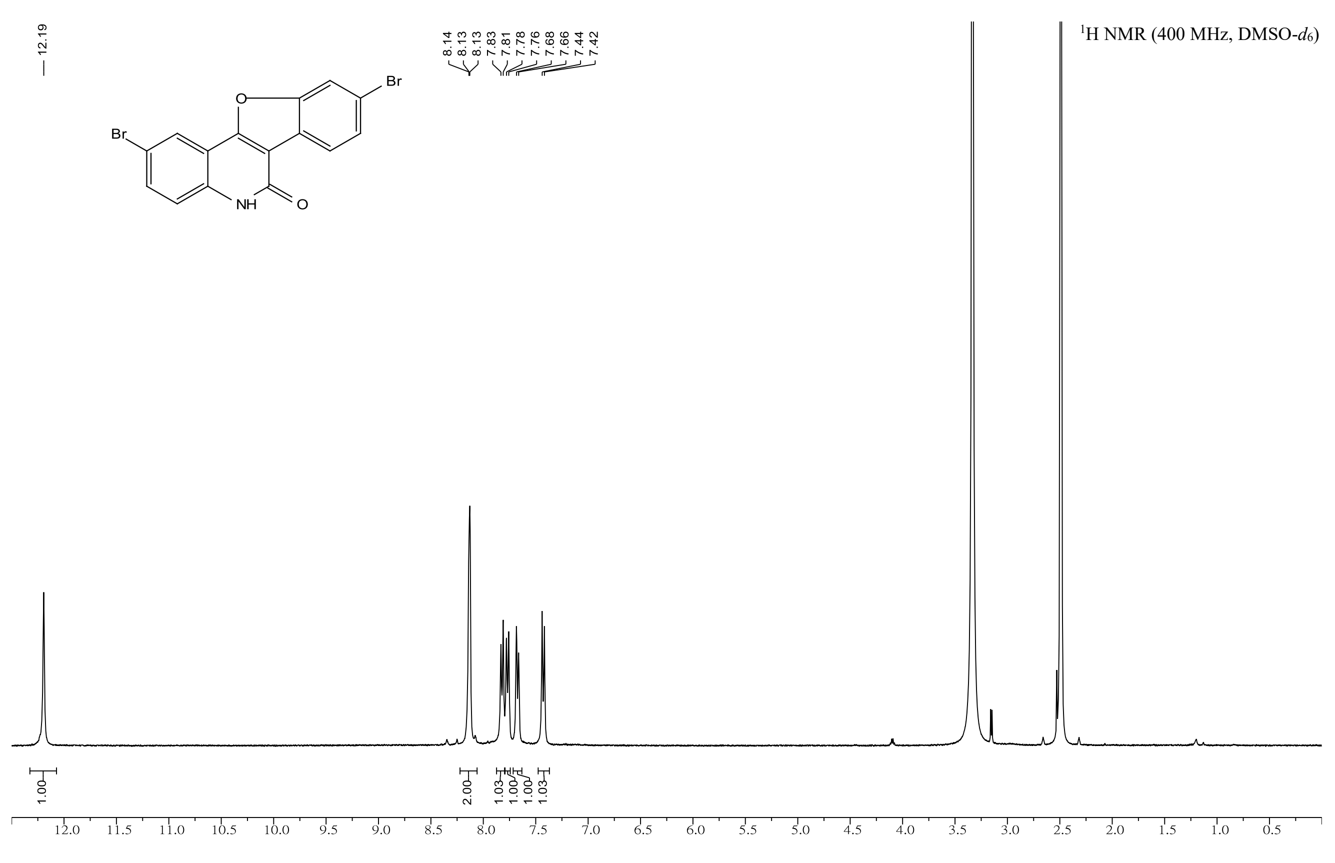

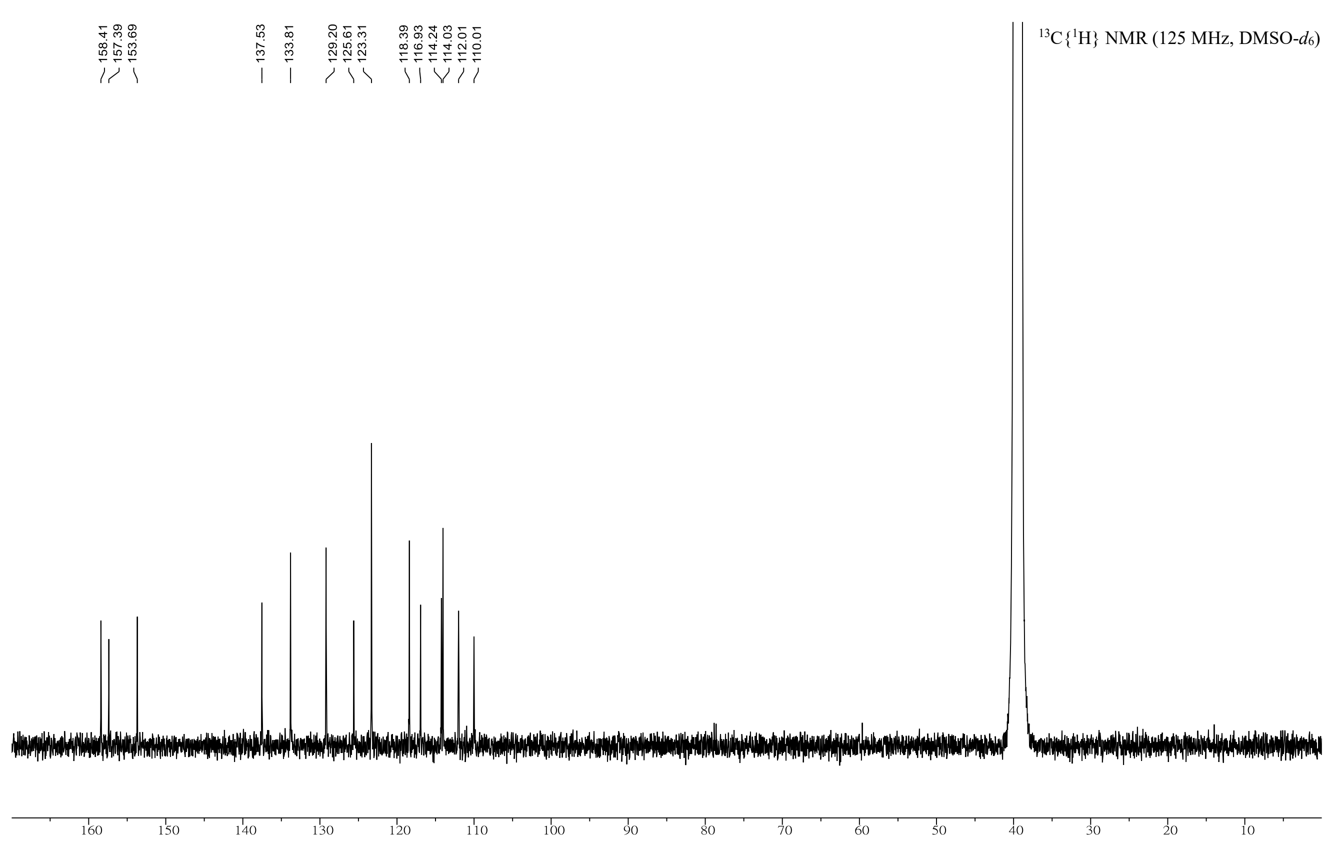

8-Chloro-2-methoxybenzofuro[3,2-*c*]quinolin-6(5*H*)-one (1D)

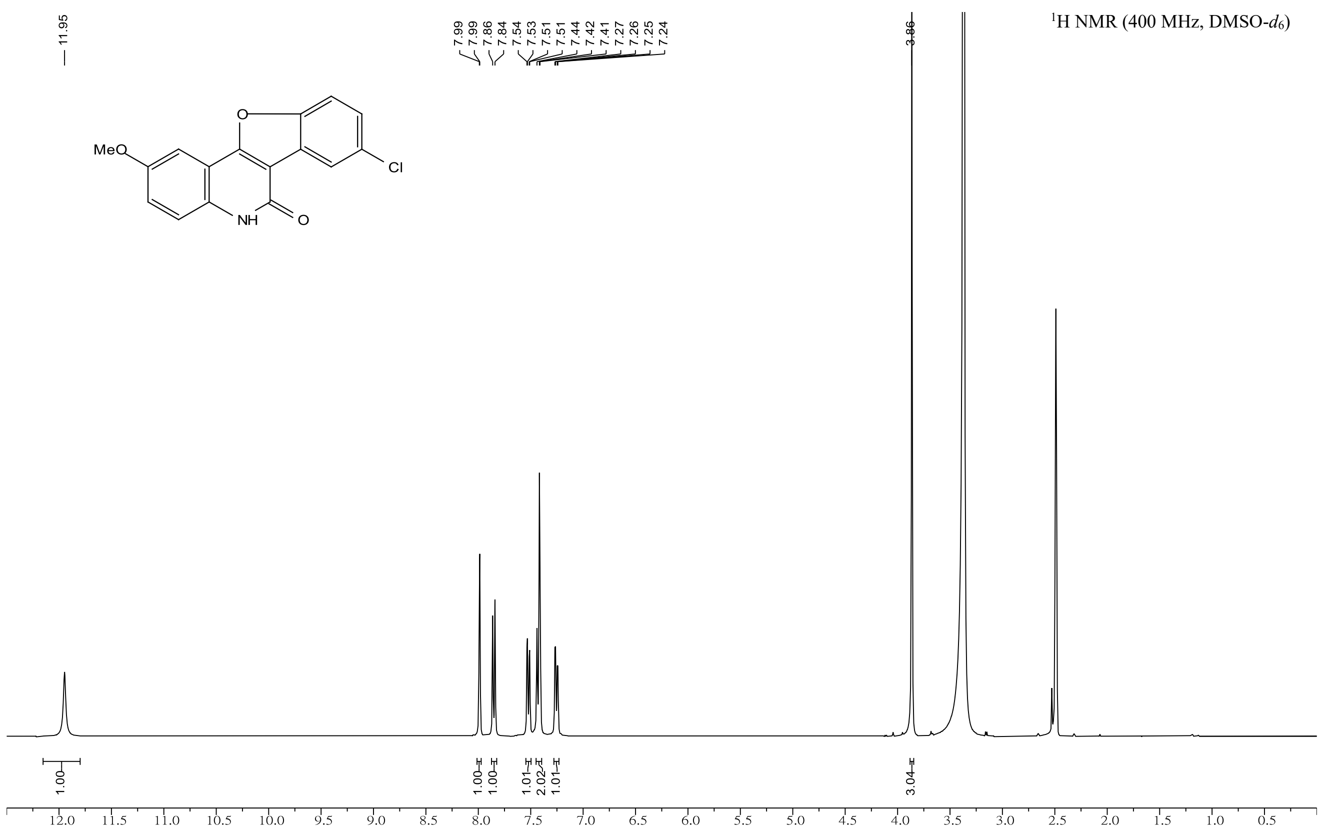

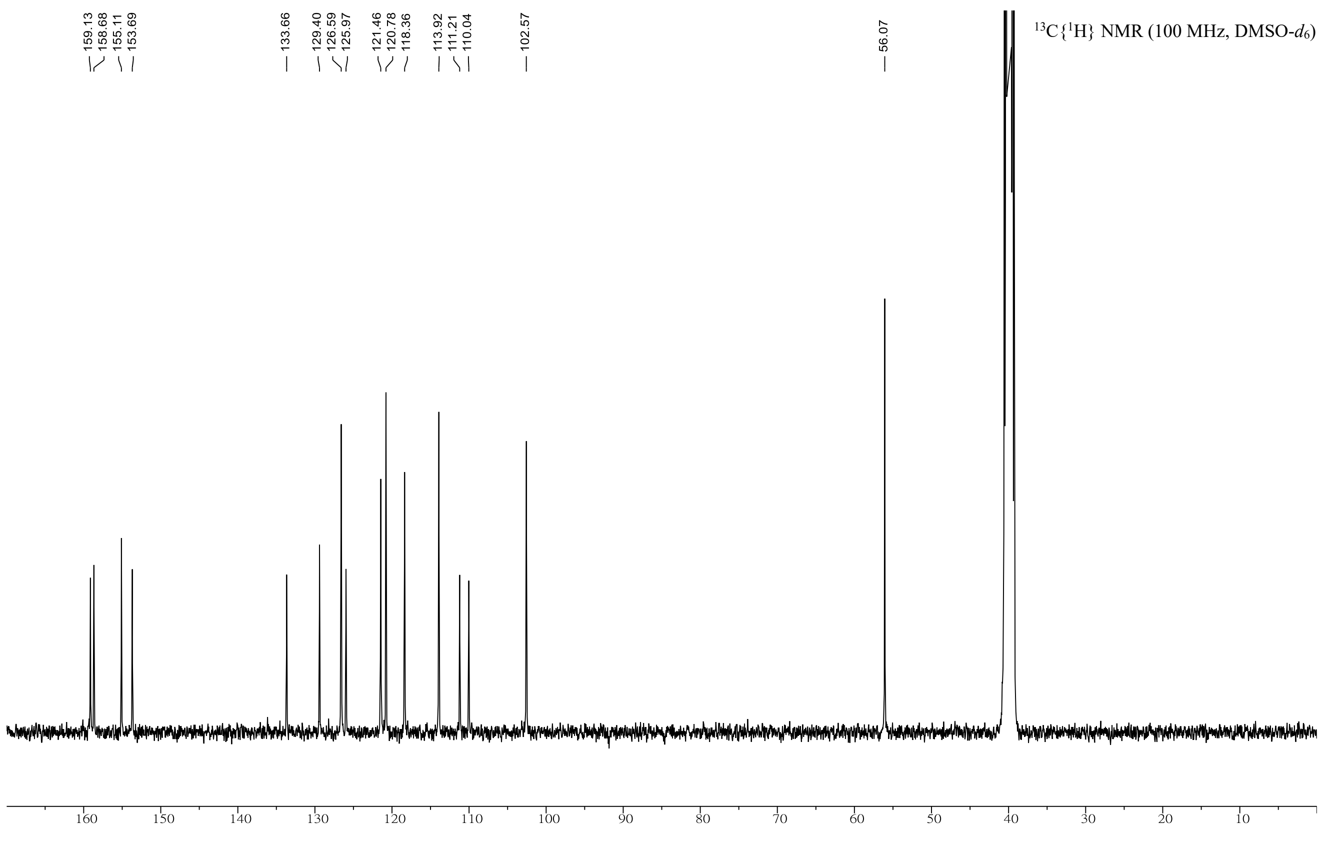

2,3-Dimethoxy[1,3]dioxolo[4',5':5,6]benzofuro[3,2-*c*]quinolin-6(5*H*)-one (1E)

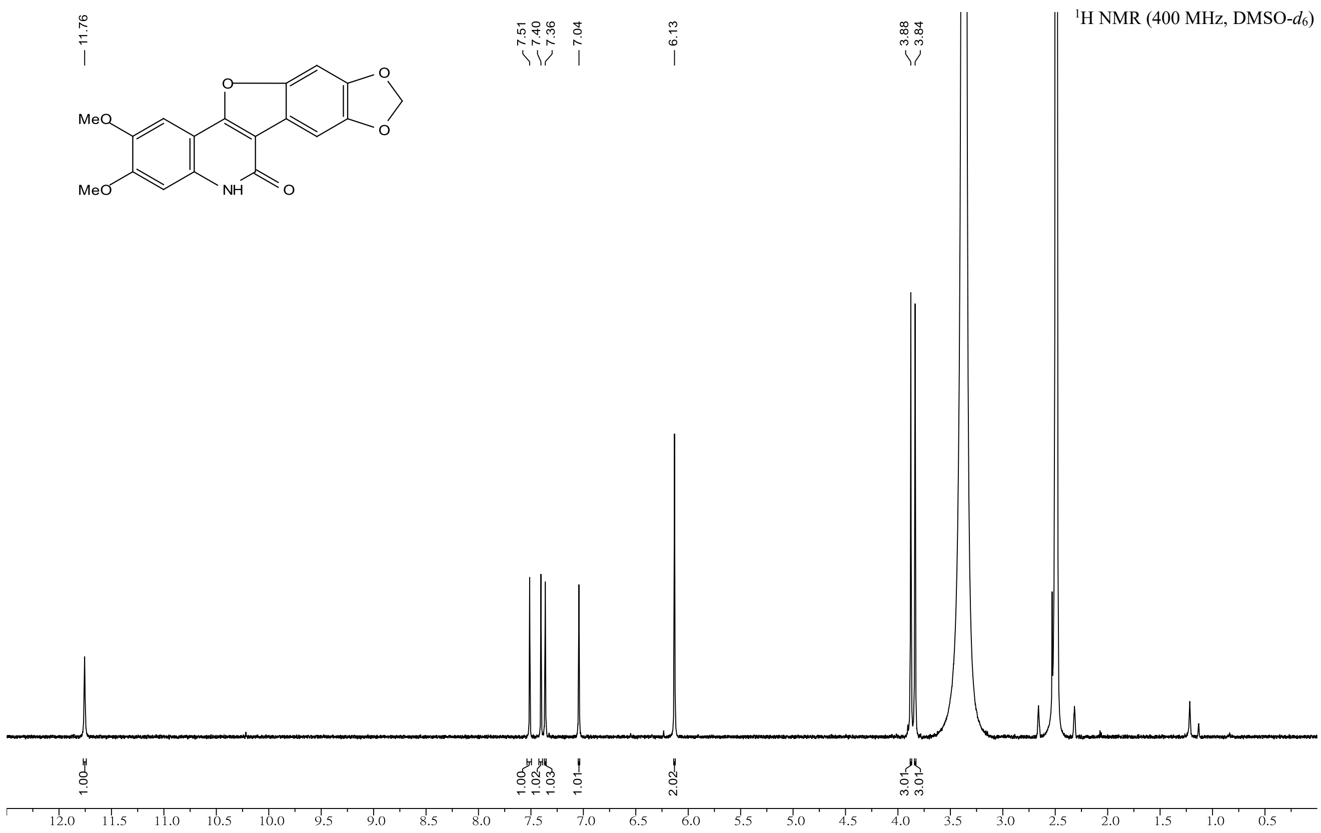

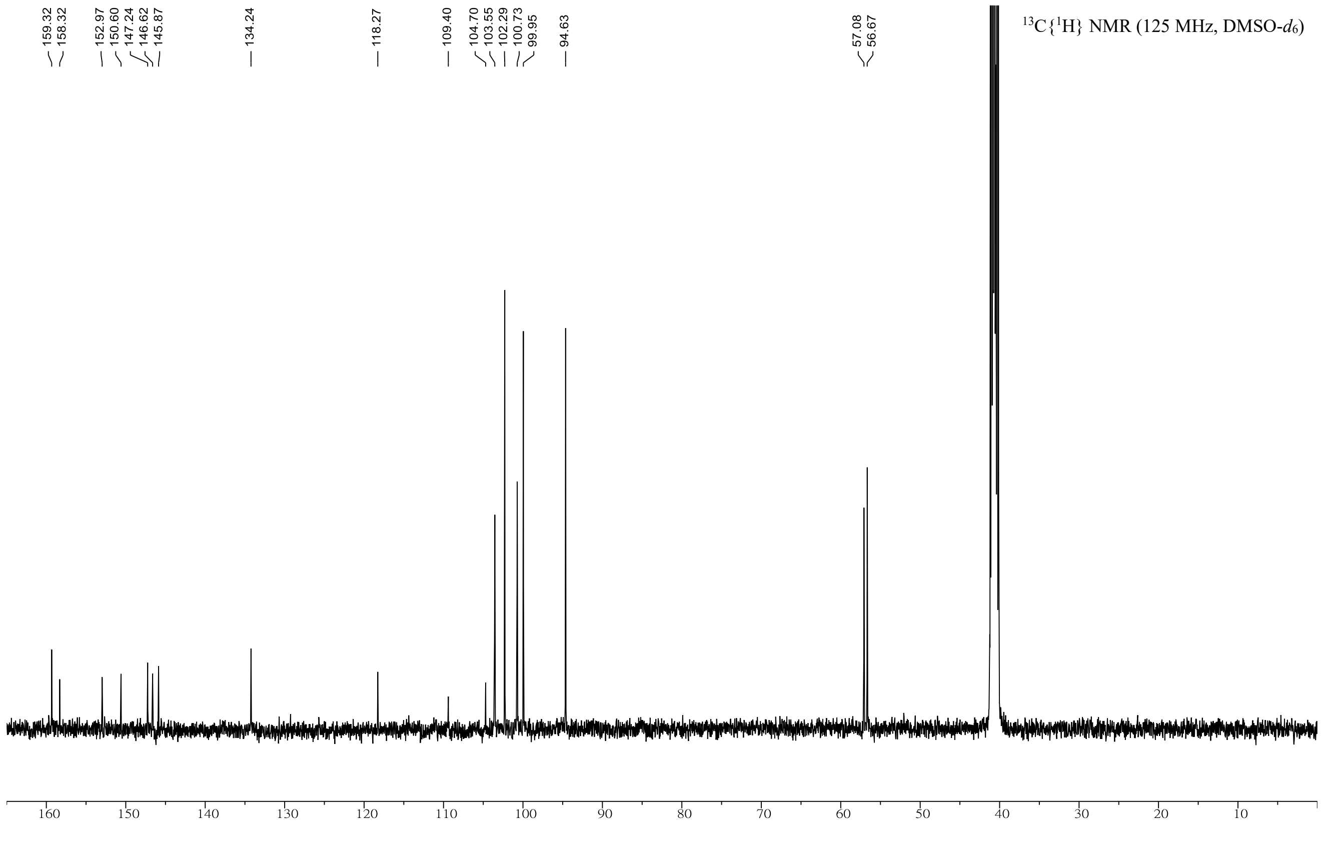

3-(2-Bromophenyl)-4-hydroxyquinolin-2(1*H*)-one (7a)

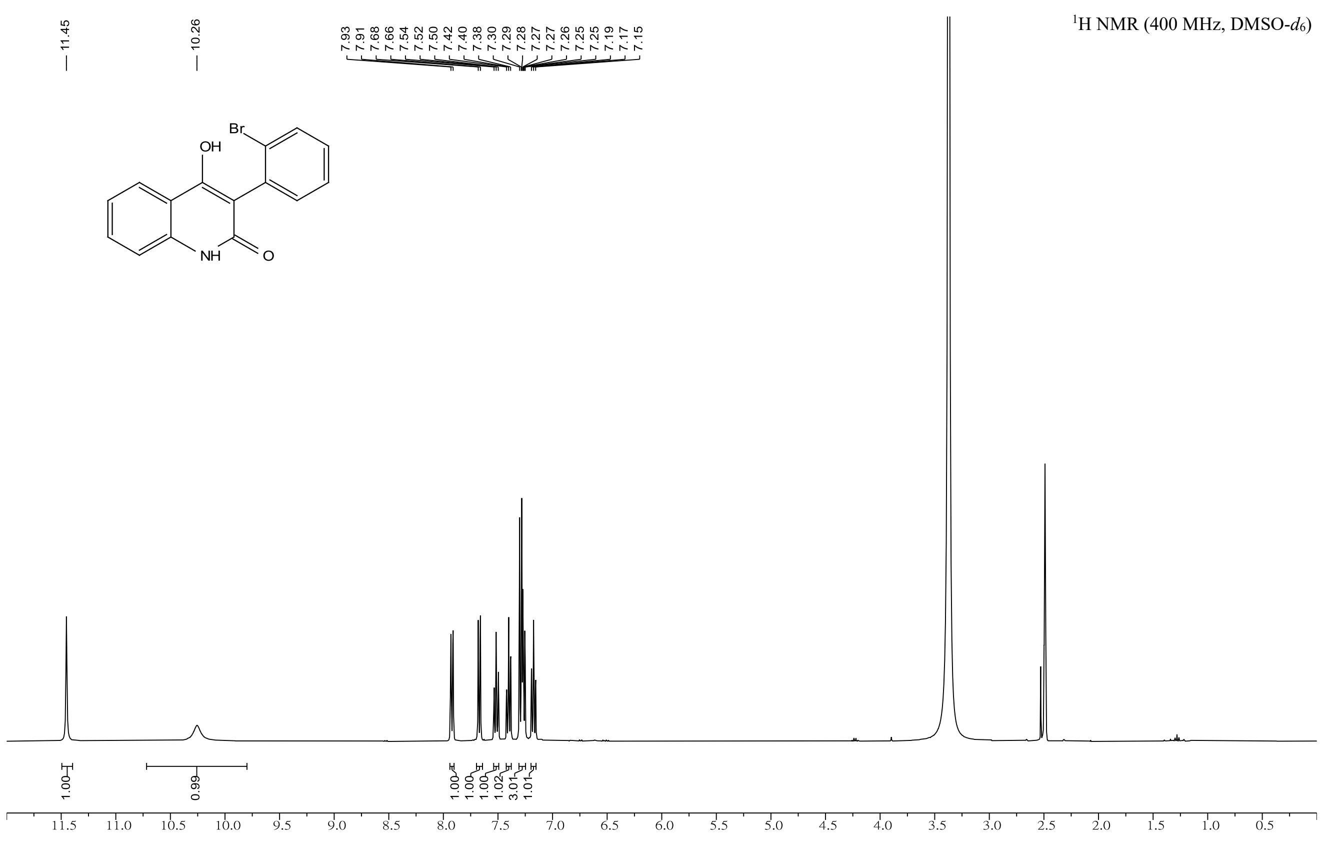

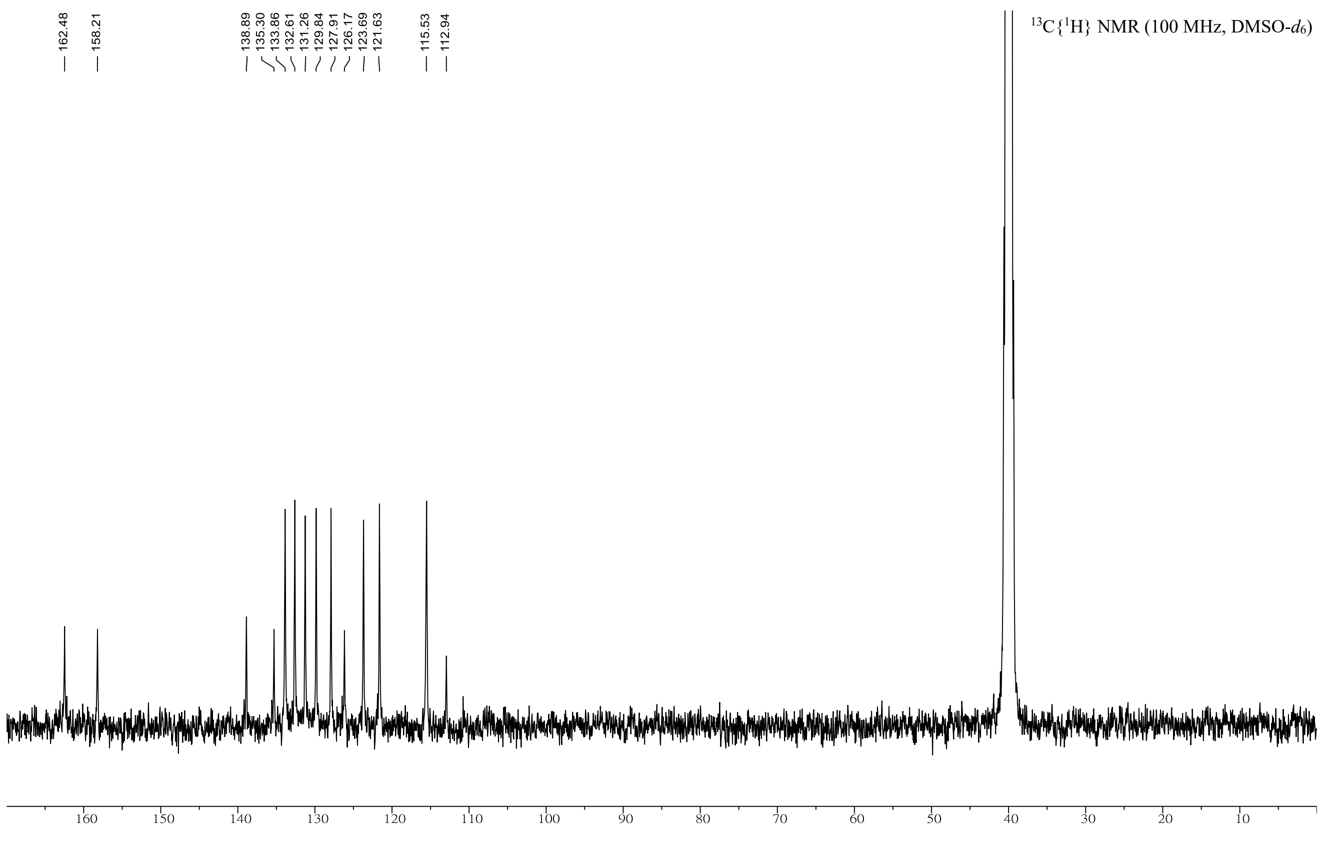

2,9-Dibromo-5-methylbenzofuro[3,2-*c*]quinolin-6(5*H*)-one (10)

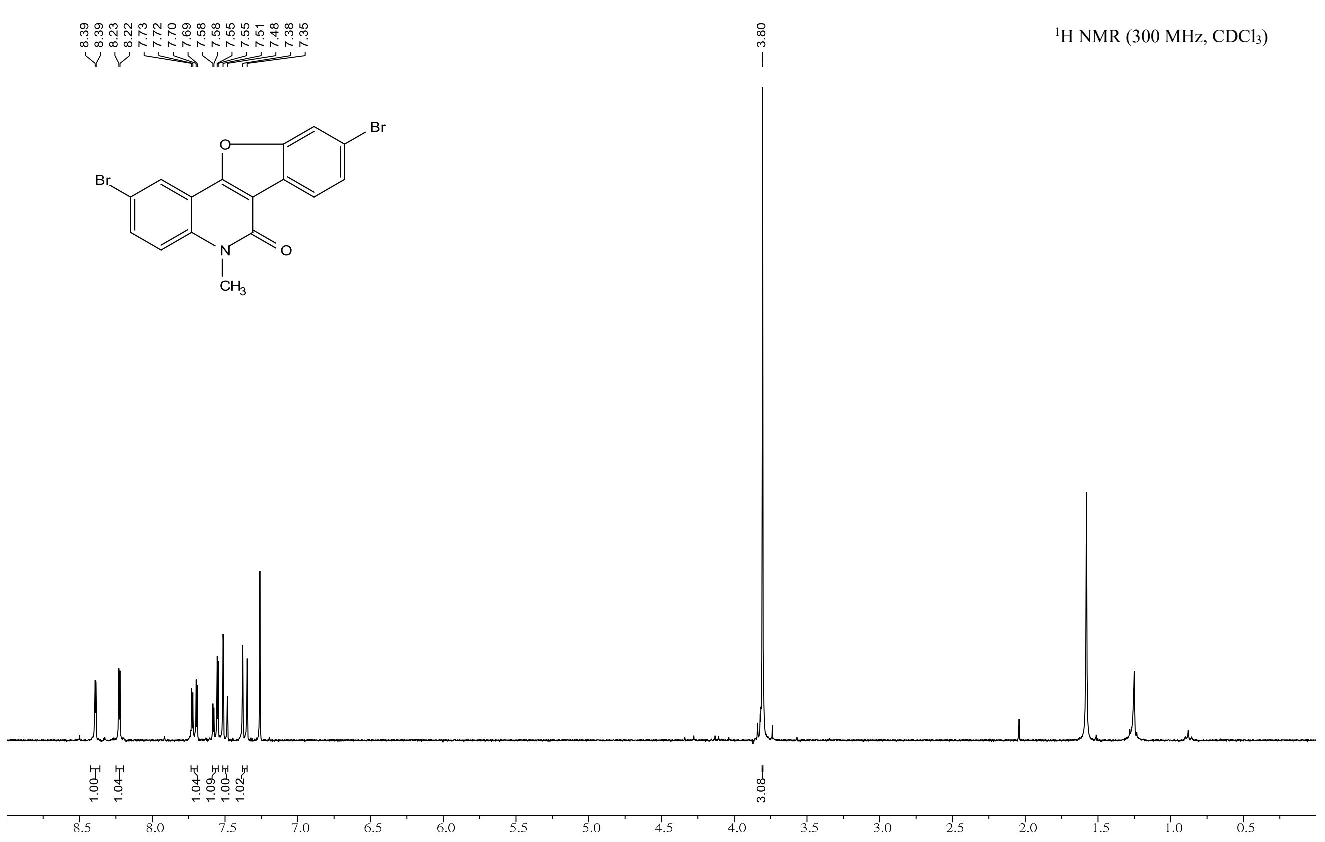

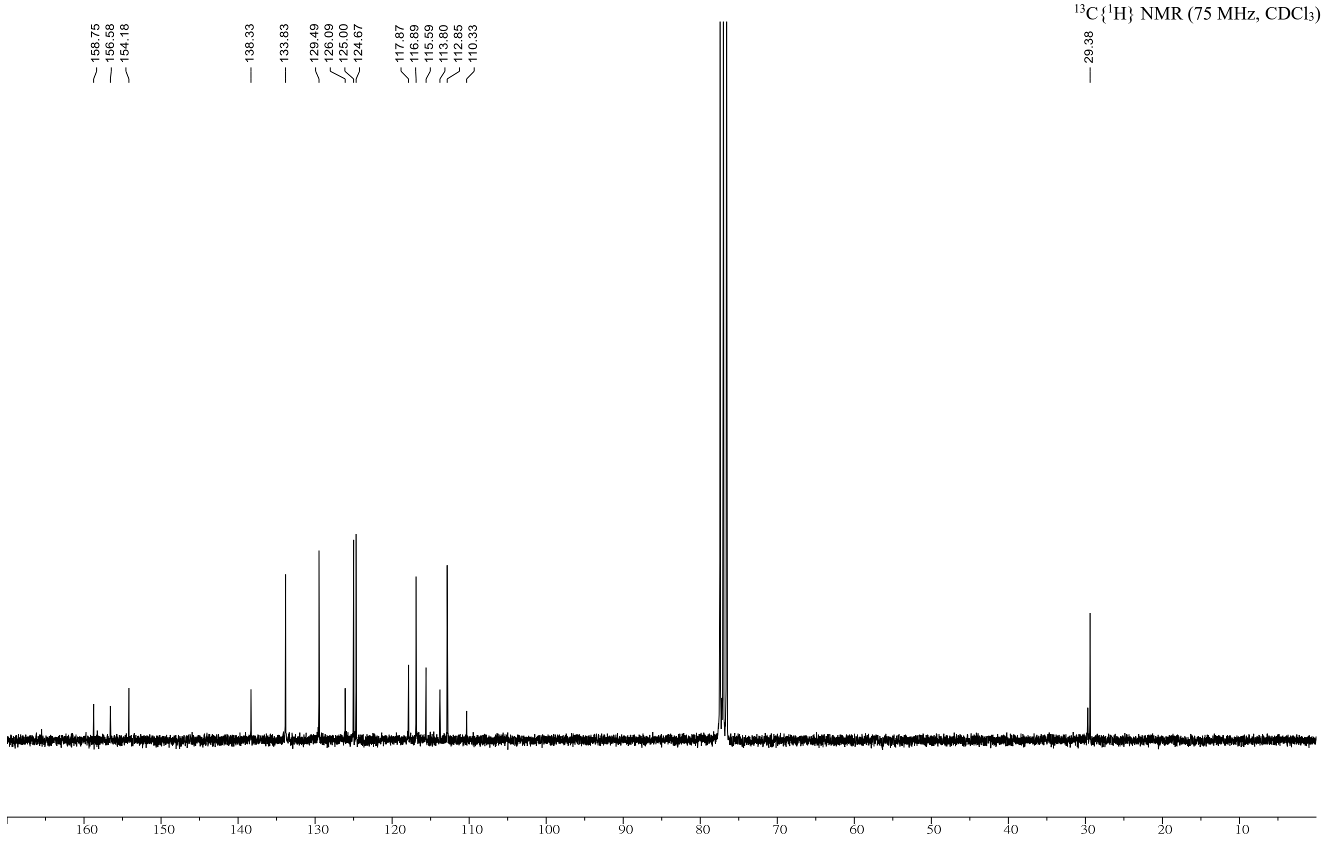

2,9-Bis{[2-(dimethylamino)ethyl]amino}-5-methylbenzofuro[3,2-*c*]quinolin-6(5*H*)-one (8a)

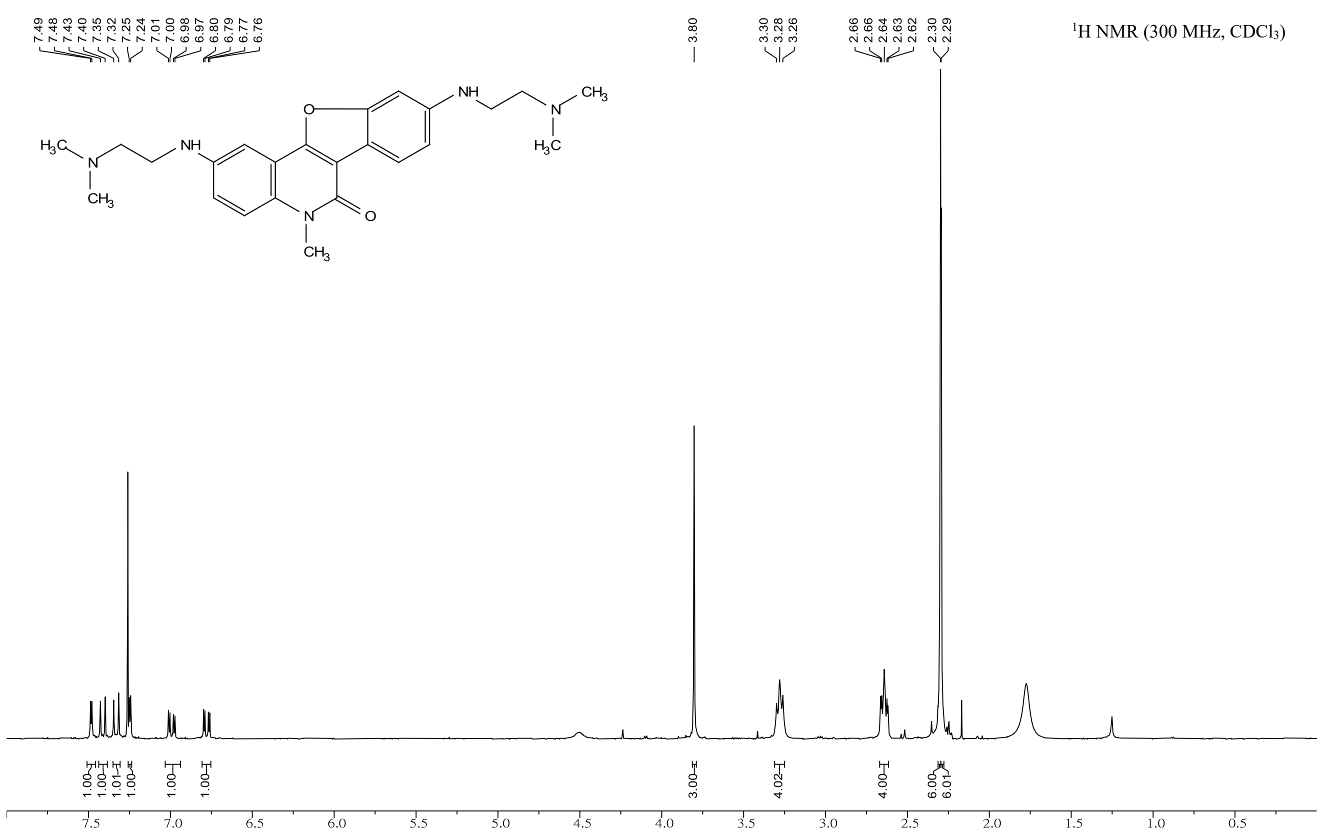

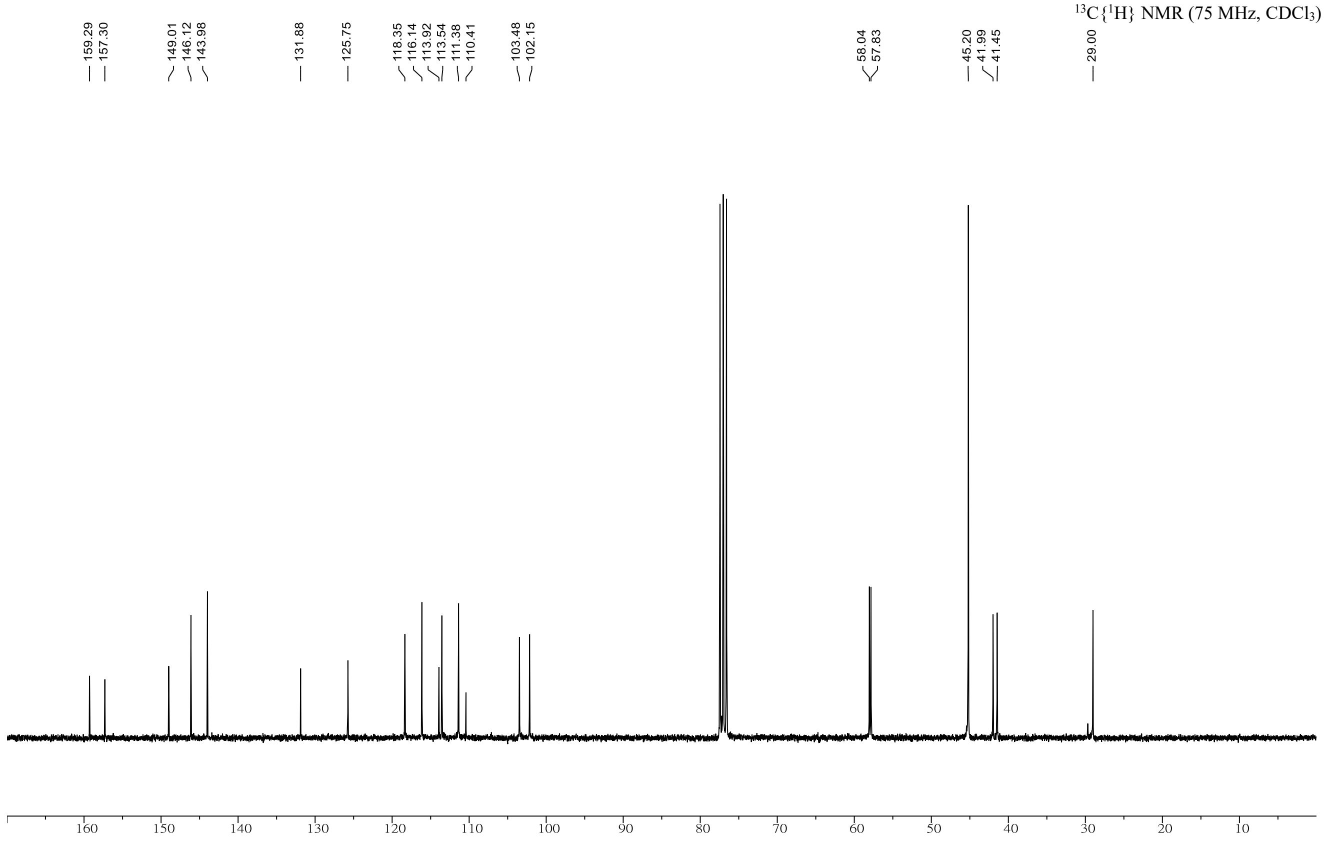

2,9-Bis{[2-(diethylamino)ethyl]amino}-5-methylbenzofuro[3,2-*c*]quinolin-6(5*H*)-one (8b)

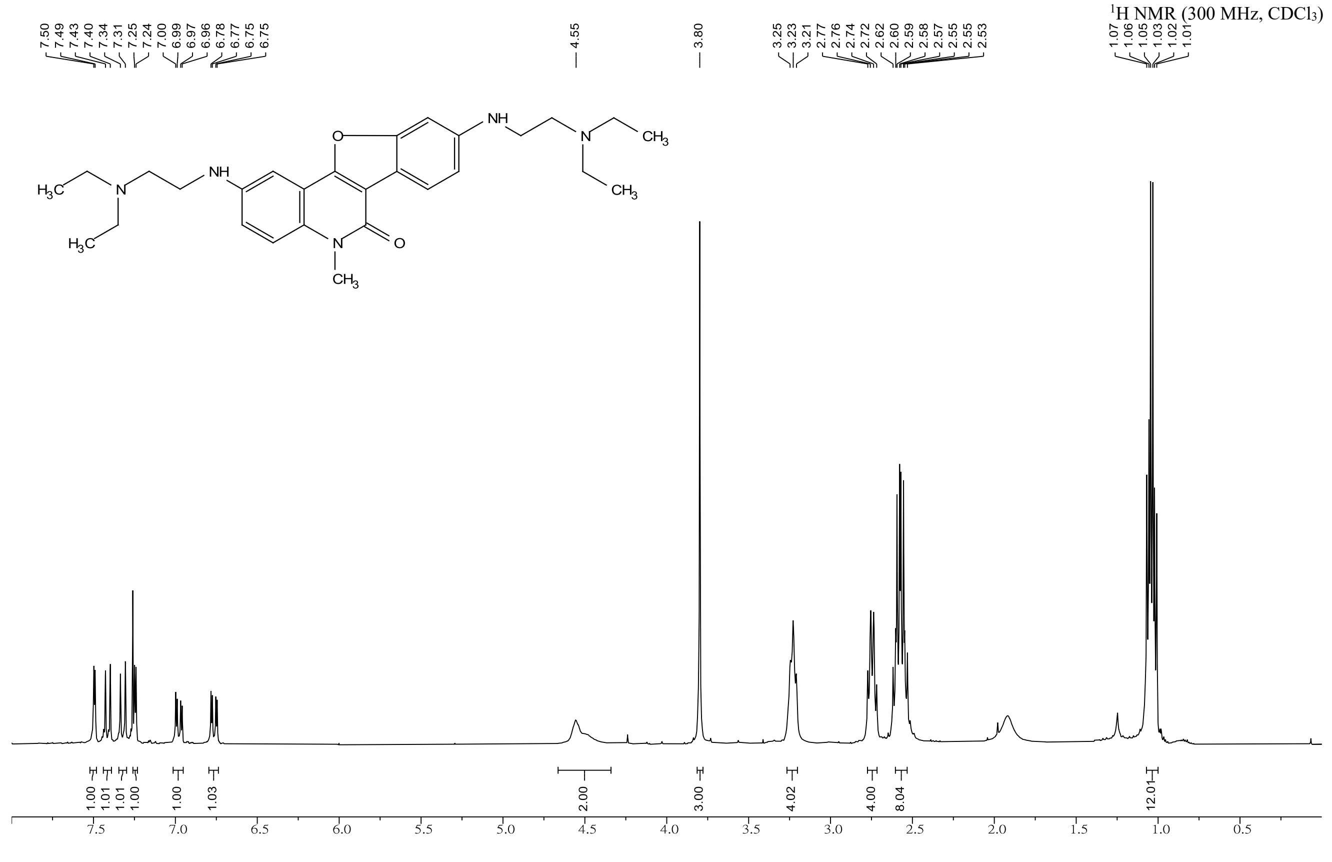

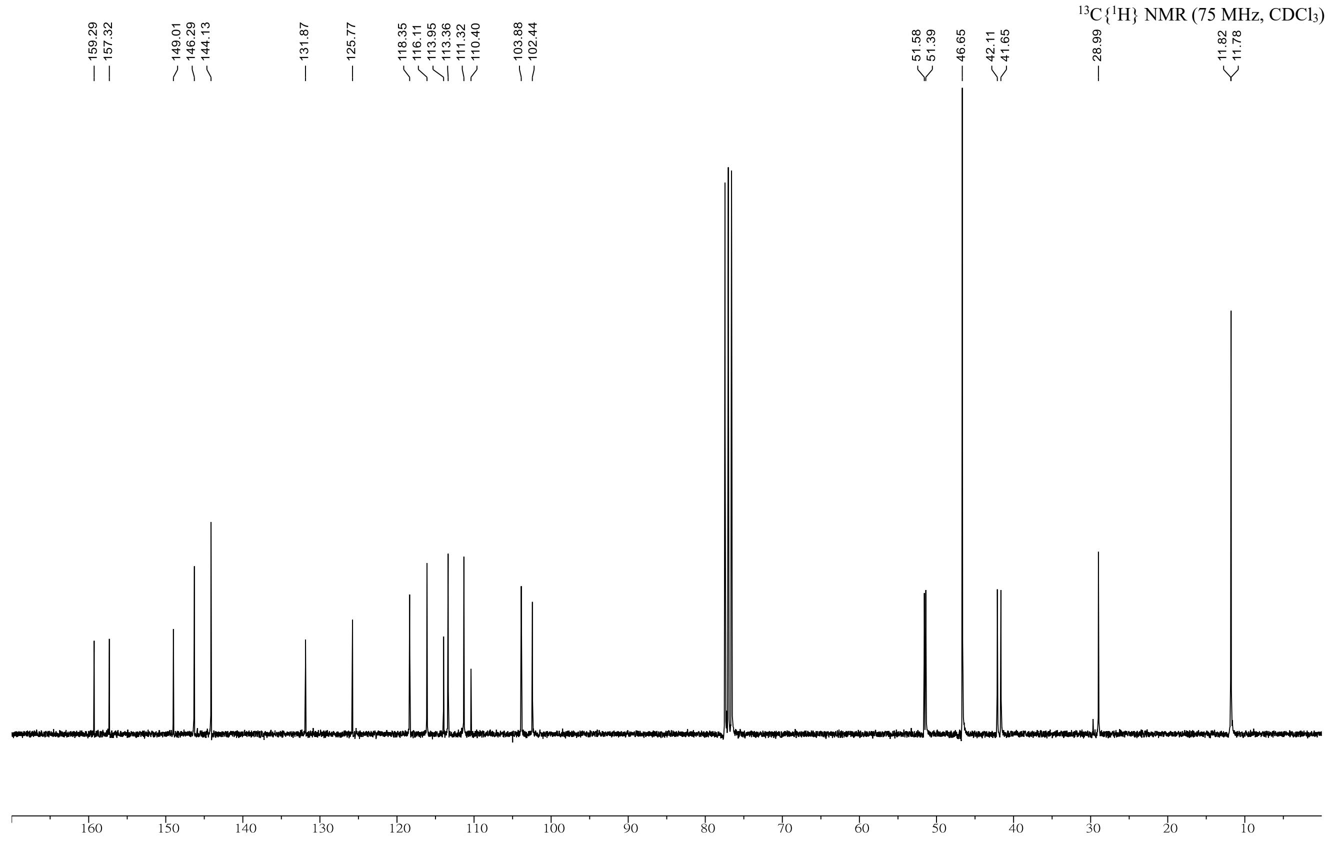

2,9-Bis{[3-(dimethylamino)propyl]amino}-5-methylbenzofuro[3,2-*c*]quinolin-6(5*H*)-one (8c)

^{1}H NMR (300 MHz, $CDCl_3$)

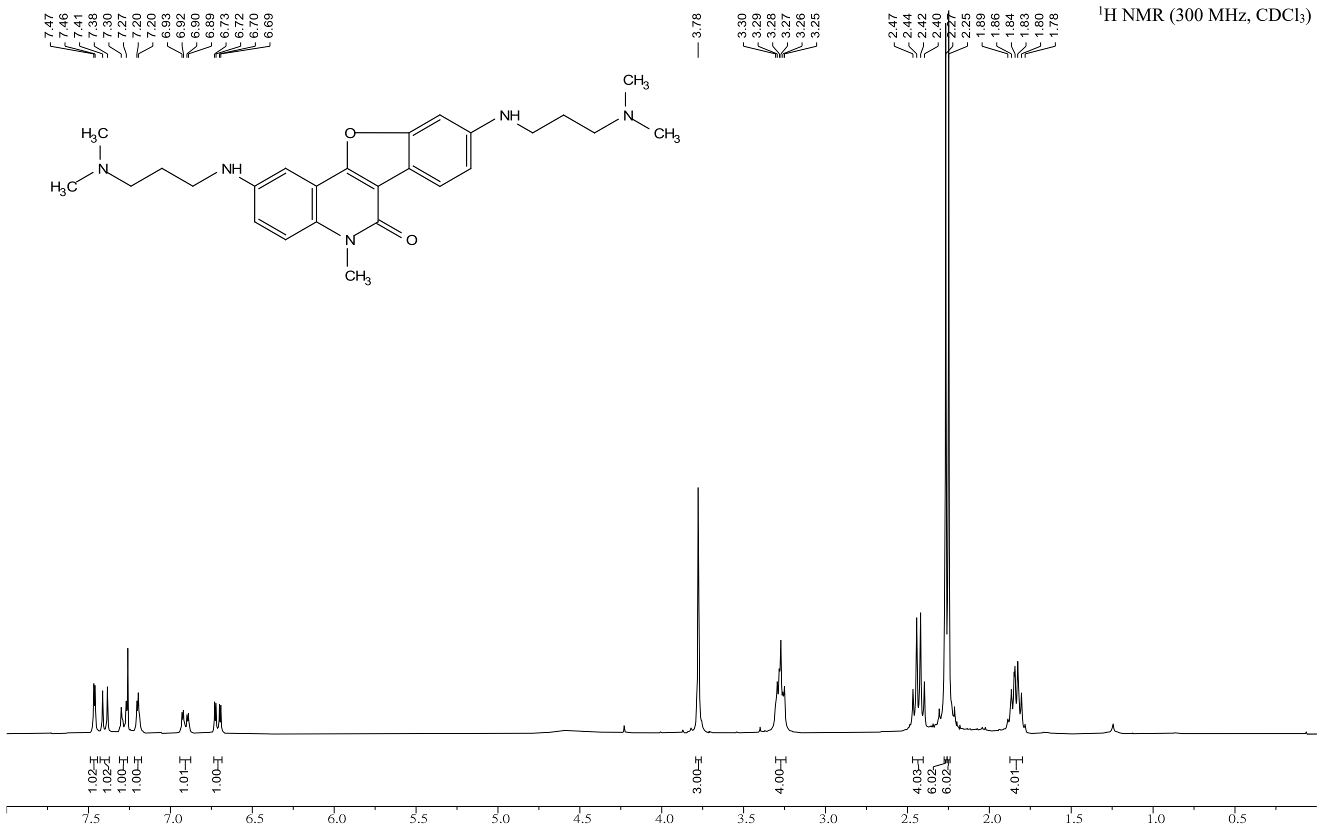

^{13}C{^{1}H} NMR (75 MHz, $CDCl_3$)

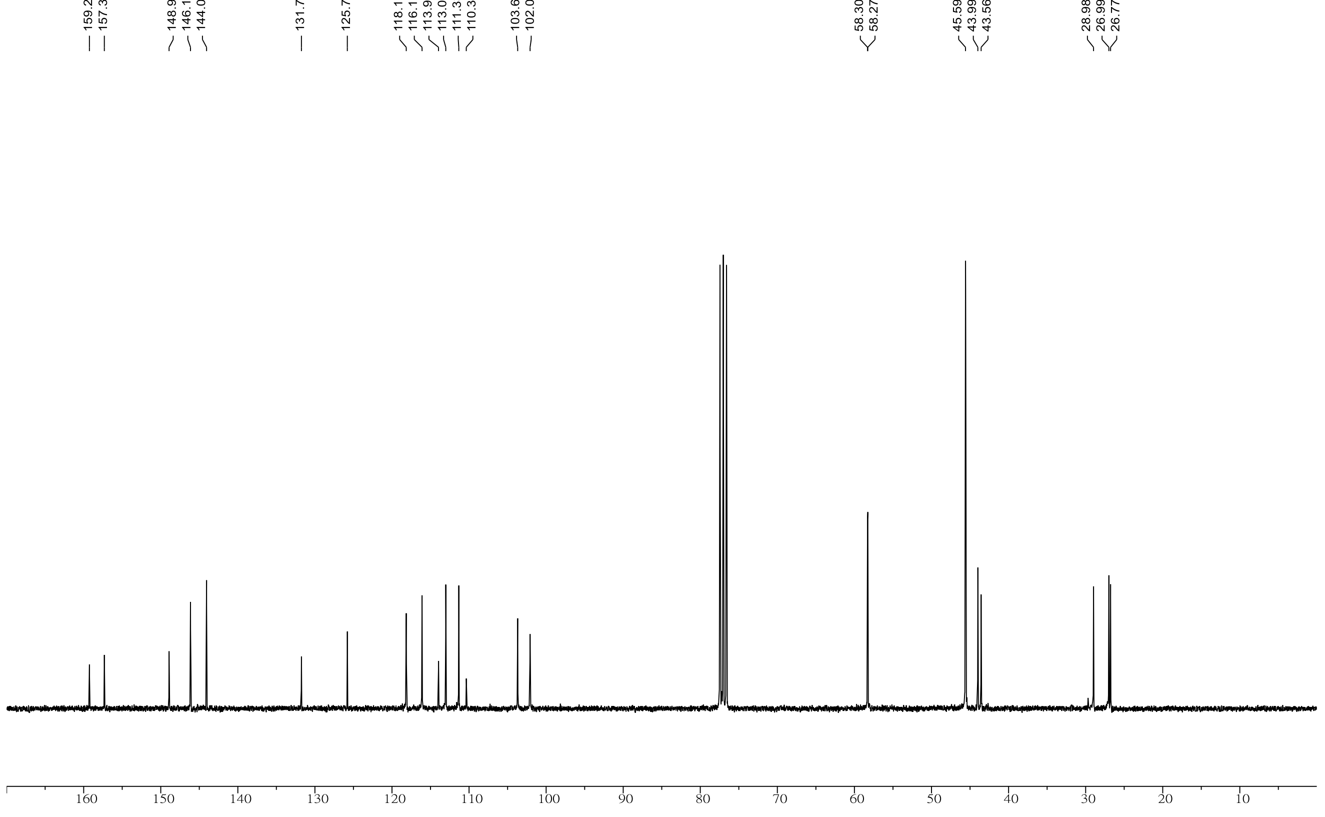

2,9-Bis{[3-(diethylamino)propyl]amino}-5-methylbenzofuro[3,2-*c*]quinolin-6(5*H*)-one (8d)

^{1}H NMR (300 MHz, $CDCl_3$)

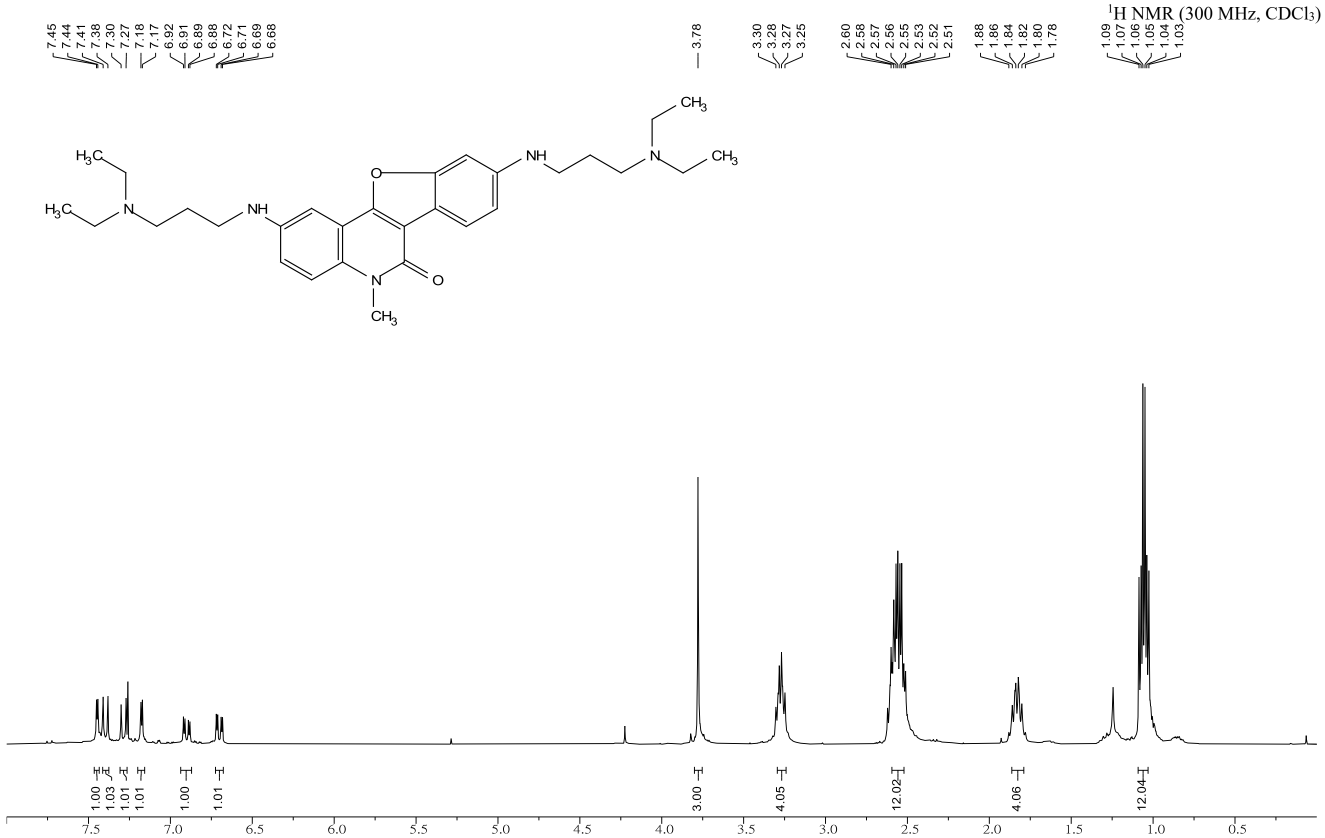

$^{13}C\{^{1}H\}$ NMR (75 MHz, $CDCl_3$)

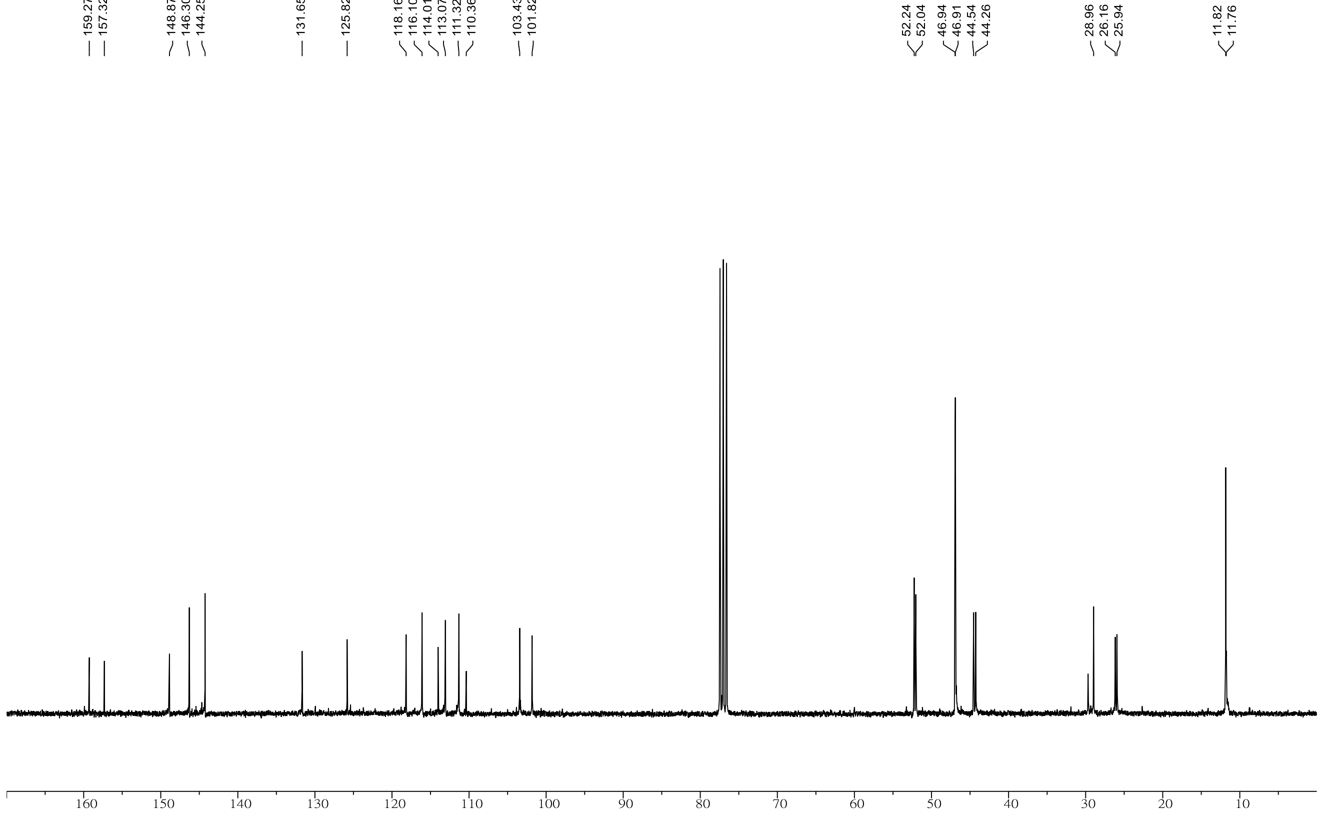

5-Methyl-2,9-bis[(3-morpholinopropyl)amino]benzofuro[3,2-*c*]quinolin-6(5*H*)-one (8e)

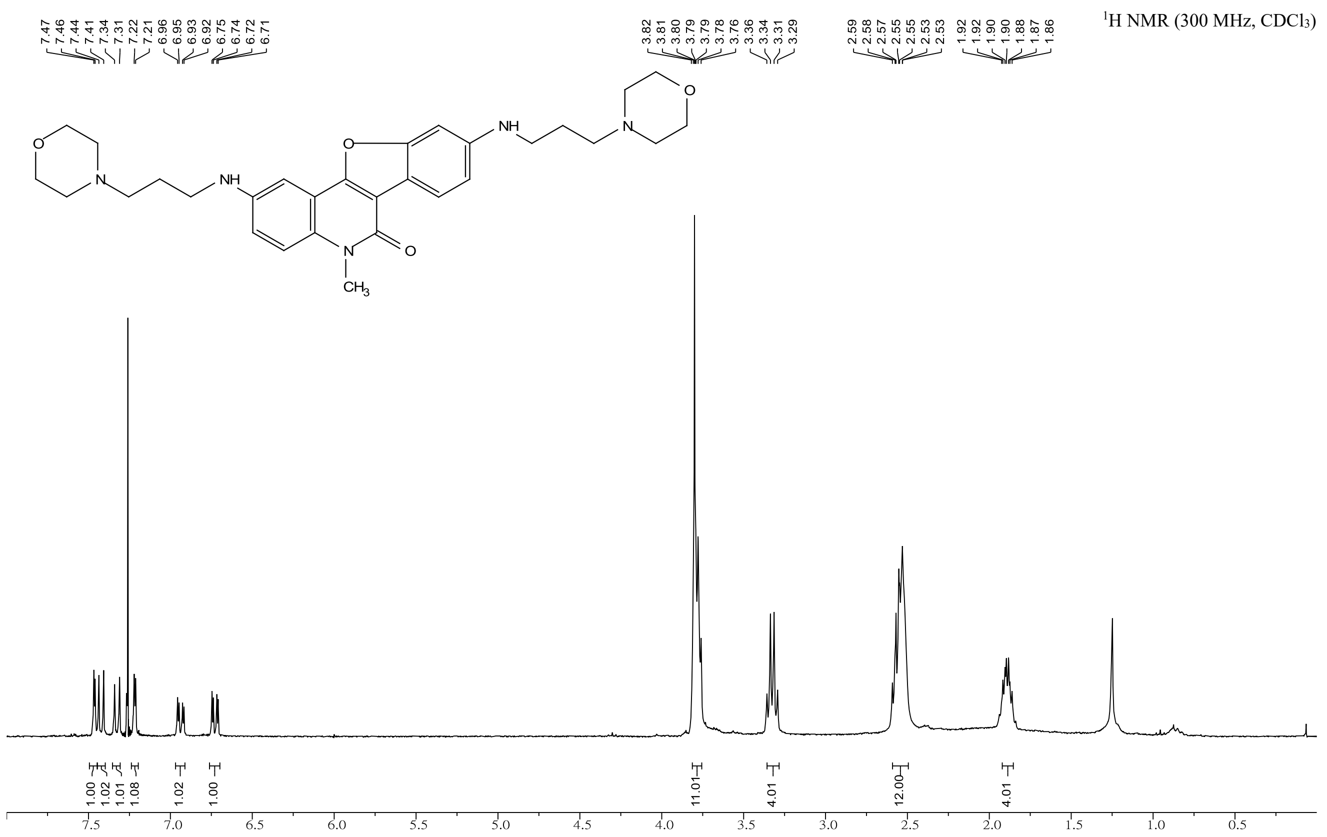

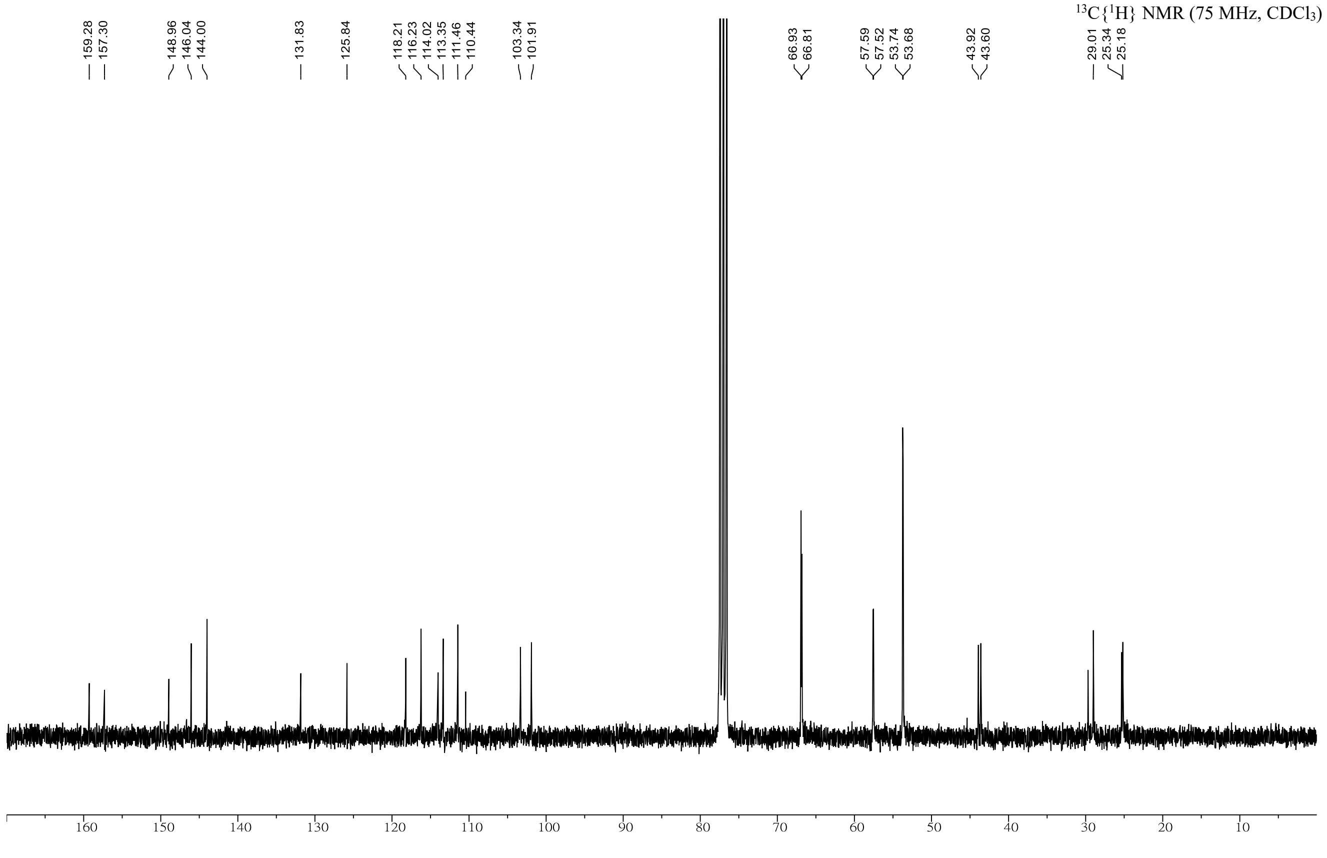

2,9-Bis{[3-(diethylamino)propyl]amino}benzofuro[3,2-*c*]quinolin-6(5*H*)-one (8f)

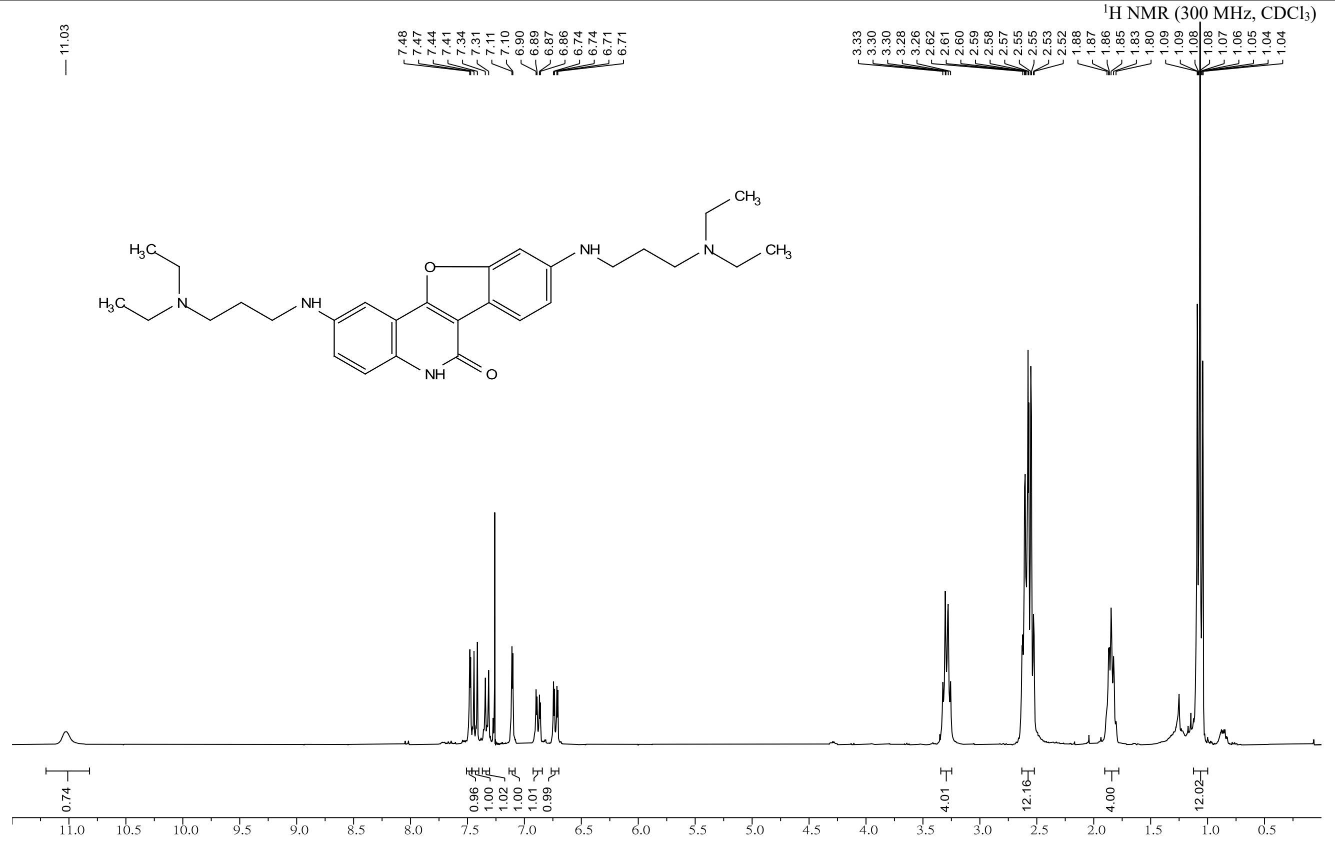

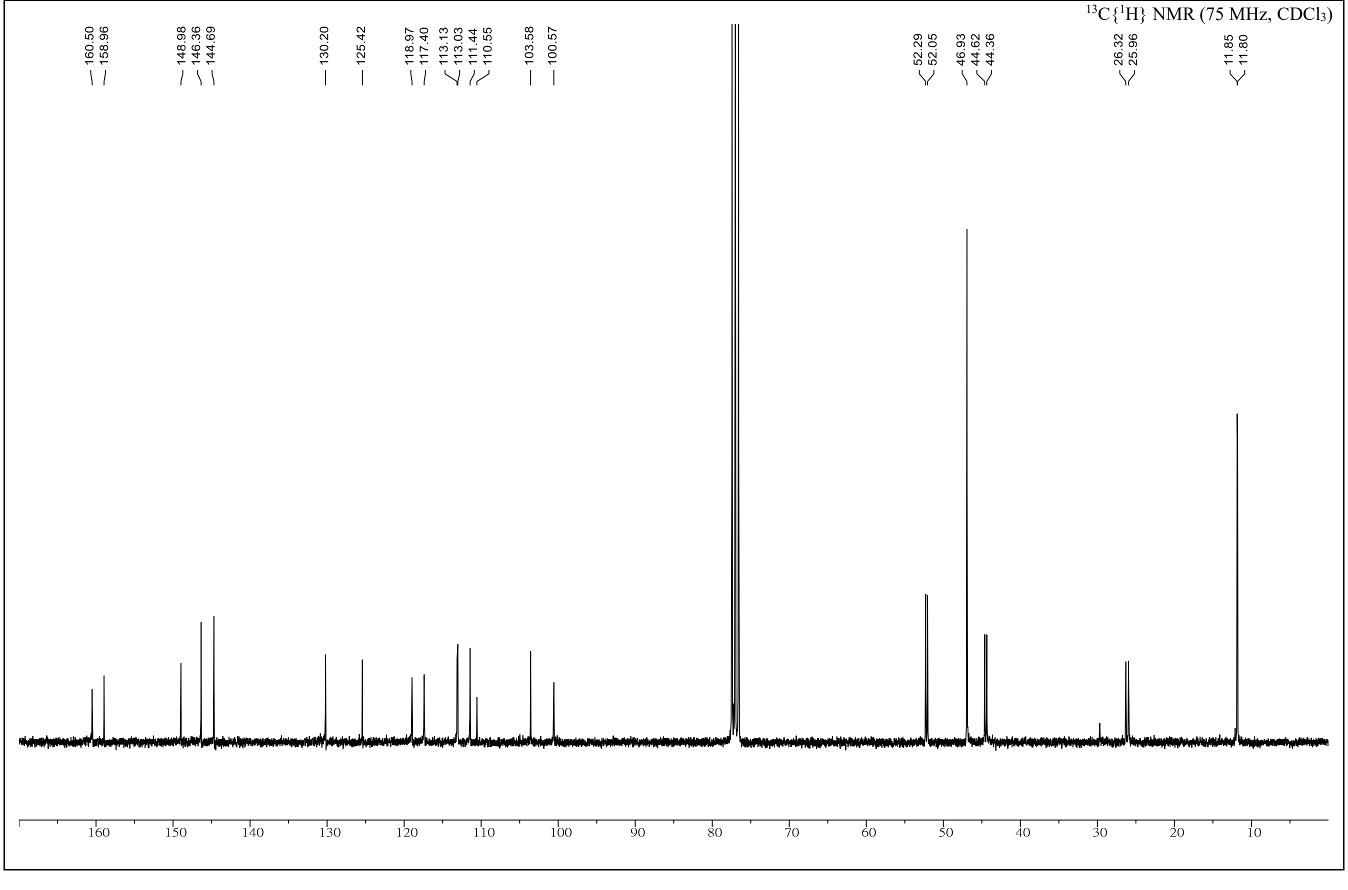

2,9-Bis{[3-(diethylamino)propyl]amino}benzofuro[3,2-*c*]quinoline (9)

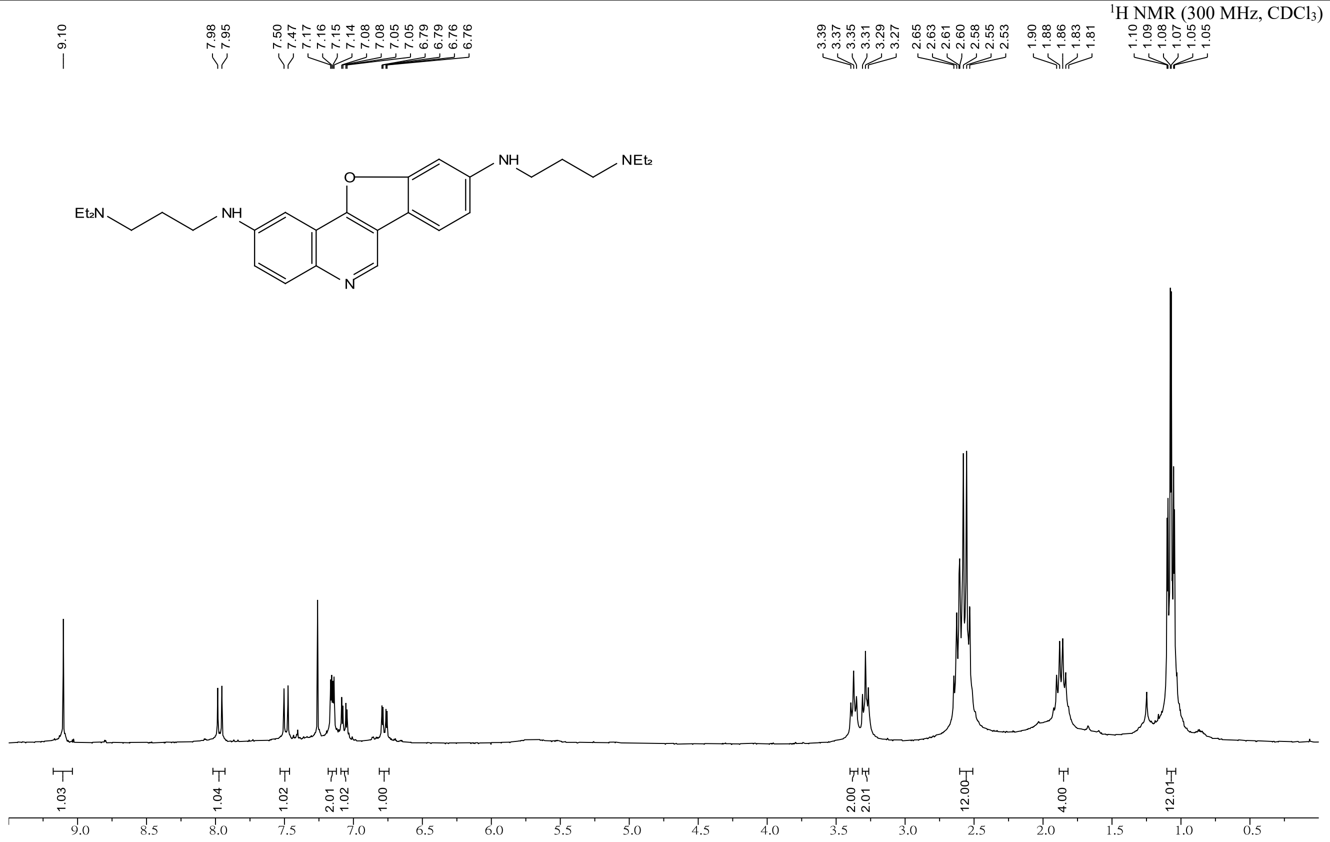

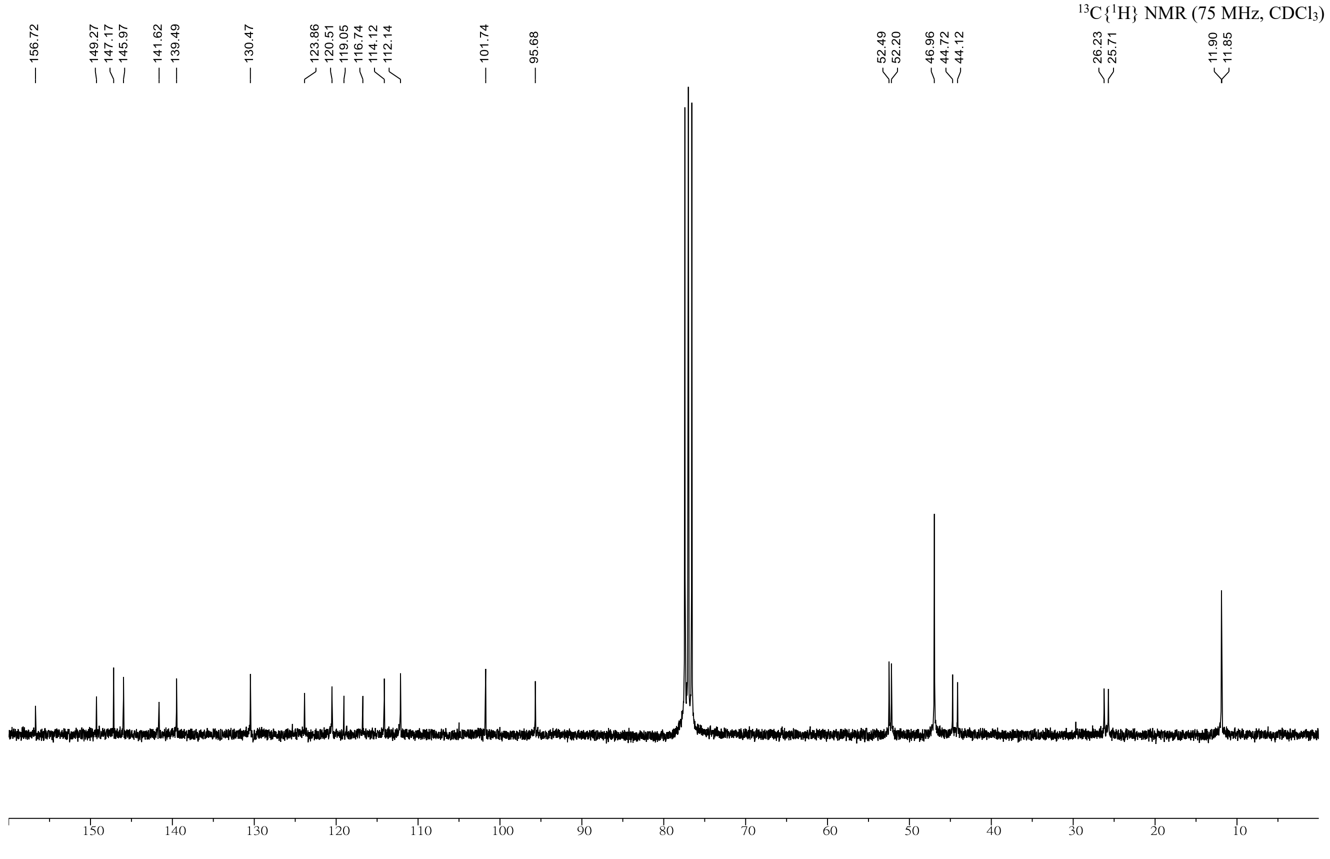

Methyl 2-[2-(2-Bromophenyl)acetamido]benzoate (4a)

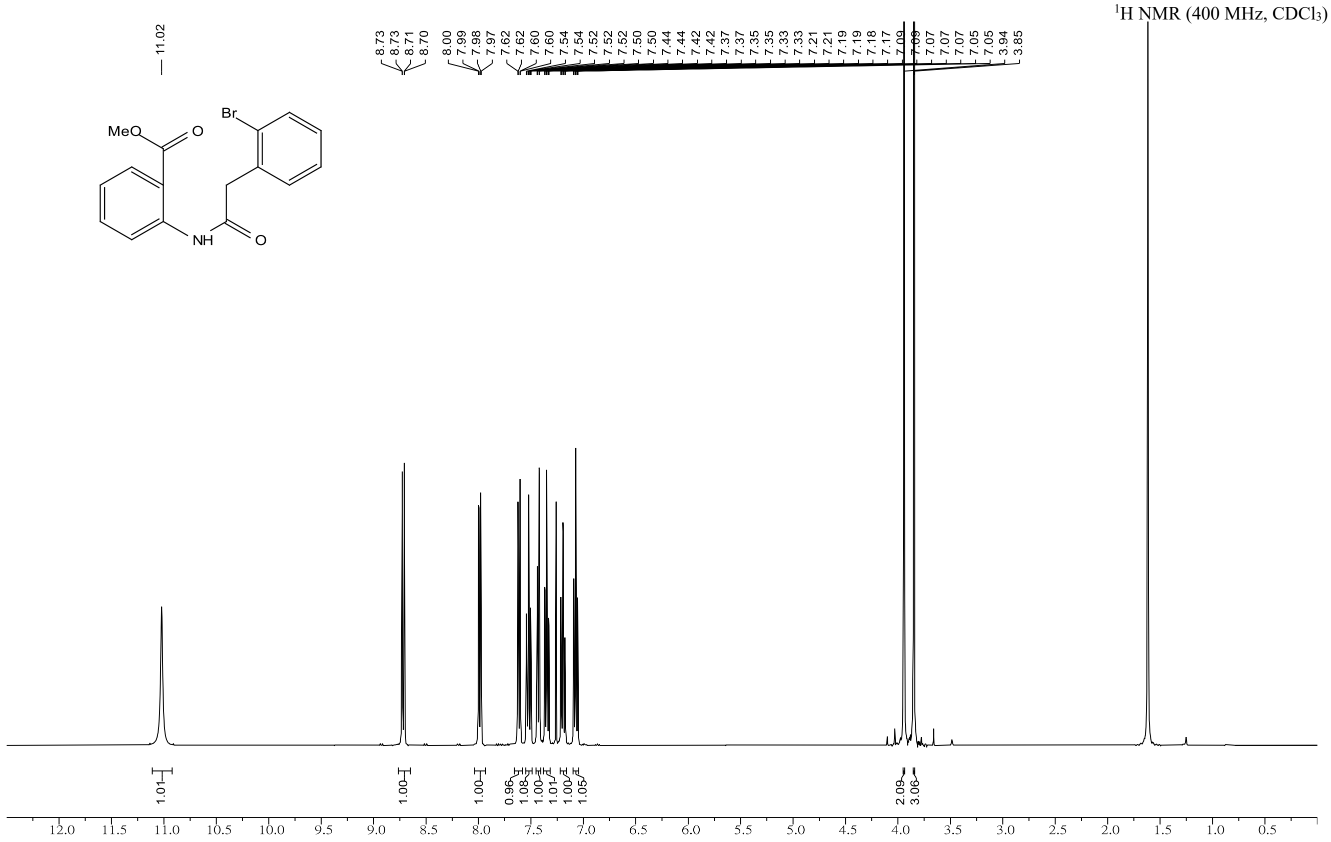

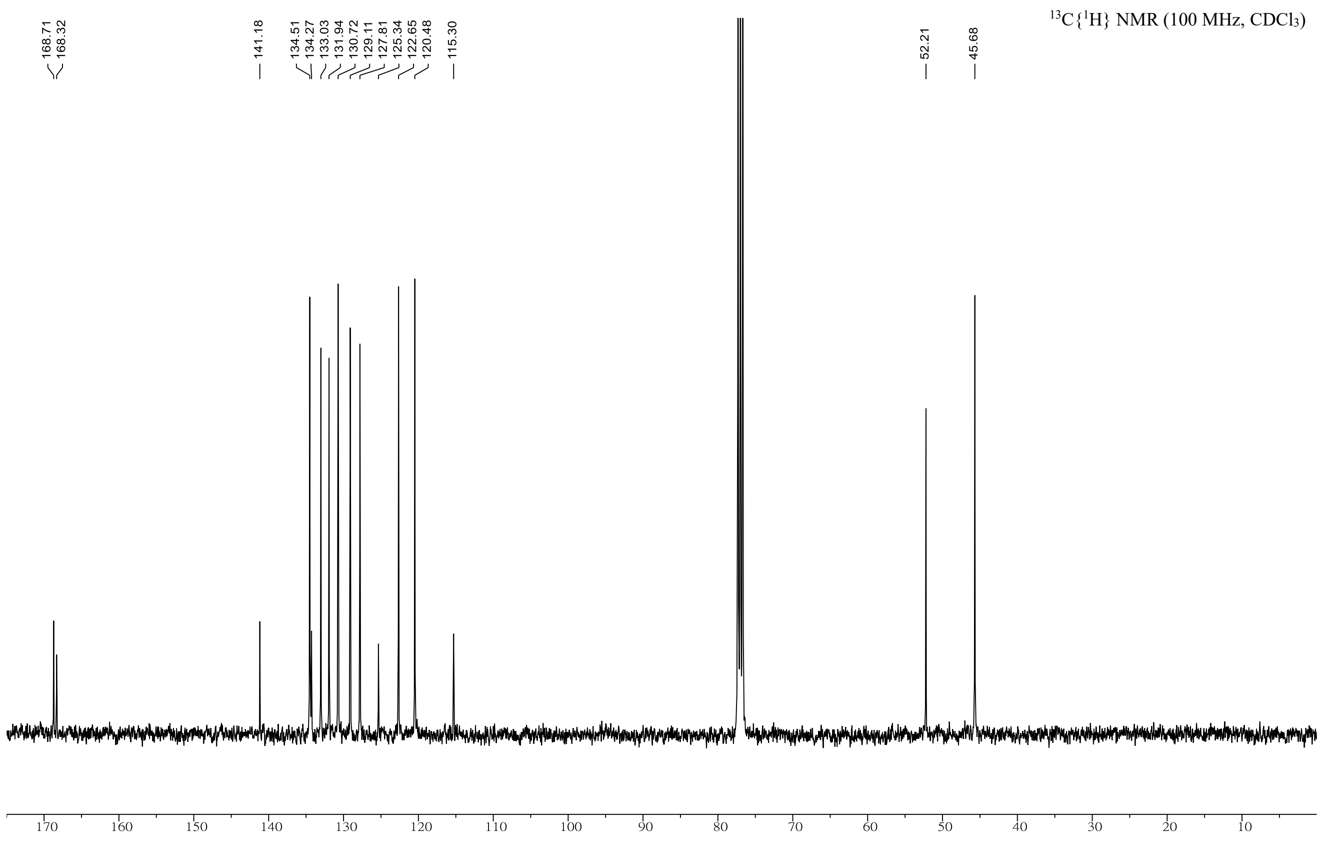

Methyl 2-[-(2-Bromophenyl)acetamido]-5-fluorobenzoate (4b)

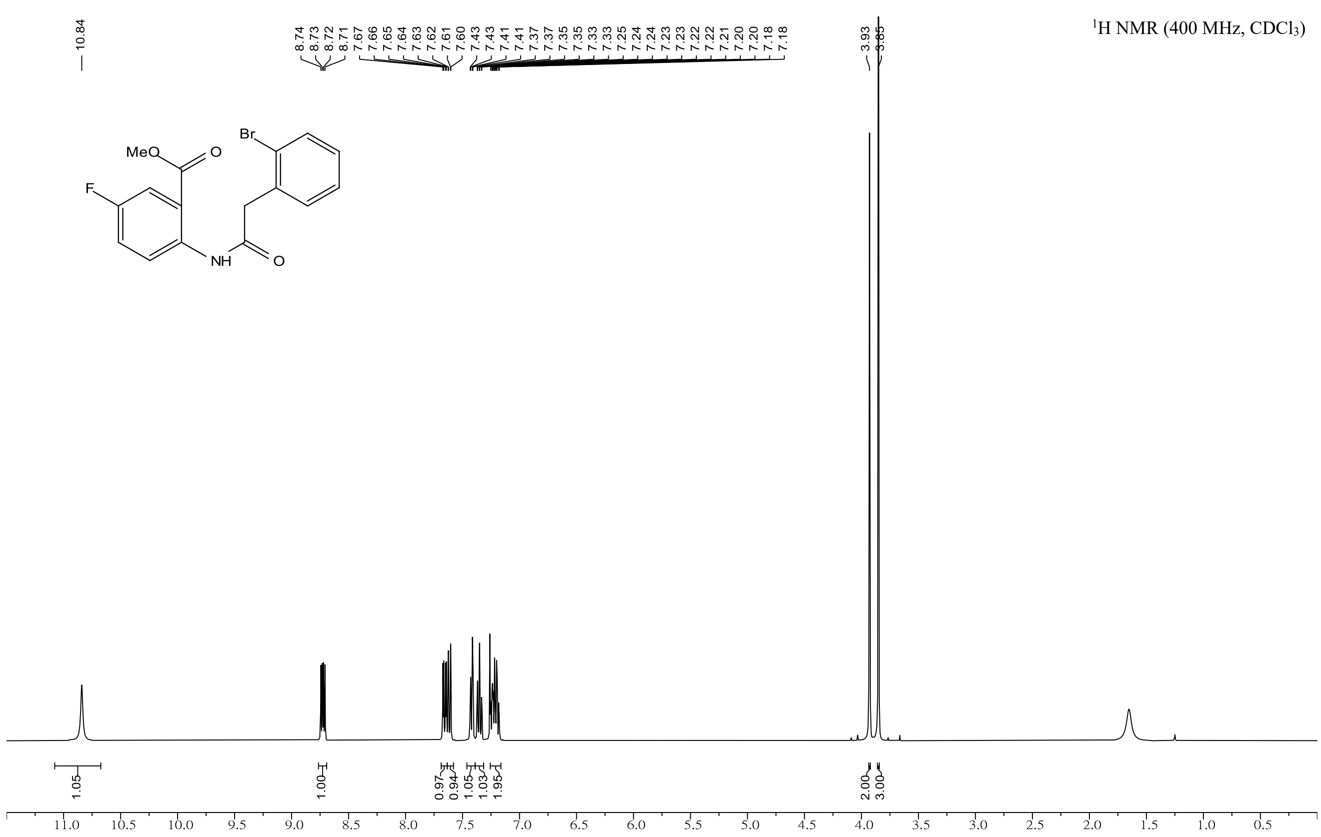

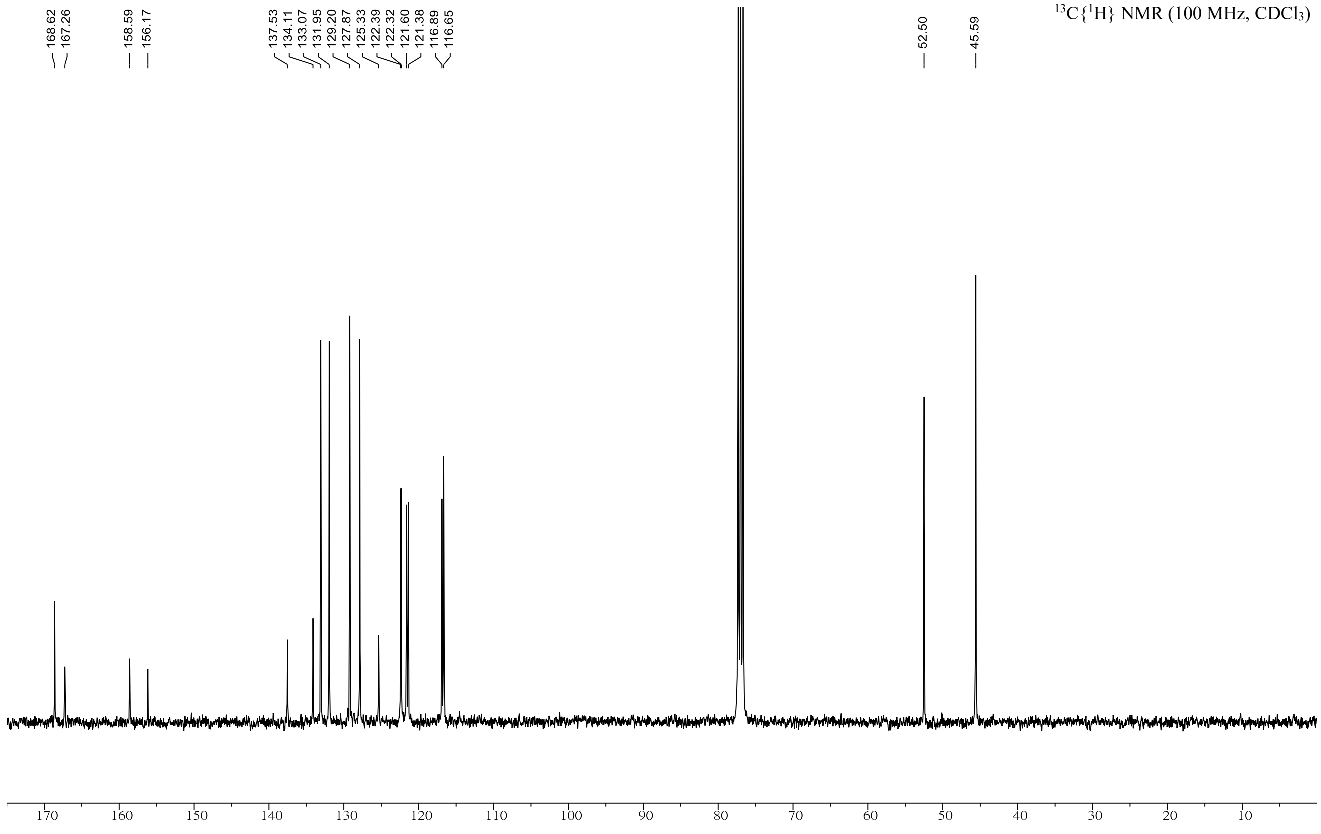

Methyl 2-[2-(2-Bromophenyl)acetamido]-5-chlorobenzoate (4c)

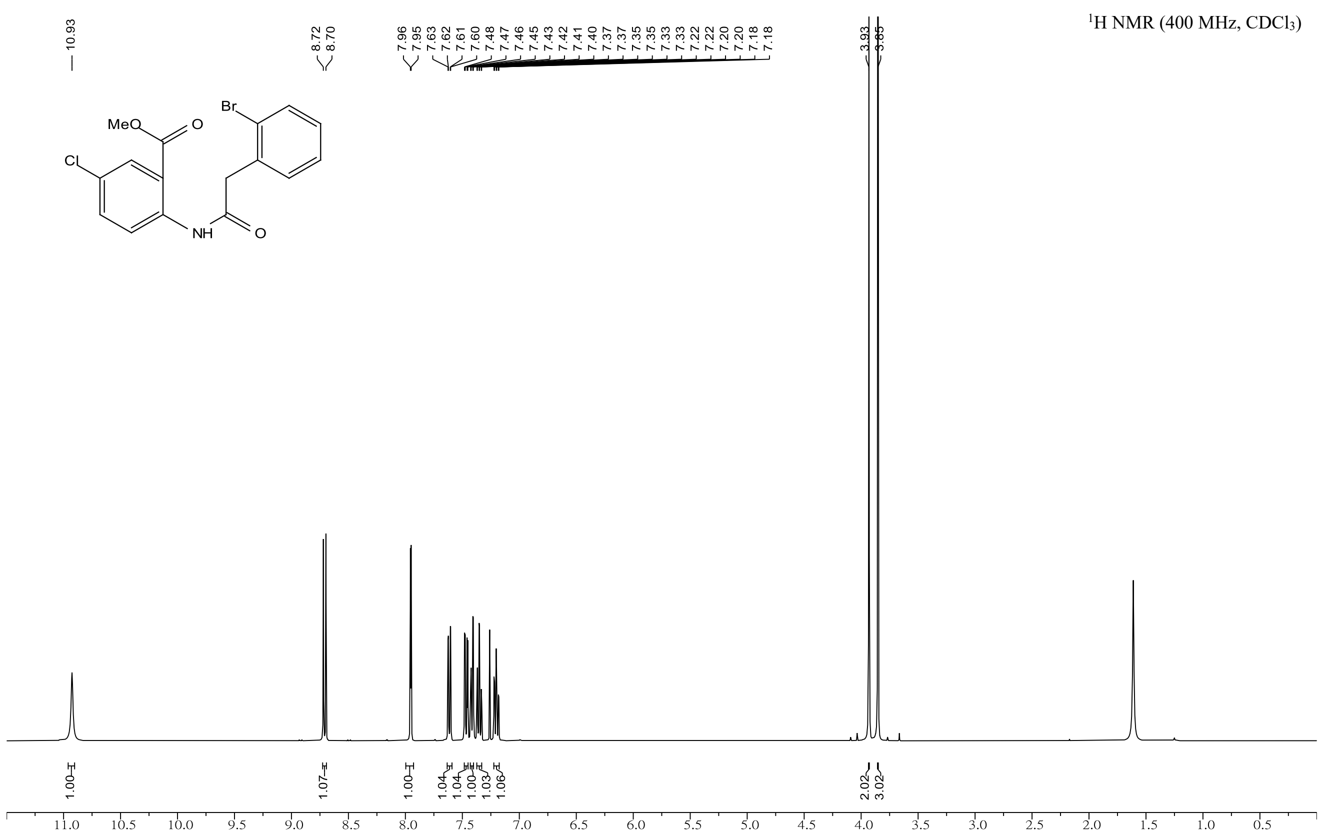

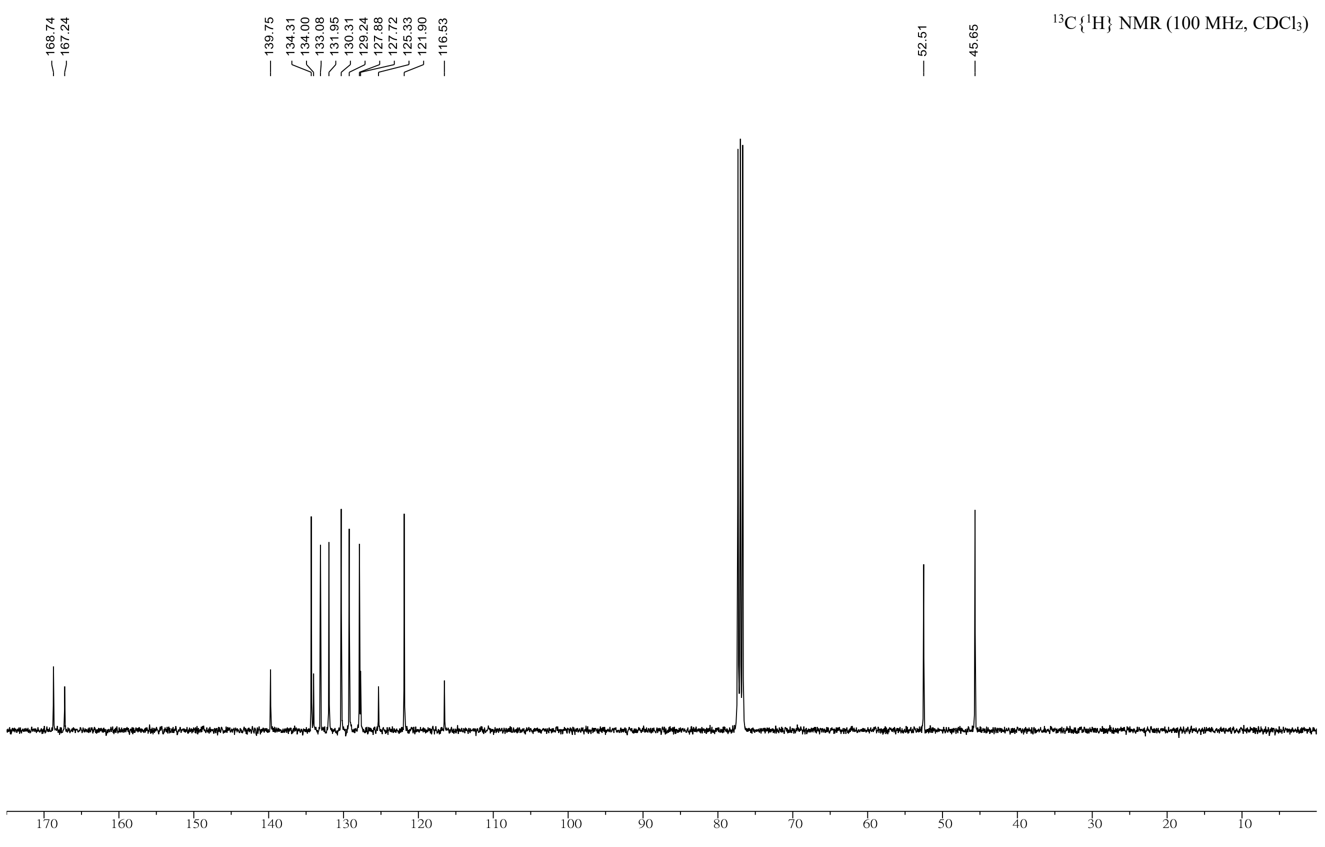

Methyl 5-Bromo-2-[2-(2-bromophenyl)acetamido]benzoate (4d)

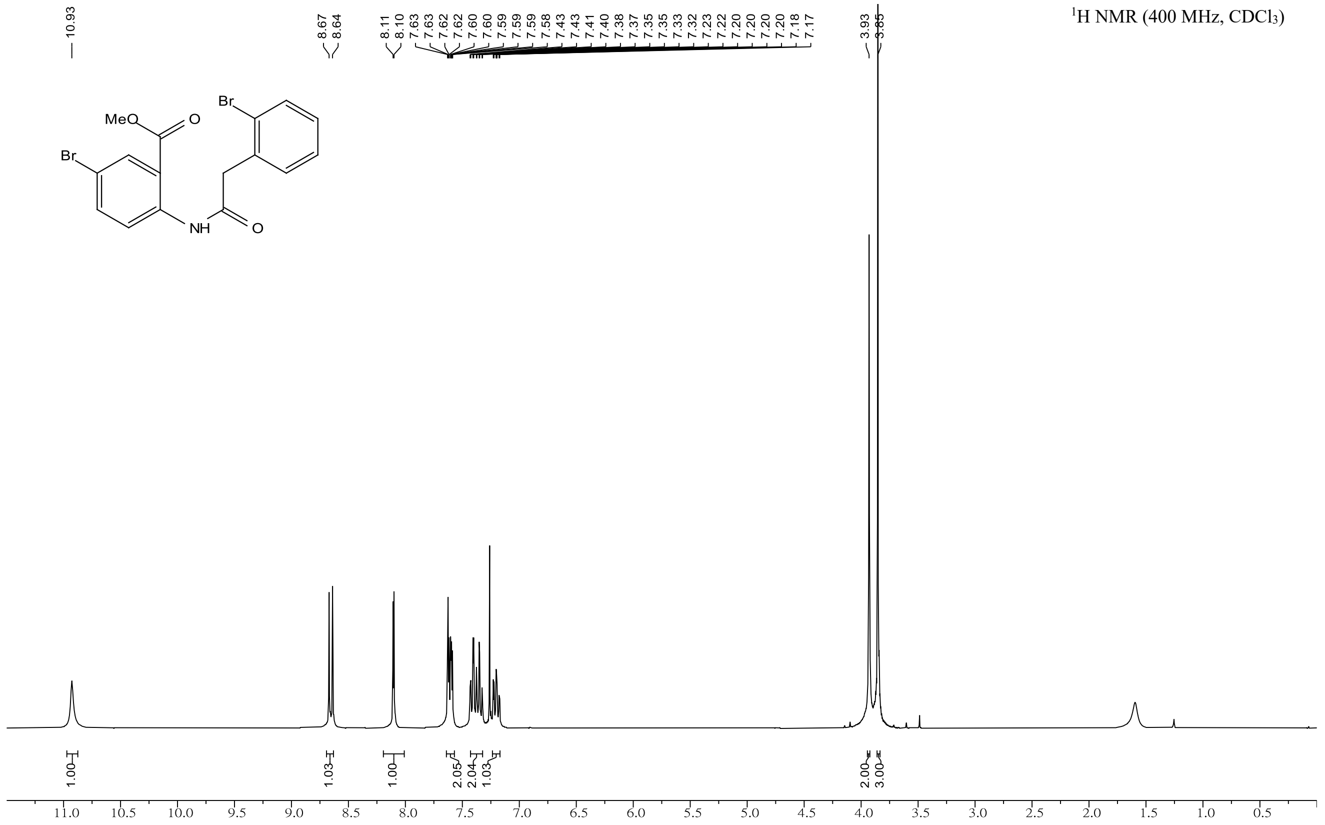

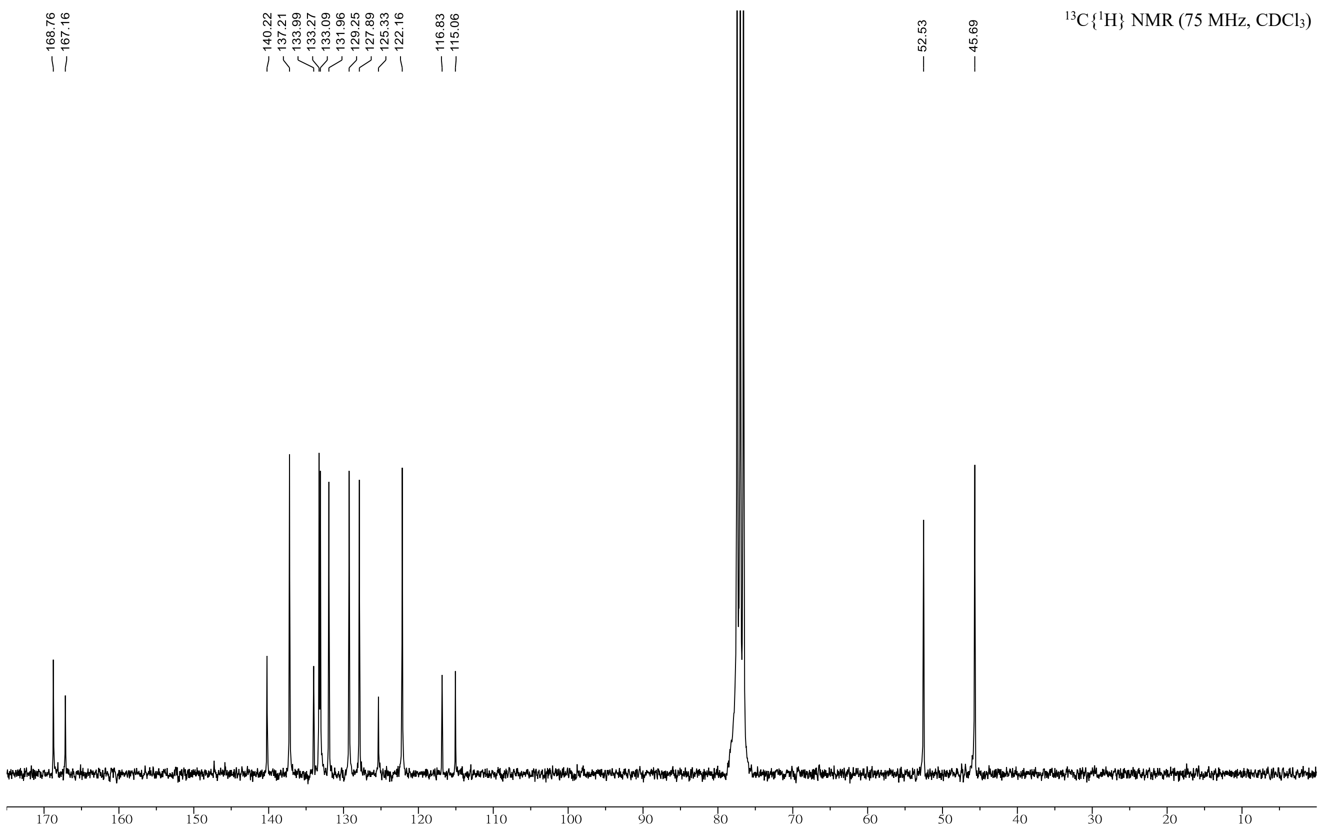

Methyl 2-[2-(2-Bromophenyl)acetamido]-5-iodobenzoate (4e)

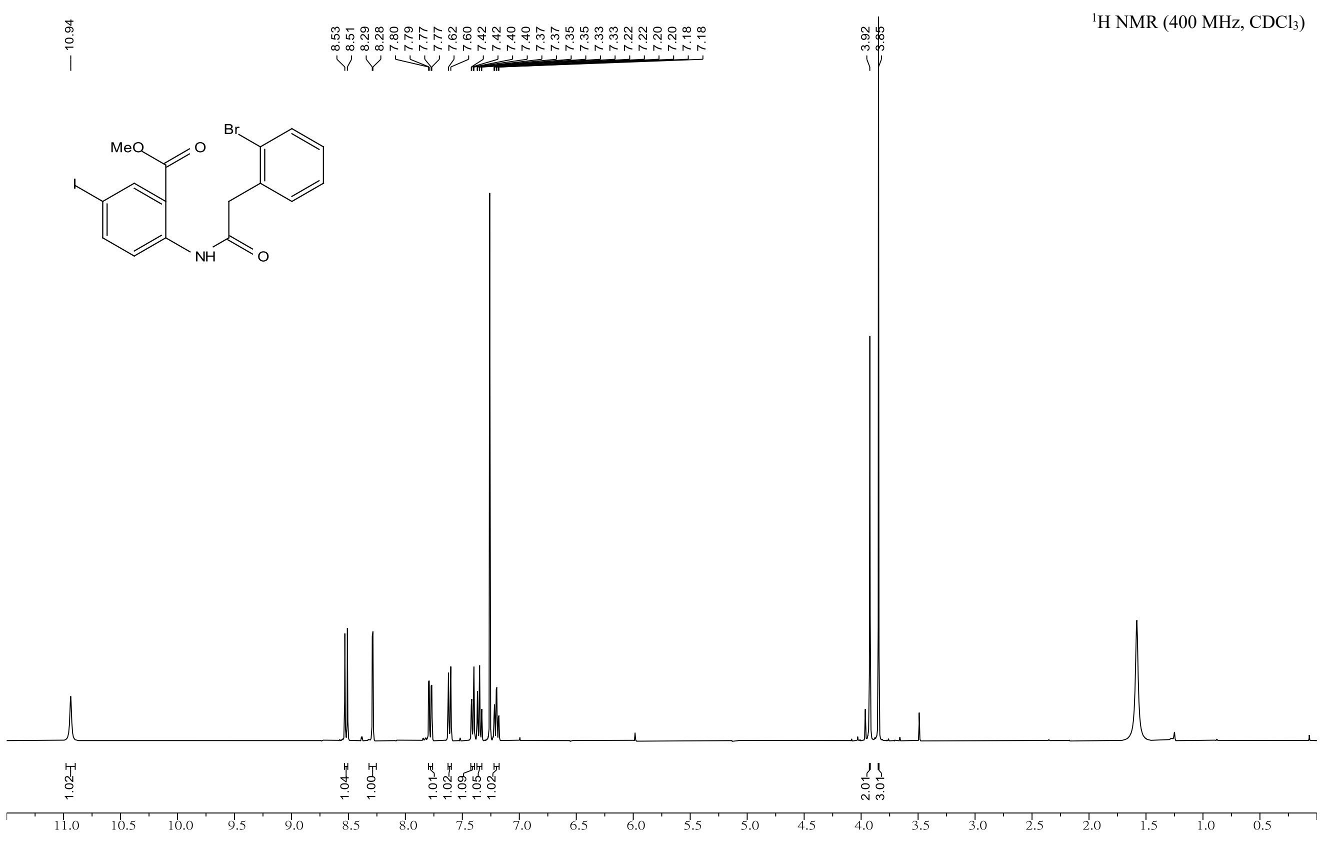

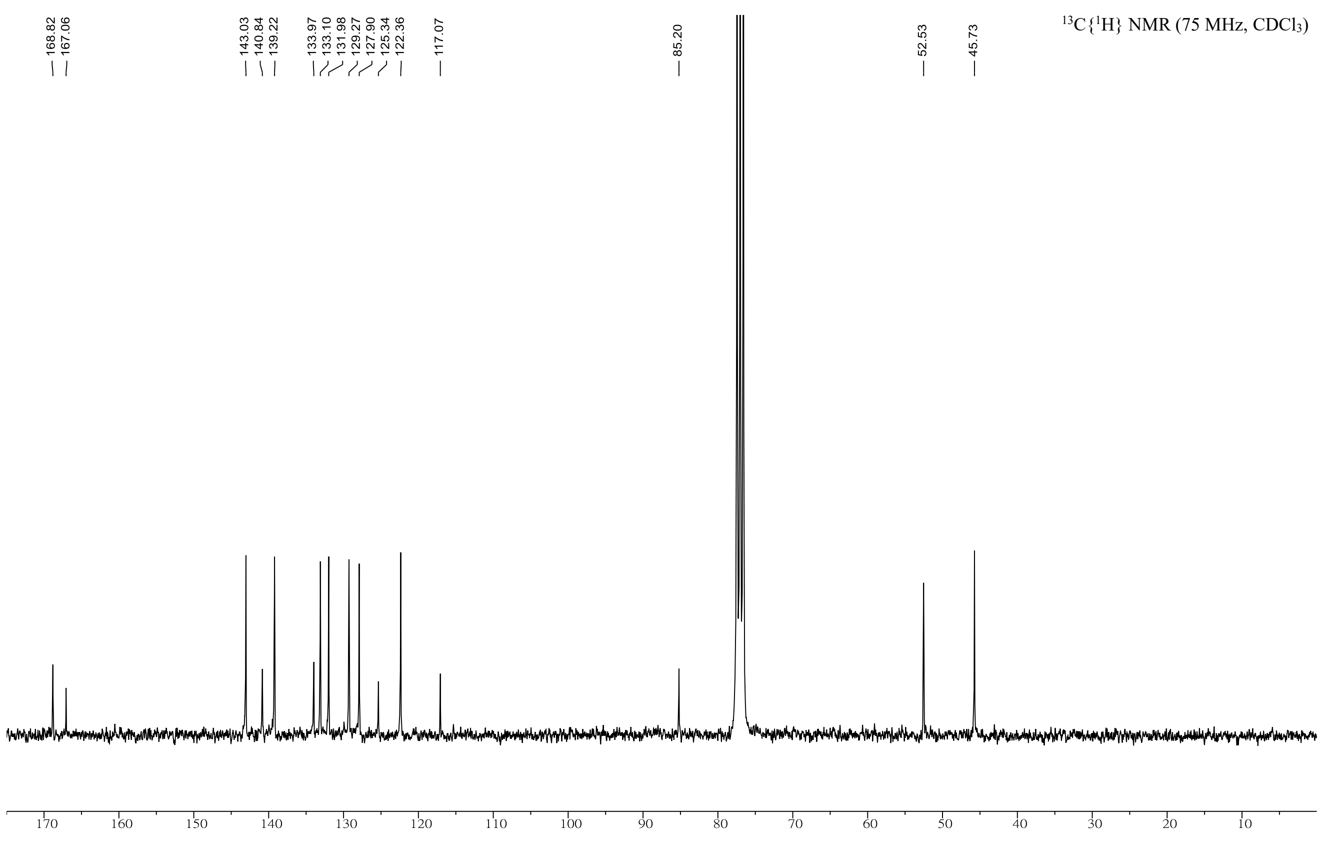

Methyl 2-[2-(2-Bromophenyl)acetamido]-5-nitrobenzoate (4f)

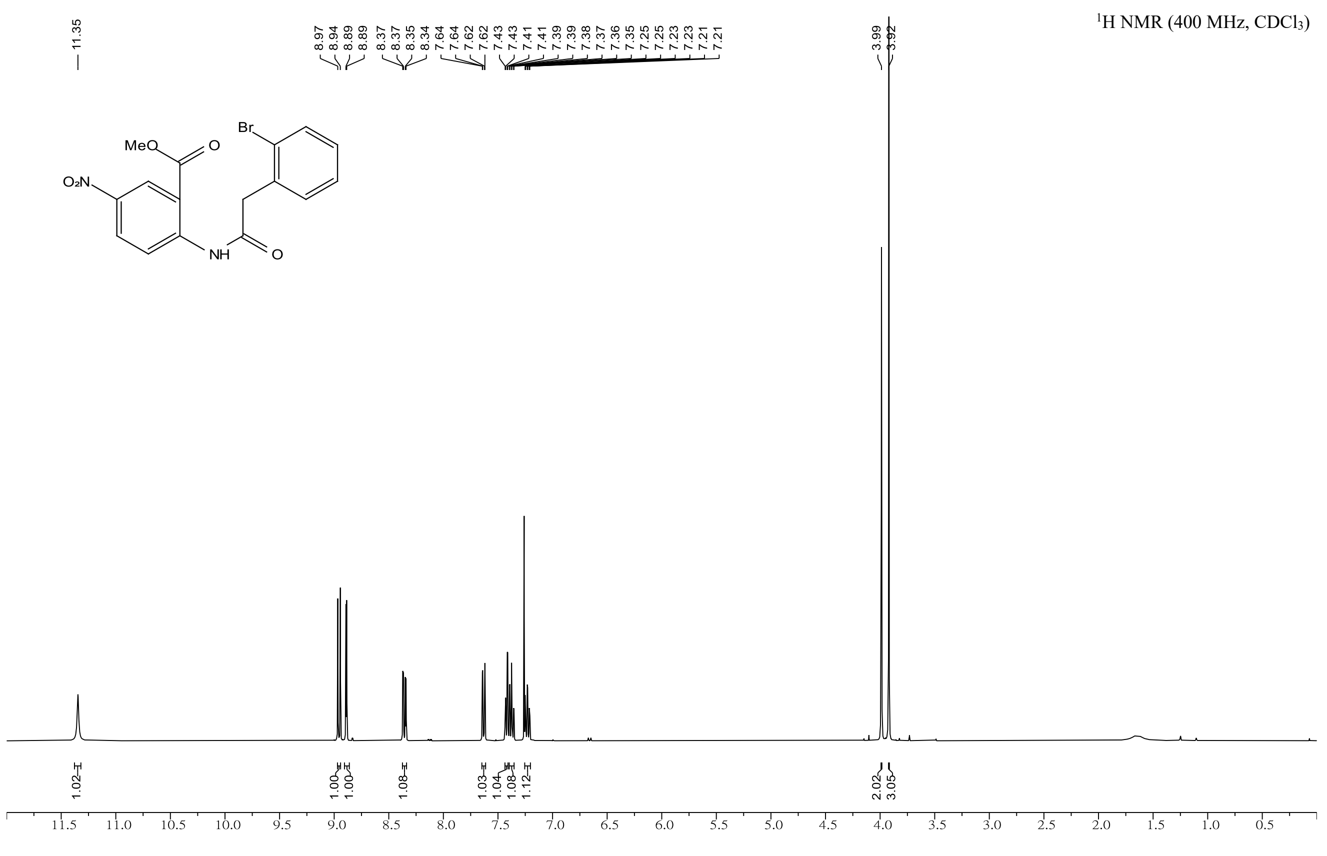

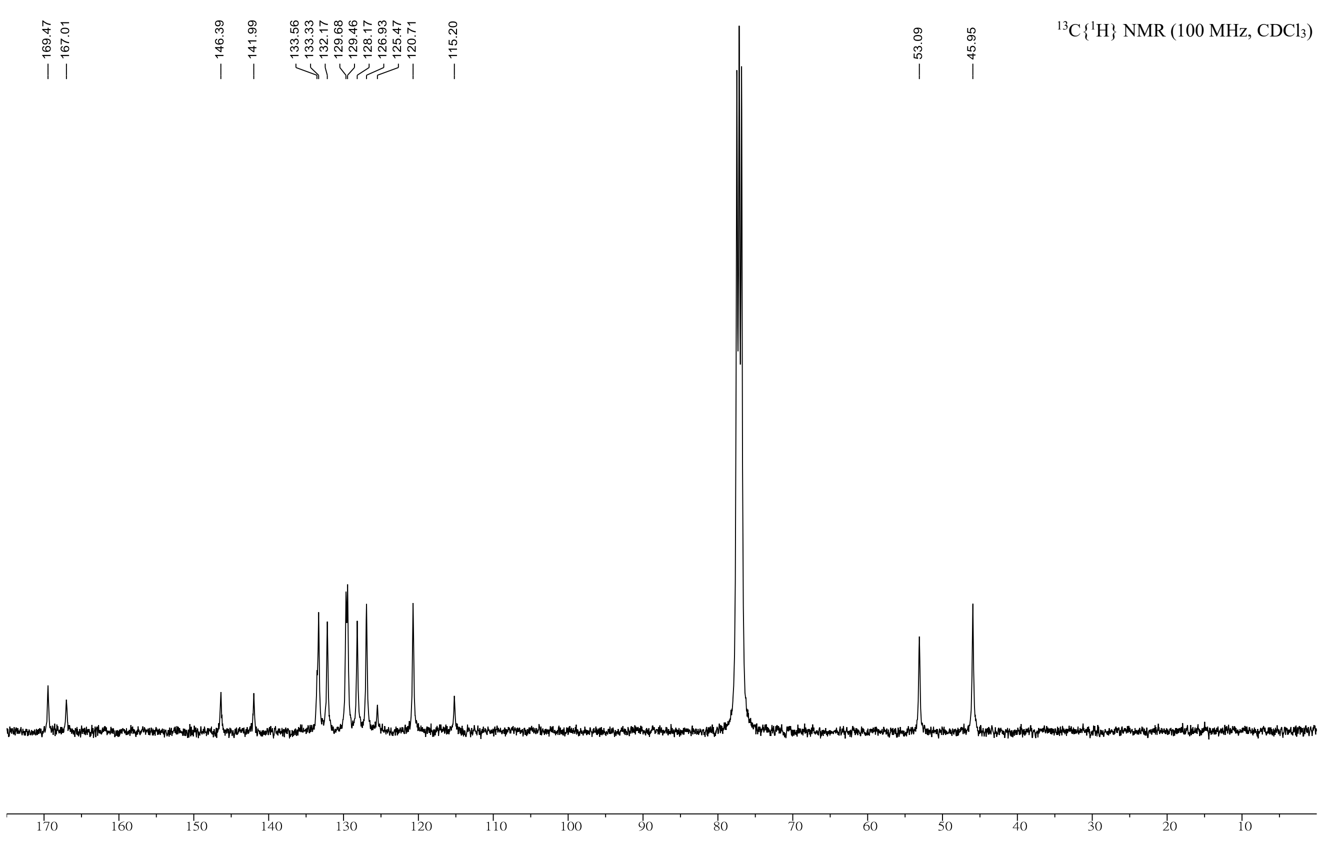

Methyl 2-[2-(2-Bromophenyl)acetamido]-5-methoxybenzoate (4g)

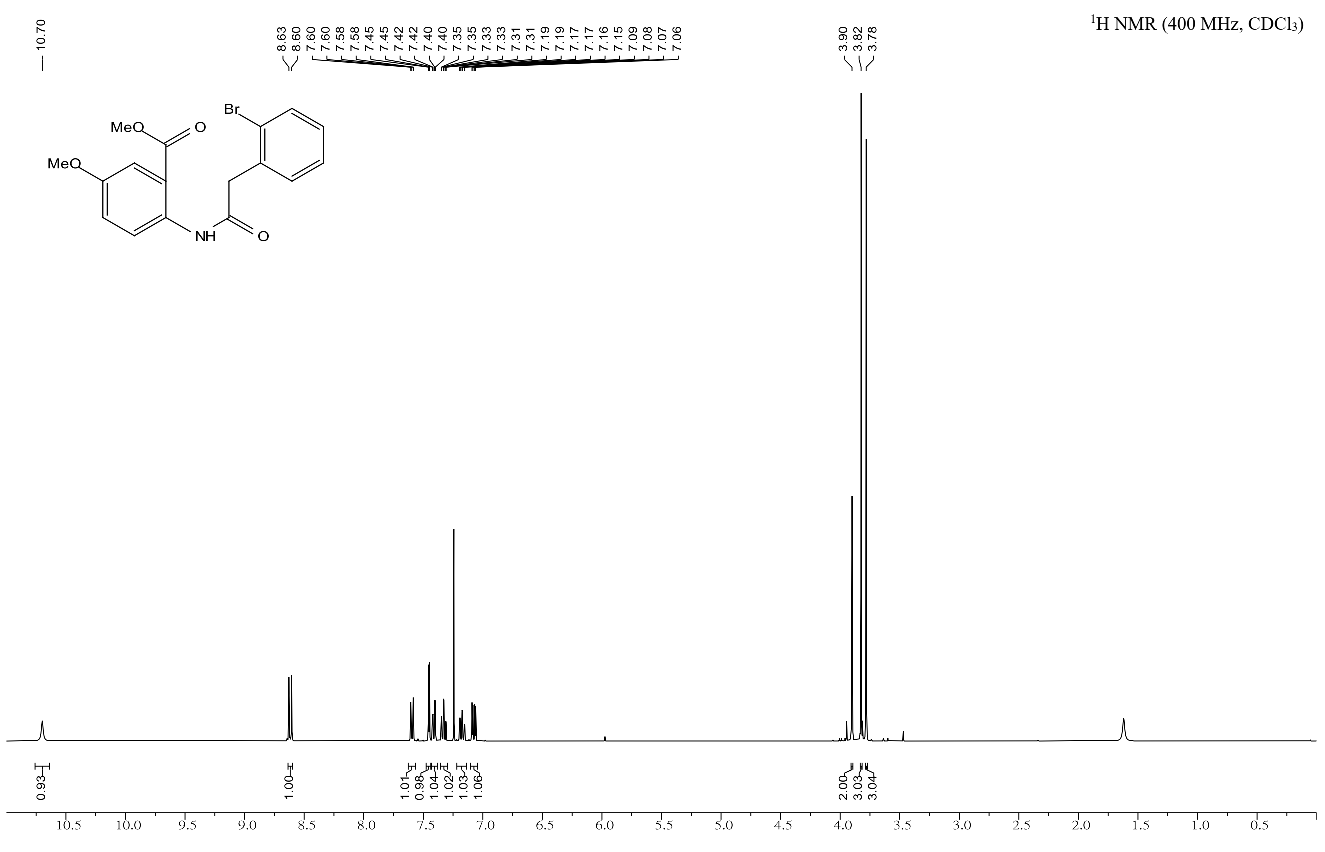

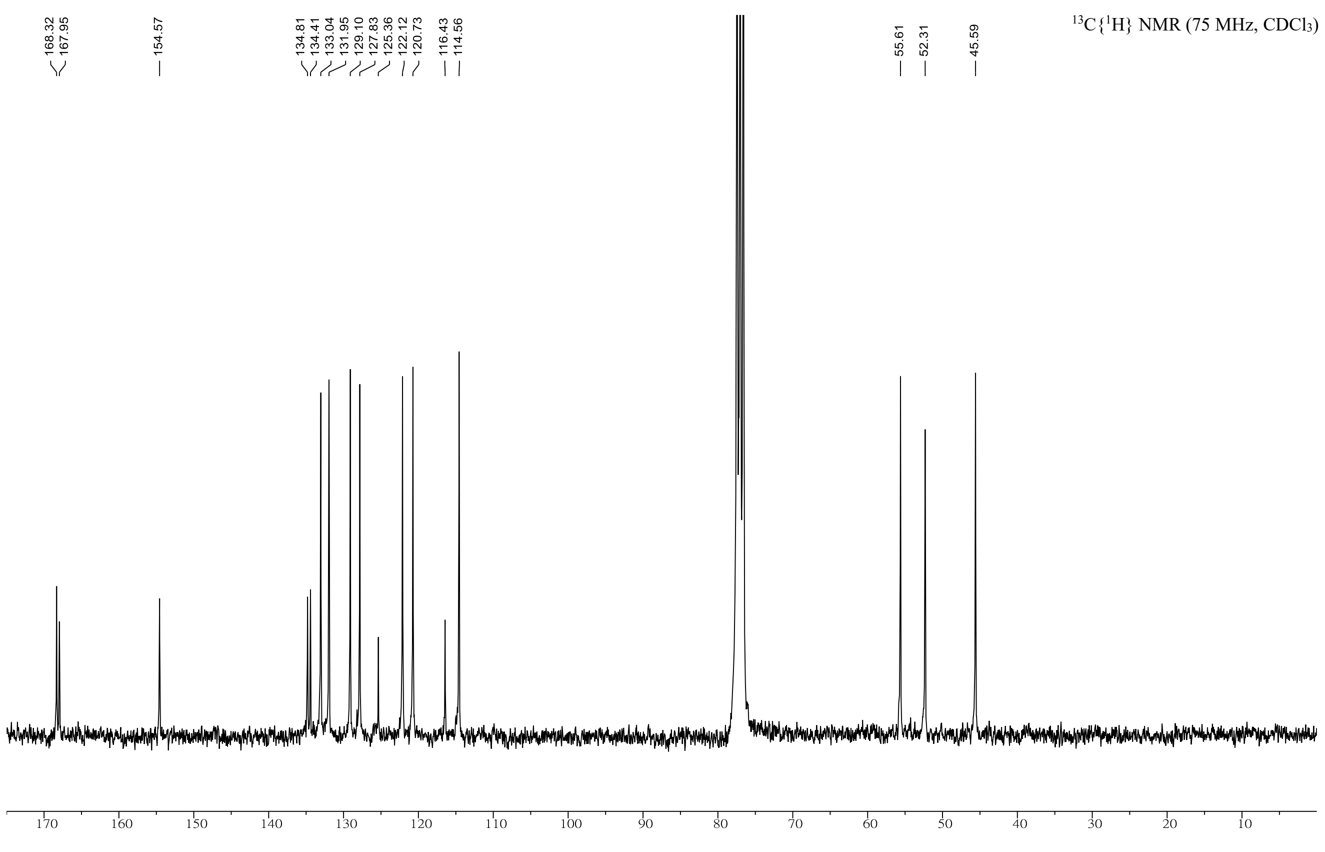

Methyl 2-[2-(2-Bromophenyl)acetamido]-4-fluorobenzoate (4h)

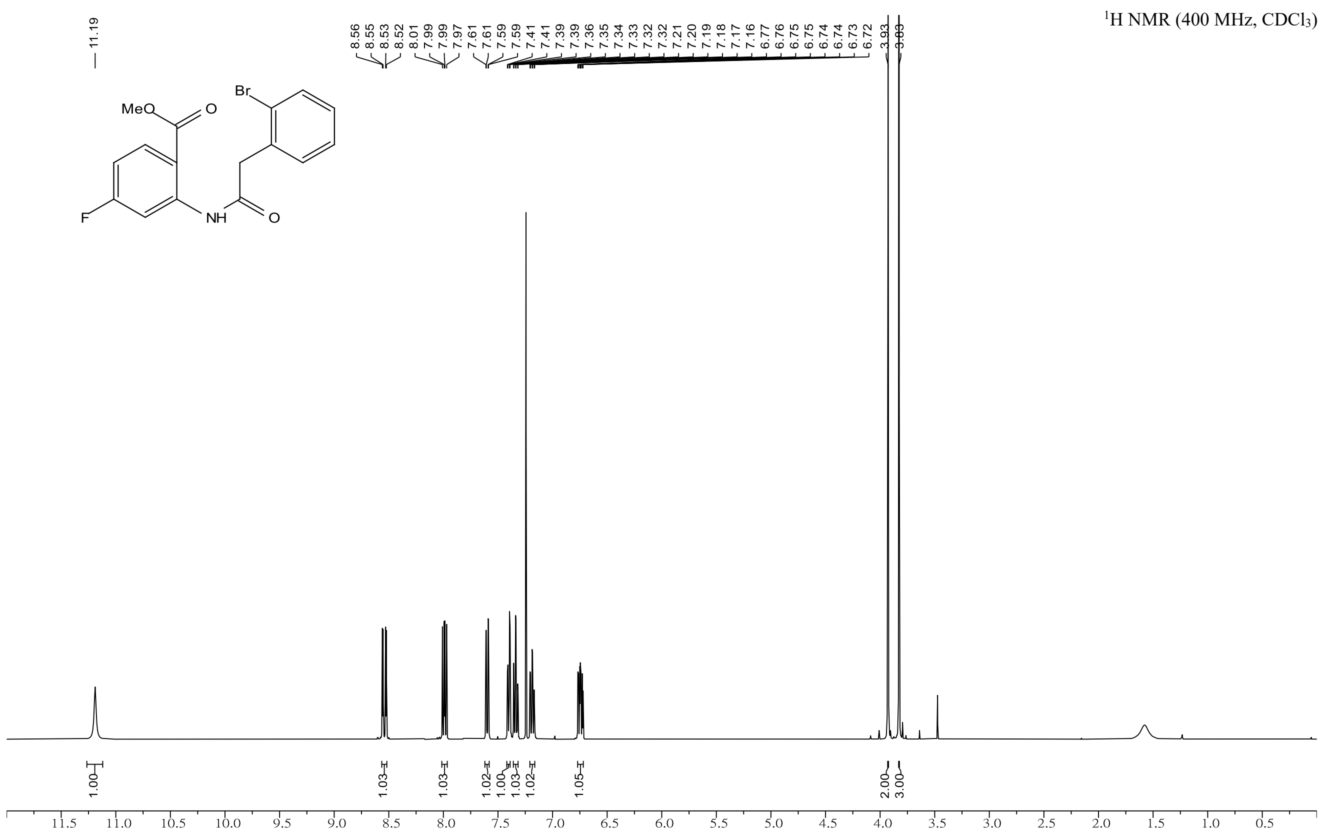

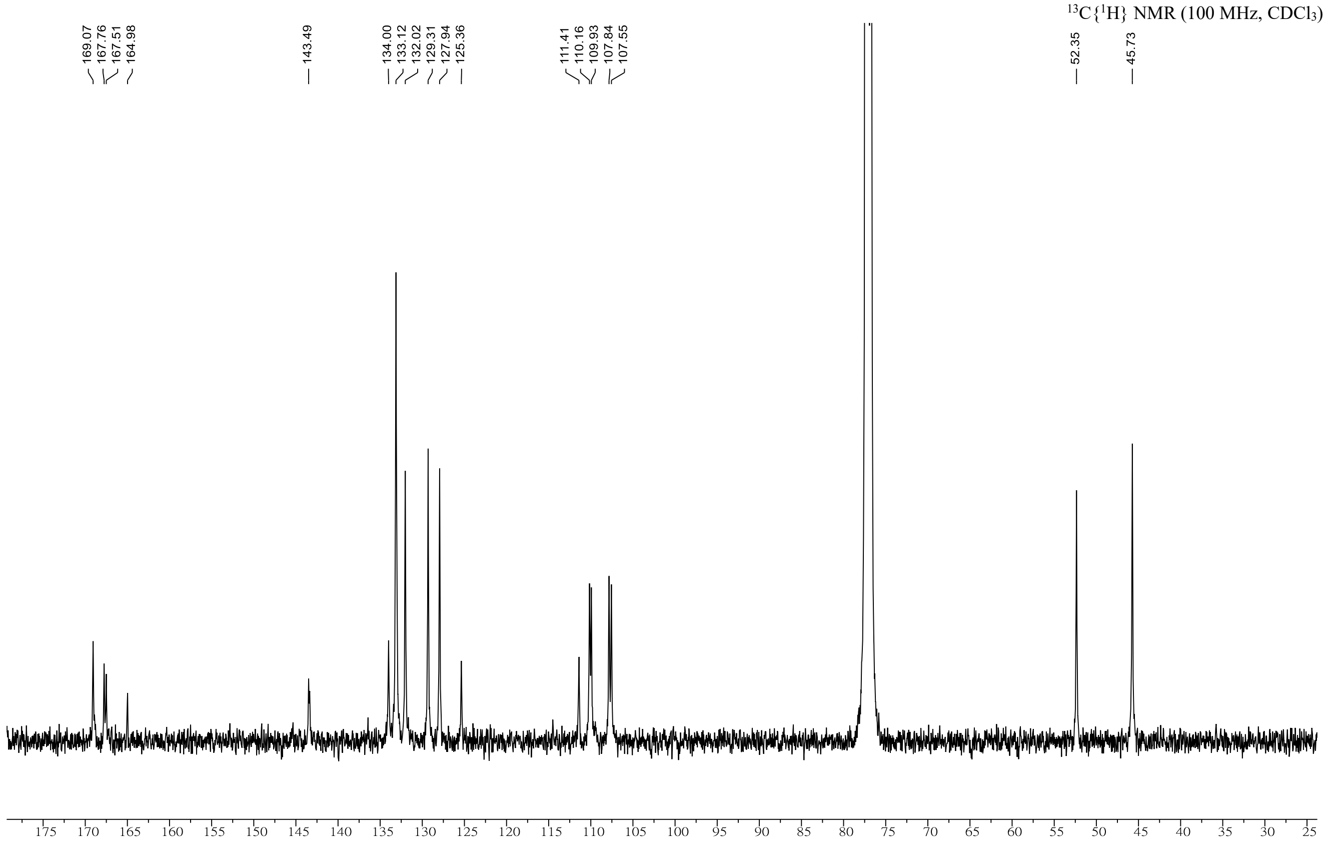

Methyl 2-[2-(2-Bromophenyl)acetamido]-4-chlorobenzoate (4i)

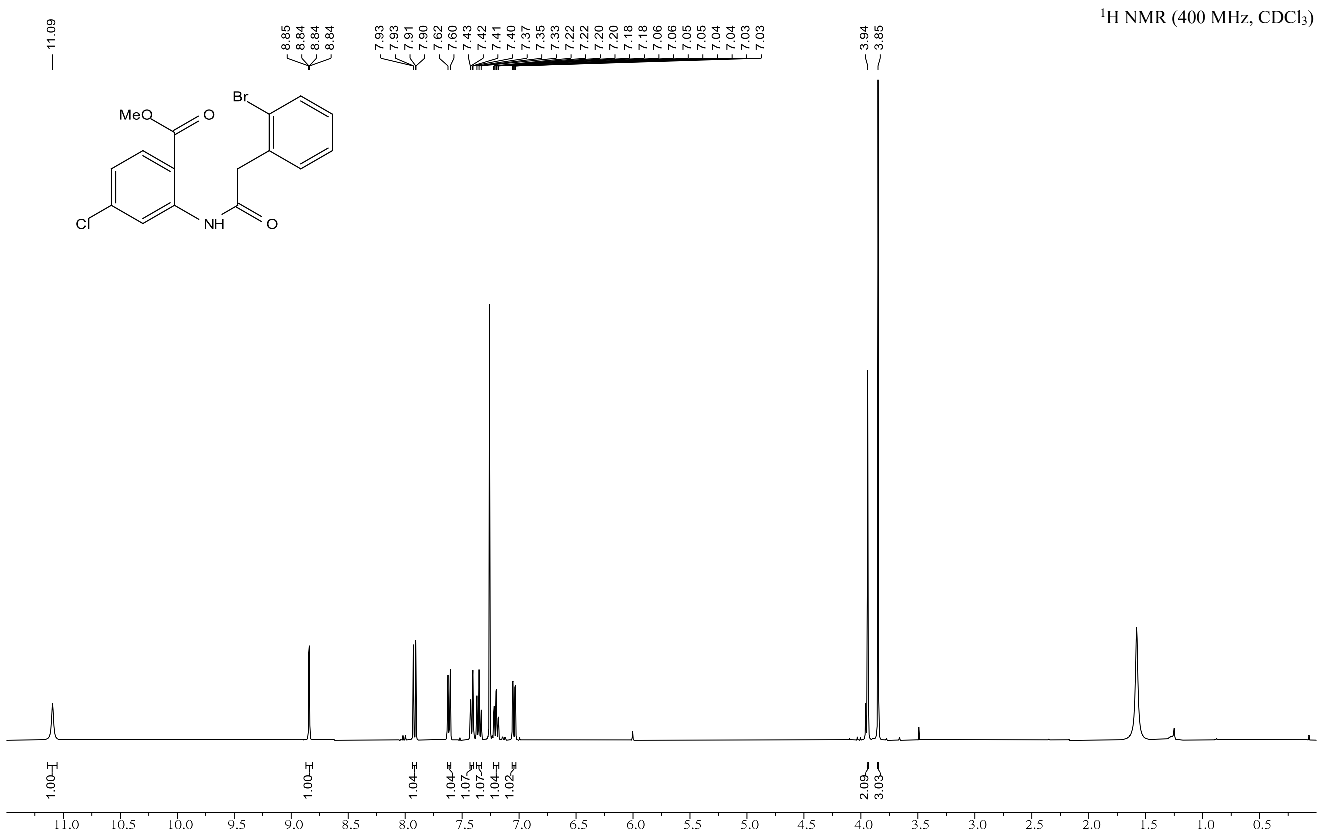

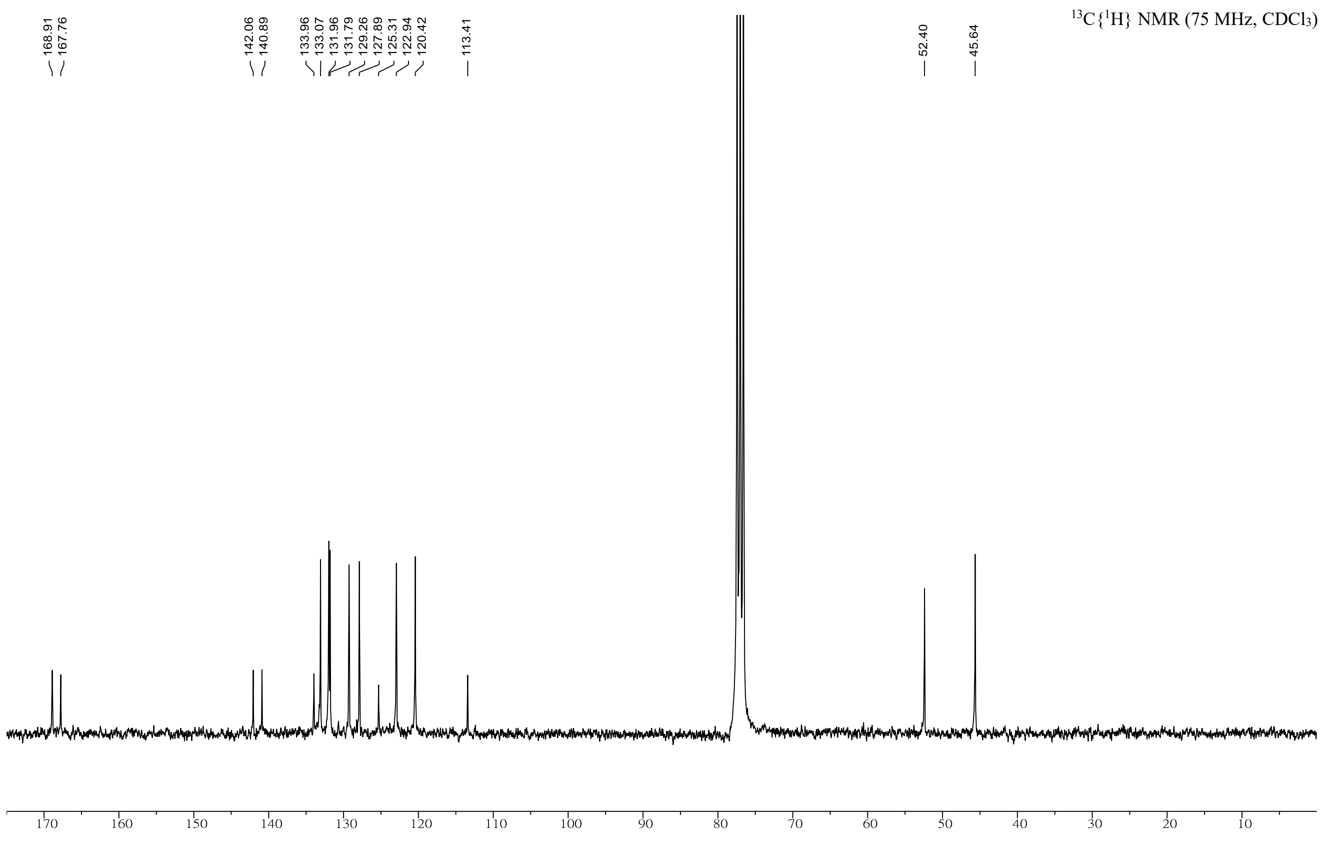

Methyl 4-Bromo-2-[2-(2-bromophenyl)acetamido]benzoate (4j)

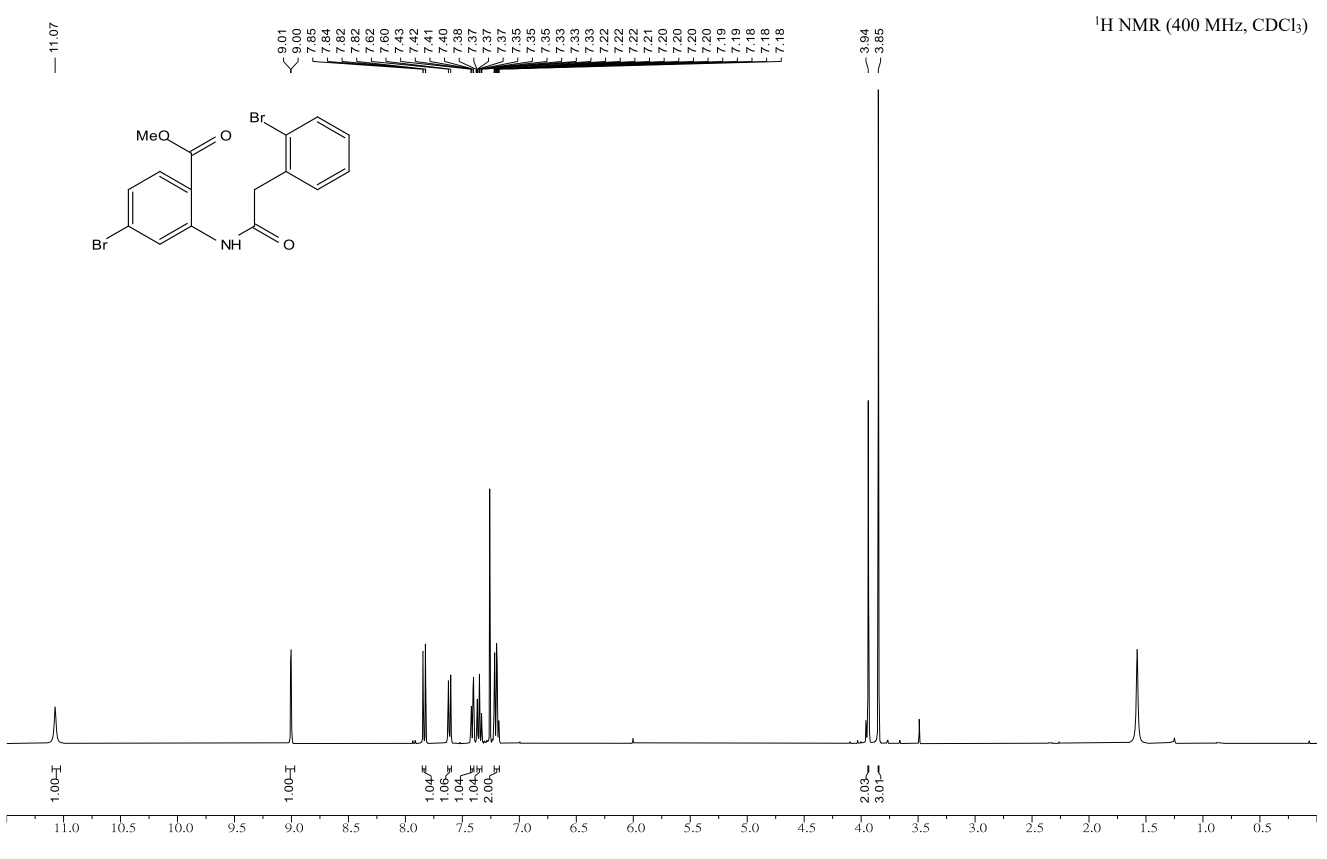

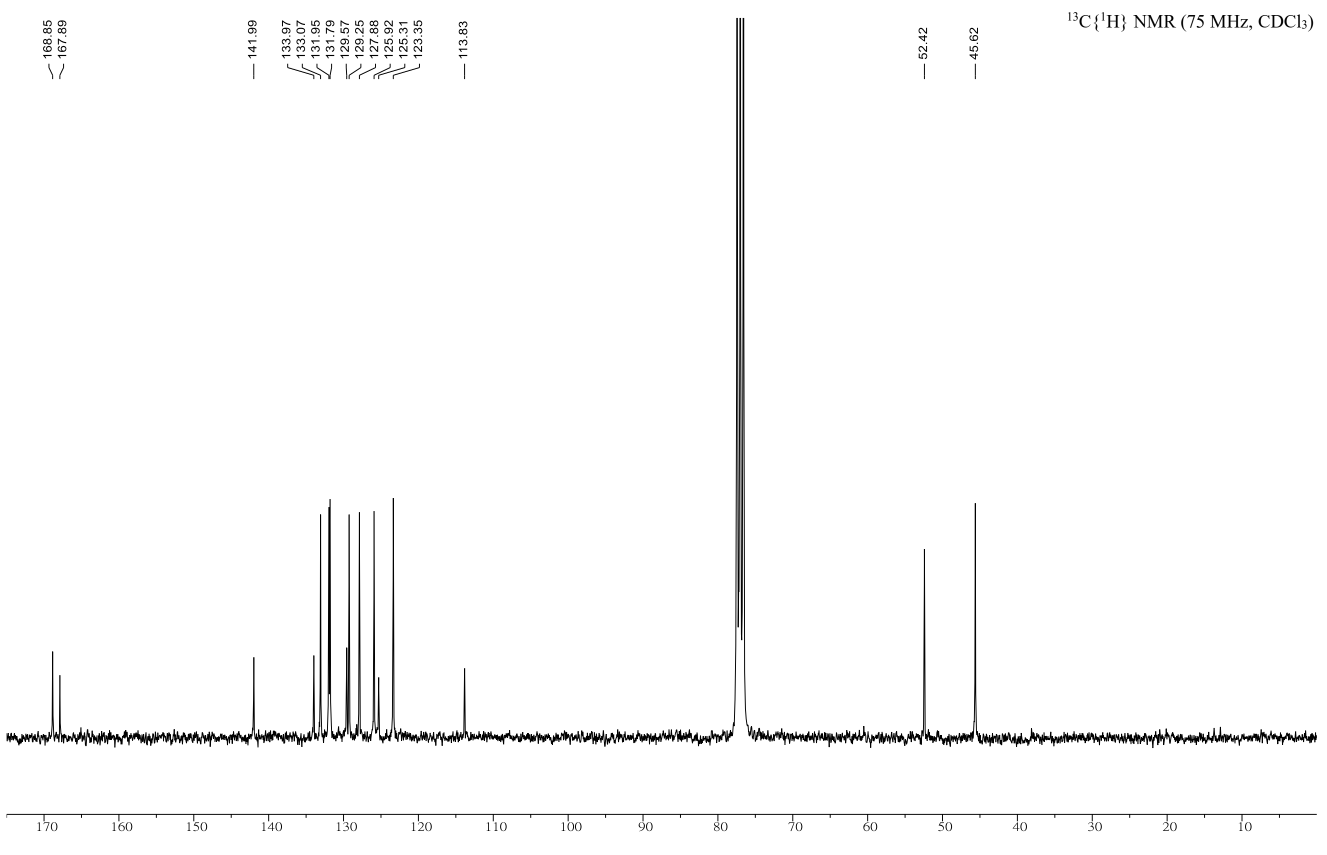

Methyl 2-[2-(2-Bromophenyl)acetamido]-4-methylbenzoate (4k)

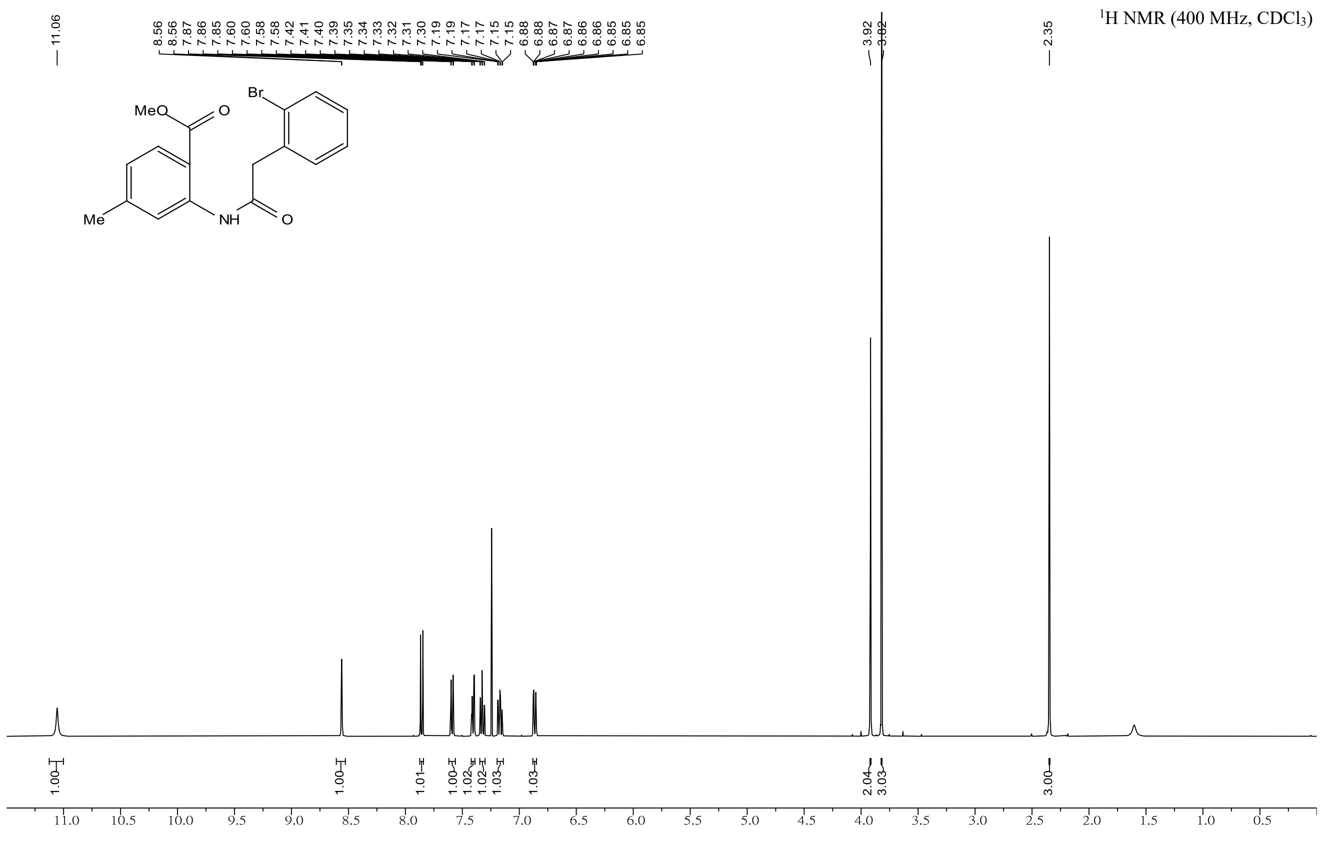

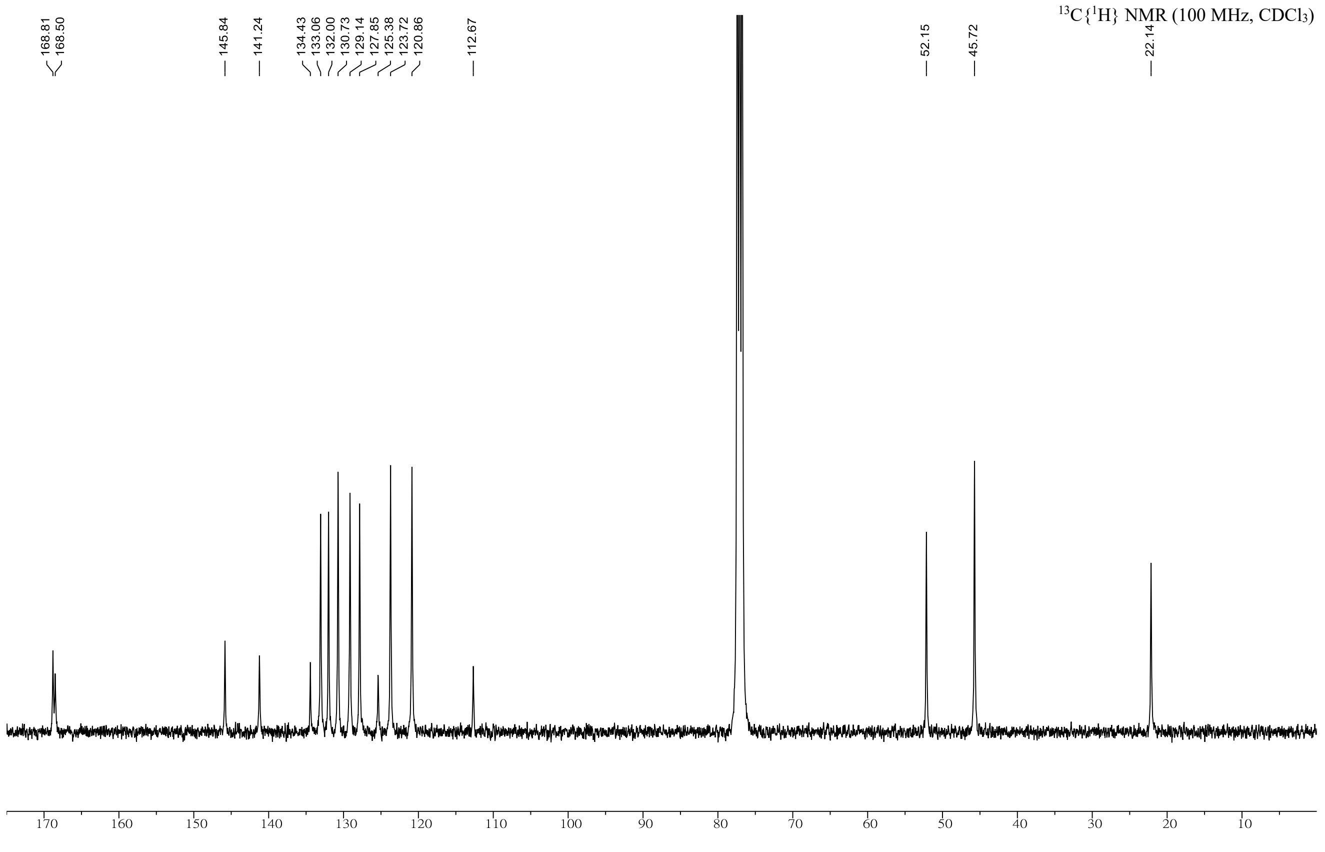

Methyl 2-[2-(2-Bromophenyl)acetamido]-4-nitrobenzoate (4l)

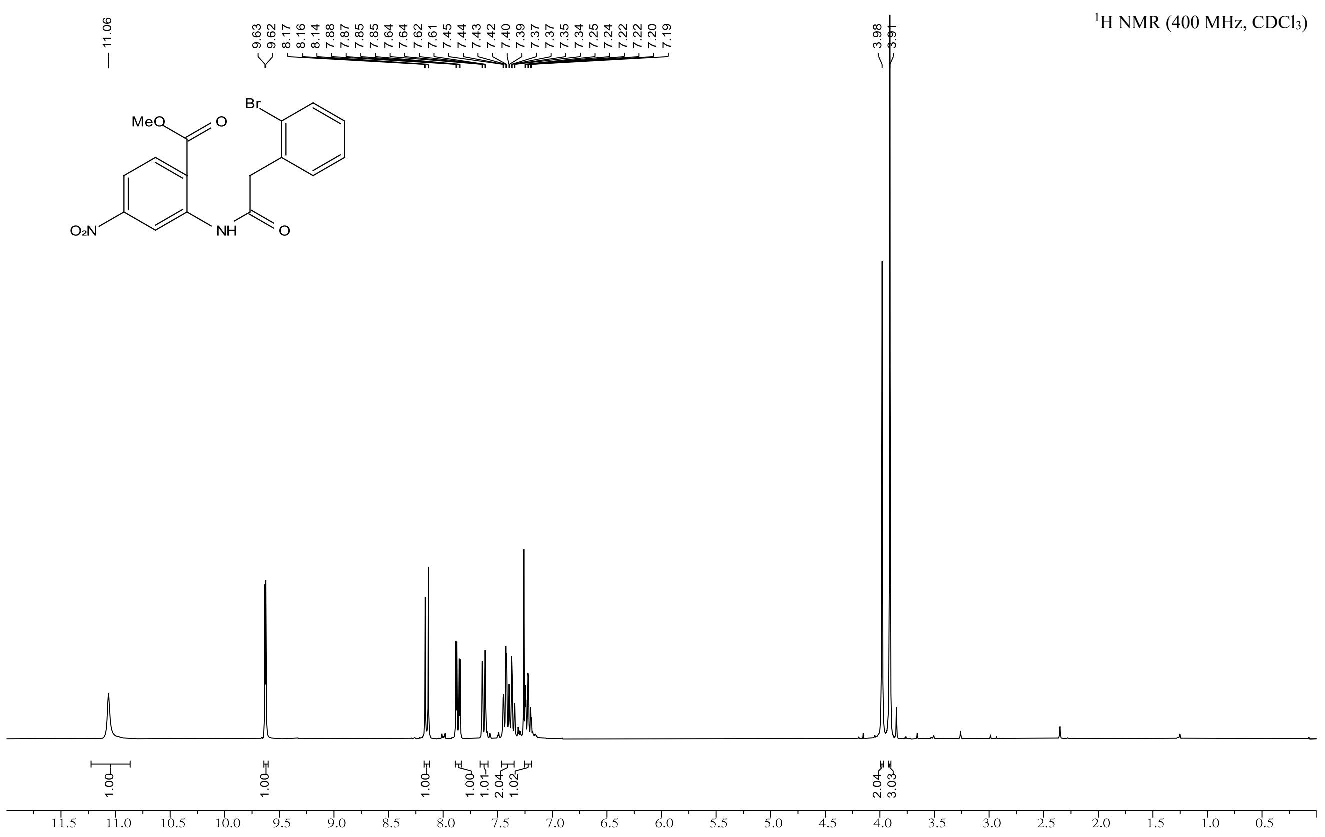

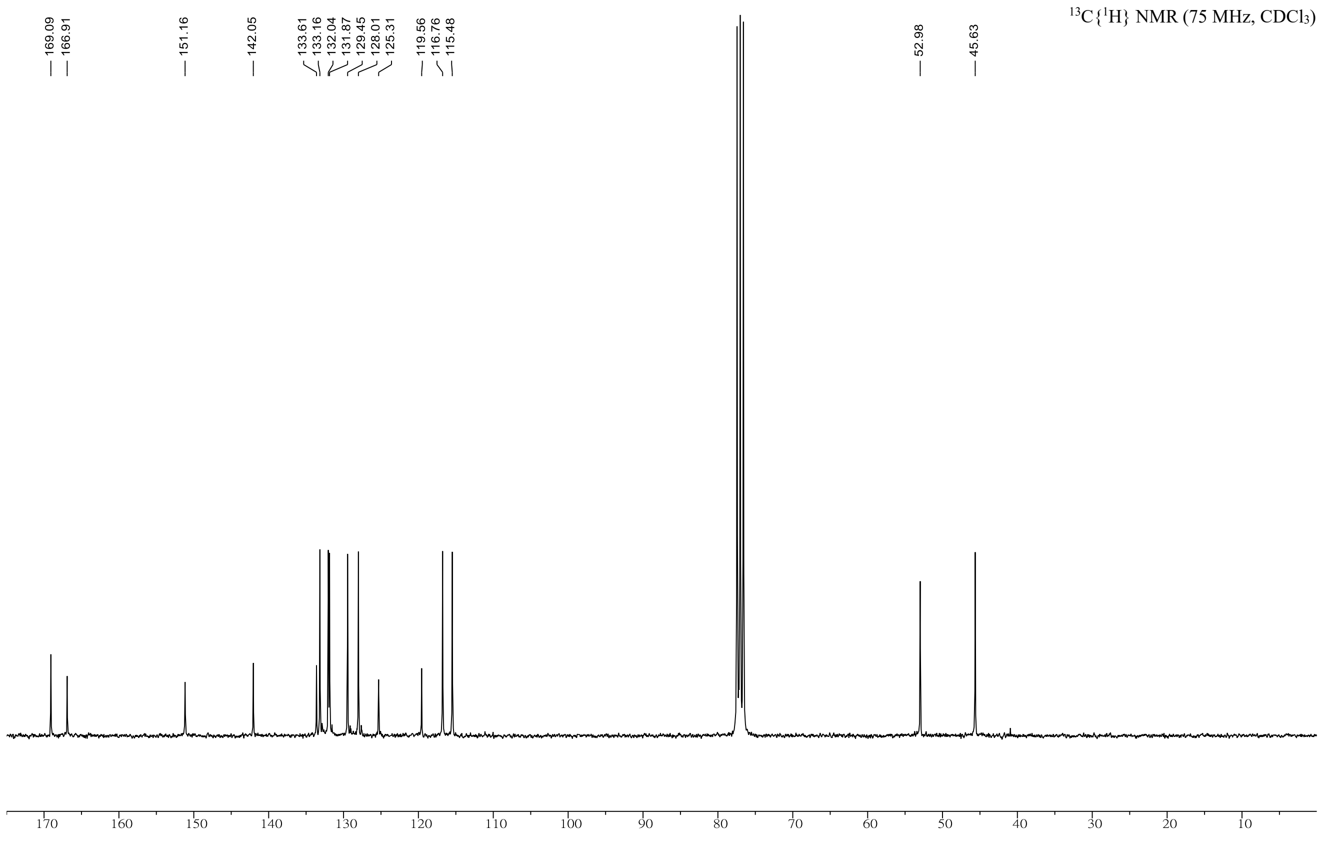

Methyl 2-[2-(2-Bromophenyl)acetamido]-4-methoxybenzoate (4m)

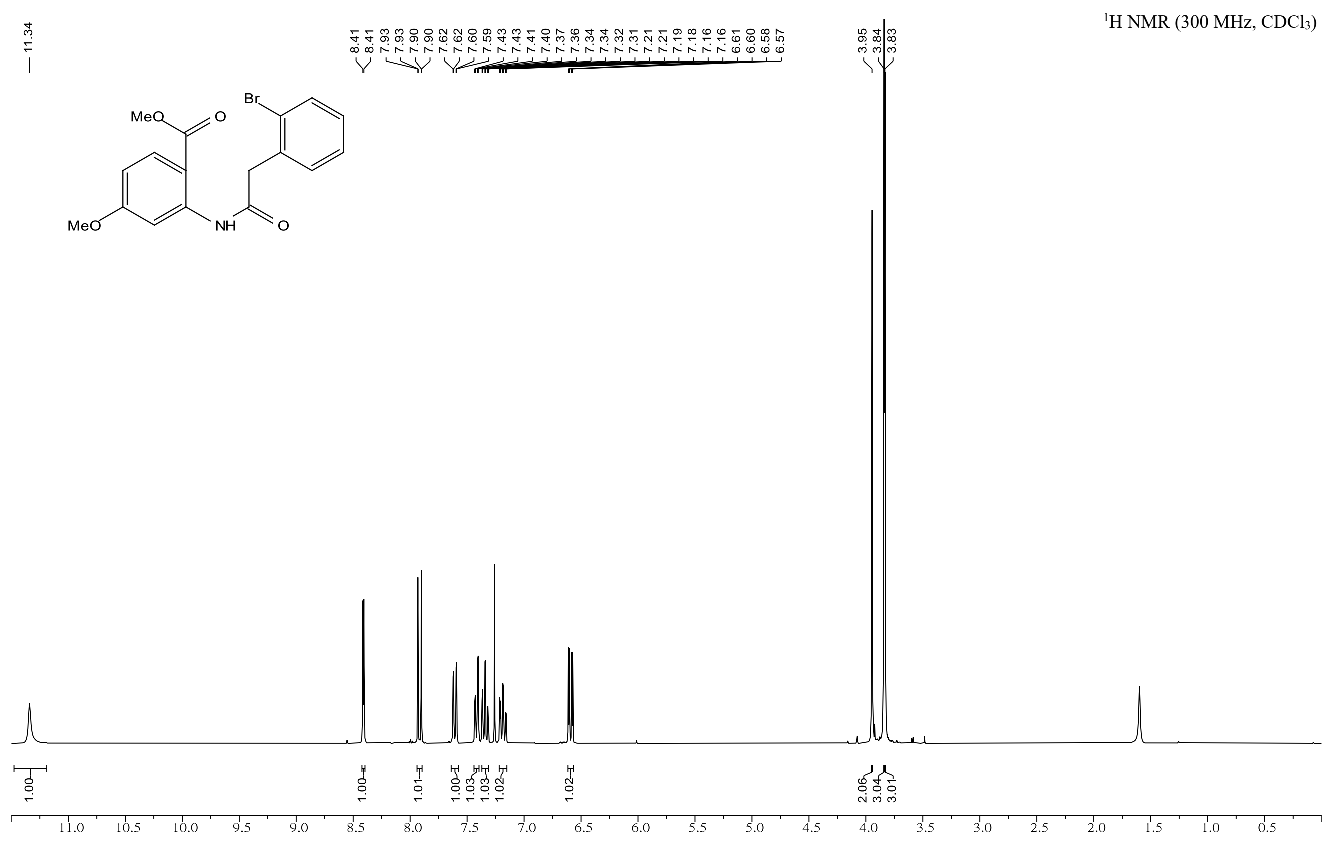

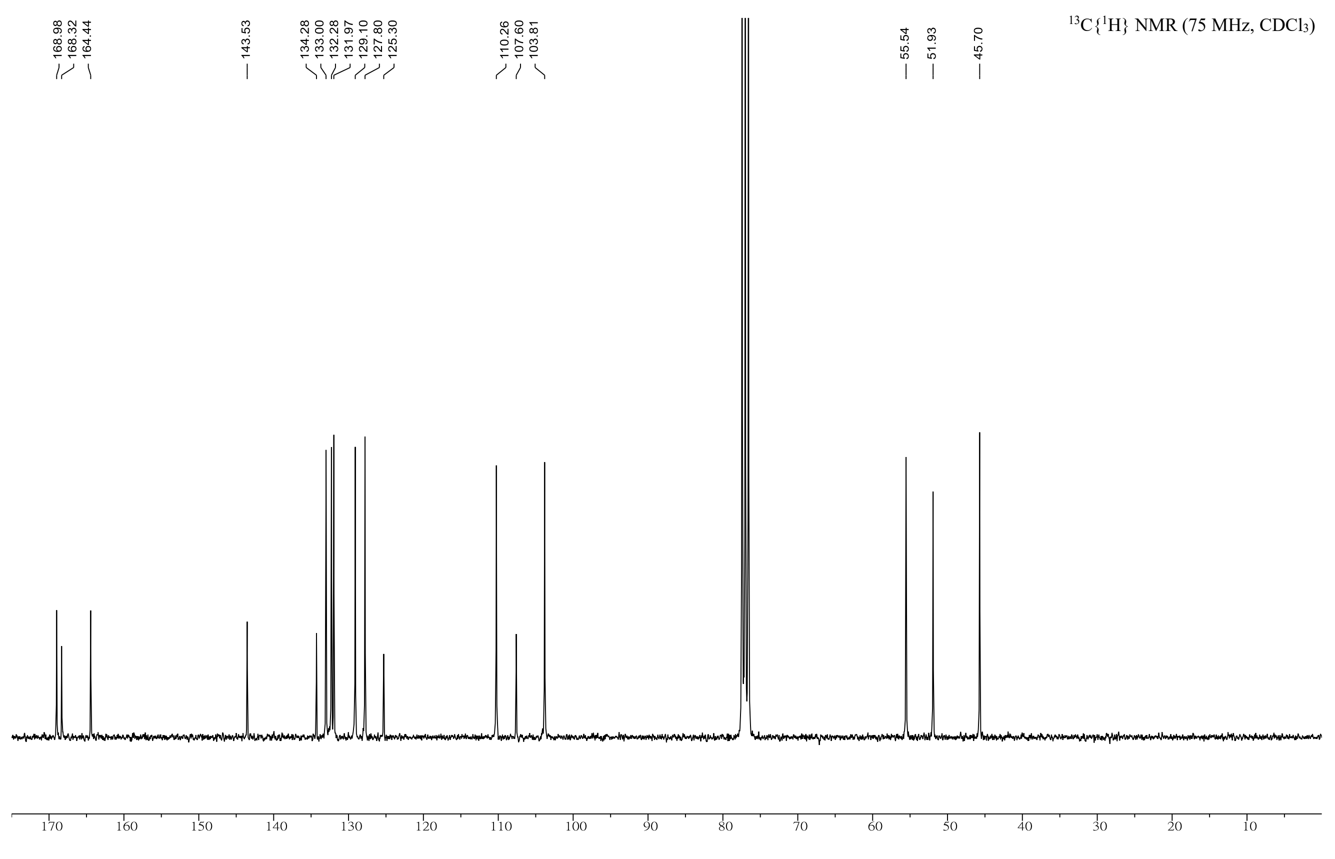

Methyl 2-[2-(2-Bromophenyl)acetamido]-4,5-dimethoxybenzoate (4n)

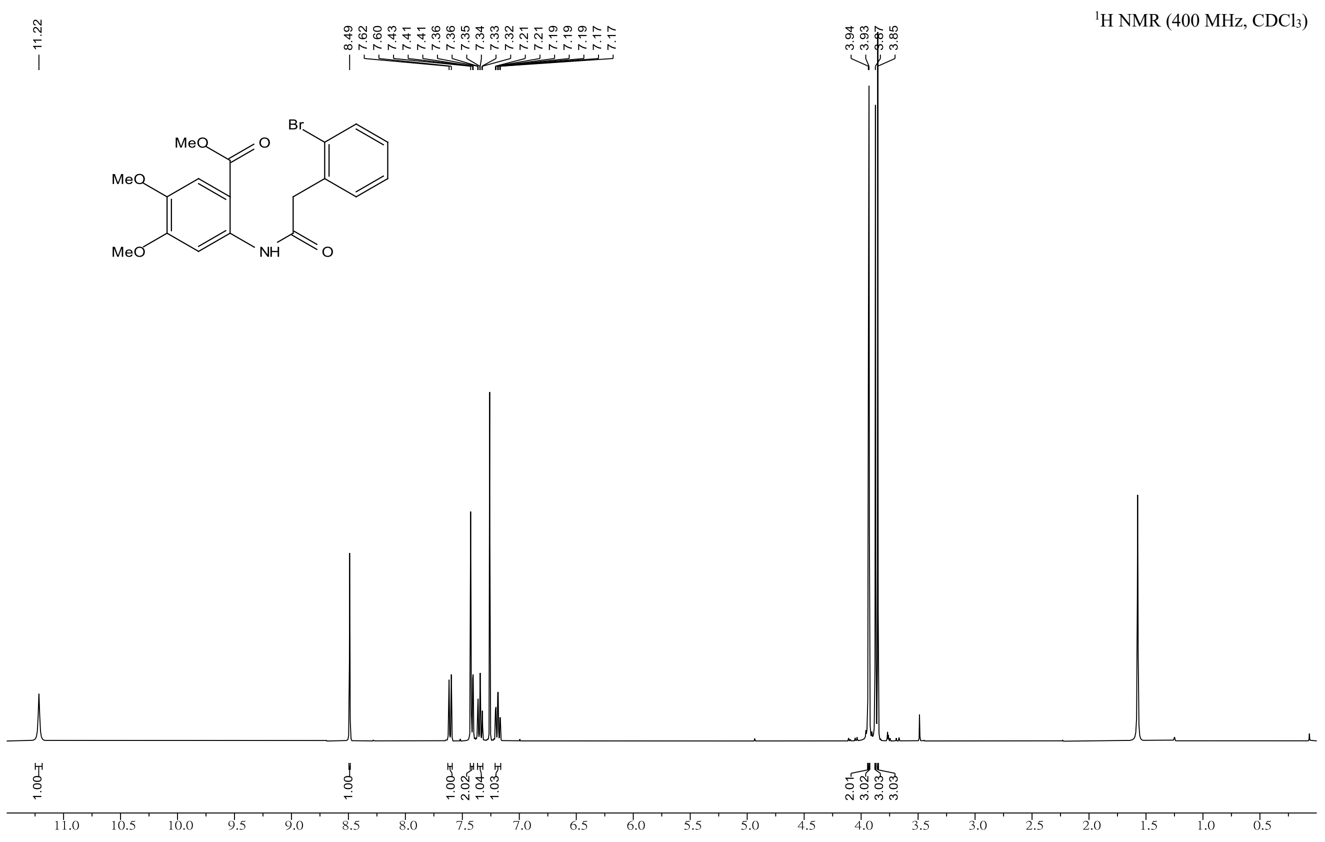

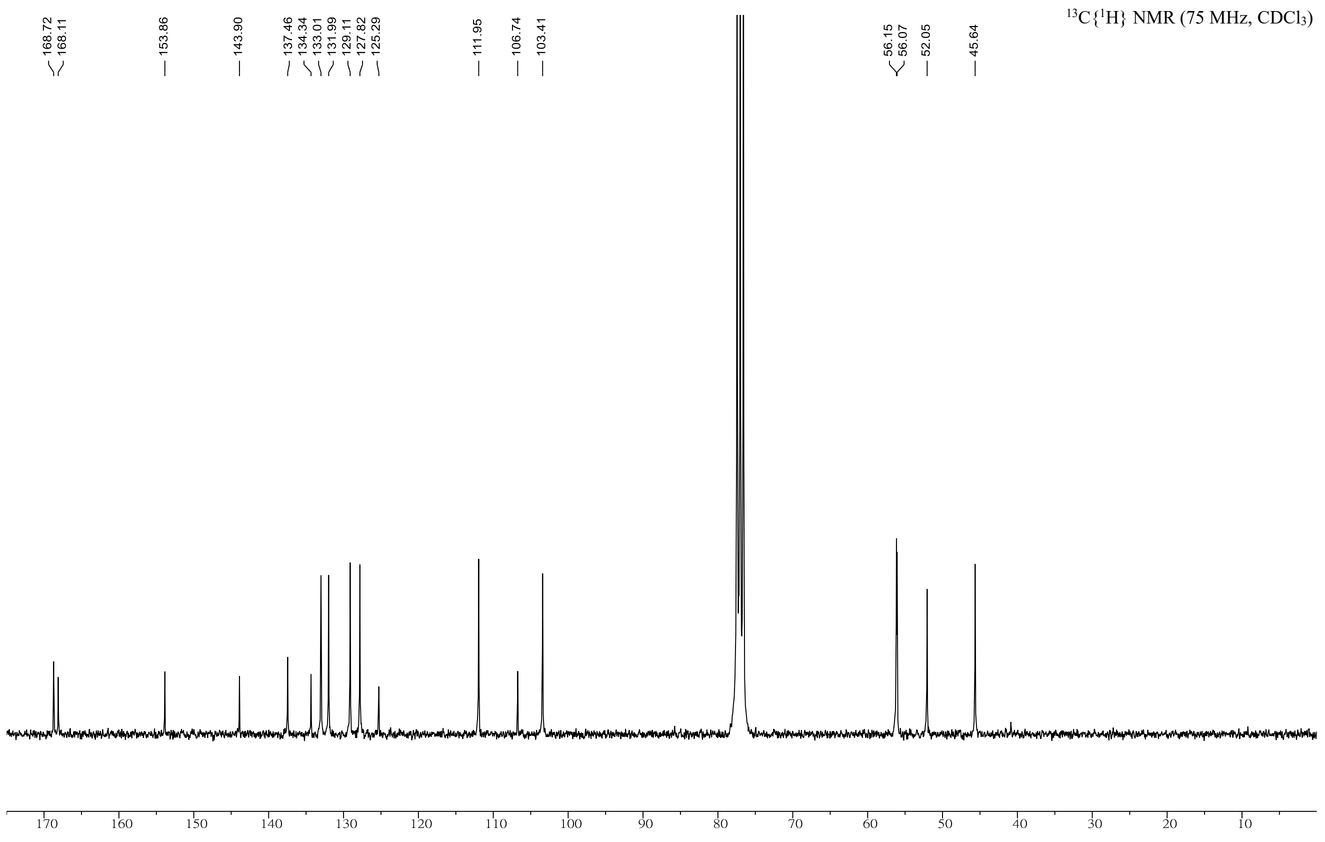

Methyl 2-[2-(2-Bromo-4-fluorophenyl)acetamido]benzoate (4o)

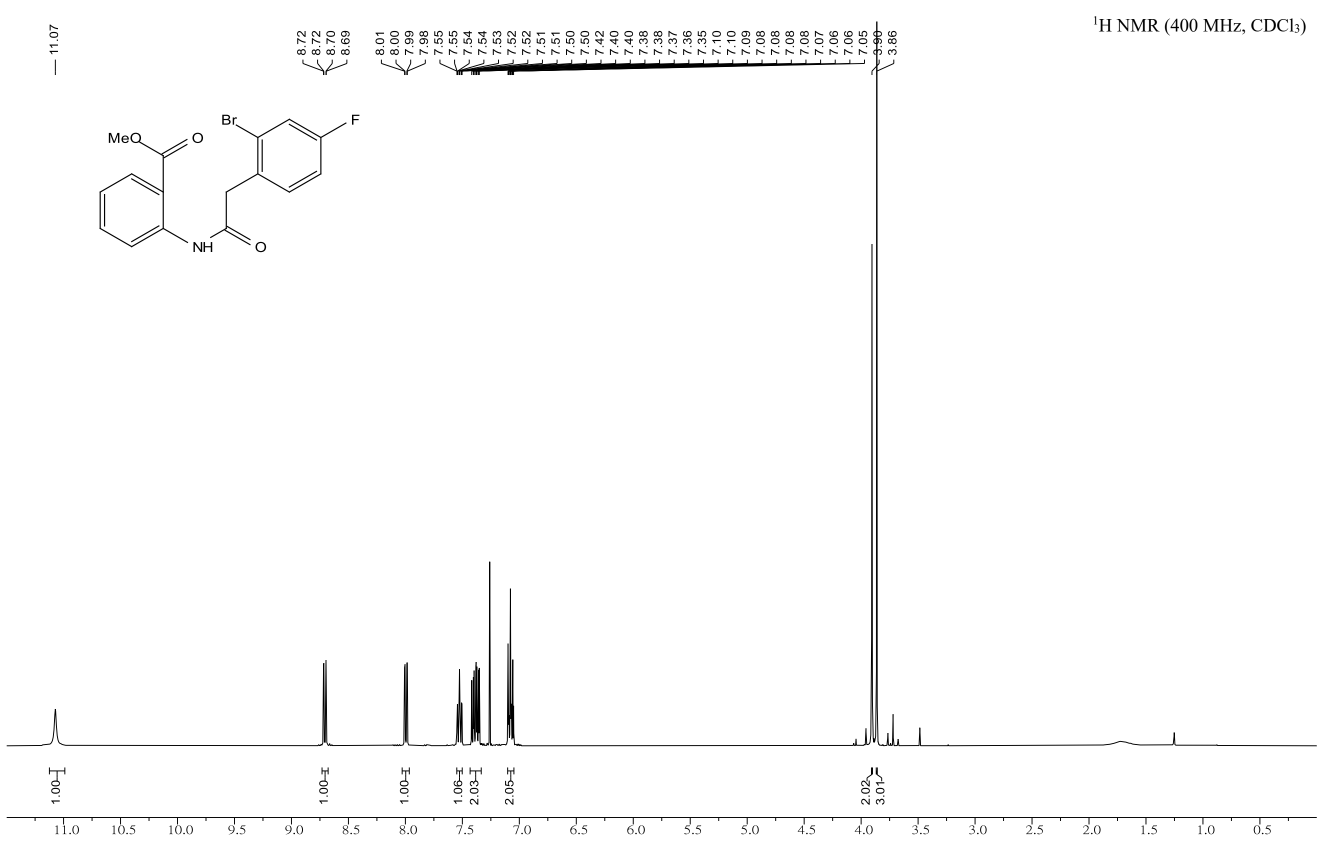

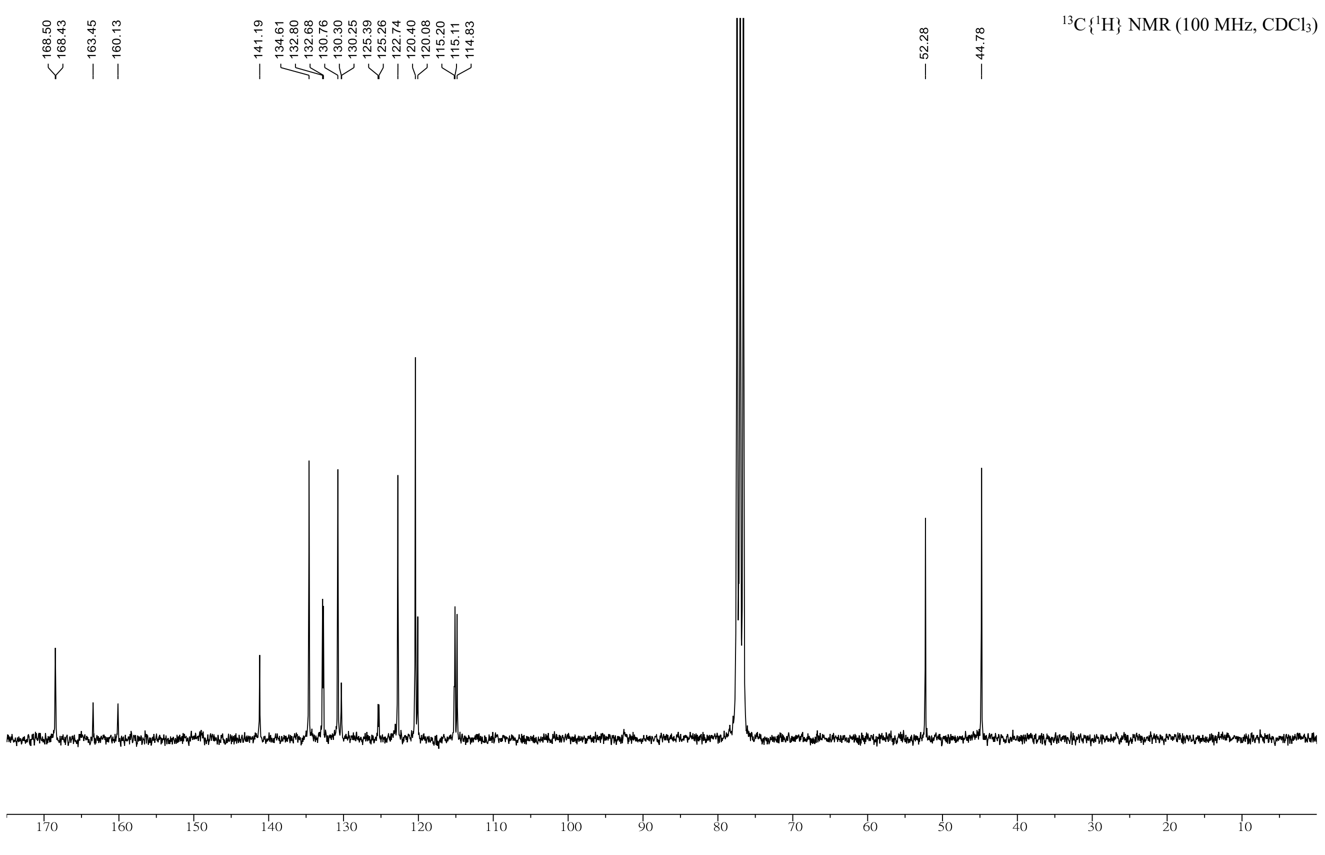

Methyl 2-[2-(2-Bromo-4-chlorophenyl)acetamido]benzoate (4p)

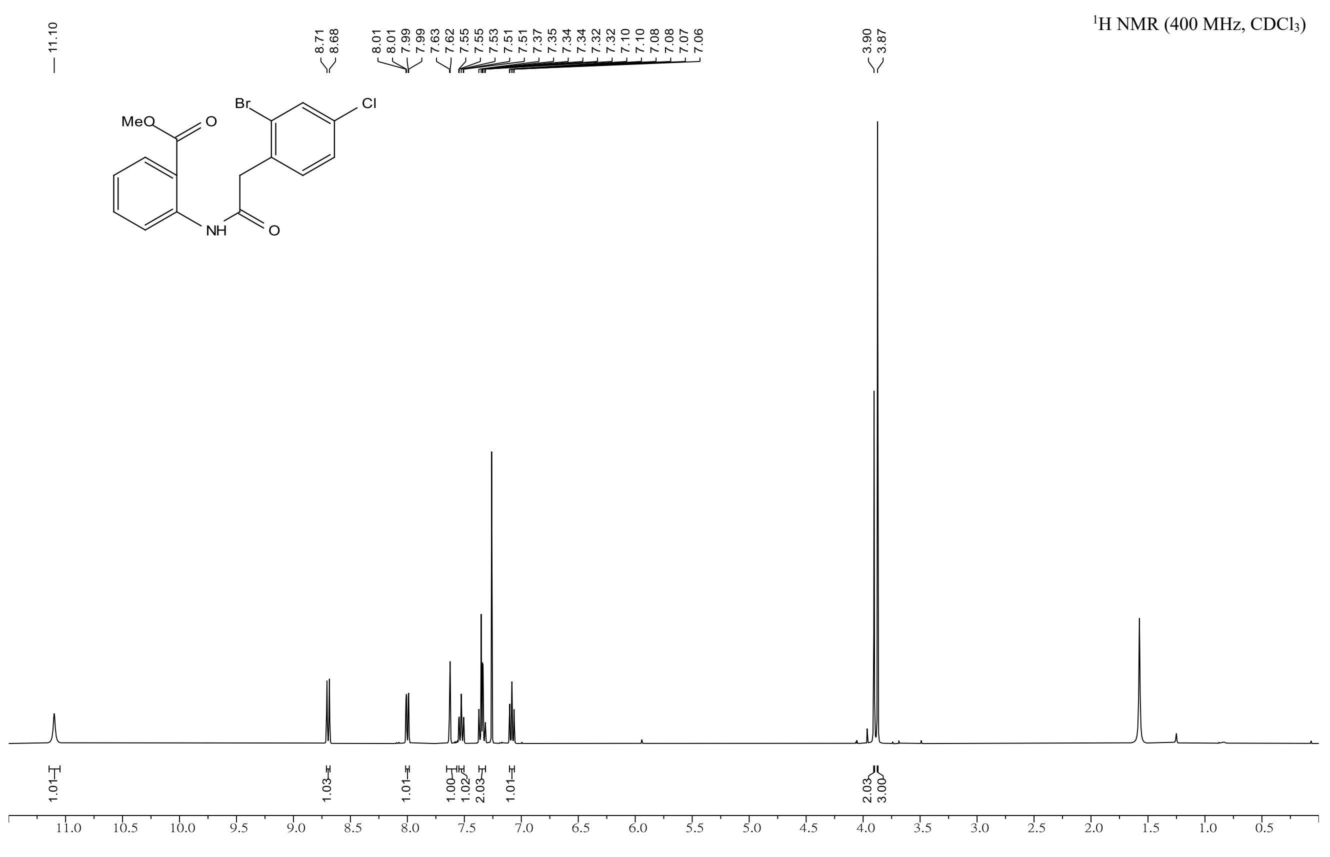

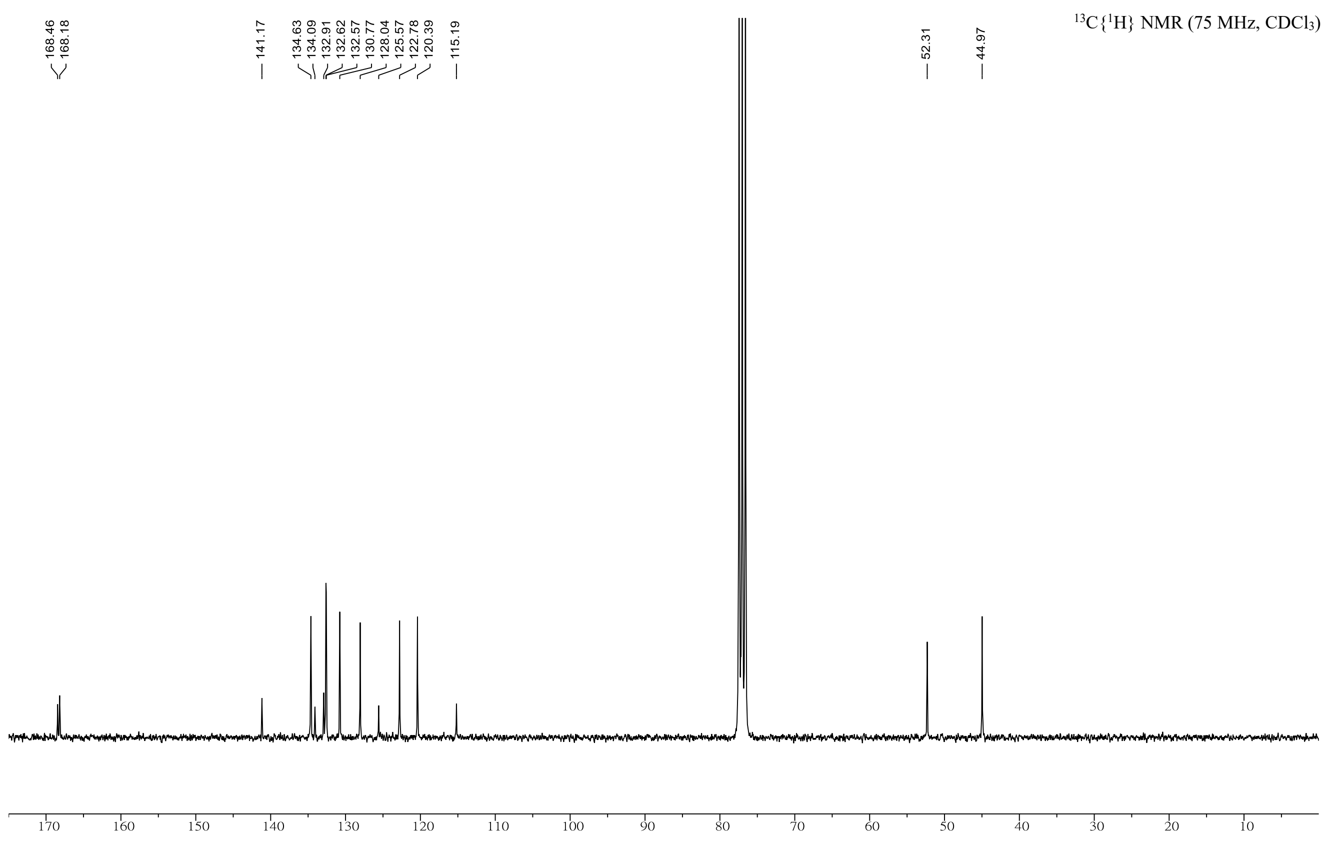

Methyl 2-[2-(2,4-Dibromophenyl)acetamido]benzoate (4q)

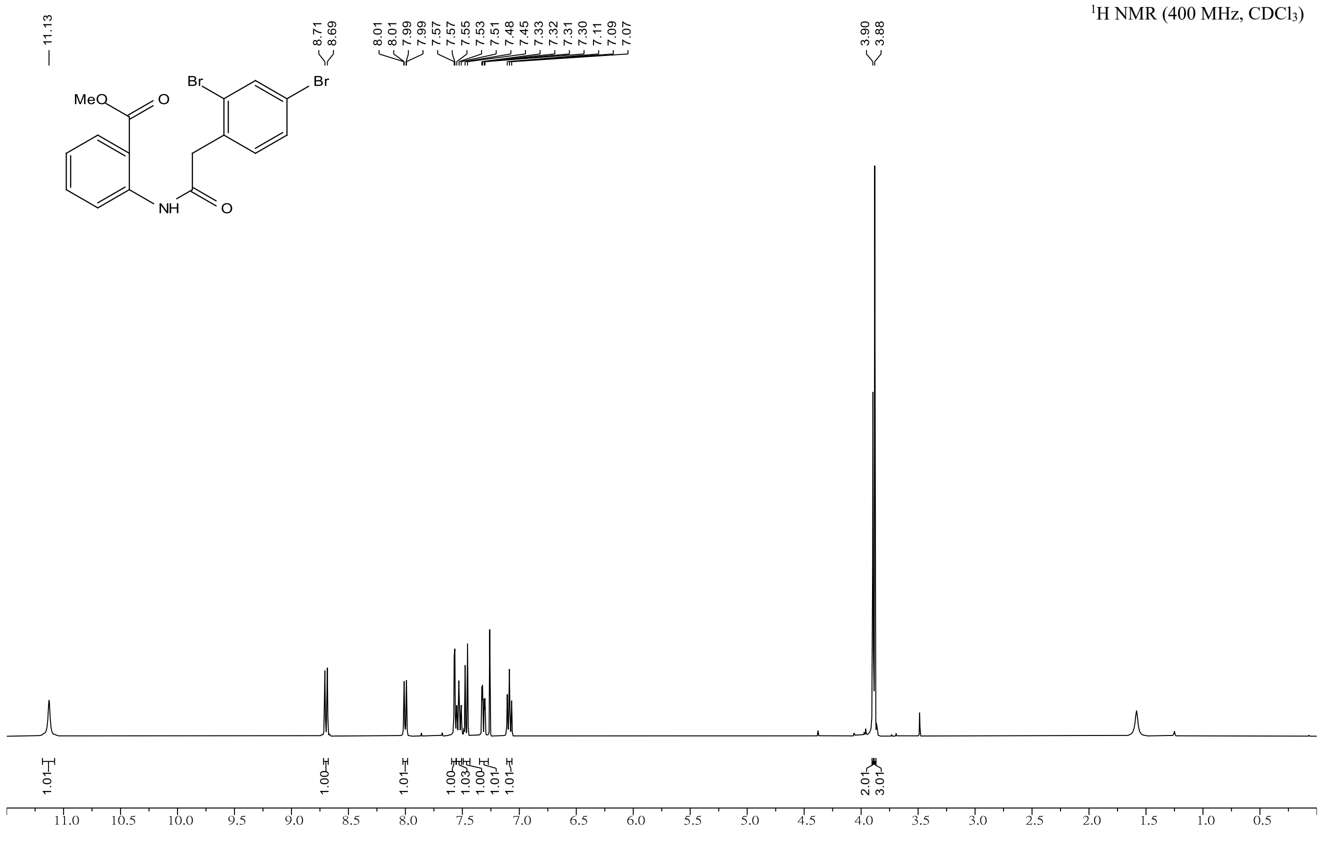

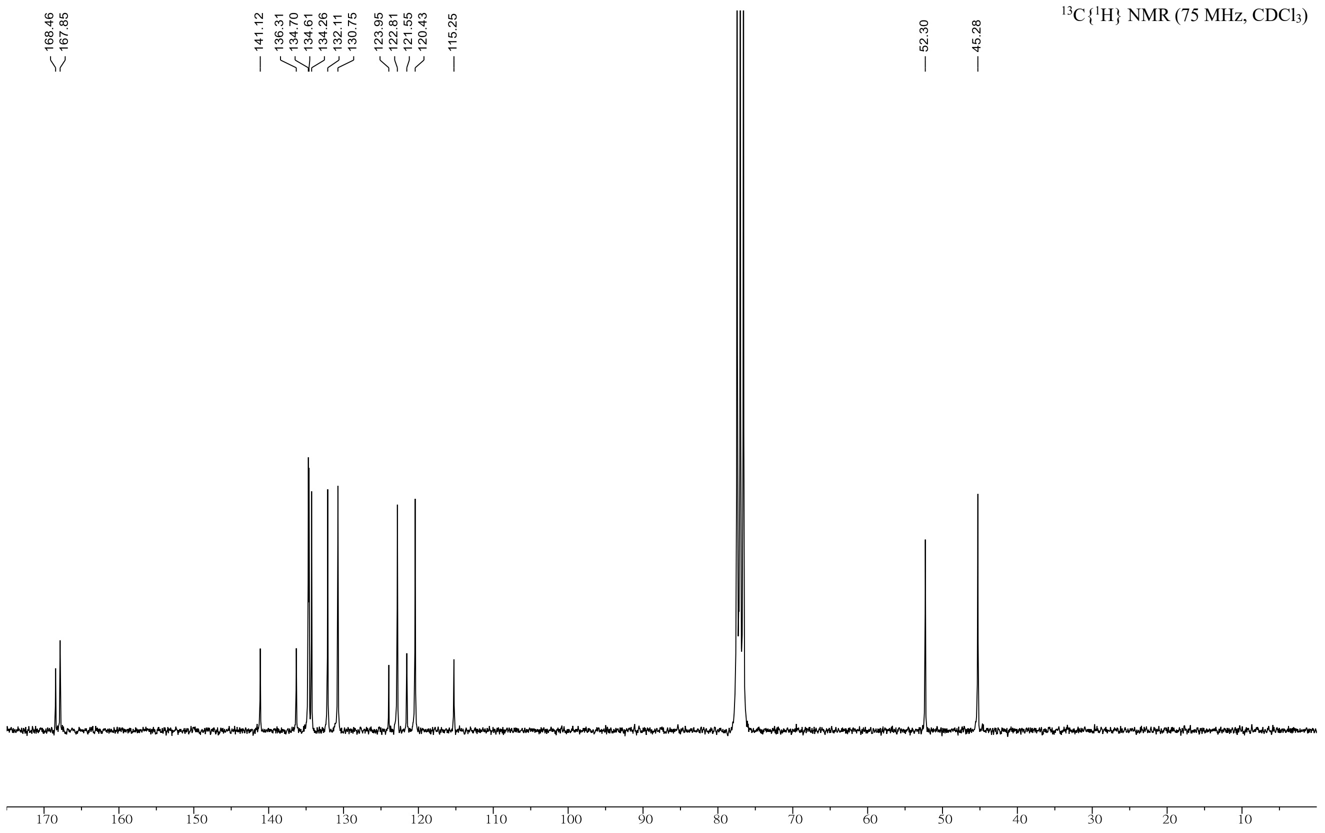

Methyl 2-[2-(2-Bromo-4-nitrophenyl)acetamido]benzoate (4r)

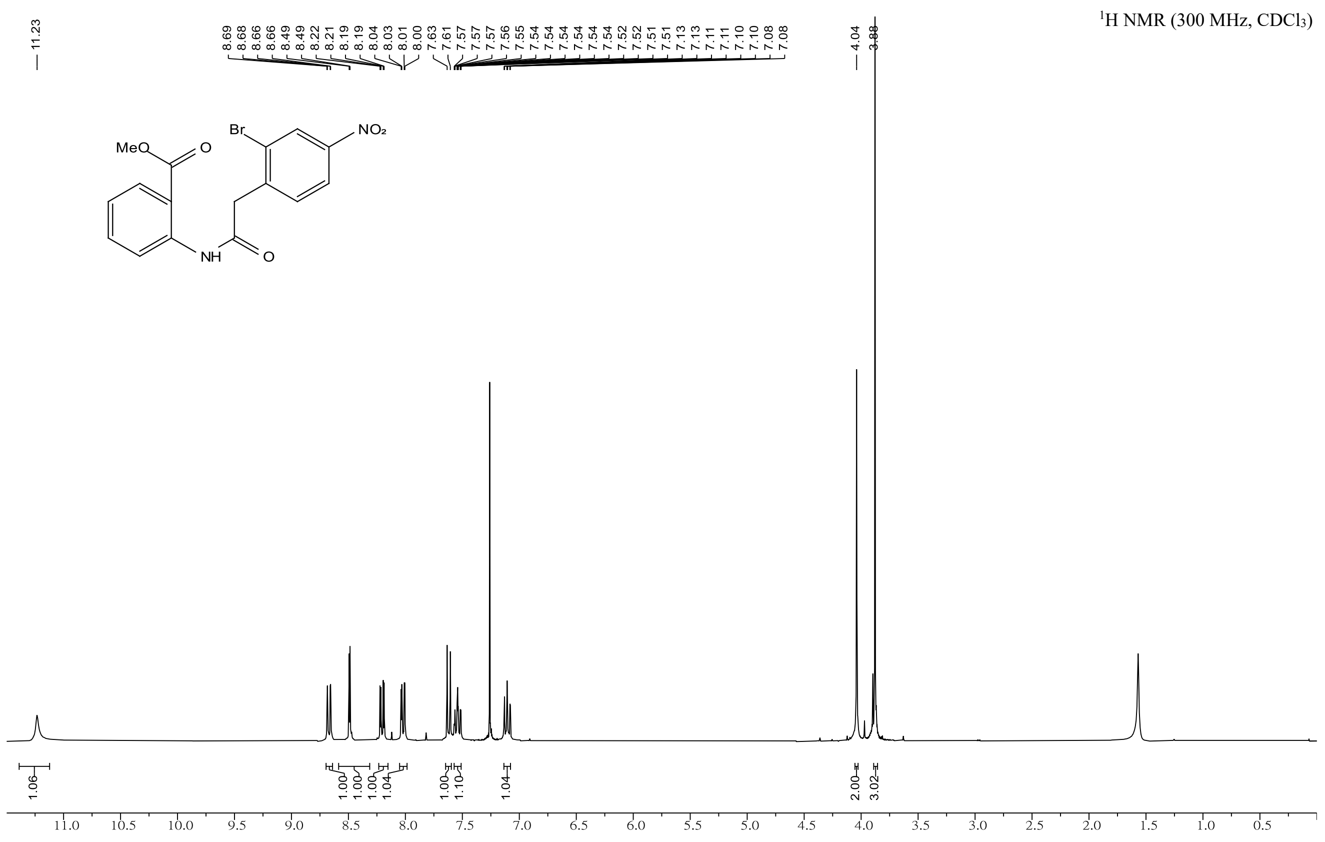

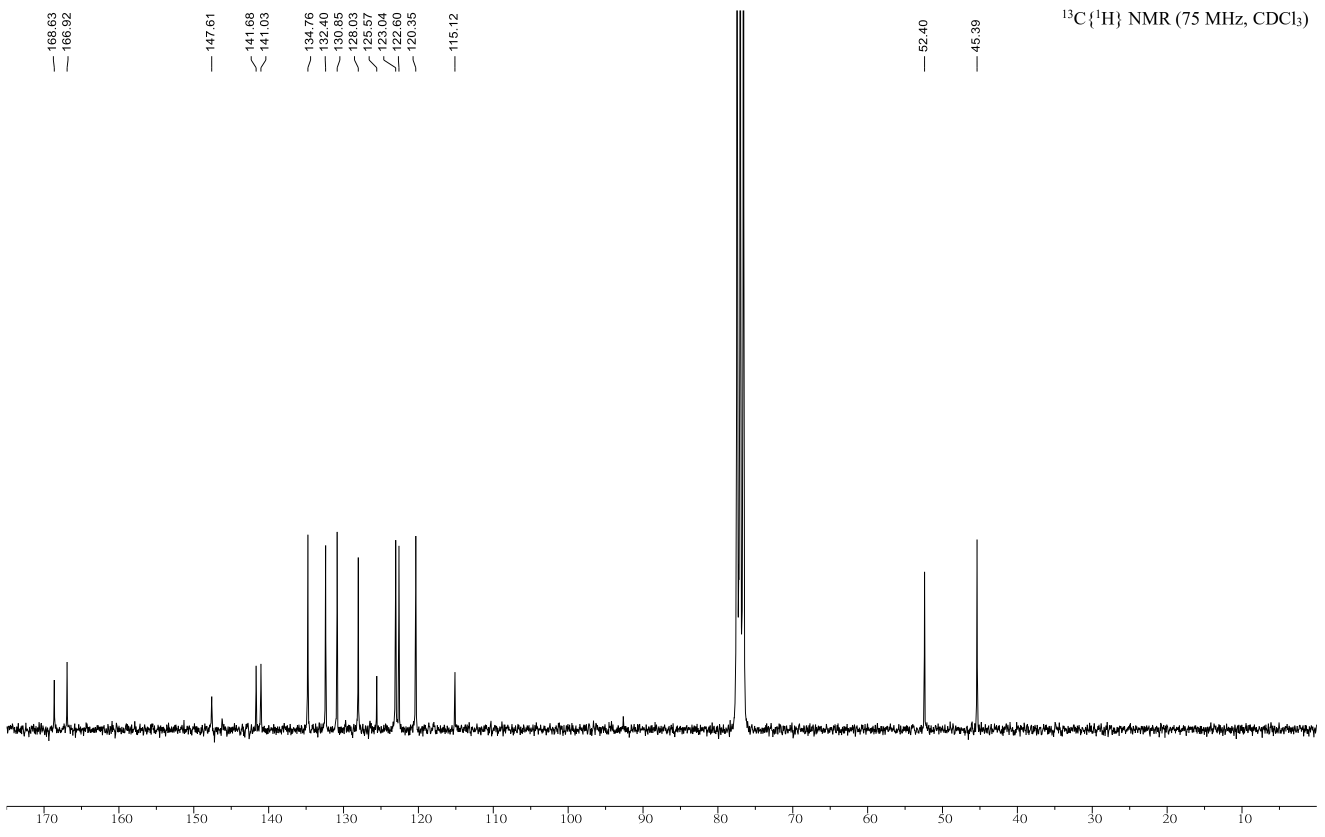

Methyl 2-[2-(2-Bromo-5-fluorophenyl)acetamido]benzoate (4s)

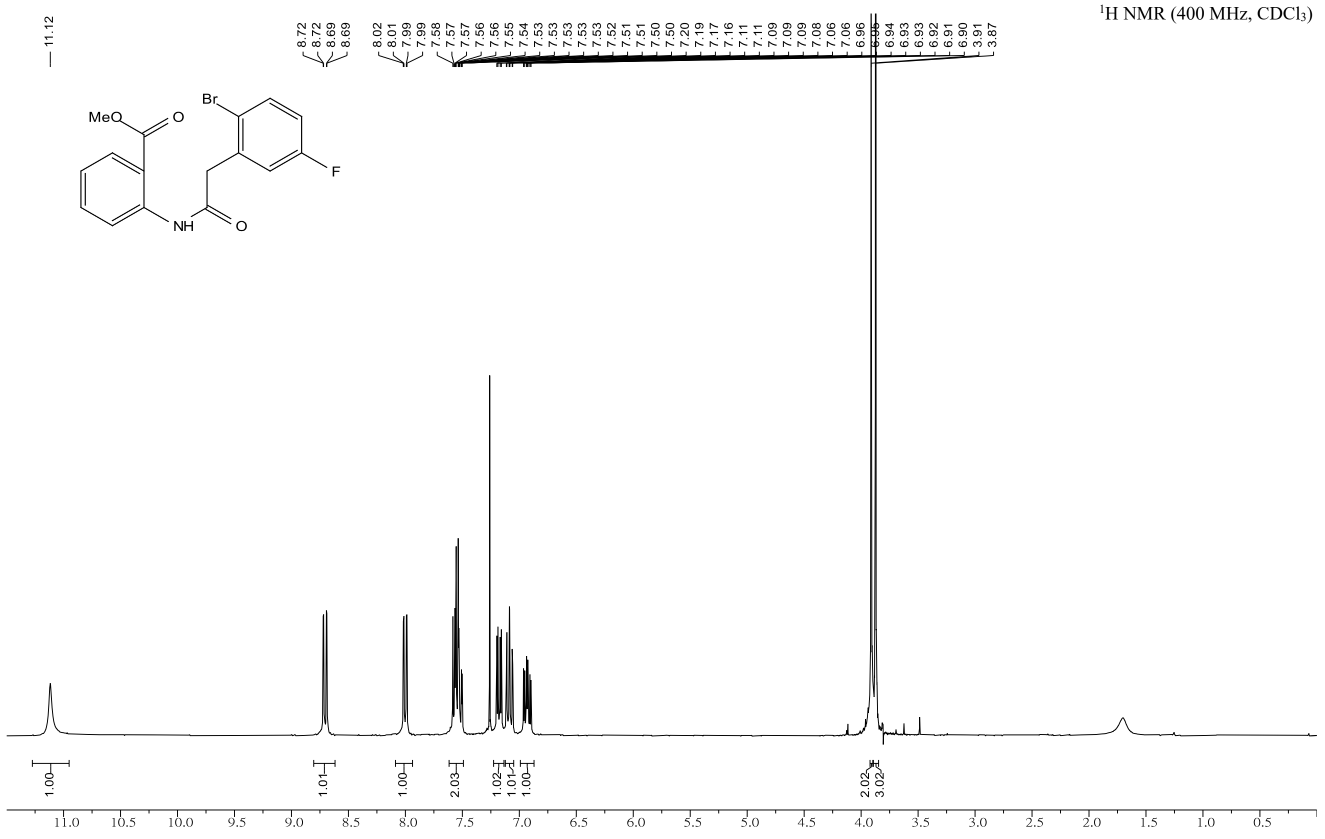

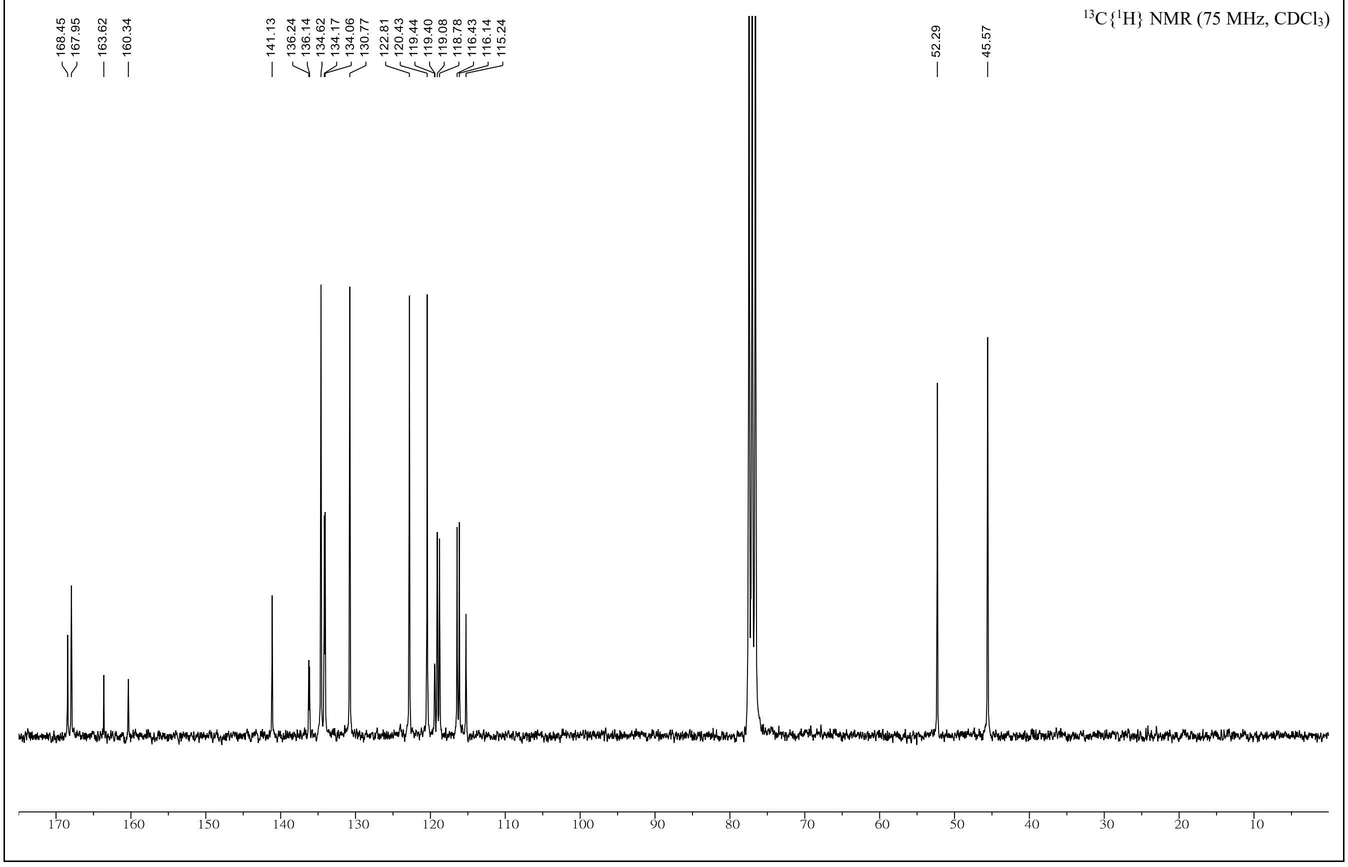

Methyl 2-[2-(2-Bromo-5-chlorophenyl)acetamido]benzoate (4t)

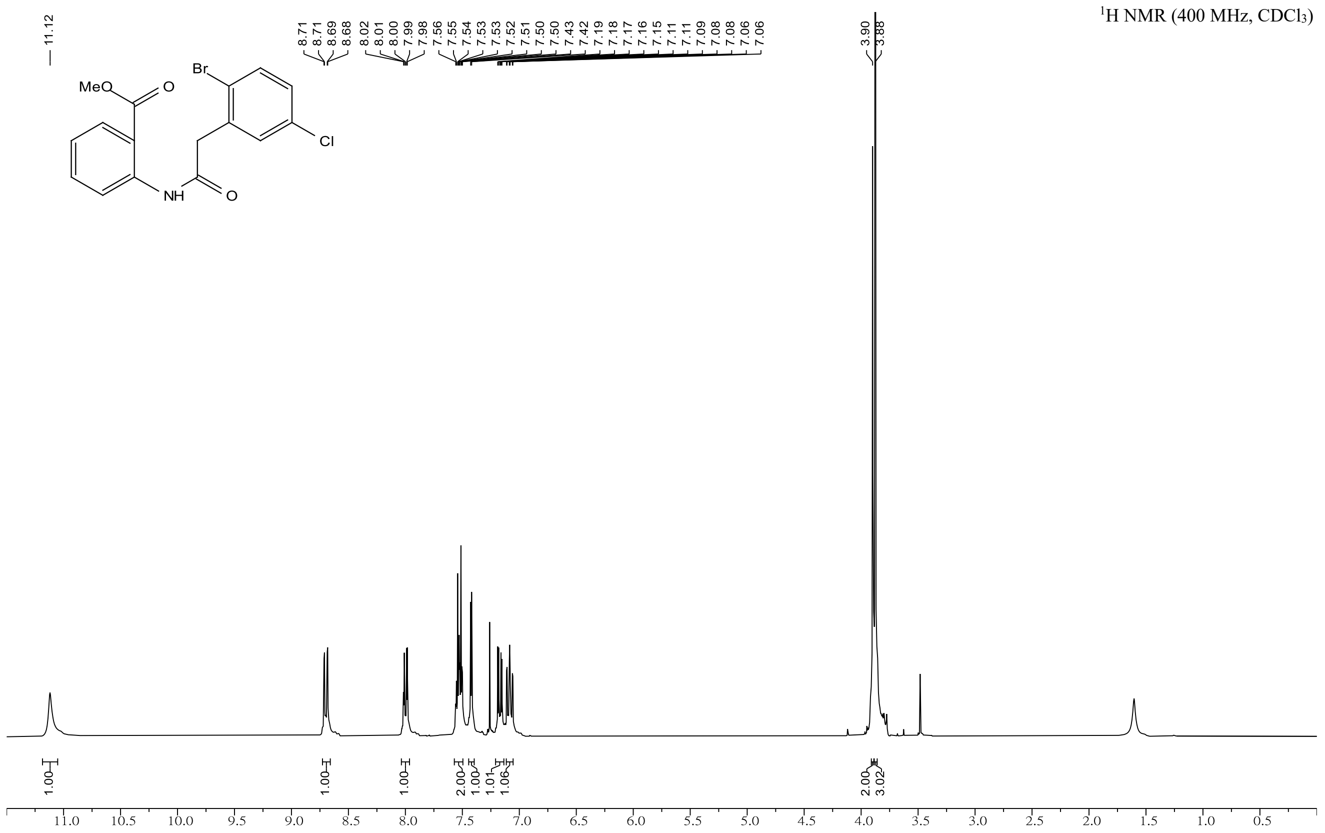

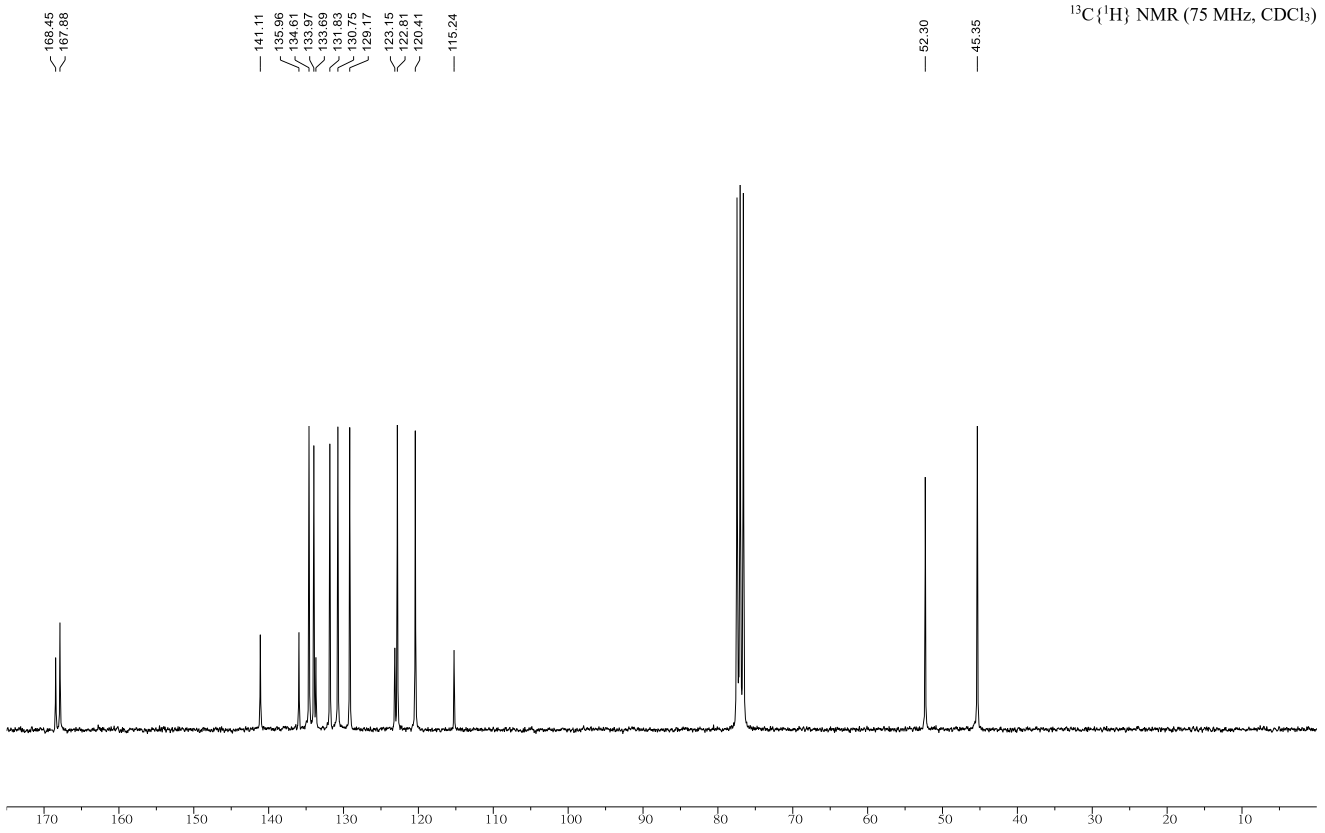

Methyl 2-[2-(2-Bromo-4,5-dimethoxyphenyl)acetamido]benzoate (4u)

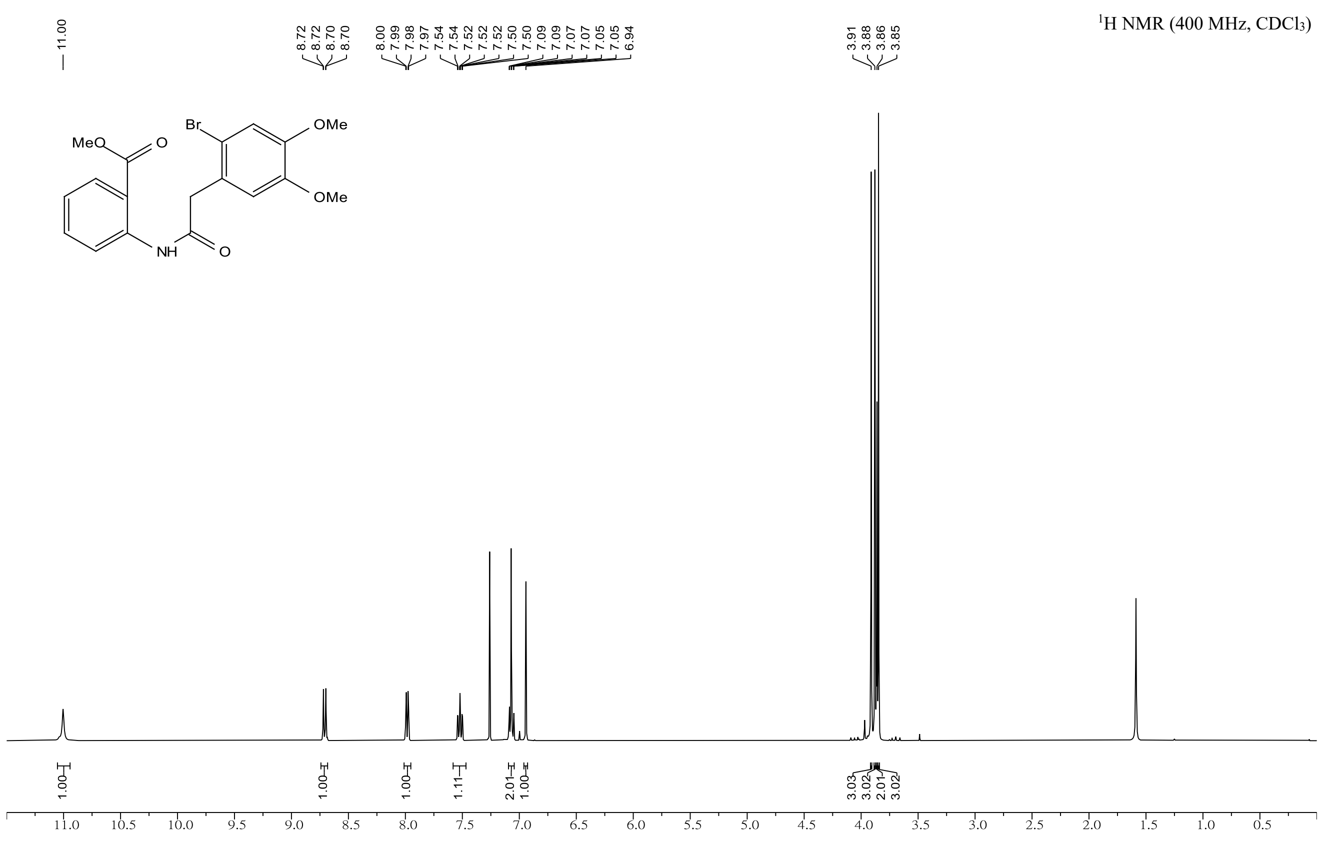

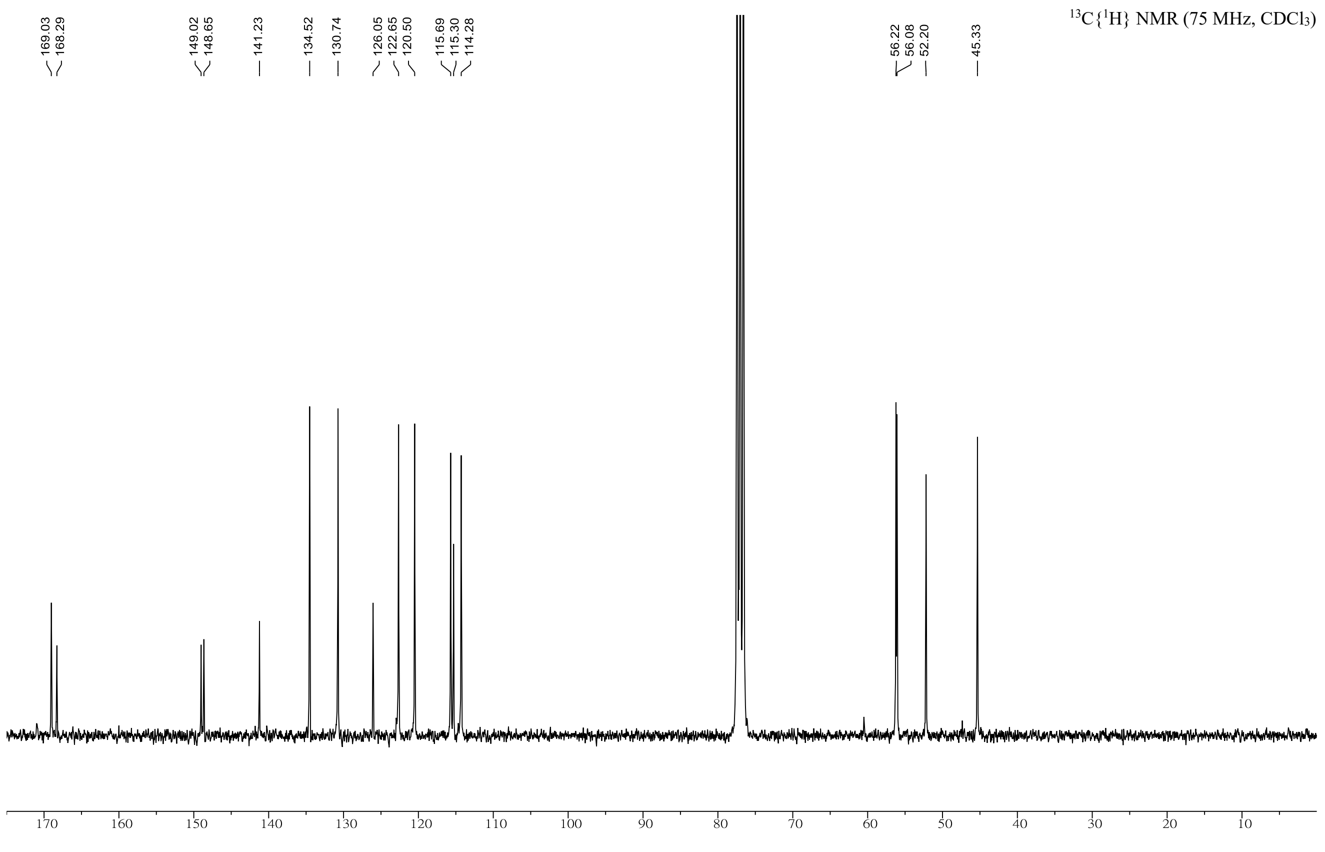

Methyl 2-[2-(6-Bromobenzo[*d*][1,3]dioxol-5-yl)acetamido]benzoate (4v)

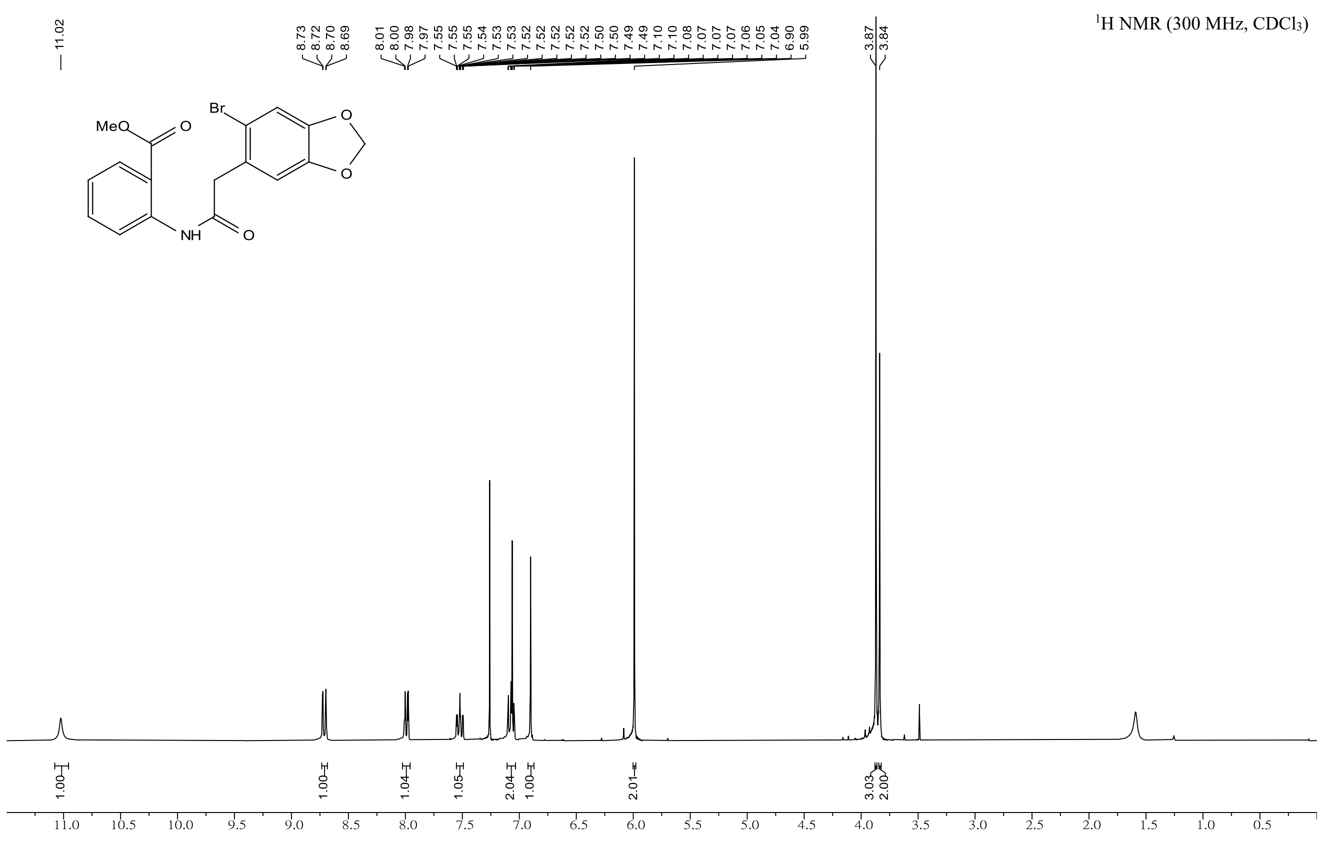

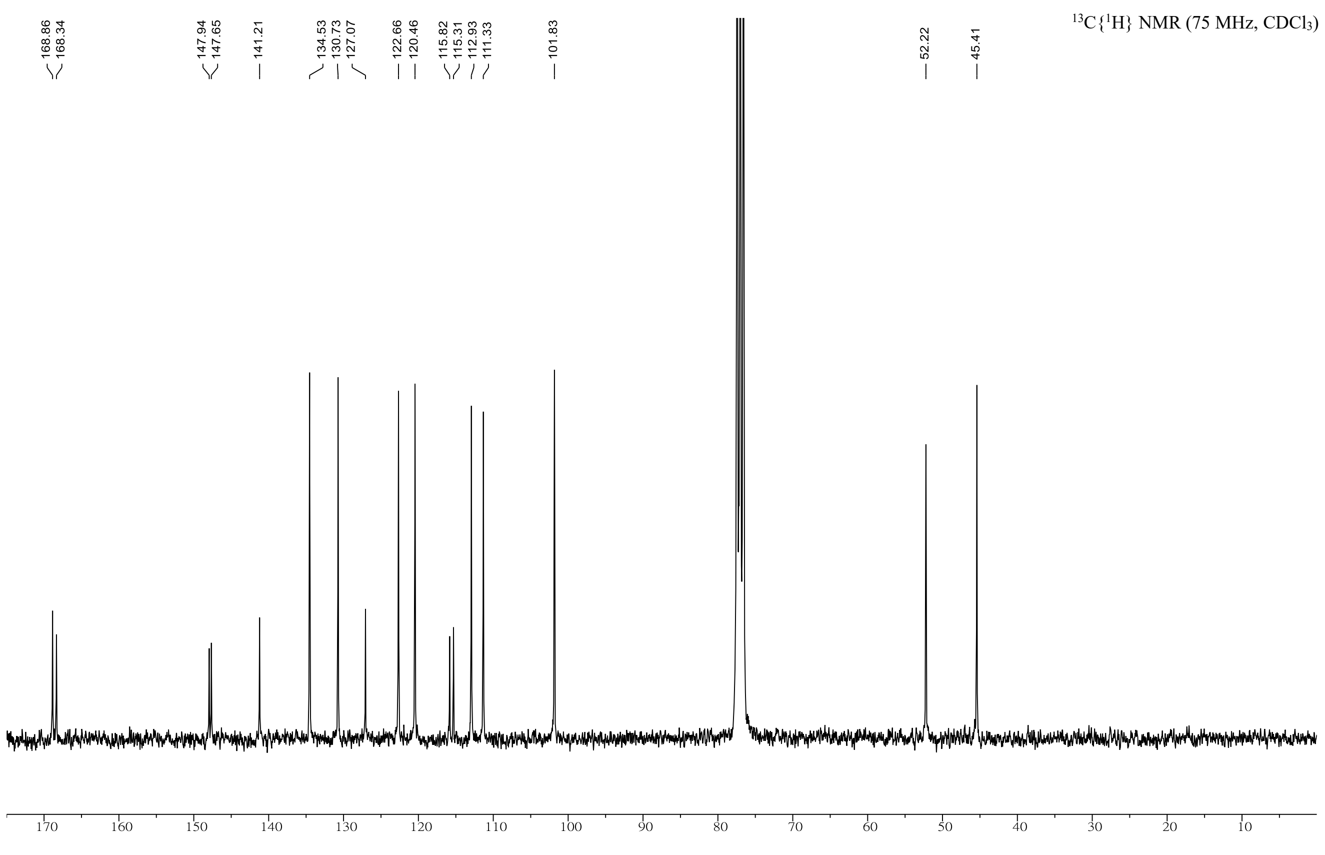

Methyl 2-[2-(2-Bromo-4-fluorophenyl)acetamido]-4-fluorobenzoate (4w)

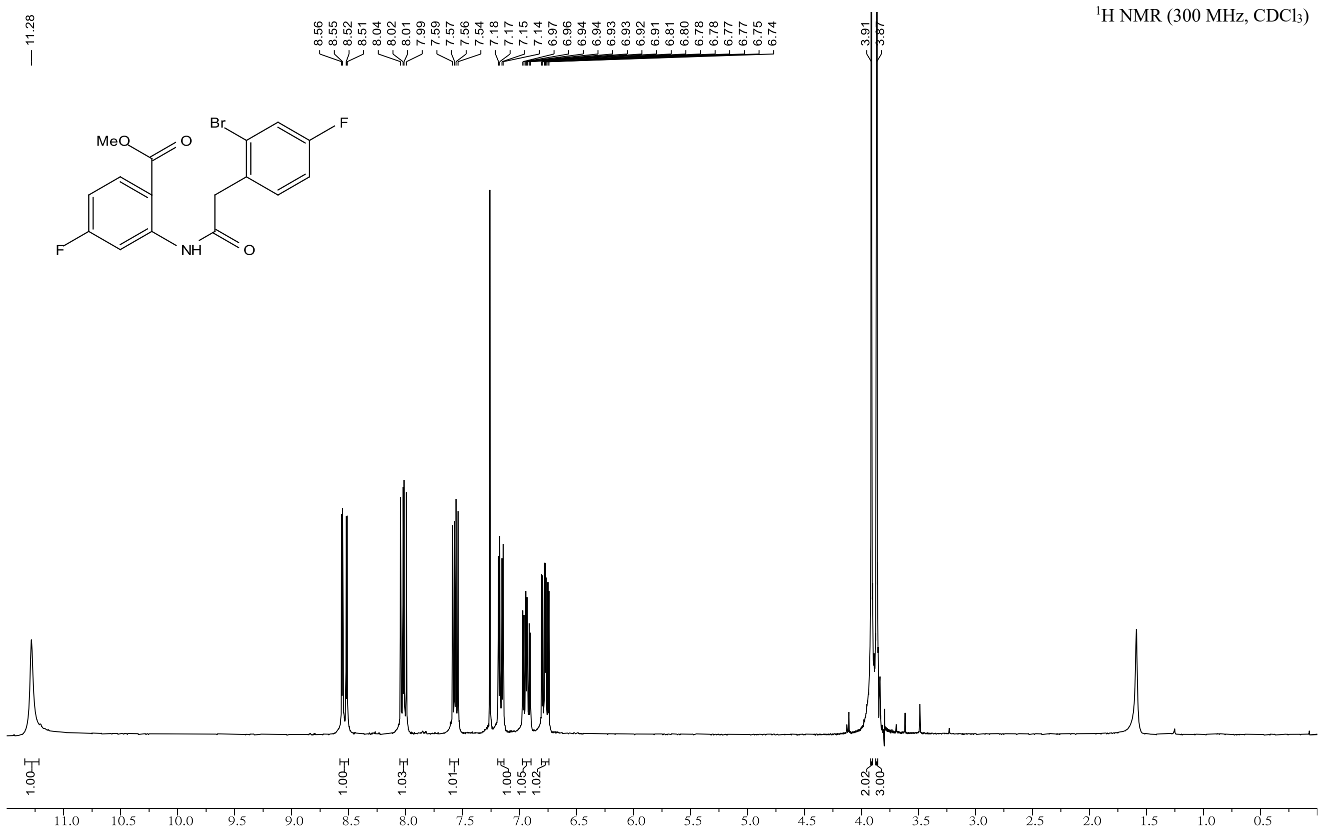

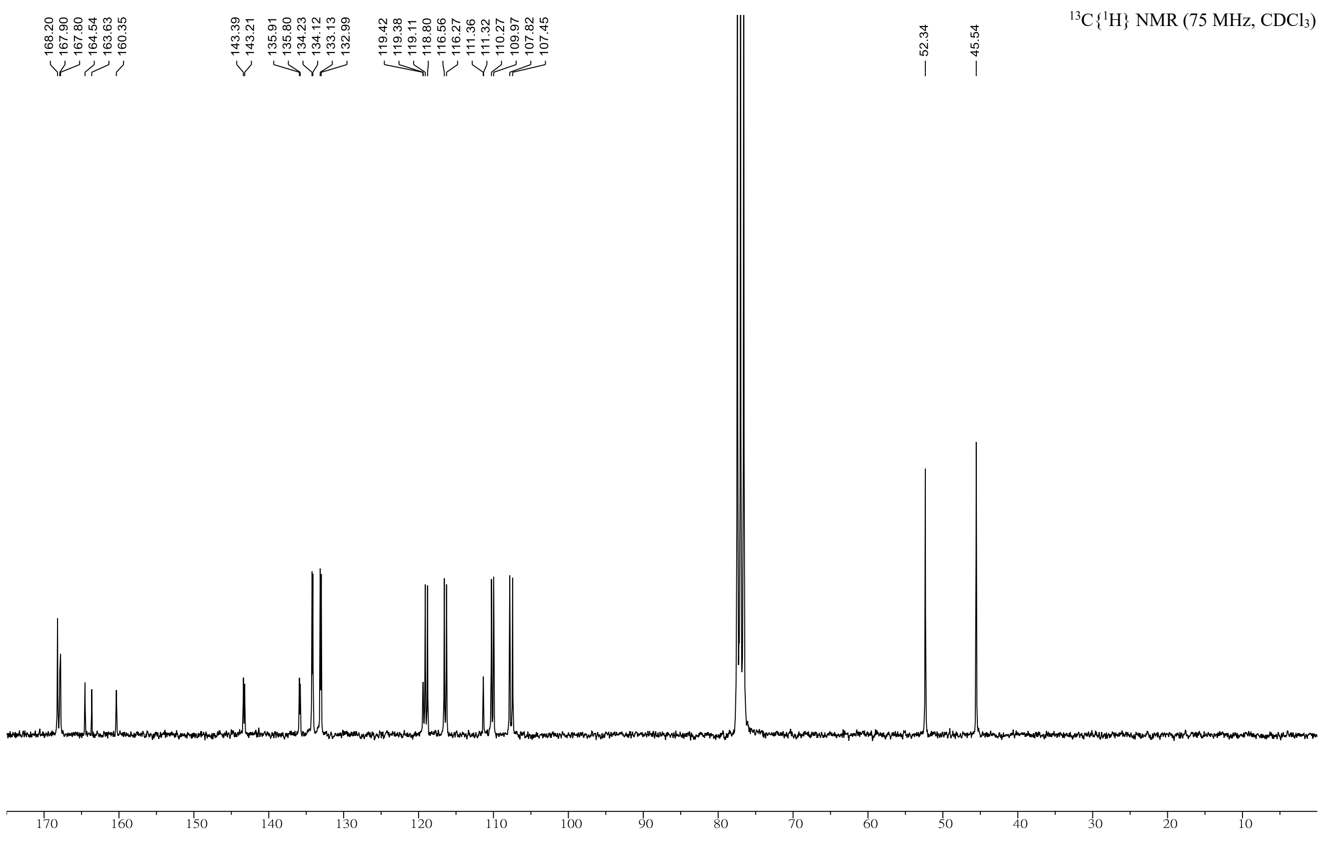

Methyl 2-[2-(2-Bromo-5-fluorophenyl)acetamido]-4-fluorobenzoate (4x)

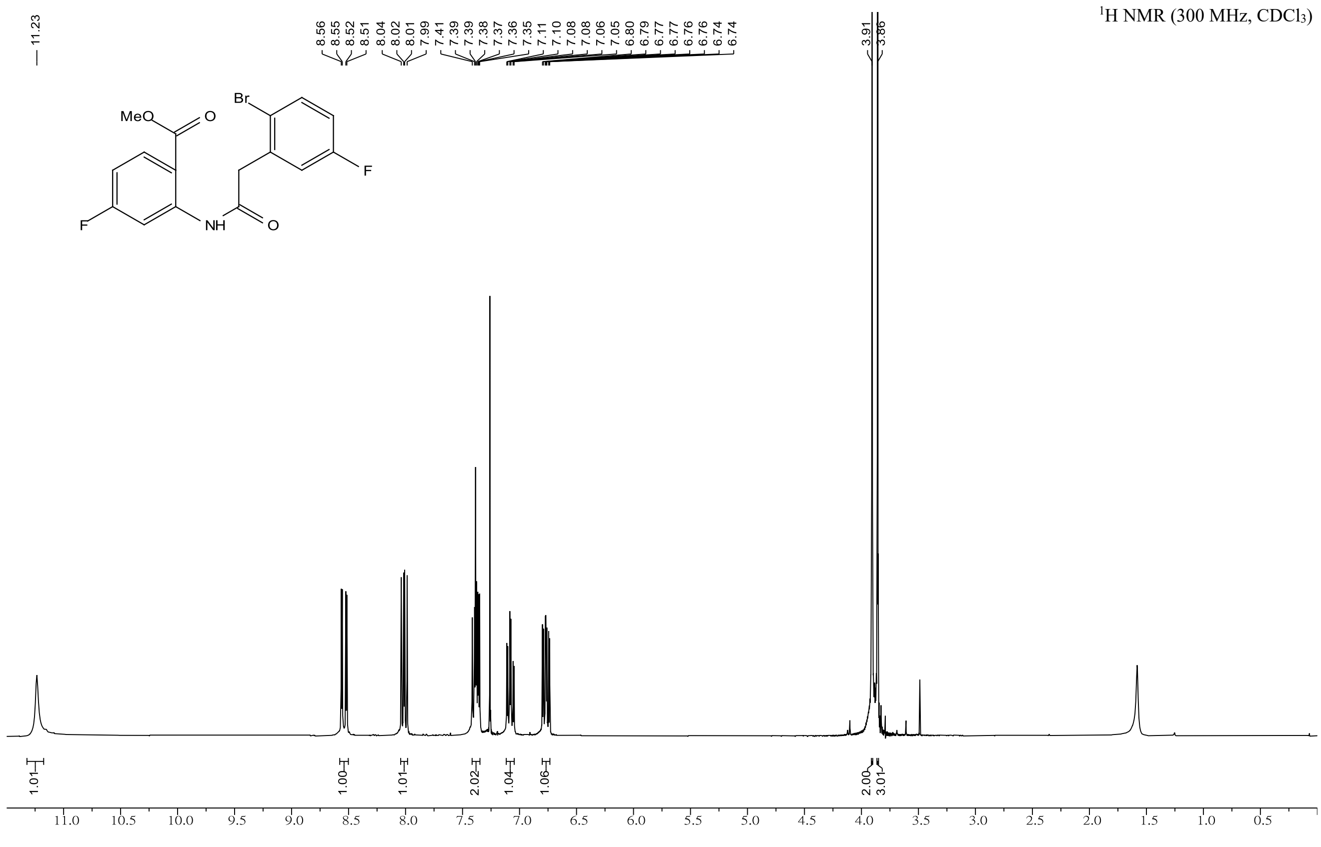

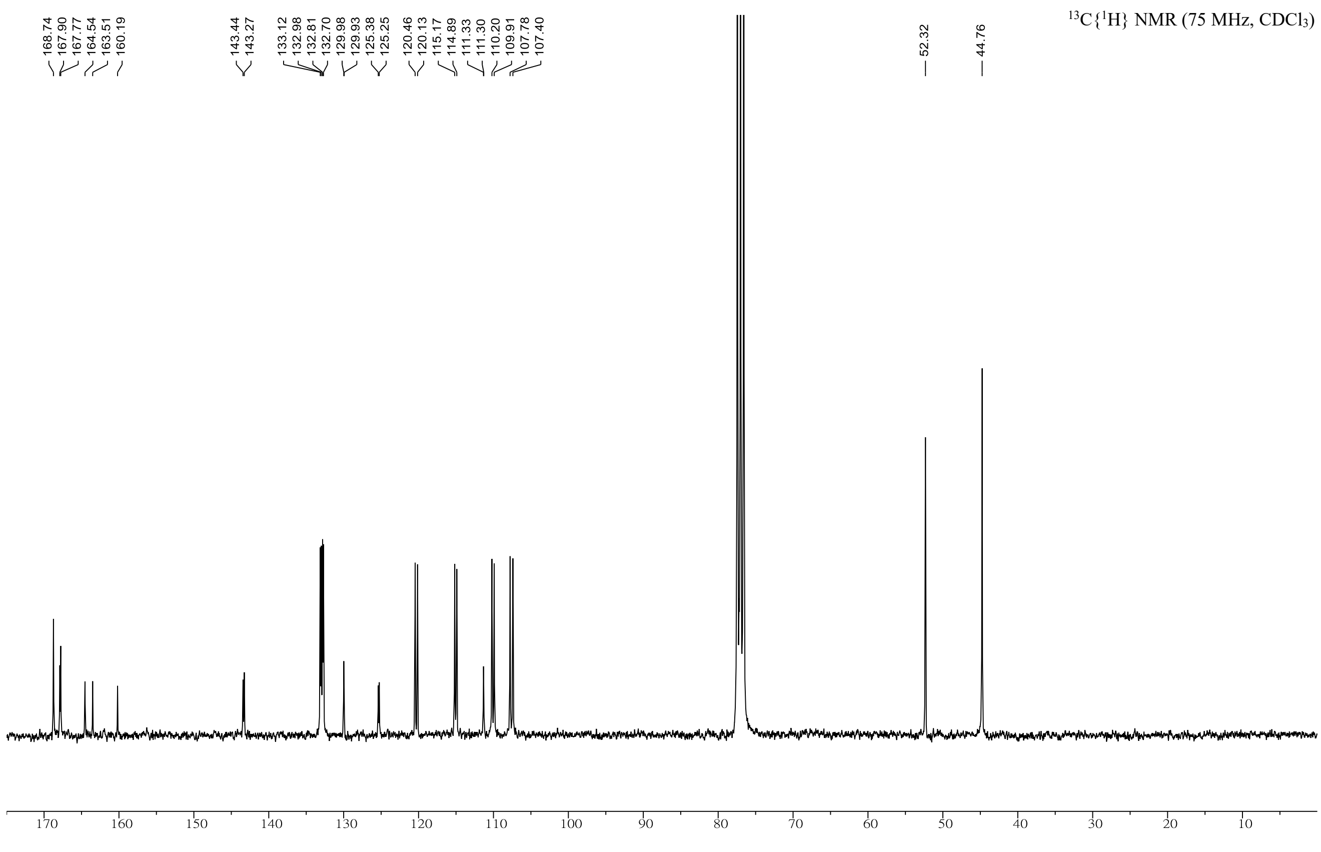

Methyl 2-[2-(2-Bromo-5-chlorophenyl)acetamido]-4-fluorobenzoate (4y)

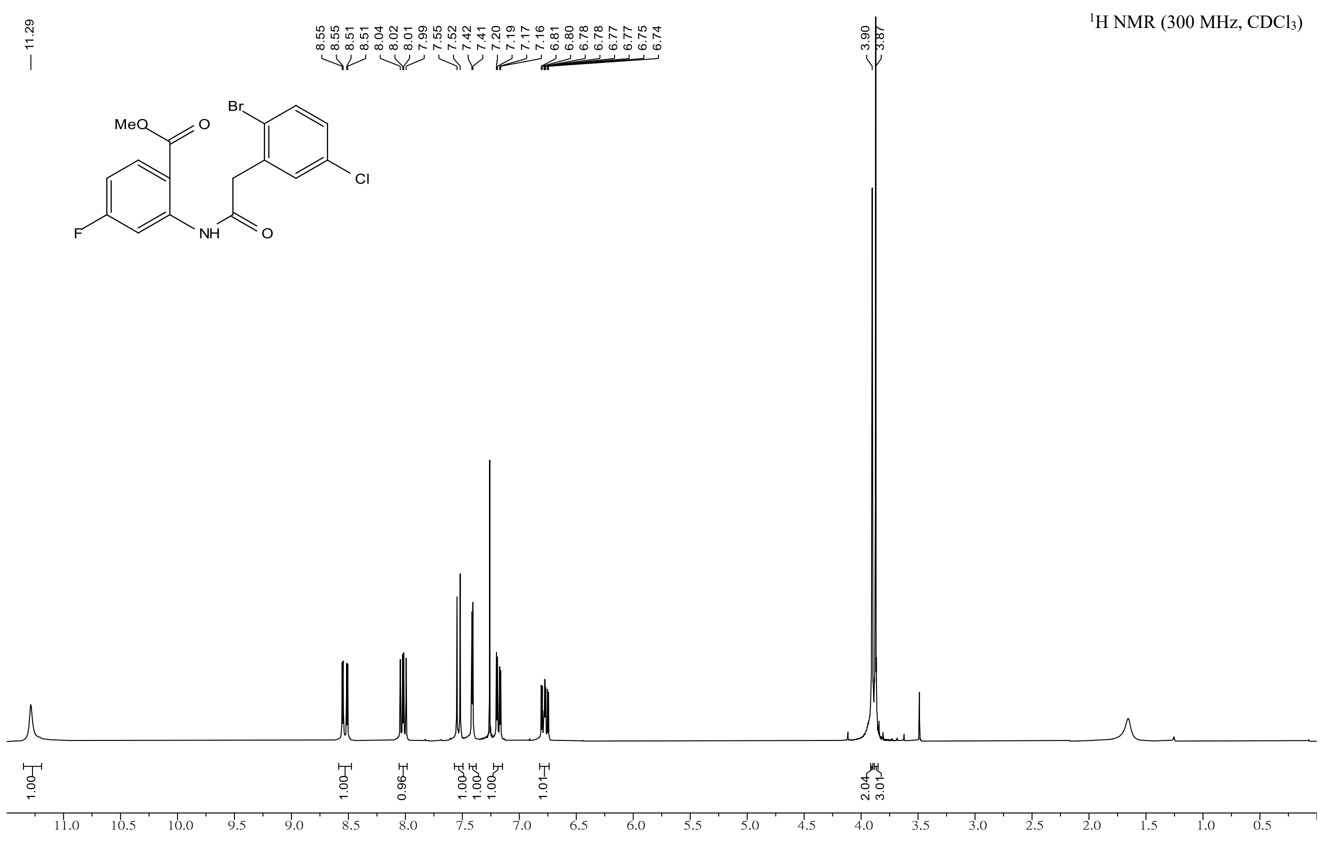

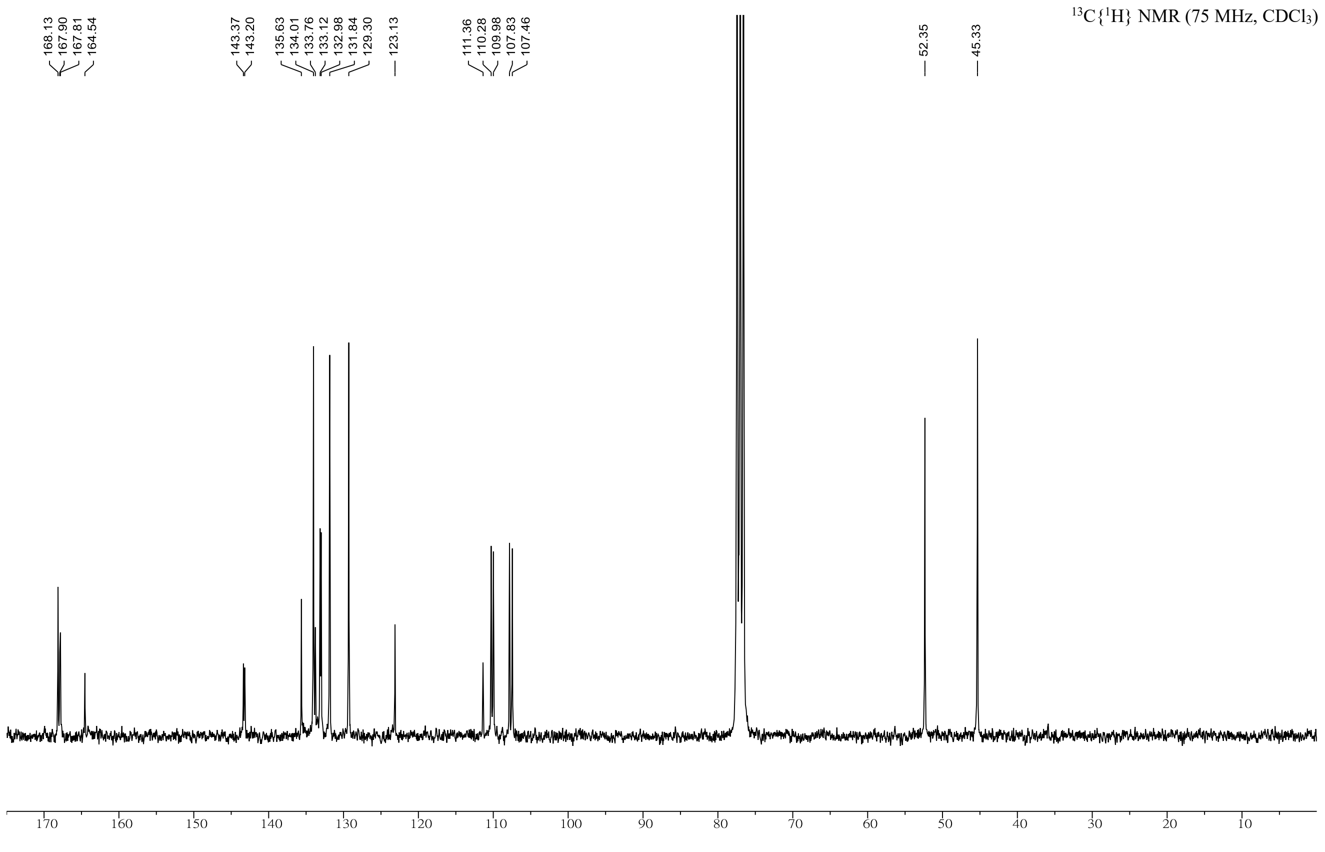

Methyl 2-[2-(2-Bromo-5-chlorophenyl)acetamido]-4-chlorobenzoate (4z)

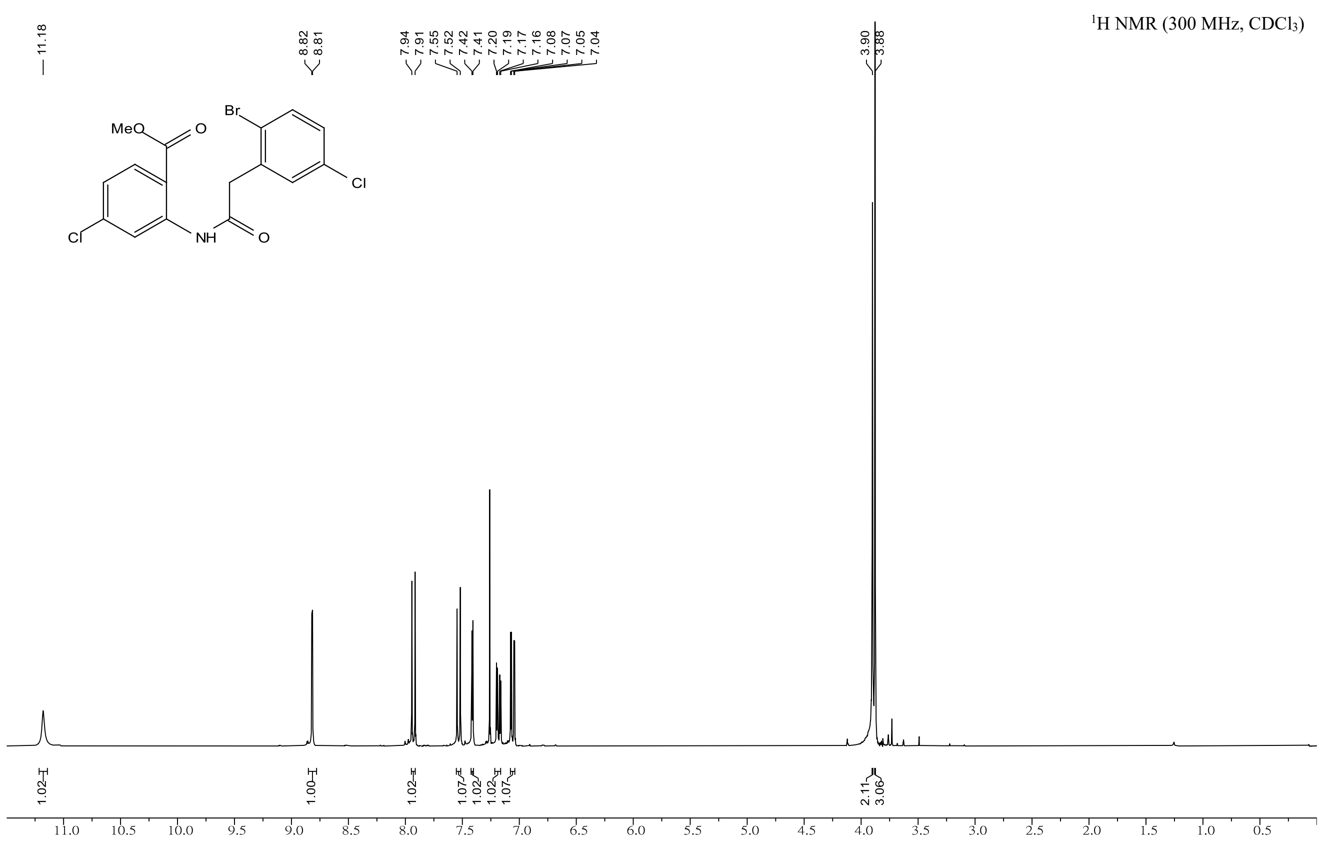

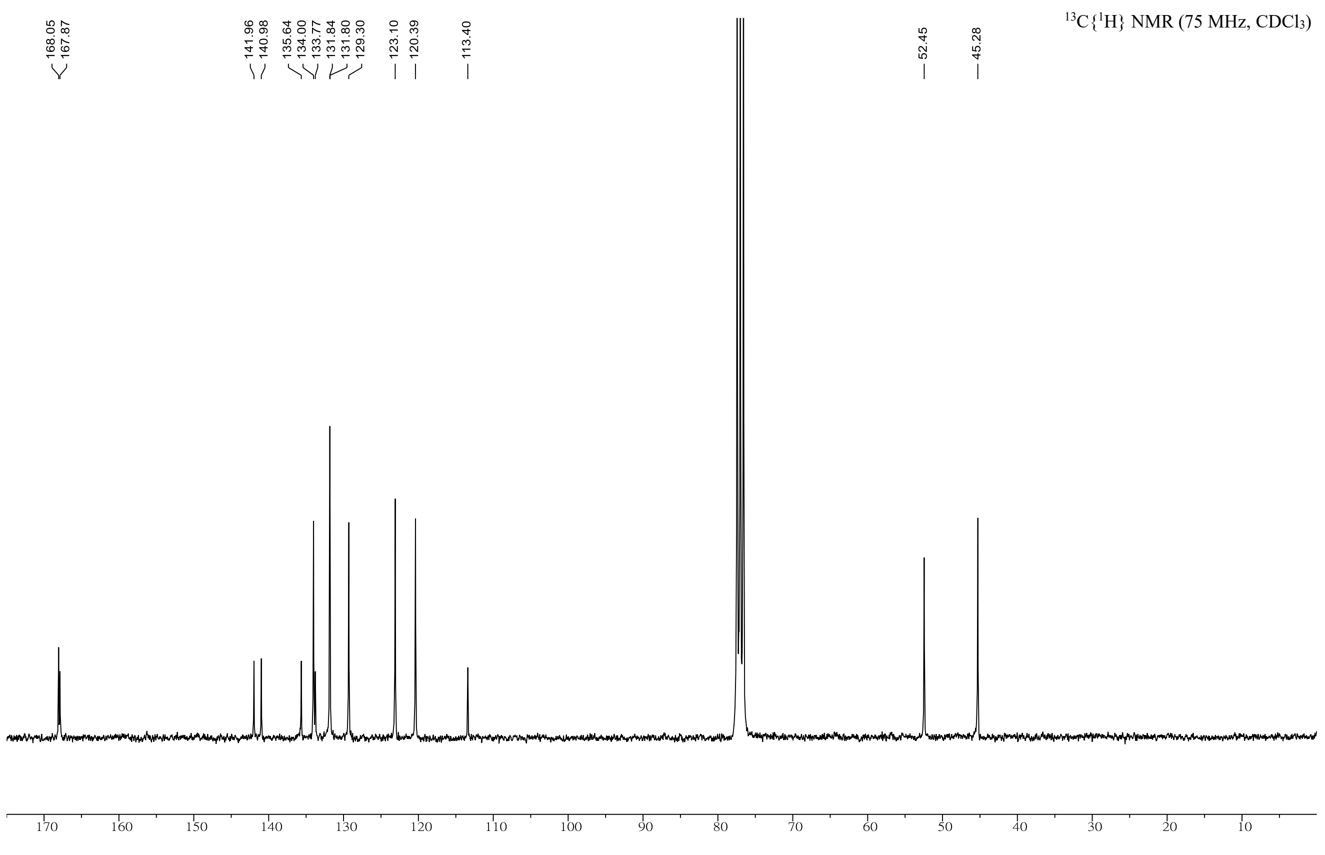

Methyl 2-[2-(2-Bromo-4,5-dimethoxyphenyl)acetamido]-4-chlorobenzoate (4A)

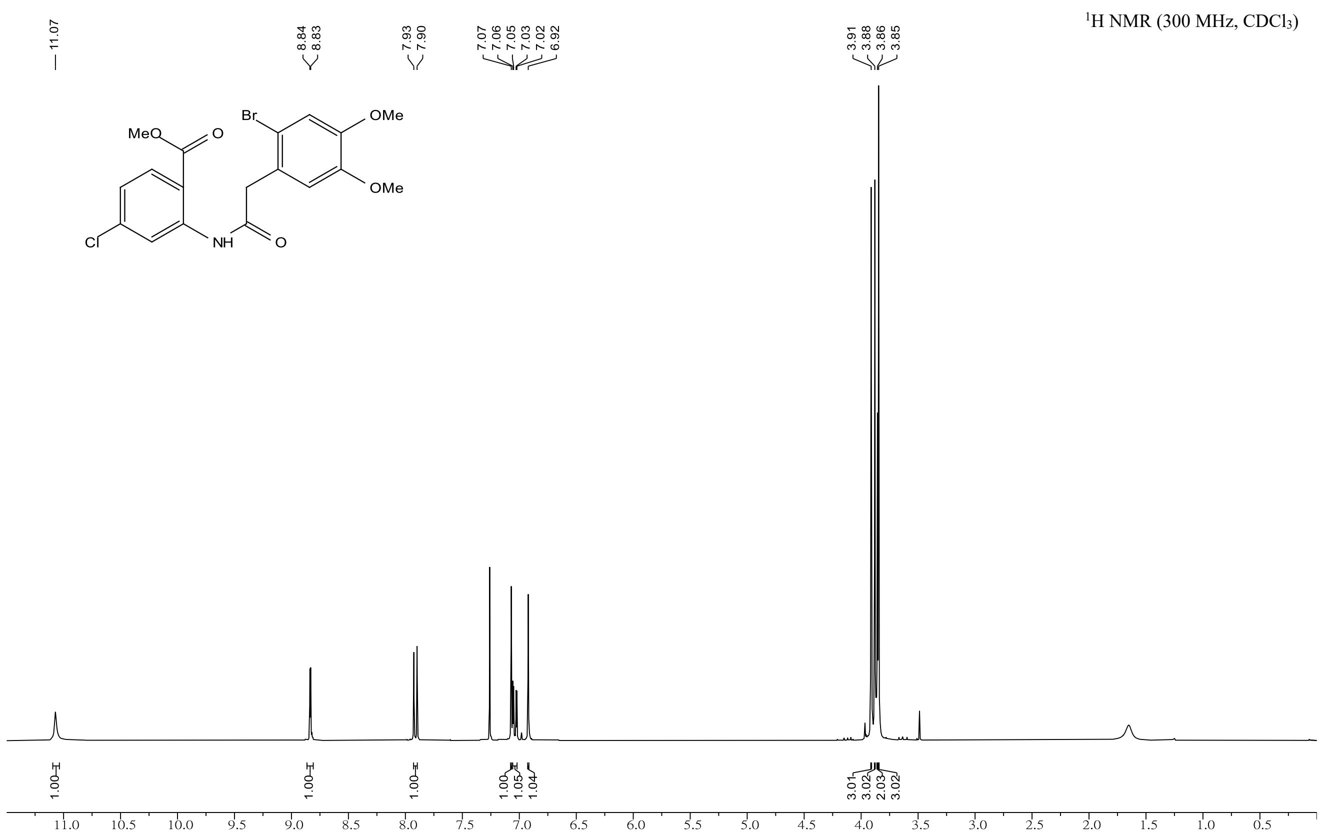

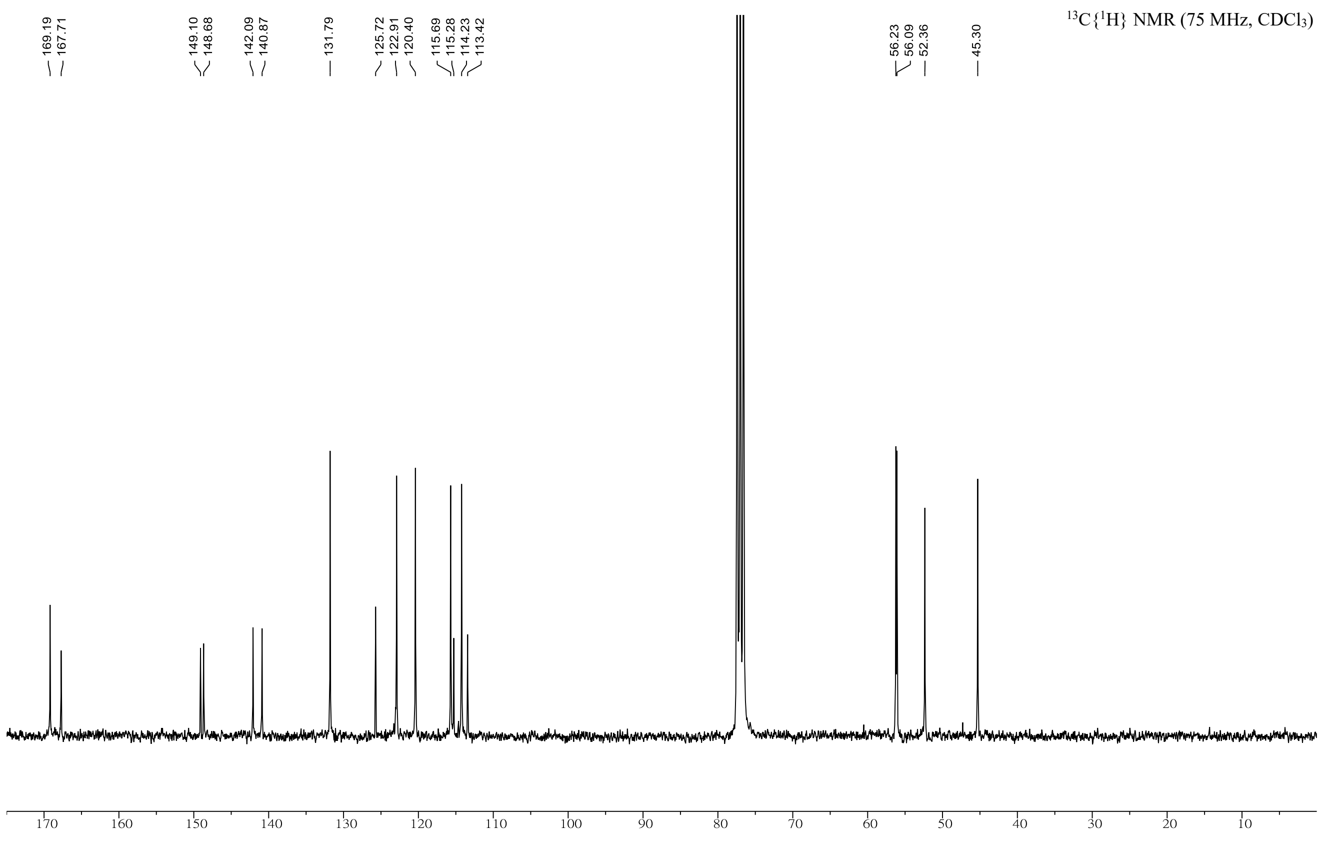

Methyl 2-[2-(6-Bromobenzo[*d*][1,3]dioxol-5-yl)acetamido]-4-chlorobenzoate (4B)

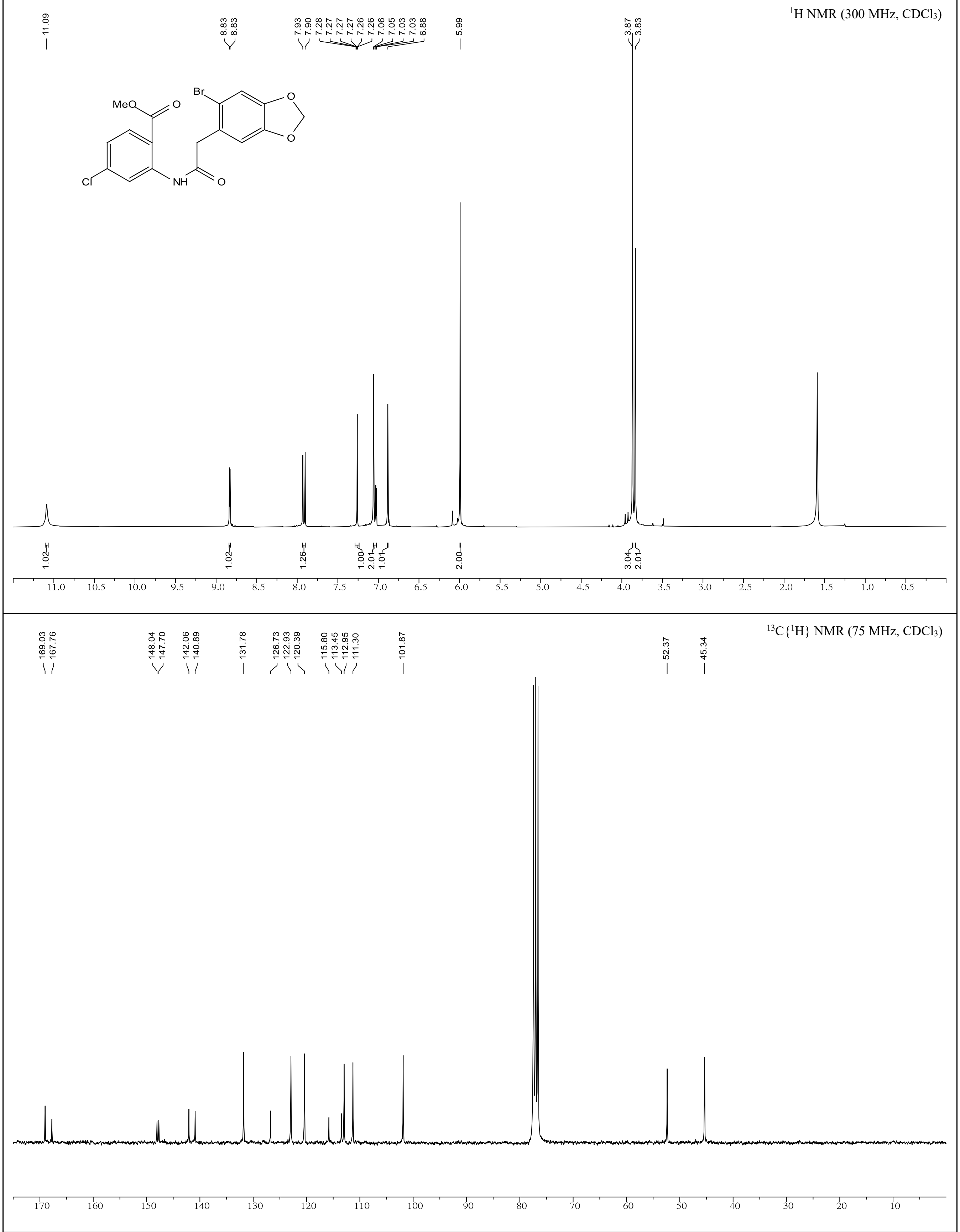

Methyl 5-Bromo-2-[2-(2,4-dibromophenyl)acetamido]benzoate (4C)

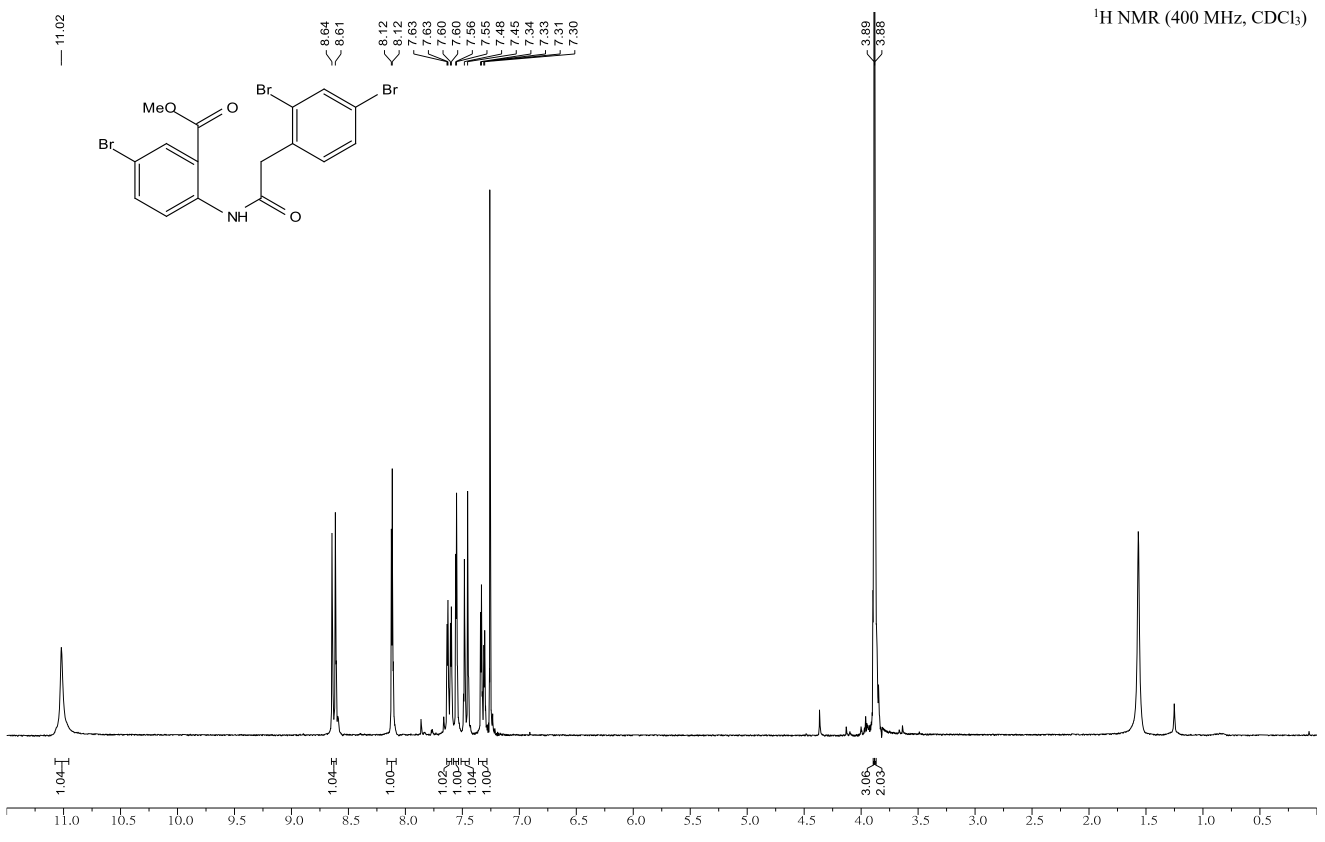

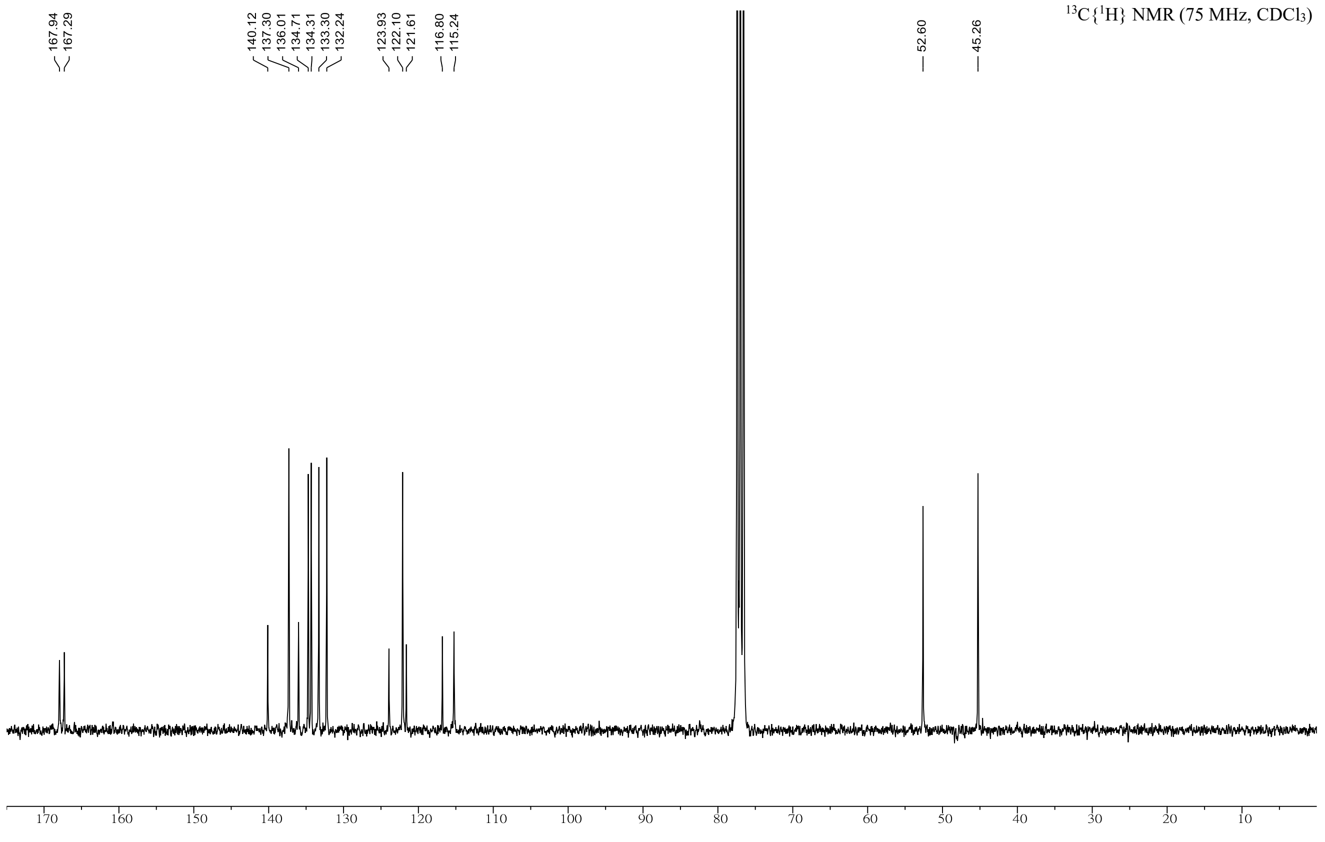

Methyl 2-[2-(2-Bromo-5-chlorophenyl)acetamido]-5-methoxybenzoate (4D)

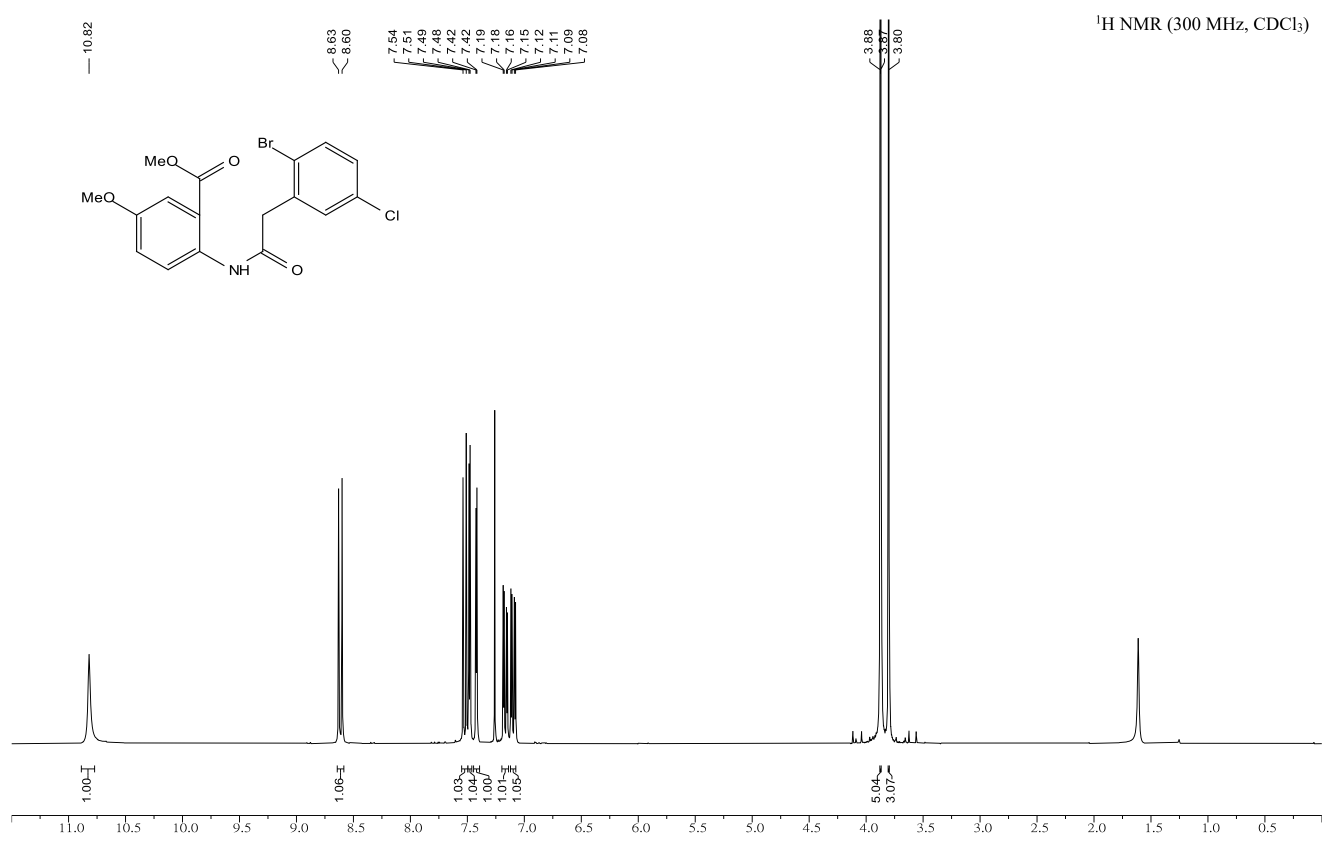

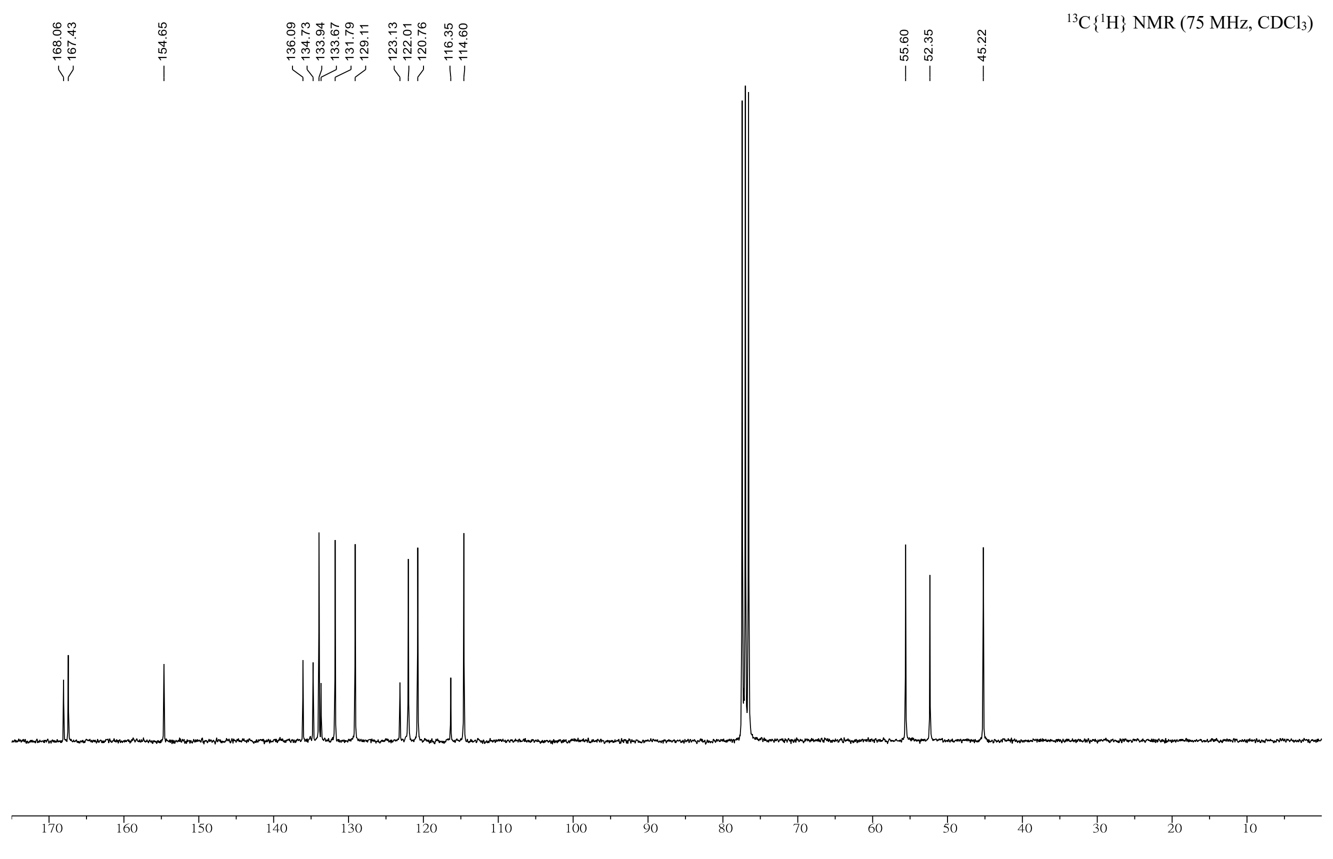

Methyl 2-[2-(6-Bromobenzo[*d*][1,3]dioxol-5-yl)acetamido]-4,5-dimethoxybenzoate (4E)

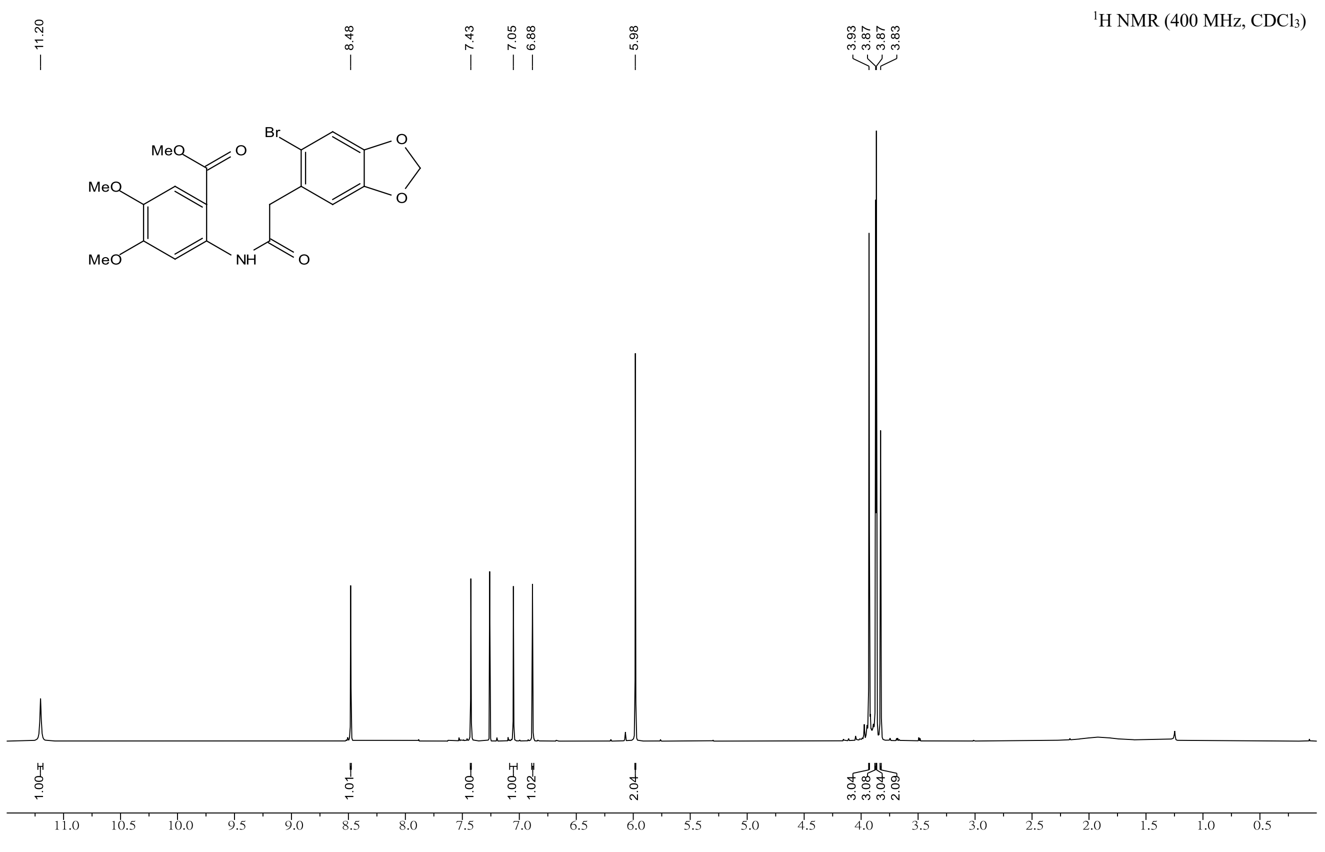

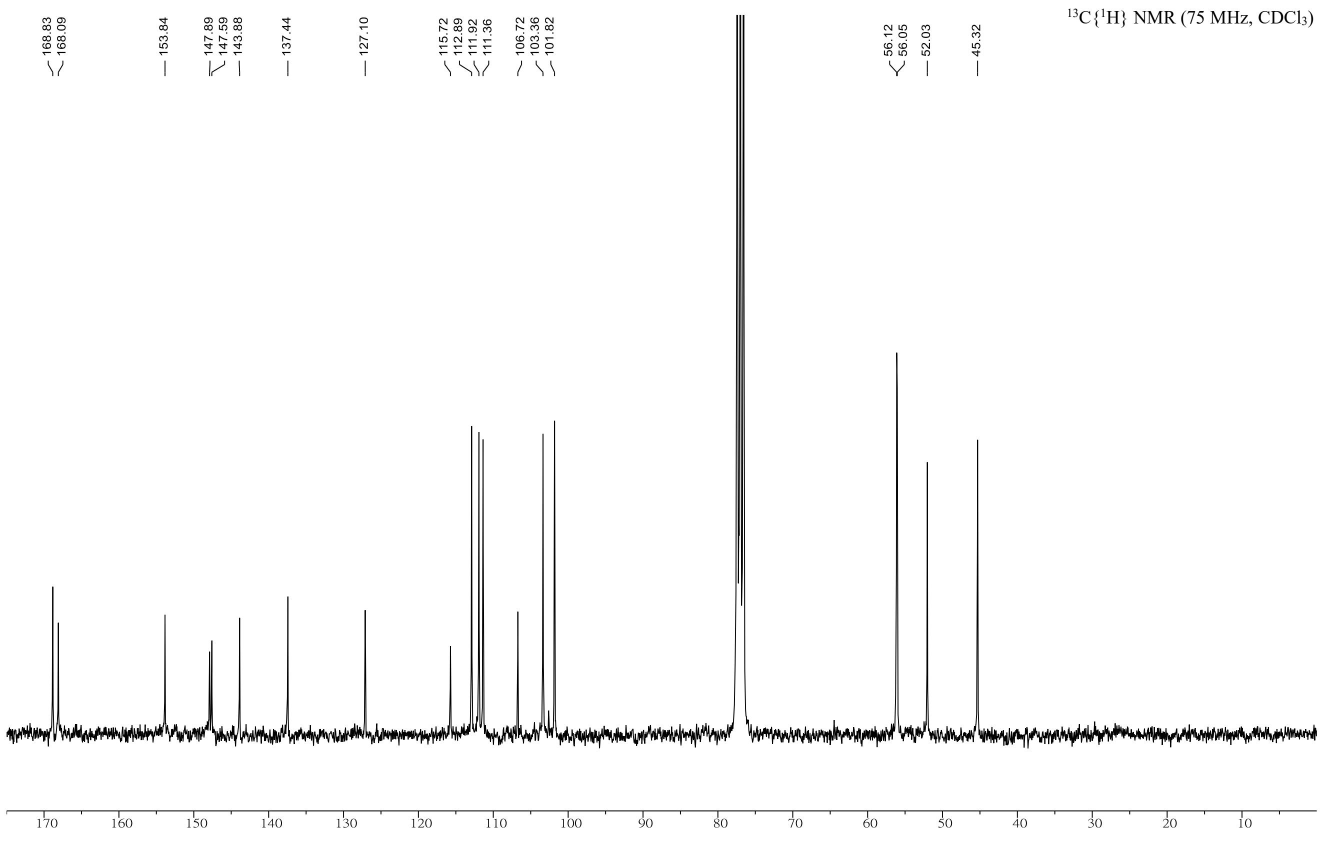

Supplement: Supplementary file 1 — jo4c02813_si_001.pdf [file jo4c02813_si_001.pdf]
